# Two Decades of Air Pollution Health Risk Assessment: Insights From the Use of WHO’s AirQ and AirQ+ Tools

**DOI:** 10.3389/phrs.2024.1606969

**Published:** 2024-06-18

**Authors:** Heresh Amini, Fatemeh Yousefian, Sasan Faridi, Zorana J. Andersen, Ellénore Calas, Alberto Castro, Karla Cervantes-Martínez, Thomas Cole-Hunter, Magali Corso, Natasa Dragic, Dimitris Evangelopoulos, Christian Gapp, Mohammad Sadegh Hassanvand, Ingu Kim, Alain Le Tertre, Sylvia Medina, Brian Miller, Stephanie Montero, Weeberb J. Requia, Horacio Riojas-Rodriguez, David Rojas-Rueda, Evangelia Samoli, Jose Luis Texcalac-Sangrador, Maayan Yitshak-Sade, Joel Schwartz, Nino Kuenzli, Joseph V. Spadaro, Michal Krzyzanowski, Pierpaolo Mudu

**Affiliations:** ^1^ Department of Environmental Medicine and Climate Science, Icahn School of Medicine at Mount Sinai, New York, NY, United States; ^2^ Institute for Climate Change, Environmental Health, and Exposomics, Icahn School of Medicine at Mount Sinai, New York, NY, United States; ^3^ Department of Environmental Health Engineering, Faculty of Health, Kashan University of Medical Sciences, Kashan, Iran; ^4^ Center for Air Pollution Research (CAPR), Institute for Environmental Research (IER), Tehran University of Medical Sciences, Tehran, Iran; ^5^ Department of Public Health, University of Copenhagen, Copenhagen, Denmark; ^6^ University of Paris-Saclay, Saint Aubin, France; ^7^ Swiss Tropical and Public Health Institute, Allschwil, Switzerland; ^8^ University of Basel, Basel, Switzerland; ^9^ Department of Environment, Climate Change and Health, World Health Organization, Geneva, Switzerland; ^10^ Department of Environmental and Occupational Health, Santé Publique France, Saint-Maurice, France; ^11^ Faculty of Medicine, University of Novi Sad, Novi Sad, Serbia; ^12^ Environmental Research Group, MRC Centre for Environment and Health, Imperial College London, London, United Kingdom; ^13^ World Health Organization Regional Office for Europe, Copenhagen, Denmark; ^14^ European Centre for Environment and Health, World Health Organization, Regional Office for Europe, Bonn, Germany; ^15^ Regional Office Bretagne, Santé Publique France, Rennes, France; ^16^ Institute of Occupational Medicine (IOM), Edinburgh, United Kingdom; ^17^ Clean Air Institute, Washington, DC, United States; ^18^ Center for Environment and Public Health Studies, School of Public Policy and Government, Fundação Getúlio Vargas, Brasília, Brazil; ^19^ Department of Environmental Health, National Institute of Public Health, Cuernavaca, Mexico; ^20^ Department of Environmental and Radiological Health Sciences, Colorado State University, Fort Collins, CO, United States; ^21^ Colorado School of Public Health, Colorado State University, Fort Collins, CO, United States; ^22^ Department of Hygiene, Epidemiology and Medical Statistics, Medical School, National and Kapodistrian University of Athens, Athens, Greece; ^23^ Department of Environmental Health, Harvard T.H. Chan School of Public Health, Boston, MA, United States; ^24^ Spadaro Environmental Research Consultants (SERC), Philadelphia, PA, United States; ^25^ School of Public Health, Imperial College London, London, United Kingdom

**Keywords:** AirQ, air pollution, burden of disease, health risk assessment, WHO

## Abstract

**Objectives:**

We evaluated studies that used the World Health Organization’s (WHO) AirQ and AirQ+ tools for air pollution (AP) health risk assessment (HRA) and provided best practice suggestions for future assessments.

**Methods:**

We performed a comprehensive review of studies using WHO’s AirQ and AirQ+ tools, searching several databases for relevant articles, reports, and theses from inception to Dec 31, 2022.

**Results:**

We identified 286 studies that met our criteria. The studies were conducted in 69 countries, with most (57%) in Iran, followed by Italy and India (∼8% each). We found that many studies inadequately report air pollution exposure data, its quality, and validity. The decisions concerning the analysed population size, health outcomes of interest, baseline incidence, concentration-response functions, relative risk values, and counterfactual values are often not justified, sufficiently. Many studies lack an uncertainty assessment.

**Conclusion:**

Our review found a number of common shortcomings in the published assessments. We suggest better practices and urge future studies to focus on the quality of input data, its reporting, and associated uncertainties.

## Introduction

Air pollution (AP) is a significant health risk, leading to a range of diseases and premature deaths [1, 2]. In 2019, AP, particularly from fine particulate matter (PM_2.5_) and household solid fuels, was linked to approximately 6.45 million early deaths globally, with ambient air pollution (AAP) accounting for about 4.1 million [2]. Health risk assessment (HRA) studies have reported similar or higher figures [3, 4]. These estimates vary based on factors like study design, population, AAP exposure, choice of concentration-response function [CRF; also called exposure-response functions (ERF)], study period, health outcomes, and counterfactual values. However, they all follow a common HRA concept [5].

Concerns about AP have led to the development of methods to assess its health impacts and predict changes due to varying AP levels. This data aids in policy-making to mitigate AP. Scientists agree on these methods, which are implemented via spreadsheets and integrated into user-friendly tools. These tools, used by scientists, policy analysts, NGOs, and the public, facilitate the HRA of AP [6]. The WHO reported on several HRA tools in 2016, but few had a long maintenance history. Notable exceptions are WHO’s AirQ software and the US EPA’s BenMAP program, both released in the early 21st century, which quantify the health effects of ambient air pollution (AAP) [7].

AirQ has been one of the most extensively used software owing to its user-friendly interface, and technical and operational characteristics [6, 8, 9]. The AirQ software was first developed as a spreadsheet program in 1999. AirQ version 2.2.3, was published in 2004, and was replace by AirQ+ 1.0 in May 2016 (most recent update is AirQ+ 2.2.4 in March 2023) [10]). The comparison of studies over a period of more than two decades was possible although the key functionality and algorithms in all releases remained unchanged, thus, enabling comparability of assessments over a period. AirQ and AirQ+ estimate the effects of short-term changes in AAP (based on risk estimates from time-series studies), and the effects of long-term exposure (based on risk estimates from cohort studies). The releases differ in the operating systems supported, the user interface, and default settings. For instance, AirQ+ does not provide default baseline incidence (BI) unlike AirQ [10].

There are four key pieces of input information necessary to estimate the health effects of AAP using computer-based tools [6, 7, 9]: [1] AP data [2]; demographic data of the exposed population [3]; health-related data including baseline death and disease rates; and [4] a CRF/ERF based on epidemiological studies. Publicly available HRA tools like AirQ and AirQ+ can be used by individuals with varying expertise, potentially leading to unreliable results due to inaccurate input parameters. This paper aims to review HRAs of AP using these tools, as reported in scientific literature until December 31, 2022, and provide best practice guidance for future assessments.

## Methods

### Search Strategy

**FIGURE 1:**
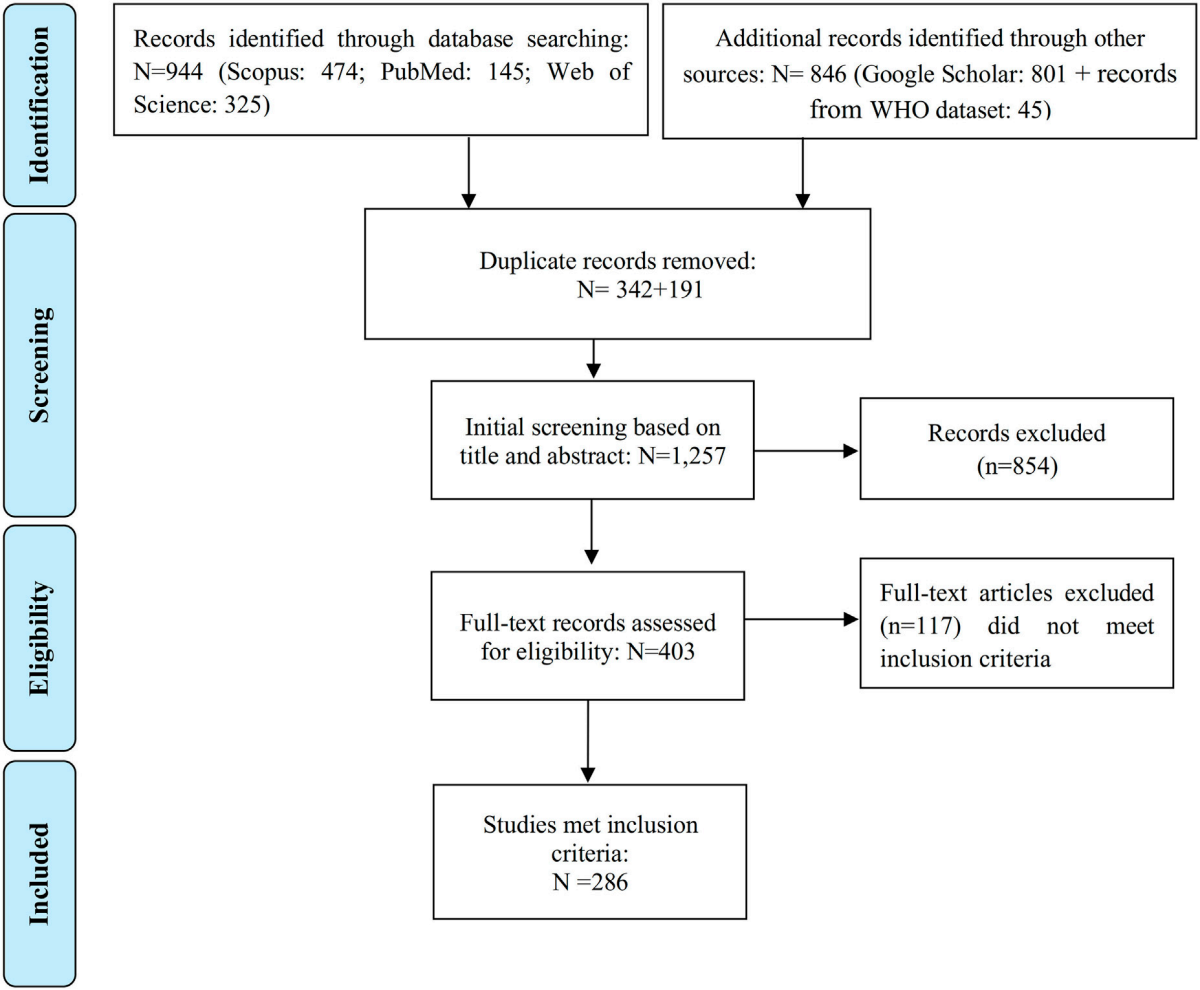
Flowchart demonstrating the steps involved in the literature search (Global, no restriction on start date-2022).

We conducted a systematic literature review following the Preferred Reporting and Items for Systematic Review and Meta-Analysis (PRISMA) (Figure 1) [11]. We reviewed literature published in print and indexed in databases from inception until 31 December 2022. The literature search was performed systematically in PubMed, Web of Science Core Collection, and Scopus using three key components (“exposure,” “health effects,” and “software”) along with their corresponding keywords (as outlined in Supplementary Table S1). Furthermore, we conducted a search on Google Scholar, albeit not in a completely systematic manner, to find studies that were not listed in the aforementioned bibliographic databases [12]. Finally, we hand-searched records identified in the references of retrieved papers [12].

We also used a database from the WHO Regional Office for Europe in which the studies estimating the health effect of AAP using AirQ (all versions of the software) were collected (these have not been collected in a systematic manner as well). The WHO database included many non-English publications, such as reports and theses that complemented our final database.

### Study Inclusion and Exclusion Criteria

Studies were included without language restriction if: 1) they were full-length peer-reviewed original articles, reports, or published theses; and 2) they reported on classical regulated air pollutants (PM_10_, PM_2.5_, NO_2_, O_3_, SO_2_, and CO) using AirQ 2.2.3, its previous versions and AirQ+. Studies were excluded if no full-text was available (e.g., conference abstracts).

### Article Selection and Data Extraction

Two co-authors (F.Y. and S.F.) independently screened titles and abstracts of all articles identified in the literature search. Duplicates were removed. If the title and abstract of papers did not provide sufficient detail for a decision, the full text of the articles was reviewed. Any discrepancies in the decision to include a study between them were resolved by a third co-author (H.A.), with discussion until a consensus was reached. The data extracted included year of publication, location (city or country of the study), WHO region, study duration, health outcome(s) addressed, air pollutants used (type and source of air quality data), duration of exposure (long- or short-term), baseline incidence (BI), and the relative risk data. We also recorded the results of our critical evaluation of the papers considering the following research questions (RQ):

• (RQ1): Was the source of air quality data provided? If yes, which types of data were used (air quality monitoring stations (AQMSs), self-measured, AQMSs + modeling, or satellite data?), and if AQMSs were the data source, what type (s) of AQMSs (e.g., traffic, background, etc.) were used?
• (RQ2): Were the air quality data coverage (daily or hourly) and their data processing/validation described?
• (RQ3): Were the exposed population and the source of their demographic data reported?
• (RQ4): Was there any information about the source of the health data? If yes, was the information extracted from an international, national, or local database?

## Results

### Literature Review Results

The initial search provided 944 records (Figure 1). We further identified 801 records from the Google Scholar database, and an additional 45 records were extracted from the WHO database (articles, reports, theses, and book chapters not identified by bibliographic databases and Google Scholar search). After removing 533 duplicate records out of the total 1,790 initial records, the titles and abstracts of the remaining 1,257 papers were reviewed. Out of these, 854 were found to be unrelated to the purpose of the study and were further excluded. Full texts of the remaining 403 records were reviewed, from which 286 met the inclusion criteria.

Of those 286 selected records identified as meeting the inclusion criteria (241 from the PubMed, Scopus, Web of Sciences, and Google Scholar databases, plus 45 from the WHO database), 241 were published in scientific peer-reviewed journals, or as theses. Most of the publications were in English (Supplementary Figure S1), while some studies were in Persian [13], Italian [14], Spanish [6], French [3], German [2], Polish [2], Czech [2], Estonian [2], Turkish [2], Croatian [1], Portuguese [1], Japanese [1], and Hungarian [1]. The Persian records were assessed by two co-authors who are Persian native speakers (F.Y. and S.F.), while the papers published in other languages were assessed by one of the authors (P.M. supported by experts who could extract information from other languages). Supplementary Tables S2, S3 provide a summary of the included studies that used AirQ and AirQ+ for HRA from 1 January 2002, to 31 December 2022.

### Publications by Year

**FIGURE 2:**
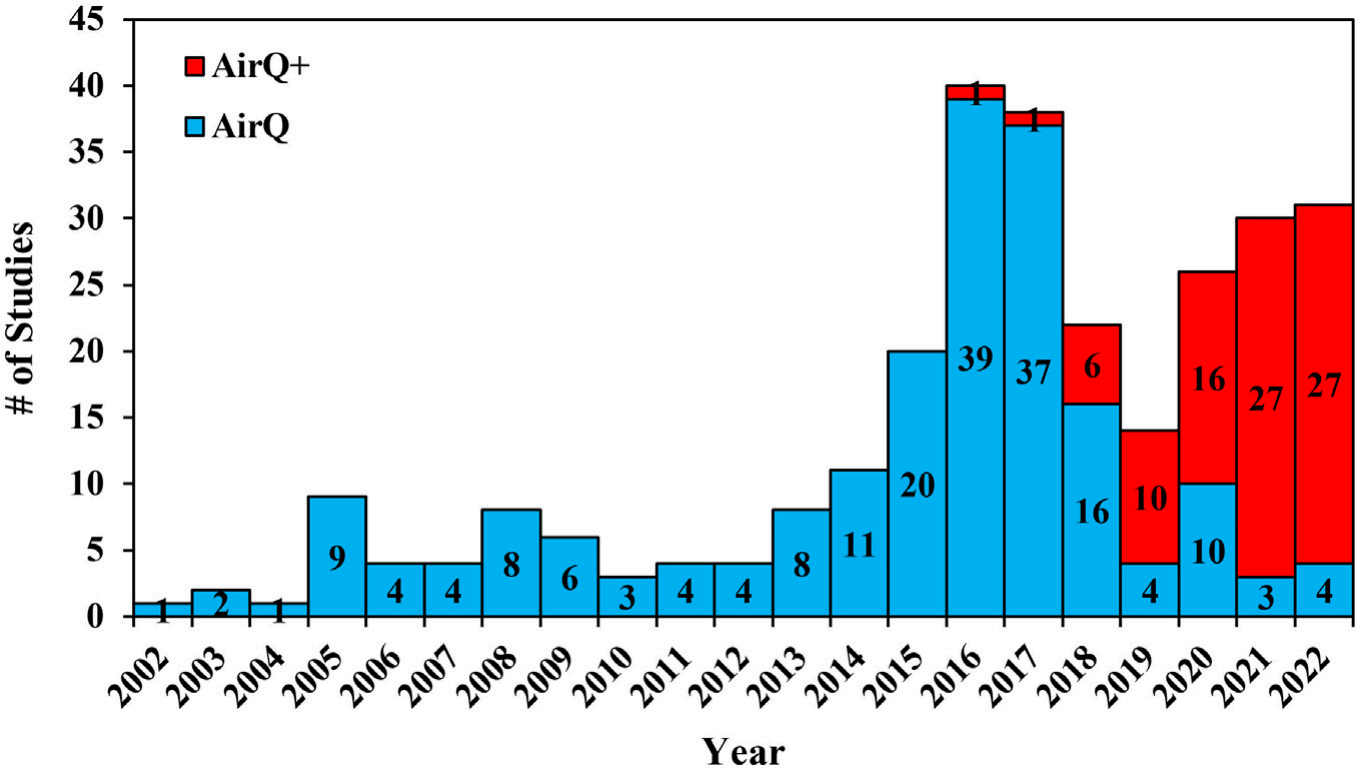
Temporal distribution of publications using all versions of AirQ and AirQ+ software (Global, 2002–2022).

The temporal distribution of the included studies using AirQ and AirQ+ between 2002 and 2022 is presented in Figure 2. Out of the 286 selected publications, 198 used AirQ and 88 used AirQ+. The year with the largest number of publications was 2016 for AirQ (39 publications), and 2021 for AirQ+ (27 publications), representing approximately 20% and 31% of the total publications, respectively. The start of the decline in publications using AirQ from 2016 onwards is likely due to the release of AirQ+ in that year. However, even in 2021 and 2022 AirQ was used in a few studies [14–23]. One study reported that they had used AirQ+ (v3.0) to estimate health effects in cities of France, Iran, and Italy, but such a version has not been released by WHO to date, and it is possible that it was a typo.

### Publications by Country and WHO Region

**FIGURE 3:**
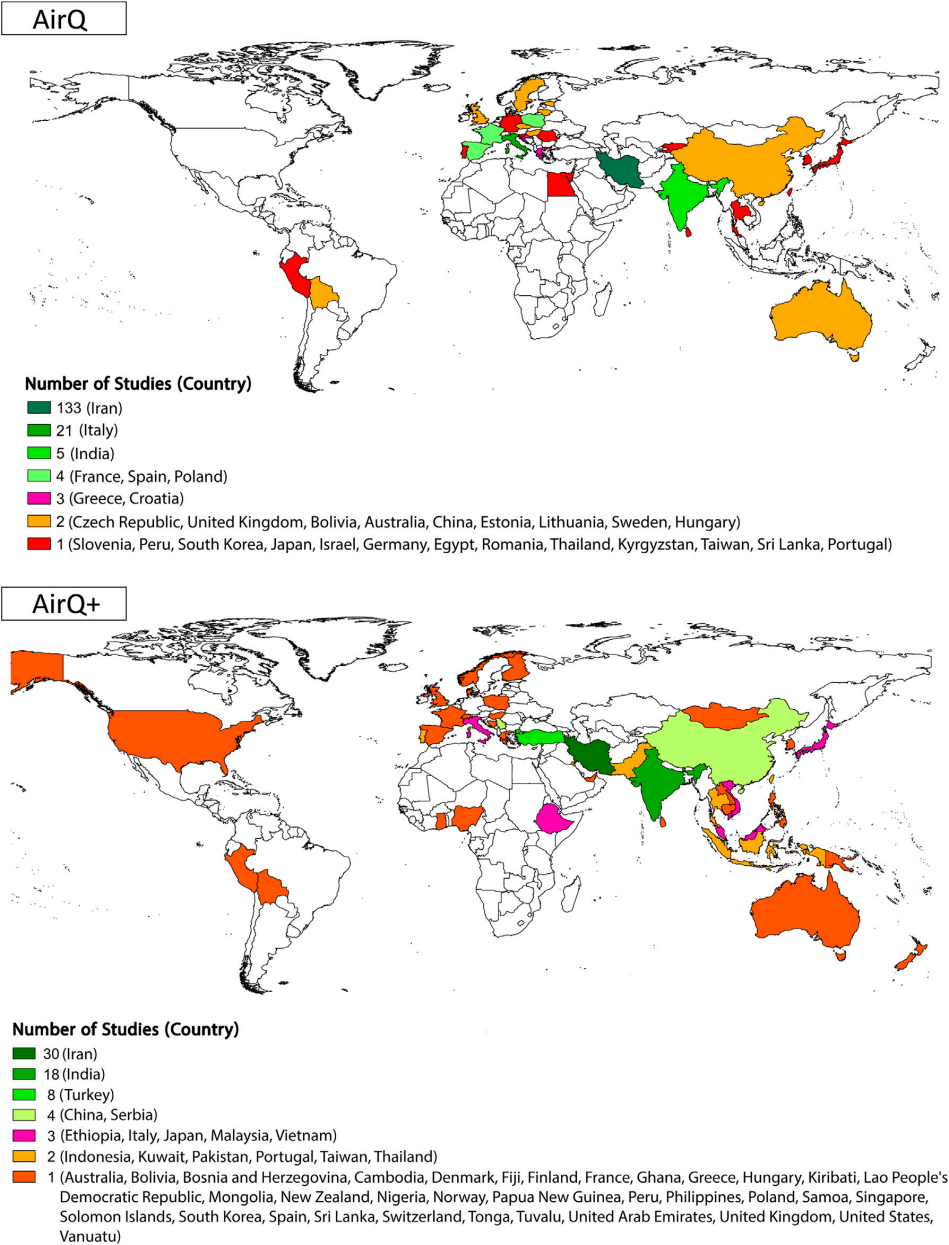
Geographic distribution and number of included studies by country [AirQ at the top and AirQ+ at the bottom; (Global, 2002–2022)].

More than half (57% out of 286) of the studies that used AirQ or AirQ+ were conducted in Iran (Figure 3; Supplementary Figure S2). For AirQ, out of the final studies included, 133 (67%) focused on Iran, followed by Italy (20 studies, 10%), India (five studies, 2.5%), France, Spain, Poland, and Croatia (three studies each). Further, 14 studies used AirQ in Austria, Bolivia, China, Czechia, Estonia, Greece, and Lithuania (two studies in each country). AirQ was used in one study in each of the following countries: Egypt, Germany, Hungary, Japan, Kyrgyzstan, Peru, Portugal, South Korea, Sri Lanka, Sweden, Thailand, Taiwan, and the UK. One study estimated the health effects of AAP in 23 European and Middle Eastern cities in Greece, Spain, France, Romania, Hungary, Slovenia, Poland, Sweden, England, Italy, and Israel (Figure 3; Supplementary Figure S2). AirQ has been applied primarily in the WHO Eastern Mediterranean Region (67% of publications), followed by Europe (25%), Southeast Asia (4%), Western Pacific (3%), and the Americas (1%) region (Supplementary Figure S3). In those studies that used the AirQ+ software, 30 (34%) of studies focused on Iran, followed by India and Turkey with 18 and eight studies, respectively (Figure 3; Supplementary Figure S2). Compared to AirQ, a smaller proportion of AirQ+ users were from the WHO Eastern Mediterranean Region (30% of records), while there has been a substantial rise in the contribution of Western Pacific Region (25% in AirQ+ vs. 3% in AirQ) and South-East Asian Region (18% for AirQ+ vs. 4% for AirQ) (Supplementary Figure S3). Although the tool is widely used in many Central and South American countries (a Spanish version of the software was launched in February 2024), and is extensively applied in France, there is a clear sub-representation of the publications that have applied AirQ+.

### Motivation of Conducted Studies

Around three-quarters of the studies using either AirQ or AirQ+ focused on research-related questions (Supplementary Figure S4, Supplementary Tables S2, S3), followed by policy planning (2% of studies that used AirQ and 15% for AirQ+), the impact assessment of sand and dust storms in various cities of Iran and Poland (14 studies using either AirQ or AirQ+) [15, 23–35], and assessment of the impact of COVID-19 on air quality and health (5 studies) [36–40]. Individual studies assessed the impact of AP episodes or extreme events, such as (Indian monsoon [13, 31] or large forest fires and megafires in the center region of Portugal [41]), and the use of residential wood combustion in cities of Sweden, Finland, Norway, and Denmark [42]. There is also an instance of the use of AirQ, on behalf of a civil society association, to conduct a study in opposition of the planned development of an incinerator in the Provincia di Lucca (Tuscany) in Italy [43].

### Exposure Assessment

#### Air Quality Data

**FIGURE 4:**
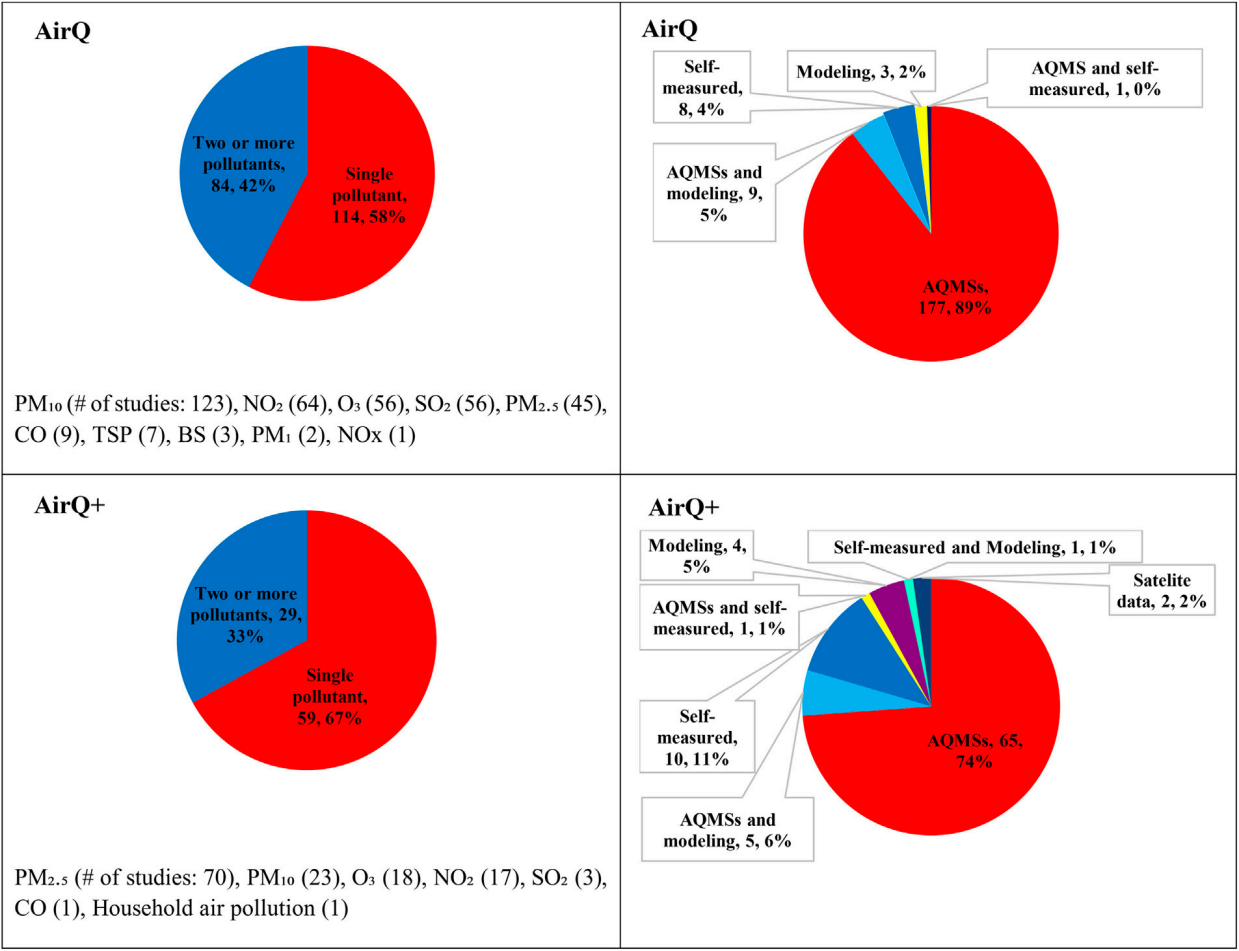
Numbers (percentage) of included studies using AirQ and AirQ+ software by air pollutant, and the source of air pollution data (Global, 2002–2022).

Since the key functionality and algorithms have not changed from AirQ to AirQ+, we assumed that differences in the use of the various software releases reflect changes in knowledge on AP health effects and research interests over time. Figure 4 illustrates the specific pollutants addressed in the selected studies from 2002 to 2022. From 2002 to 2022, most studies using these tools focused on the health effects of a single pollutant, with PM_10_ being the most studied in AirQ and PM_2.5_ in AirQ+. However, some studies investigated the effects of multiple pollutants. This shift towards PM_2.5_ reflects its increased data availability and recognized health impacts [44, 45]. The health impacts of PM_10_, O_3_, and NO_2_ were estimated by 23, 18 and 17 AirQ+ studies, respectively.

All studies stated the source of the AAP data that were used. Most of the AirQ studies (187) used ground-monitored air pollution concentrations from the regulatory monitoring network stations alongside modeled and self-measured data, while 8 studies reported they have conducted their own measurements [35, 46–52]. All but six studies that applied AirQ did not report which type of AQMSs were used [47, 53–57]. For AirQ+, 74% of the studies (65 out of 88) gathered AAP data from the AQMSs, and only 16 studies reported the types of AQMSs used (Supplementary Tables S2, S3) [25, 31, 36, 39, 41, 58–68].

Almost three-quarters of studies that used AirQ did not report the temporal coverage of the data (Supplementary Figure S5). The remaining studies reported that they only used the AQMS with >75%, 60%, or 50% completeness of the total hours in a year to calculate the short- or long-term exposure to AAP (Supplementary Table S2). Approximately, half of AirQ+ studies (40 out of 88) stated their AAP data coverage (Supplementary Figure S5). These studies mostly used the AQMSs with >75% completeness over a year to estimate the HRA of short- or long-term exposure to AP (Supplementary Table S3).

Nearly 80% of the studies that used AirQ or AirQ+, did not report air quality data processing and validation approaches (Supplementary Figure S6). As shown in Supplementary Tables S2, S3, the remaining studies that provided this information removed zero, negative, invalid and outlier values from the data set [25, 60, 64, 68–75], though none of them reported how the invalid or outlier data were identified. On the other hand, a few studies used a Z-score approach, which is a variation of scaling methods [8, 76–79], and defined other rules to identify and remove outlier values [76, 80].

#### Choice of Relative Risk (RR)

The RRs that have been applied to calculate the health risks attributed to air pollutants can be classified into the following five categories (Supplementary Figure S7; Supplementary Table S2):

• Previous study values: 74 studies (39%) reported that they utilized RR values from previous assessments. Furthermore, 5 studies used previous values of RR, AirQ default values, and RRs derived from European studies (like the APHEA study mentioned further below) in the same assessment. It should be highlighted that 58 of the 74 studies utilized a RR from the previous ones were conducted by Iranian researchers. Studies conducted by Iranian researchers mainly used the RR from the study of Naddafi and others (2012) [81], in which the authors have used the default RR in the AirQ software, a quantitative meta-analysis of peer-reviewed studies focused on European investigations, or directly from published studies on short-term effects, such as the APHEA (Air Pollution and Health: a European Approach) project. For example, two studies [54, 82] stated: “*The RR values for ambient PM*_*2.5*_ *and PM*_*10*_ *were “borrowed” from a meta-analysis of 23 European cities because there has been no study for RR calculation in Iran.*” The rest of the studies [18] reported that RRs were extracted directly from literature (e.g., APHEA) or were the recommended AirQ default RRs.
• AirQ software default values: Almost 25% of the studies have reported that they used the AirQ software default RR values.
• WHO study (meta-analysis and epidemiological), report, or database values: Approximately 23% of the studies (44 studies of which 38 were in Iran) used RR reported from WHO studies (meta-analysis and epidemiological ones), reports [83], and databases as the source of their RR values.
• Used national or local/regional values: About 2% of the studies used national and local/regional RR values from a single specific study.
• No data reported: 12% of the studies did not report the sources of RR values.

When comparing AirQ with AirQ+ studies in terms of RR (Supplementary Figure S7; Supplementary Table S3), 92% of AirQ+ studies have used the default RR values in the software; 83% of them stated that they used the RR of AirQ+ software and 9% have reported that they have utilized RR from the previously published HRA articles [42, 62, 63, 67, 84–87] (Supplementary Figure S7). The study [88], for instance, stated that “*The default relative risk (RR) values available in the AirQ+ software for each health endpoint were retained for the analysis.*” Only 8% of the studies did not report the source of RR.

#### Choice of Baseline Disease Incidence (BI)

The choices for BI values across studies were as follows (Supplementary Figure S8):

• 24% of studies used national/local BI values, and 25% of the studies utilized BI values from the previous studies, regardless of assessing their validity.
• 21% of the studies reported that they utilized BI values from WHO studies, data, and reports (Supplementary Figure S8).
• 18% of the studies did not report the source of BI values they used in their assessment.
• 10% of the studies applied AirQ software default BI values for quantifying premature deaths and hospital admissions due to cardiovascular and respiratory diseases related to AP.

In contrast to AirQ, AirQ+ does not provide default BI values (Supplementary Figure S8). Seven studies used international sources like the Institute for Health Metrics and Evaluation (IHME) database, or WHO data [20, 86, 88–92]. Nearly, 55% of AirQ+ studies used BI values obtained from local or national sources, which could be a good practice if high-quality data are available locally. 18 out of 88 studies did not report the source of BI values, and 14 studies used BI values from the previous studies. In one study [93], the BI value for all-cause mortality [42] was less than the BI value for ischemic heart disease (IHD) mortality, which was reported to be 112. Other researchers have commented on this error [94].

The BI rates differ between populations as the age structure, the environmental or behavioral stressors, and susceptibility of populations, among other factors, could be different. When a default BI value from the AirQ software is used, the estimated values (e.g., of premature deaths or hospital admissions) attributable to AAP exposures could be biased, if not adjusted for demographic differences.

#### Choice of Cut-Off/Counterfactual Values

The cut-off (or counterfactual) scenario used in the HRA analysis, fall into three main categories across studies: a) the default AirQ or AirQ+ software values, b) the national ambient standards in the countries where the studies were conducted, and c) missing information as the authors did not report these values (Supplementary Figure S9; Supplementary Tables S2, S3). In more than three-quarters of the AirQ studies (77%), users have reported the cut-off values, which is slightly higher than the AirQ+ users’ reporting rate of 72%. Among the 77% of studies reporting the cut-off values, 120 studies have used the counterfactual value of 10 μg/m³ for PM_2.5_. About 58% of the studies that used either AirQ or AirQ+ software were conducted in Iran, and across these, approximately all used the software default cut-off values. The detailed information regarding counterfactual value of other air pollutants is presented in Supplementary Tables S2, S3.

#### Population Age Groups

Most studies using AirQ and AirQ+ did not detail population data, such as age groups. They reported total population without age stratification. This can affect health effect calculations, as CRF and BI values should correspond to the relevant population segment. For example, using a larger population size than the underlying true one [93] for Tehran, Iran, led to a 35% overestimation of air pollution’s health burden [94].

#### Health Endpoints

In studies using the AirQ software, 53% investigated short-term exposure effects, while 7% explored long-term health impacts. In contrast, 57% of studies using AirQ+ estimated long-term health effects, compared to 17% that focused on short-term exposure. Notably, only 6% of AirQ+ studies did not report exposure duration, a significant improvement over the 23% using the AirQ version (Supplementary Figure S11). In the AirQ studies, all but one reported the number of cases, with 79% and 81% providing population attributable fraction (PAF) and the 95% confidence interval respectively (Supplementary Figures S12–S14). Among the studies using AirQ+, all reported the number of cases and their 95% confidence interval, while 63% also included the PAF (Supplementary Figures S12–S14).

Supplementary Figure S15 shows the health endpoints assessed with the AirQ and AirQ+ software. All-cause mortality was the main health outcome assessed due to the availability of BI data, and the robust epidemiological evidence. Most studies estimated more than one health endpoint. In the AirQ studies, cardiovascular disease mortality was the most studied outcome, included by 138 studies, followed by all-cause mortality (131), respiratory mortality (116), and lung cancer (LC) mortality [4]. For studies that used the AirQ+ software, the majority investigated all-cause mortality (104 studies), followed by chronic obstructive pulmonary disease, ischemic heart disease, LC, stroke, and respiratory mortality by 39, 36, 32, 31, and 29 studies, respectively.

#### Sensitivity or Comparative Analysis

In the studies utilizing the AirQ software, nine (5%) have conducted a sensitivity or comparative risk analysis, while only eight (9%) studies have performed a sensitivity assessment using AirQ+ [85, 95–97]. The remaining studies have not reported sensitivity assessment (Supplementary Figure S16). In the two studies [9, 85], the authors compared the output from AirQ+ and BenMAP–CE (software versions not reported by the authors), considering different choices of the input parameters for air quality, demographic and mortality statistics (BI data), and CRFs (RR and counterfactual value). The comparative analysis showed that both models gave consistent health impact assessment results. In the study conducted by Al-Hemoud and others [95], the authors calculated the preventable premature deaths if the current ambient PM_2.5_ concentration in Kuwait would be reduced to the WHO Interim Target-1 (35 μg/m^3^) for the years 2025–2035 and 2045 and considered these estimates as a sensitivity analysis. However, they did not disclose the method used to determine BI, a crucial factor in assessing the health impact due to AAP for the years 2025, 2035, and 2045. Also, Y.A. Aliyu and J.O. Botai (2018) estimated and compared health effects of PM_2.5_ and PM_10_ using two different RRs; WHO AirQ+ default and based on multiple analysis of peer-reviewed findings conducted in Asia [66]. In the study of Ebrahimi and others [96], the health burden attributable to ambient NO_2_ concentrations in Tehran using AirQ+ was compared to predictions using the WANN (wavelet transformation and wavelet neural network) approach. They reported that “*analyzing the sensitivity of mortality resulted from NO*_*2*_ *concentration was done by using of wavelet neural network and AirQ+ software, and it was concluded that the increase or decrease in the parameters affecting NO*_*2*_ *concentration will affect the mortality rate*” [96].

## Discussion

Due to compelling evidence of AP health effects, there is an increasing interest in monitoring and modeling the health effects. From the analysis of published papers and reports using the AirQ and AirQ+ HRA tools, we learned useful lesson that can be beneficial in future applications. Our critical appraisal of published literature revealed serious reporting issues on all input data categories. The most common deficiencies included poor reporting of AP exposure data and its quality (data coverage and validity, monitoring station types), and/or poor reporting of epidemiological data with justifications for the choices that were made, e.g., population size, CRF, BI, AP scenarios, associations of interest, and lack of conducting uncertainty assessment.

### Examples For the Significance of Input Data in Estimating the Health Effects of AAP

To demonstrate the significance of some of input data in estimating the health effects of AAP, we utilized WHO AirQ+ (v.2.2) software [10]. We modified certain input parameters, including annual mean of ambient PM_2.5_ concentration and BI (in hypothetical cities in the United States and Iran) by 10%–20%, and calculated all-cause mortality (Supplementary Figures S17–20). We used two categories of annual mean of ambient PM_2.5_ concentrations; high (a hypothetical city in Iran) and low (a hypothetical city in the Unites States) based on the concentrations observed in Iran (30–45 μg m^−3^) and the Unites States (6–10 μg m^−3^) in 2019, respectively (Tables 1, 2).

**TABLE 1:** The number of all-cause mortality for adults (aged 25+ years) by changing the annual mean of ${\text{PM}}_{2.5}$ concentration in two categories of high (a hypothetical city in Iran, 2019) and low (a hypothetical city in the United States, 2019) levels of ${\text{PM}}_{2.5}$ .

Hypothetical location	Input parameters	Concentration (µg m^-3^)	Mortality nonaccidental deaths (NCDs + ALRI), adults age 25+		
			Central	Lower	Upper
**A hypothetical city in Iran**	**Baseline Incidence (BI):** 670 per 10^5^ population at risk; **Population of adults age 25+:** 6,800,000; **Calculation Method:** Global Exposure Mortality Model—GEMM (2018); **Cut-off value:** 2.4 μg m^−3^	38.6	11,403	8,877	13,755
		42.0	12,001	9,361	14,448
		35.0	10,743	8,345	12,986
**A hypothetical city in the United States**	**Baseline Incidence (BI):** 1,165 per 10^5^ population at risk; **Population of adults age 25+:** 2,100,000; **Calculation Method:** Global Exposure Mortality Model—GEMM (2018); **Cut-off value:** 2.4 μg m^−3^	9.0	2,390	1820	2,944
		11.0	2,768	2,113	3,404
		7.0	1940	1,474	2,396

**TABLE 2:** The number of all-cause mortality for adults (aged 25+ years) by changing the baseline incidence per 10^5^ population at risk (for hypothetical cities in Iran and the United States, 2019).

Hypothetical location	Input parameters	Baseline incidence per 10^5^ population at risk	Mortality nonaccidental deaths (NCDs + ALRI), adults age 25+		
			Central	Lower	Upper
**A hypothetical city in Iran**	**Annual mean of** $PM_{2.5}$ **:** 38.6 μg m^−3^; **Population of adults age 25+:** 6,800,000; **Calculation Method:** Global Exposure Mortality Model—GEMM (2018); **Cut-off value:** 2.4 μg m^-3^	670	11,403	8,877	13,755
		540	9,191	7,155	11,086
		800	13,616	10,600	16,424
**A hypothetical city in the United States**	**Annual mean of** $PM_{2.5}$ **:** 9.0 μg m^−3^; **Population of adults age 25+:** 2,100,000; **Calculation Method:** Global Exposure Mortality Model—GEMM (2018); **Cut-off value:** 2.4 μg m^−3^	1,165	2,390	1820	2,944
		1,325	2,718	2070	3,349
		1,005	2061	1,570	2,540

By changing the annual mean of ambient PM_2.5_ concentrations from 38.6 μg m^−3^–42 μg m^−3^ or 35 μg m^−3^ (Table 1), the number of attributed premature deaths changed from 11,403 (95% CI: 8,877; 13,755) to 12,001 (95% CI: 9,361; 14,448) and 10,743 (95% CI: 8,345; 12,986), respectively. For low category of annual mean of ambient PM_2.5_ concentrations, by changing it from 9 μg m^−3^–11 μg m^−3^ or 7 μg m^−3^, the number of attributable cases changed from 2,390 (95% CI: 1,820; 2,944) to 2,768 (95% CI: 2,113; 3,404) and 1,940 (95% CI: 1,474; 2,396), respectively (Table 1).

With respect to BI for all-cause mortality (Table 2), at the annual mean concentrations of ambient PM_2.5_ 38.6 μg m^−3^, by changing it from 670 to 540 or 800, the number attributable cases changed from 11,403 (95% CI: 8,877; 13,755) to 9,191 (95% CI: 7,155; 11,086) and 13,616 (95% CI: 10,600; 16,424), respectively. At the annual mean concentrations of ambient PM_2.5_ 9 μg m^−3^, by changing BI from 1,165 to 1,005 or 1,325, the number of attributable cases changed from 2,390 (95% CI: 1,820; 2,944) to 2,061 (95% CI: 1,570; 2,540) and 2,718 (95% CI: 2070, 2,070; 3,349), respectively (Table 2). As a result, inaccurate information on the ambient air quality data and BI can result in some degree of uncertainties affecting the AAP-related health effects.

### Proposals for Good Practice

We propose that a good practice for impact assessment of AP should include:

• Clear definitions of the scope, motivation, and objectives, e.g., HRA or BoD (burden of disease) assessment, including the population size (with age-groups of interest), air pollutants of interest, AP exposure data source, (with data quality and coverage across time, data cleaning plans and procedures, data validation) and clear, justification for choices made, especially for CRF/ERF, BI, the exposure scenarios, the health outcomes of interest, uncertainty assessment procedures, and the stakeholders involved/targeted.
• Use of reliable and representative sources of data, such as population data, mortality/morbidity rates, monitoring stations, or validated estimates from satellite data, or modelling outputs, to ensure their quality and validity.
• Selection of appropriate CRF/ERF (such as available ones in the AirQ+ software), based on the health outcomes and pollutants of interest. The CRF/ERF should be consistent with the available evidence and reflect the uncertainty and variability in the estimates, as well as be applicable over the range of exposures considered by the analysis.
• Estimation of the attributable number of cases of mortality/morbidity due to AP for each exposure scenario and health outcome as well as presenting PAF and attributable mortality/morbidity.
• Interpretation and communication of the results of the HRA, considering the limitations, assumptions, and uncertainties of the analysis. The results should be presented in a clear and transparent way, using tables, graphs, maps, or other visual aids and should also be discussed in relation to the policy context, the stakeholders’ interests, and the ethical implications of the HRA.
• Communication and dissemination of the findings of the HRA or BoD assessment to the scientific community, the relevant stakeholders and decision-makers, or media and the general public. If feasible, we recommend openly publishing the data in the analysis, as Supplementary Material across the articles or reports to ensure transparency and replicability.
• Use of clear and concise language and visual aids to convey the main messages and implications.
• Evaluation and monitoring the HRA process and outcomes, including the methods, data, results, and impacts.
• Identify and report the strengths and weaknesses of the HRA and provide feedback for future improvements.
• Also, proposing detailed guidelines and offering training courses on HRA of AP, using AirQ+ or other tools, has been shown to deliver the greatest benefit to new users of health risk assessment tools [9].

By following the proposed steps, the impact assessment of AP using AirQ+ or other software, can be a powerful tool to inform policymakers and stakeholders, and support evidence-based decisions to protect and promote public health.

### Strengths and Limitations

Our study involving databases PubMed, Web of Science Core Collection, and Scopus forms a systematic review with no language restrictions. We are assured that we have discovered all English and non-English studies published and indexed in these databases from inception until 31 December 2022. An additional 801 records were identified through our Google Scholar search, again without any language restrictions. The WHO database also contained numerous non-English studies. Anyhow, albeit we have gathered and incorporated most of the studies related to AirQ and AirQ+, there is a possibility that we might have overlooked some records (especially non-English) that were not among the 801 records retrieved on Google Scholar.
