# Supplementary material for: Two Decades of Air Pollution Health Risk Assessment: Insights From the Use of WHO’s AirQ and AirQ+ Tools
Source: Public Health Rev. 2024 Jun 18;45:1606969. doi: 10.3389/phrs.2024.1606969 (PMC11217191; doi:10.3389/phrs.2024.1606969)
Supplement: Supplementary file 1 [file DataSheet1.PDF]

# Supplementary Information to:

## Two Decades of Air Pollution Health Risk Assessment: Insights from the use of WHO's AirQ and AirQ+ Tools

*Heresh Amini[1,2,†,*], Fatemeh Yousefian[3,†] Sasan Faridi[4,†], Zorana J. Andersen[5],Ellénore Calas[6], Alberto Castro[7,8], Karla Cervantes-Martínez[9], Thomas Cole-Hunter[5], Magali Corso[10], Natasa Dragic[11], Dimitris Evangelopoulos[12], Christian Gapp[13], Mohammad Sadegh Hassanvand[4], Ingu Kim[14], Alain Le Tertre[15], Sylvia Medina[10], Brian Miller[16], Stephanie Montero[17], Weeberb J. Requia[18], Horacio Riojas-Rodriguez[19], David Rojas-Rueda[20,21], Evangelia Samoli[22], Jose Luis Texcalac-Sangrador[19], Maayan Yitshak-sade[1,2], Joel Schwartz[23], Nino Kuenzli[7,8], Joseph V. Spadaro[24], Michal Krzyzanowski[25], Pierpaolo Mudu[14]*

[1]*Department of Environmental Medicine and Climate Science, Icahn School of Medicine at Mount Sinai, New York, USA*
[2]*Institute for Climate Change, Environmental Health, and Exposomics, Icahn School of Medicine at Mount Sinai, New York, USA*
[3]*Department of Environmental Health Engineering, Faculty of Health, Kashan University of Medical Sciences, Kashan, Iran*
[4]*Center for Air Pollution Research (CAPR), Institute for Environmental Research (IER), Tehran University of Medical Sciences, Tehran, Iran*
[5]*Department of Public Health, University of Copenhagen, Denmark*
[6]*University of Paris-Saclay, France*
[7]*Swiss Tropical and Public Health Institute, Allschwil, Switzerland*
[8]*University of Basel, Basel, Switzerland*
[9]*Department of Environment, Climate Change and Health, World Health Organization, Geneva, Switzerland*
[10]*Department of Environmental and Occupational Health, Santé Publique France*
[11]*Faculty of Medicine, University of Novi Sad, Novi Sad, Republic of Serbia*
[12]*Environmental Research Group, MRC Centre for Environment and Health, Imperial College London, UK*
[13]*World Health Organization Regional Office for Europe, DK-2100 Copenhagen, Denmark*
[14]*European Centre for Environment and Health, World Health Organization, Regional Office for Europe, Bonn, Germany*
[15]*Regional Office Bretagne, Santé Publique France, Rennes, France*
[16]*Institute of Occupational Medicine (IOM), Edinburgh, Scotland, UK*
[17]*Clean Air Institute, Washington DC, United States*
[18]*Center for Environment and Public Health Studies, School of Public Policy and Government, Fundação Getúlio Vargas, Brasília, Brazil*
[19]*Department of Environmental Health, National Institute of Public Health, Cuernavaca, Mexico*
[20]*Department of Environmental and Radiological Health Sciences, Colorado State University, Fort Collins, USA*
[21]*Colorado School of Public Health, Colorado State University, Fort Collins, USA*
[22]*Department of Hygiene, Epidemiology and Medical Statistics, Medical School, National and Kapodistrian University of Athens, Athens, Greece*
[23]*Department of Environmental Health, Harvard T.H. Chan School of Public Health, Boston, MA, USA*
[24]*Spadaro Environmental Research Consultants (SERC), Philadelphia, PA, USA*
[25]*School of Public Health, Imperial College London, London, UK*

[†]Co-first authors

*Corresponding author. Email: heresh.amini@mssm.edu

**Table S1:** Full search strategy for PubMed, Scopus, and Web of Science (# of search results).

| **PubMed: #145** |
|---|
| (((((((((((((((((("Air pollution"[Title/Abstract]) OR ("air pollutant*"[Title/Abstract])) OR ("air quality"[Title/Abstract])) OR ("particulate matter"[Title/Abstract])) OR (PM10[Title/Abstract])) OR (PM2.5[Title/Abstract])) OR ("fine particulate matter"[Title/Abstract])) OR (NOx[Title/Abstract])) OR (NO2[Title/Abstract])) OR ("nitrogen dioxide"[Title/Abstract])) OR ("nitrogen Oxide"[Title/Abstract])) OR (SO2[Title/Abstract])) OR ("sulfur dioxide"[Title/Abstract])) OR (ozone[Title/Abstract])) OR ("Carbon monoxide"[Title/Abstract]))) OR (CO[Title/Abstract])) AND ((((((((((("Air Q"[Title/Abstract]) OR ("AirQ"[Title/Abstract])) OR ("AirQ+"[Title/Abstract])) OR ("AirQ plus"[Title/Abstract])) OR ("Air Q model"[Title/Abstract])) OR ("WHO software"[Title/Abstract])) OR ("Air Q software"[Title/Abstract])) OR ("Air Quality model"[Title/Abstract])) OR (AQM[Title/Abstract])) OR ("WHO method"[Title/Abstract])) OR ("WHO approach"[Title/Abstract]))) AND ((((((((((((((((((((((((((((((((((("Health impact assessment"[Title/Abstract]) OR (HIA[Title/Abstract])) OR ("exposure-response function"[Title/Abstract])) OR (Mortality[Title/Abstract])) OR (Morbidity[Title/Abstract])) OR ("Long-term effect"[Title/Abstract])) OR ("Short-term effect"[Title/Abstract])) OR ("Life expectancy"[Title/Abstract])) OR ("Risk assessment"[Title/Abstract])) OR ("Relative Risk"[Title/Abstract])) OR ("Chronic obstructive pulmonary disease"[Title/Abstract])) OR (COPD[Title/Abstract])) OR ("Cardiovascular mortality"[Title/Abstract])) OR ("Respiratory mortality"[Title/Abstract])) OR ("Cardiovascular death"[Title/Abstract])) OR ("Respiratory death"[Title/Abstract])) OR ("Hospital admission*"[Title/Abstract])) OR ("Baseline Incidence"[Title/Abstract])) OR (Stroke[Title/Abstract])) OR (Cancer[Title/Abstract])) OR (Death[Title/Abstract])) OR ("Health effect"[Title/Abstract])) OR ("Human health risk"[Title/Abstract])) OR ("All cause mortality"[Title/Abstract])) OR ("All-cause mortality"[Title/Abstract])) OR ("Lung cancer"[Title/Abstract])) OR (LC[Title/Abstract])) OR (IHD[Title/Abstract])) OR ("Ischemic Heart Diseases"[Title/Abstract])) OR (LRI[Title/Abstract])) OR ("Lower respiratory infection"[Title/Abstract])) OR ("Acute LRI"[Title/Abstract])) OR (ALRI[Title/Abstract])) OR (YLL[Title/Abstract])) OR ("Years of life lost"[Title/Abstract])) |
| **Scopus: # 474** |
| ( ( TITLE-ABS-KEY ( "Air Q" ) OR TITLE-ABS-KEY ( "AIRQ+" ) OR TITLE-ABS-KEY ( "AIRQ plus" ) OR TITLE-ABS-KEY ( "WHO software" ) OR TITLE-ABS-KEY ( "AirQ" ) OR TITLE-ABS-KEY ( "WHO method" ) OR TITLE-ABS-KEY ( "Air Q Model" ) OR TITLE-ABS-KEY ( "AirQ software" ) OR TITLE-ABS-KEY ( "WHO approach" ) OR TITLE-ABS-KEY ( "Air quality model" ) OR TITLE-ABS-KEY ( "AQM" ) ) ) AND ( ( TITLE-ABS-KEY ( "Air Q" ) OR TITLE-ABS-KEY ( "WHO software" ) OR TITLE-ABS-KEY ( "Health impact assessment" ) OR TITLE-ABS-KEY ( "HIA" ) OR TITLE-ABS-KEY ( "exposure-response function" ) OR TITLE-ABS-KEY ( "Mortality" ) OR TITLE-ABS-KEY ( "Morbidity" ) OR TITLE-ABS-KEY ( "Long-term effect" ) OR TITLE-ABS-KEY ( "Short-term effect" ) OR TITLE-ABS-KEY ( "Life expectancy" ) OR TITLE-ABS-KEY ( "Risk assessment" ) OR TITLE-ABS-KEY ( "Relative Risk" ) OR TITLE-ABS-KEY ( "Chronic obstructive pulmonary disease" ) OR TITLE-ABS-KEY ( "COPD" ) OR TITLE-ABS-KEY ( "Cardiovascular mortality" ) OR TITLE-ABS-KEY ( "Respiratory mortality" ) OR TITLE-ABS-KEY ( "Cardiovascular death" ) OR TITLE-ABS-KEY ( "Hospital admissions" ) OR TITLE-ABS-KEY ( "Baseline Incidence" ) OR TITLE-ABS-KEY ( "Health effect" ) OR TITLE-ABS-KEY ( "Human health risk" ) OR TITLE-ABS-KEY ( "All cause mortality" ) OR TITLE-ABS-KEY ( "All-cause mortality" ) OR TITLE-ABS-KEY ( "Lung cancer" ) OR TITLE-ABS-KEY ( "LC" ) OR TITLE-ABS-KEY ( "IHD" ) OR TITLE-ABS-KEY ( "Ischemic Heart Diseases" ) OR TITLE-ABS-KEY ( "Stroke" ) OR TITLE-ABS-KEY ( "LRI" ) OR TITLE-ABS-KEY ( "Lower respiratory infection" ) OR TITLE-ABS-KEY ( "Acute LRI" ) OR TITLE-ABS-KEY ( "ALRI" ) OR TITLE-ABS-KEY ( "YLL" ) OR TITLE-ABS-KEY ( "Years of life lost" ) OR TITLE-ABS-KEY ( "cancer" ) OR TITLE-ABS-KEY ( "death" ) ) ) AND ( ( TITLE-ABS-KEY ( "Air pollution" ) OR TITLE-ABS-KEY ( "Air quality" ) OR TITLE-ABS-KEY ( "Air pollutant*" ) OR TITLE-ABS- |

| |
|---|
| KEY ( "Fine Particulate matter" ) OR TITLE-ABS-KEY ( "Particulate matter" ) OR TITLE-ABS-KEY ( "Ozone" ) OR TITLE-ABS-KEY ( "PM2.5" ) OR TITLE-ABS-KEY ( "PM10" ) OR TITLE-ABS-KEY ( "O3" ) OR TITLE-ABS-KEY ( "NO2" ) OR TITLE-ABS-KEY ( "NOX" ) OR TITLE-ABS-KEY ( "SO2" ) OR TITLE-ABS-KEY ( "CO" ) OR TITLE-ABS-KEY ( "Nitrogen Dioxide" ) OR TITLE-ABS-KEY ( "Nitrogen Oxide" ) OR TITLE-ABS-KEY ( "Sulfur dioxide" ) OR TITLE-ABS-KEY ( "Carbon monoxide" ) ) ) AND ( EXCLUDE ( PUBYEAR , 2023 ) ) |
| **WOS:# 325** |
| ((((((((((((((((((((((((((((((((((TS=("Health impact assessment")) OR TS=(HIA)) OR TS=("exposure-response function")) OR TS=(Mortality)) OR TS=(Morbidity)) OR TS=("Long-term effect")) OR TS=("Short-term effect")) OR TS=("Life expectancy")) OR TS=("Risk assessment")) OR TS=("Relative Risk")) OR TS=("Chronic obstructive pulmonary disease")) OR TS=(COPD)) OR TS=("Cardiovascular mortality")) OR TS=("Respiratory mortality")) OR TS=("Cardiovascular death")) OR TS=("Respiratory death")) OR TS=("Hospital admission*")) OR TS=("Baseline Incidence")) OR TS=(Stroke)) OR TS=(Cancer)) OR TS=(Death)) OR TS=("Health effect")) OR TS=("Human health risk")) OR TS=("All cause mortality")) OR TS=("All-cause mortality")) OR TS=("Lung cancer")) OR TS=(LC)) OR TS=(IHD)) OR TS=("Ischemic Heart Diseases")) OR TS=(LRI)) OR TS=("Lower respiratory infection")) OR TS=("Acute LRI")) OR TS=(ALRI)) OR TS=(YLL)) OR TS=("Years of life lost") AND ((((((((((TS=("Air Q")) OR TS=("AirQ")) OR TS=("AirQ+")) OR TS=("AirQ plus")) OR TS=("Air Q model")) OR TS=("WHO software")) OR TS=("Air Q software")) OR TS=("Air Quality model")) OR TS=(AQM)) OR TS=("WHO method")) OR TS=("WHO approach") AND ((((((((((((((TS=("Air pollution")) OR TS=("air pollutant*")) OR TS=("air quality")) OR TS=("particulate matter")) OR TS=(PM10)) OR TS=(PM2.5)) OR TS=("fine particulate matter")) OR TS=(NOx)) OR TS=(NO2)) OR TS=("nitrogen dioxide")) OR TS=("nitrogen Oxide")) OR TS=(SO2)) OR TS=("sulfur dioxide")) OR TS=(O3)) OR TS=(ozone)) OR TS=("Carbon monoxide") |

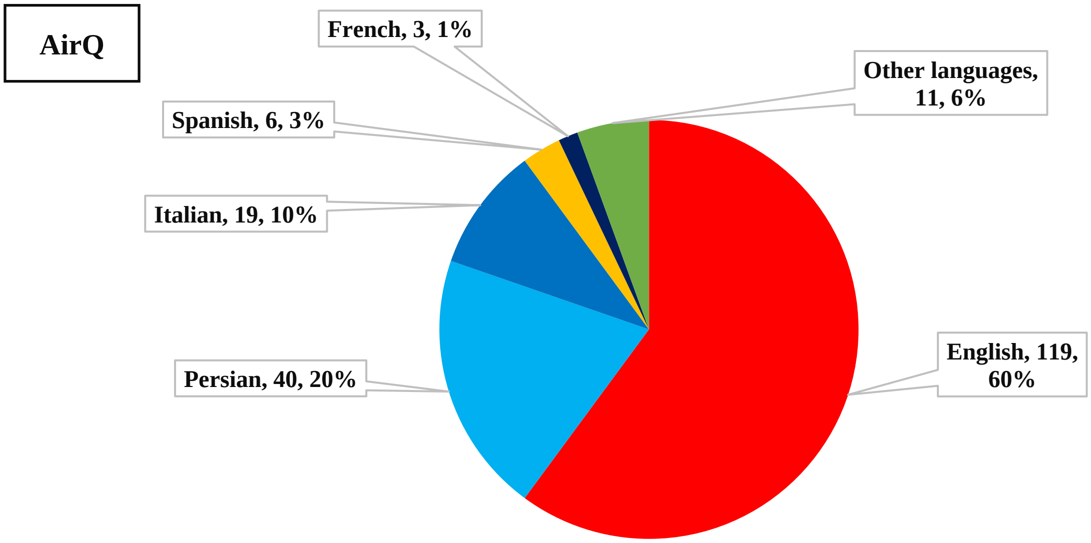


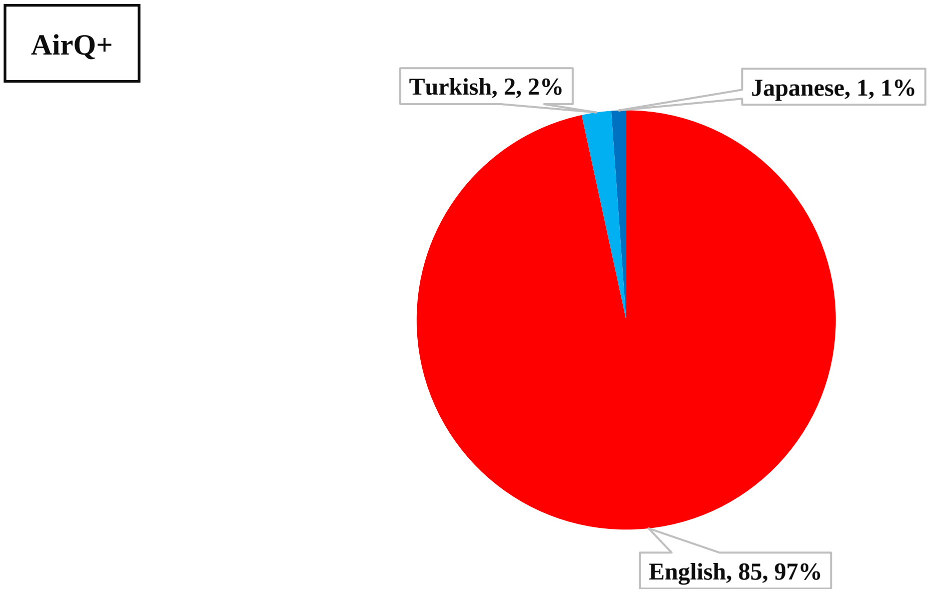


**Figure S1:** The language of published studies using AirQ and AirQ+ software (Other languages: Czech, Estonian, German, Polish, Hungarian, Croatian, and Portuguese).

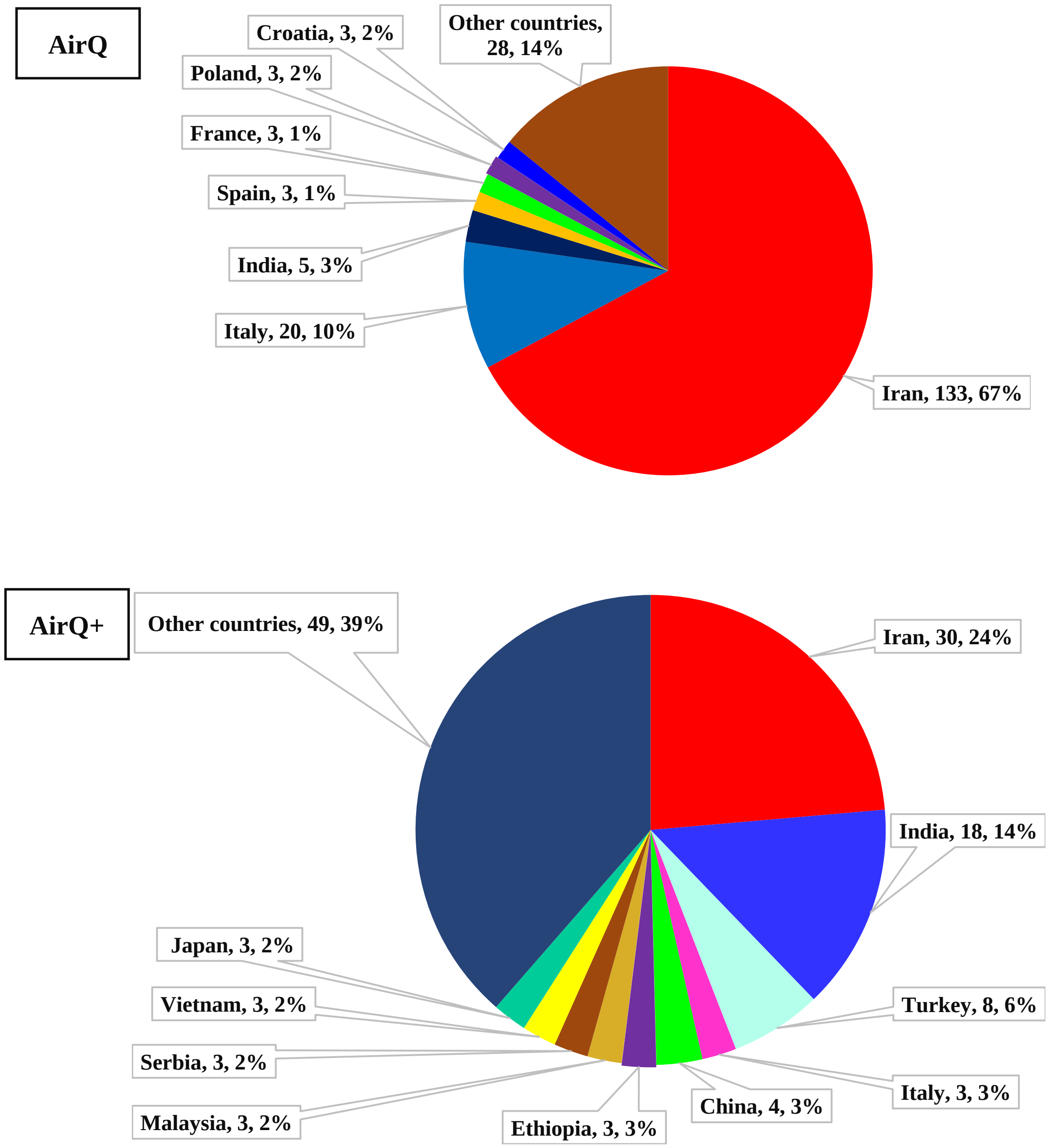


**Figure S2:** The number (percentage) of studies using AirQ and AirQ+ software by country.

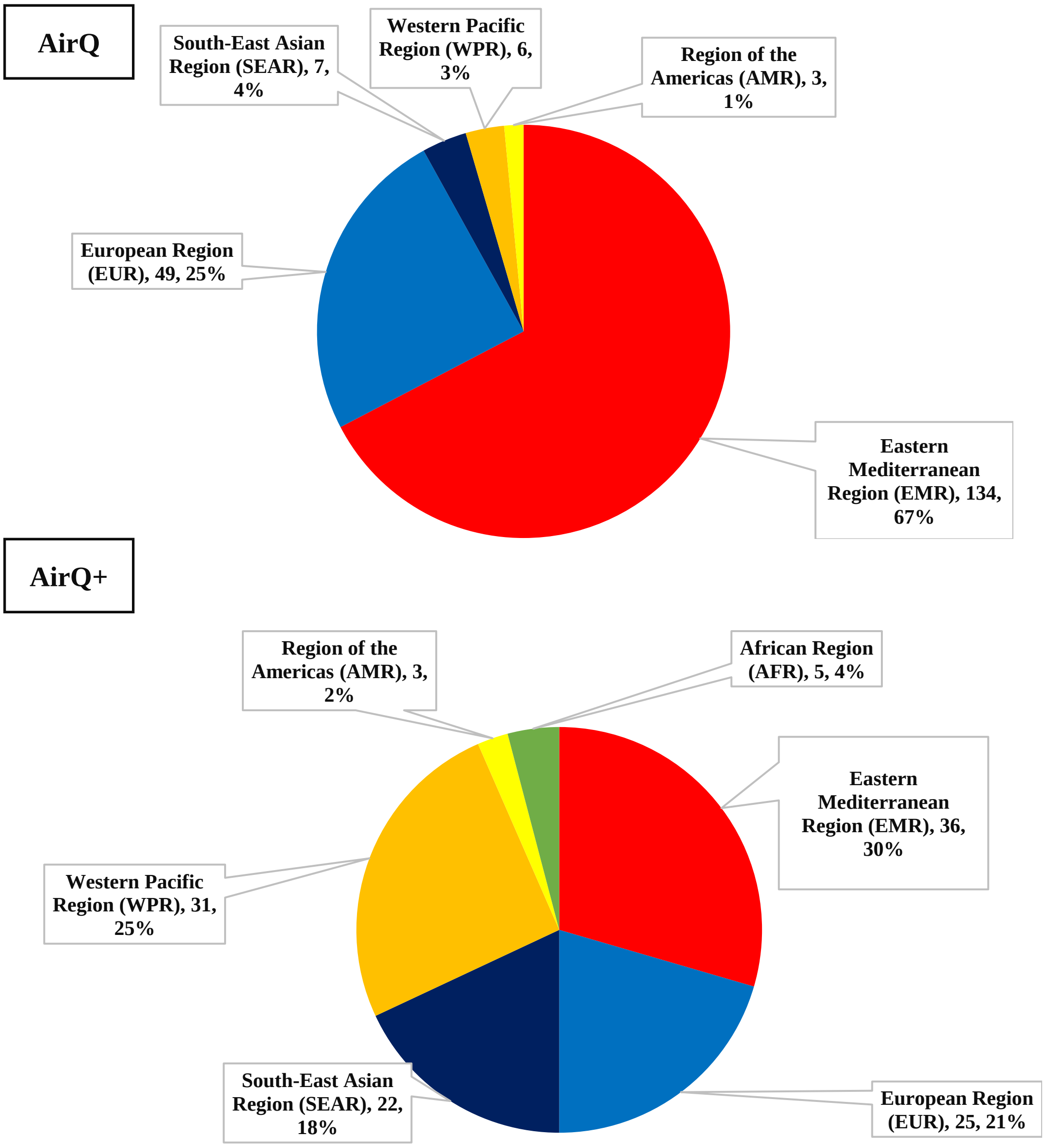


**Figure S3:** The number and percentage of studies using AirQ (top) and AirQ+ (bottom) software by the WHO region

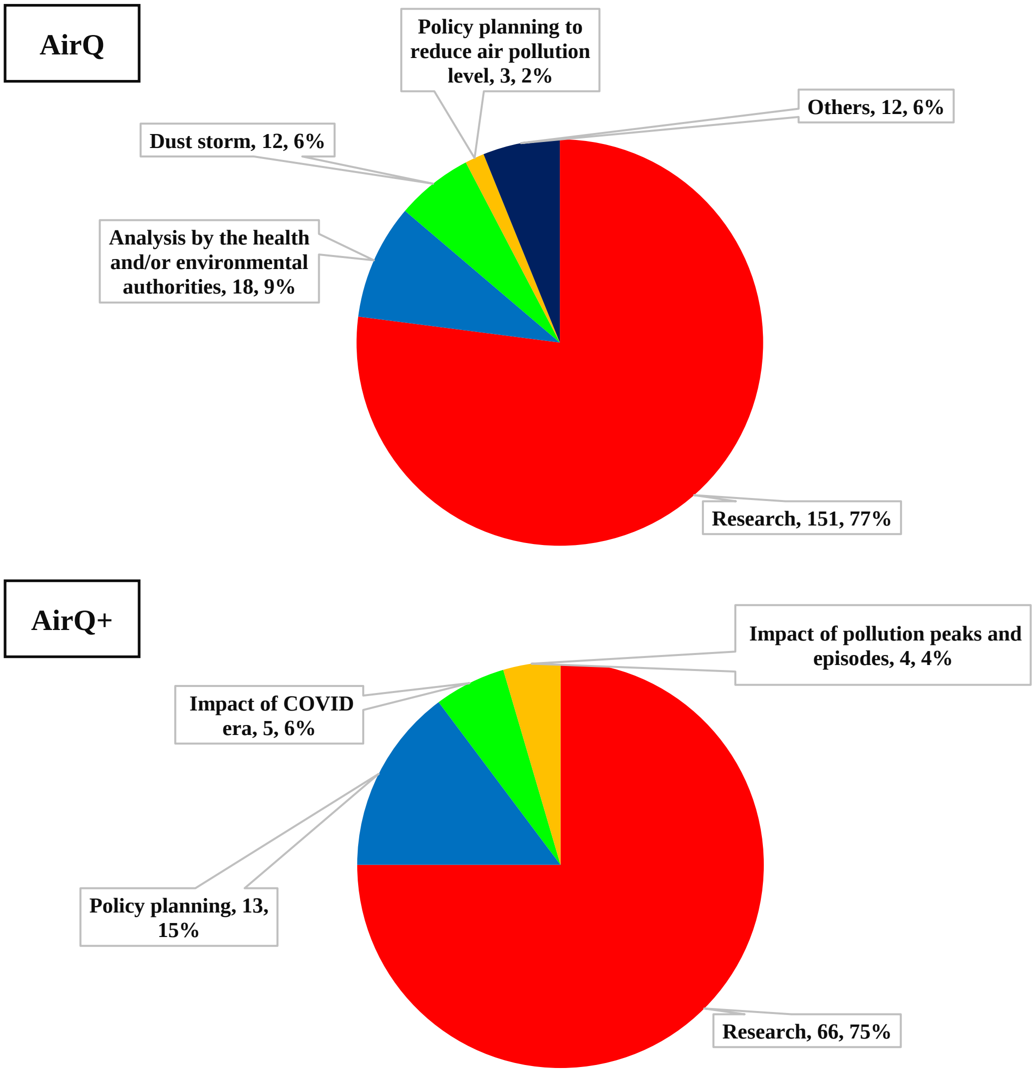


**Figure S4:** The number and percentage of studies using AirQ (top) and AirQ+ (bottom) software by motivation or the context of the study

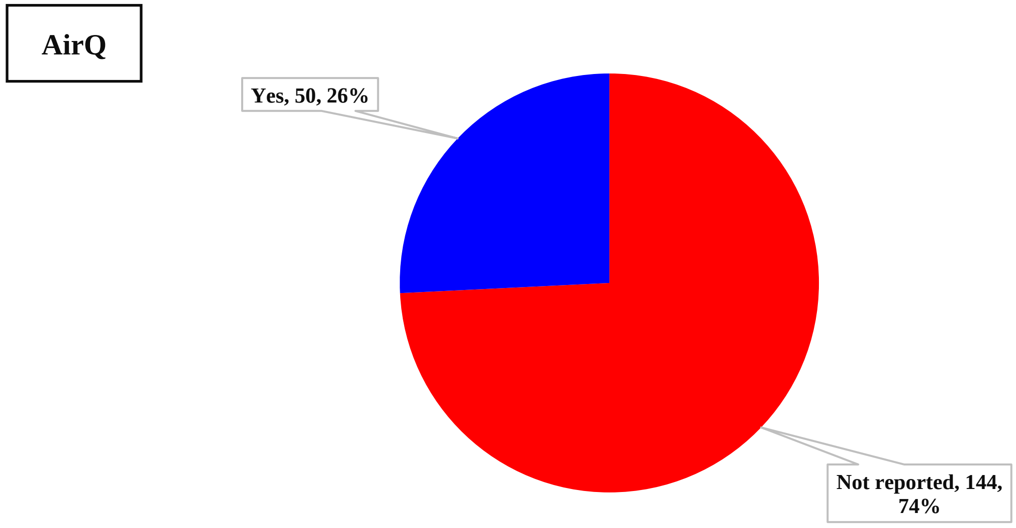


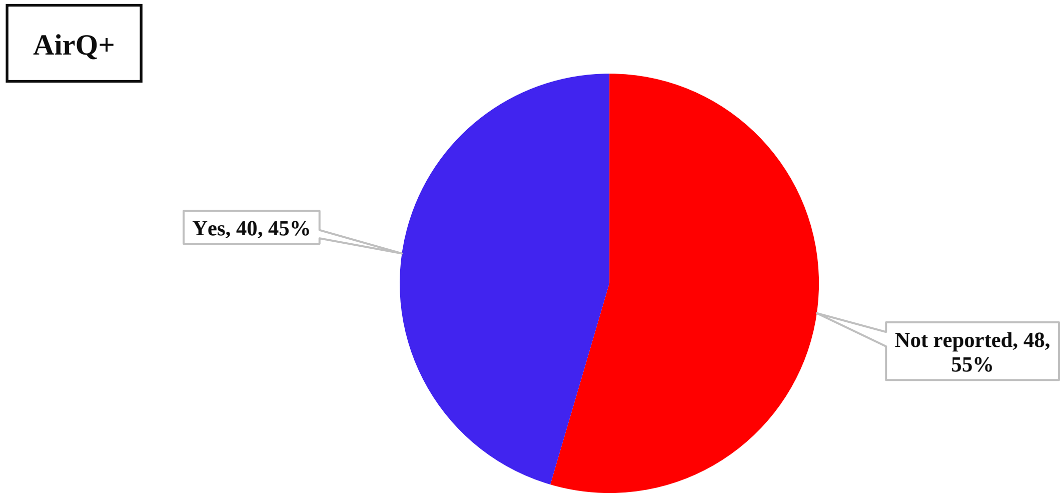


**Figure S5:** The number and percentage of studies using AirQ (top) and AirQ+ (bottom) software reporting (or not reporting) data coverage.

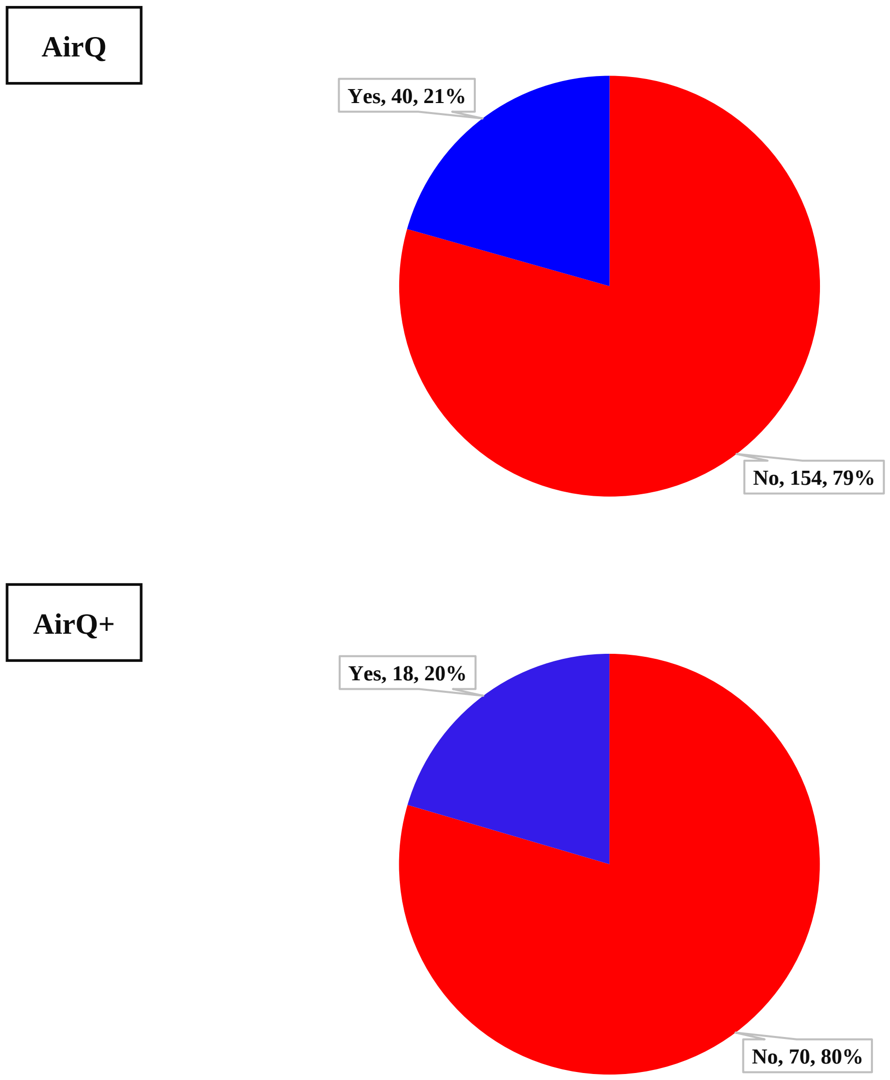


**Figure S6:** The number and percentage of studies using AirQ (top) and AirQ+ (bottom) software reporting (or not reporting) AP data processing and validation.

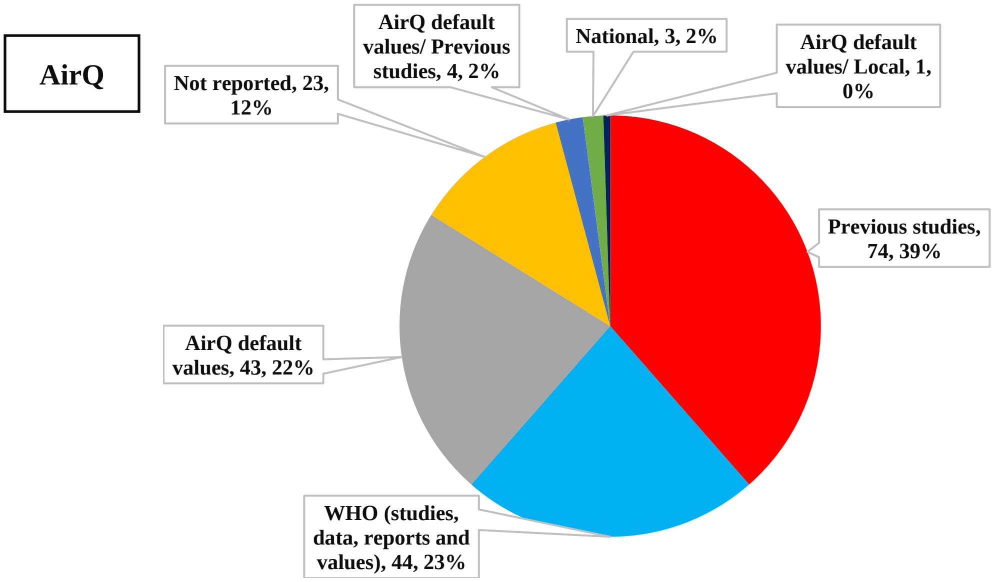


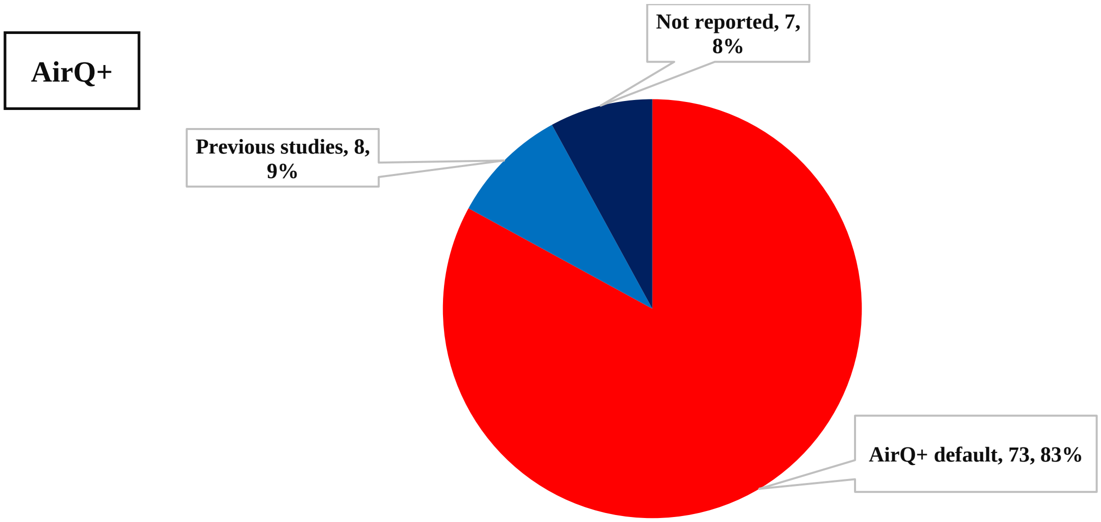


**Figure S7:** The number and percentage of studies using AirQ (top) and AirQ+ (bottom) software by the source of RR estimate.

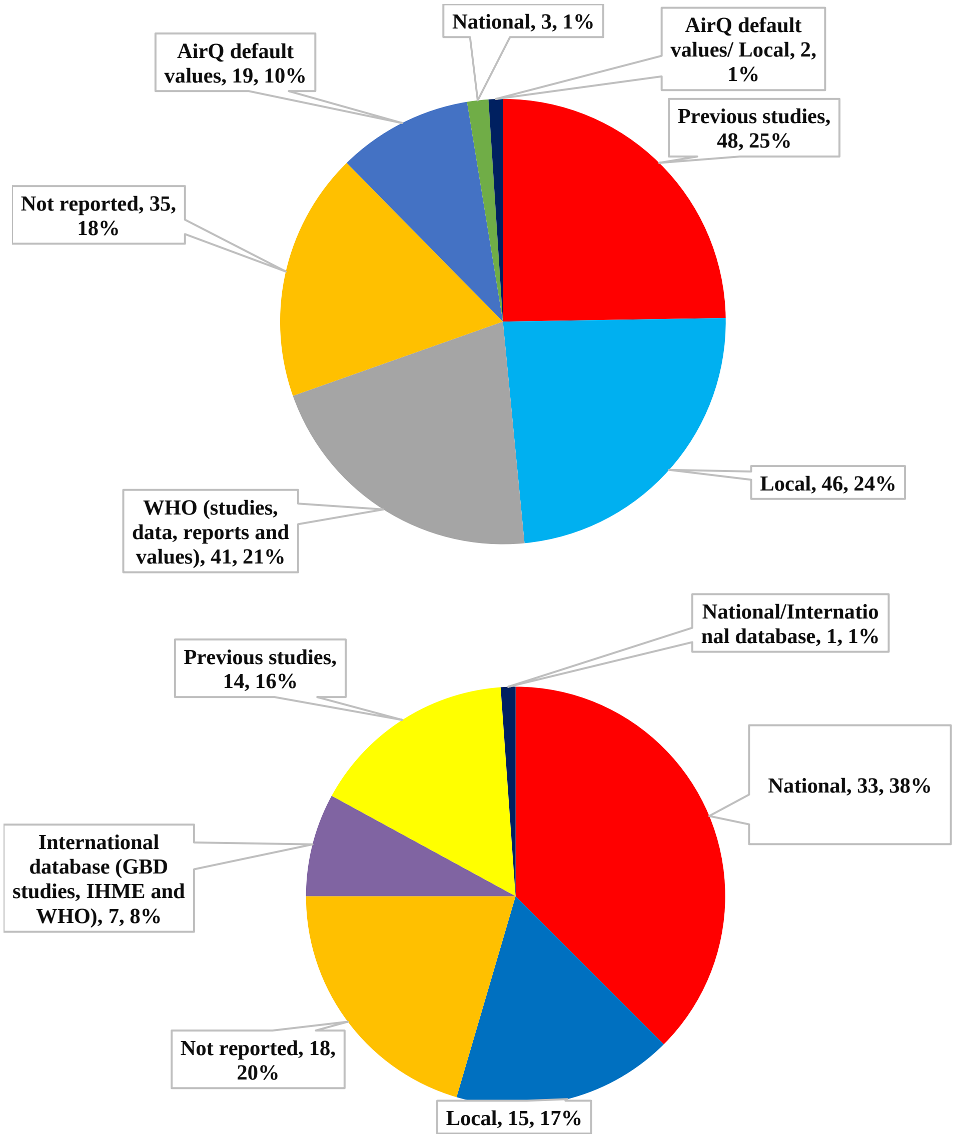


**Figure S8:** The number and percentage of studies using AirQ (top) and AirQ+ (bottom) software by the source of the data on baseline incidence.

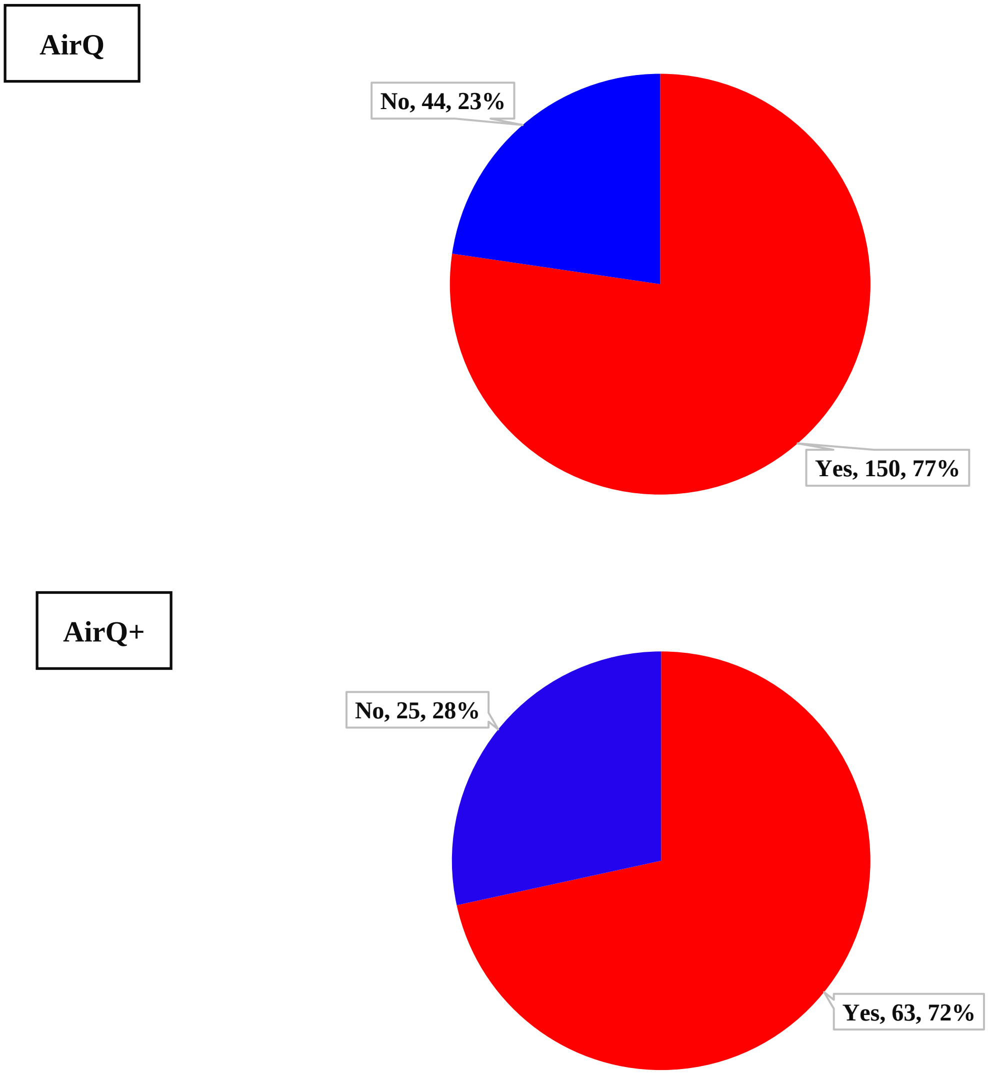


**Figure S9:** The number and percentage of studies using AirQ (top) and AirQ+ (bottom) software reporting (or not) the cut-off value in the assessment.

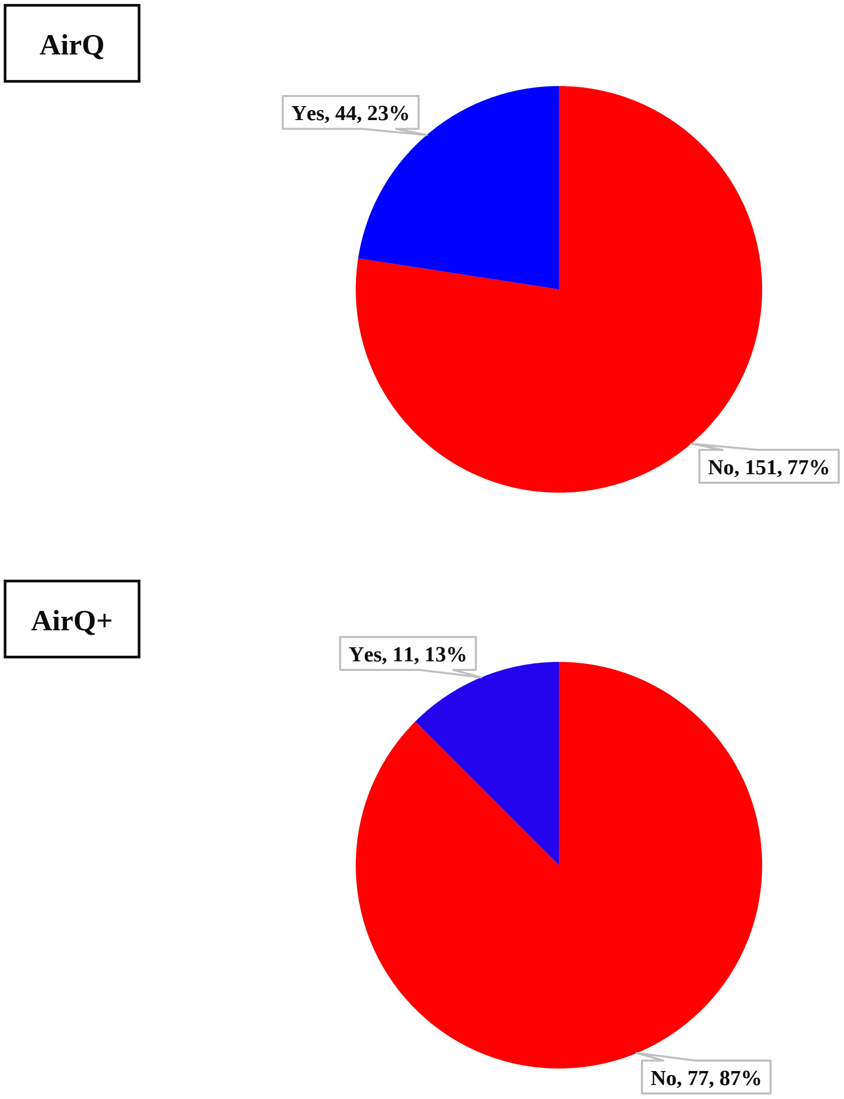


**Figure S10:** The number (percentage) of studies using AirQ and AirQ+ software reported population

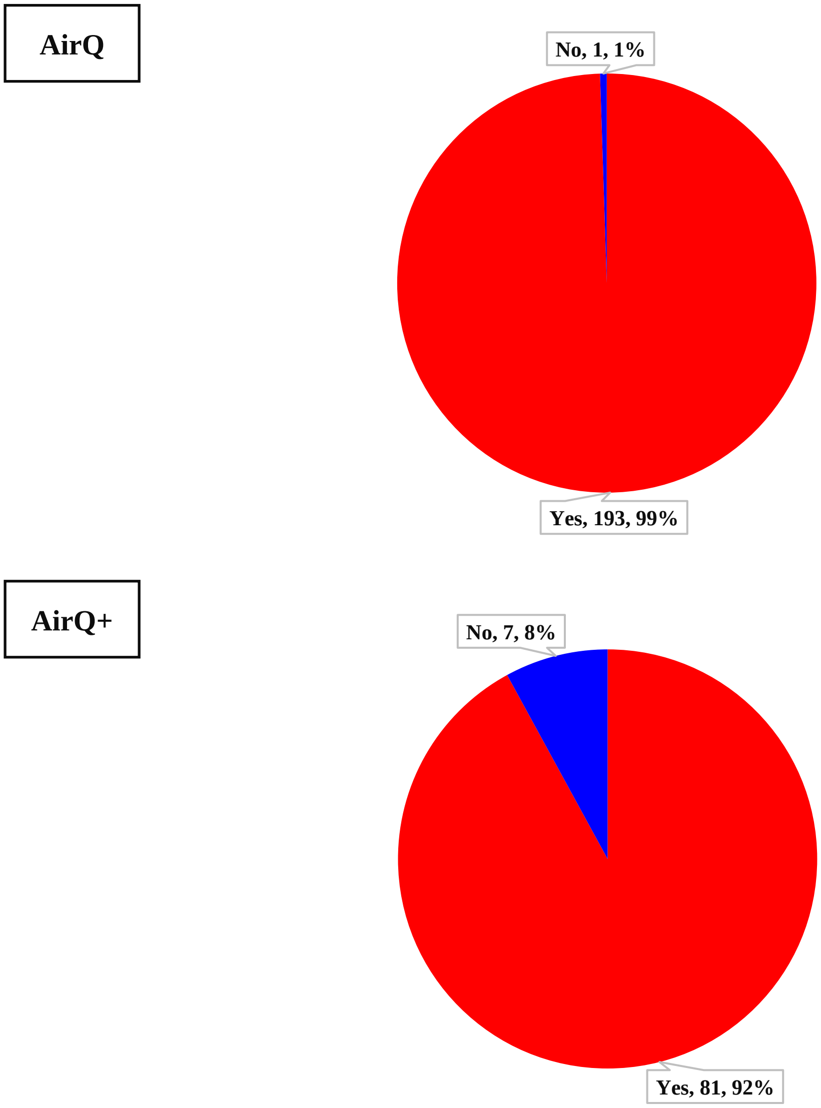


**Figure S11:** The number and percentage of studies using AirQ (top) and AirQ+ (bottom) software reporting number of cases attributed to the exposure.

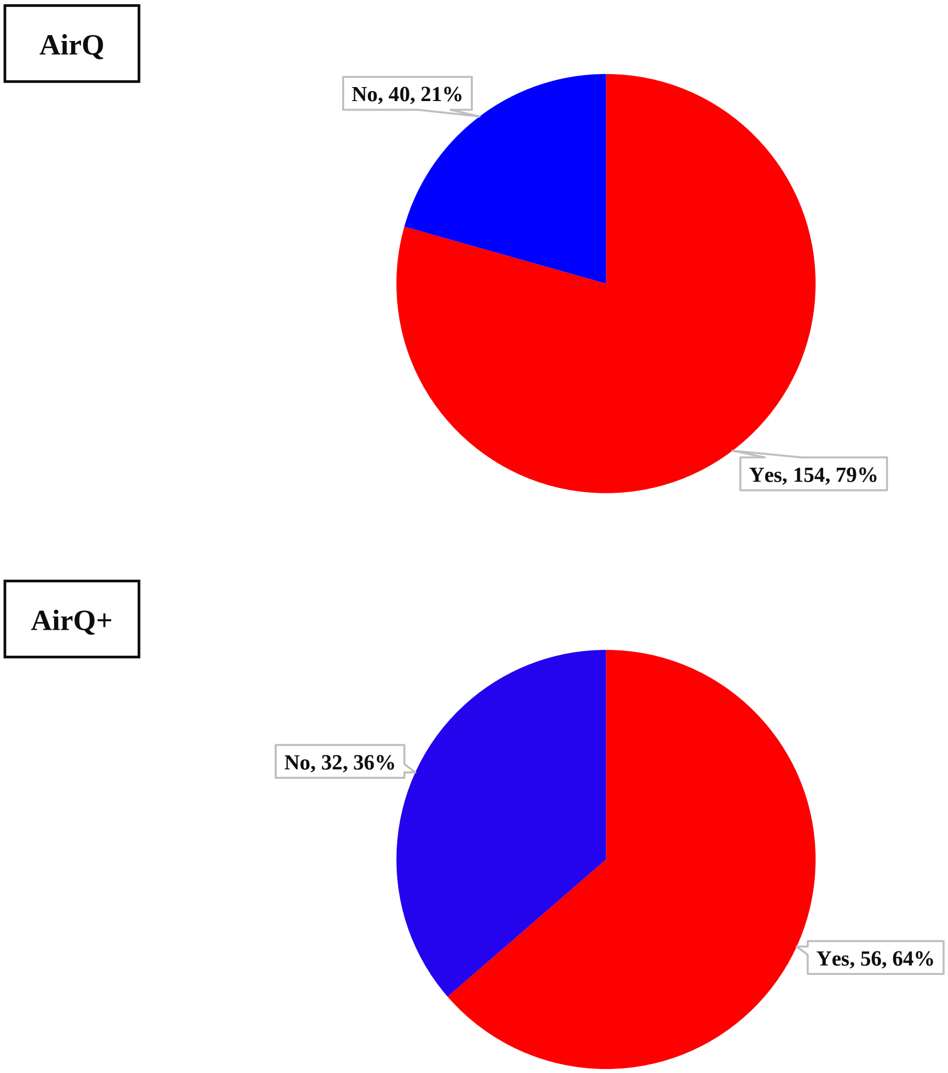


**Figure S12:** The number and percentage of studies using AirQ (top) and AirQ+ (bottom) software reporting population attributable fraction (PAF).

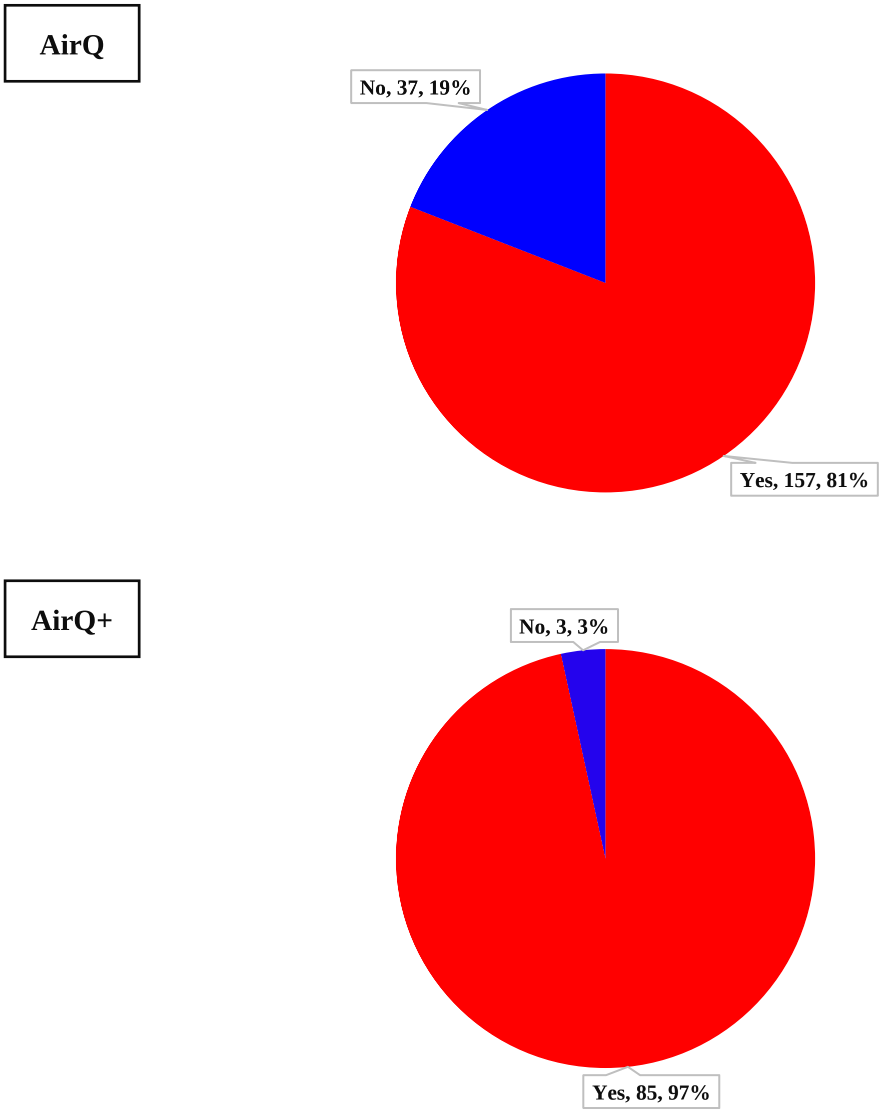


**Figure S13:** The number and percentage of studies using AirQ (top) and AirQ+ (bottom) software reporting confidence interval of the outcome parameters.

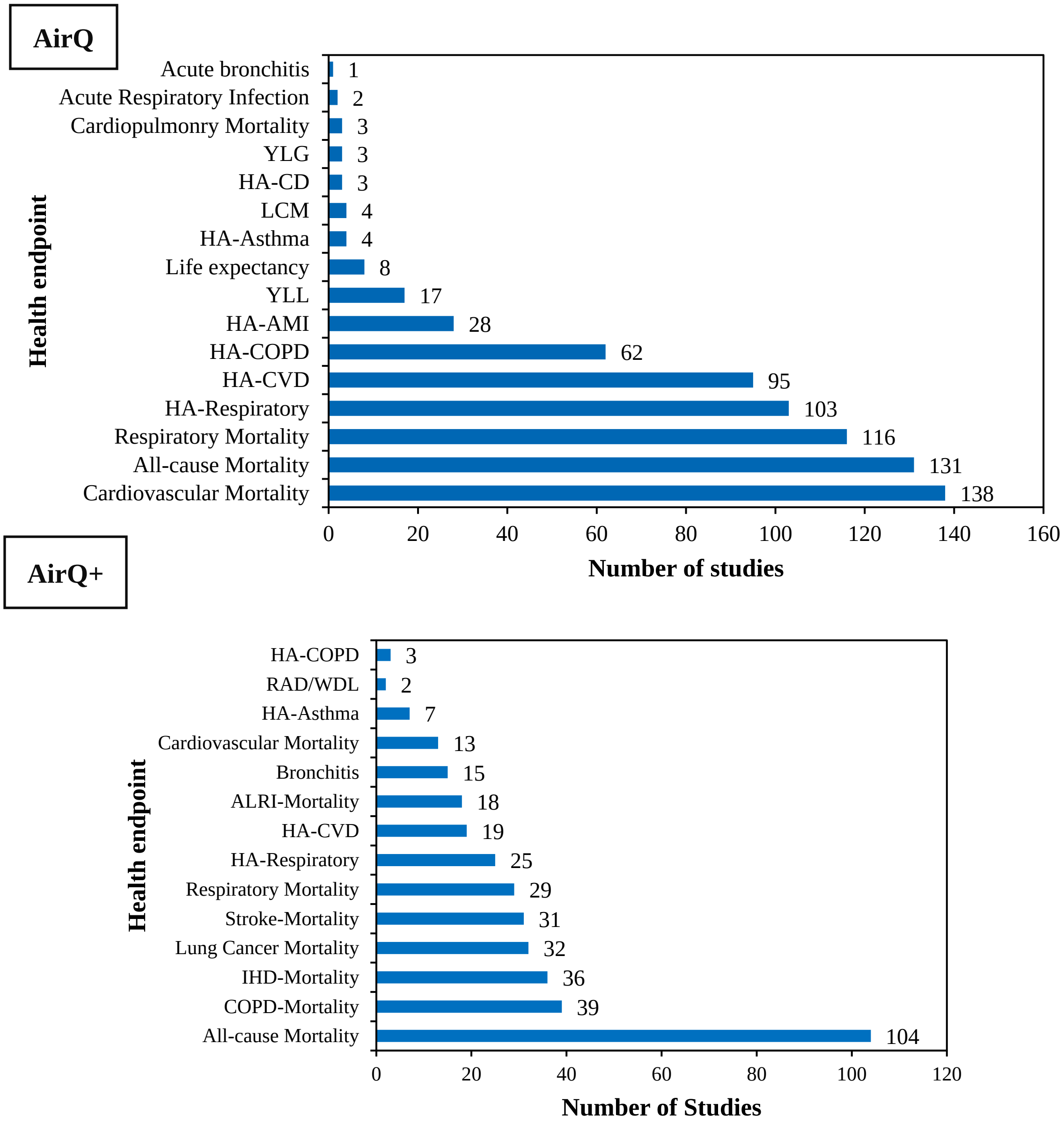


**Figure S14:** Health endpoints assessed in the included studies using AirQ (top) or AirQ+ (bottom) software. Abbreviations: AMI (acute myocardial infarction), ARI (acute respiratory infection), Bronchitis* (Mortality, Incidence of chronic bronchitis, prevalence of bronchitis), CD (cerebrovascular disease), COPD (chronic obstructive pulmonary disease), CVD (cardiovascular disease), HA (hospital admission), IHD (ischemic heart diseases), LCM (lung cancer mortality), RAD/WDL (Restricted activity days/ work days lost), YLG (years of life gained), and YLL (years of life lost).

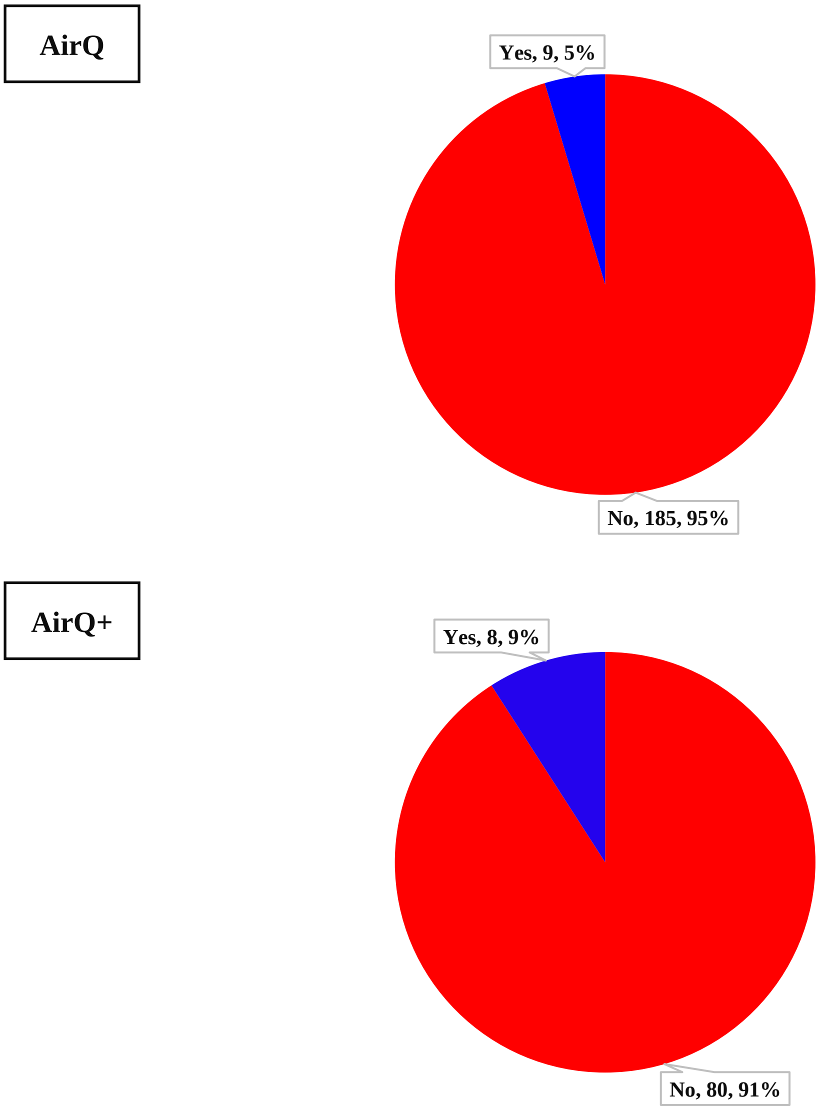


**Figure S15:** The number and percentage of studies using AirQ (top) and AirQ+ (bottom) software including a sensitivity or comparative analysis.

AirQ+

English

Glossary | Manuals | Disclaimer | Citation

Burden Evaluation | Detailed Results

**Burden Evaluation (PM2.5)**

Evaluation Name: NCDs+ALRI

**Health Endpoint**

**Health Endpoint:** Mortality nonaccidental deaths (NCDs+ALRI), adults age 25+

**Incidence (per 100 000 Population at risk per year):** 670

Population at risk: 6800000

**Calculation Parameters**

**Calculation Method:** Global Exposure Mortality Model - GEMM (2018)

Location mean concentration: 38.6

Cut-off value: 2.4

Calculate

**Results (last calculation 2024-02-29 09:32:16)**

| | Central | Lower | Upper |
|---|---|---|---|
| Estimated Attributable Proportion | 25.03% | 19.48% | 30.19% |
| Estimated number of Attributable Cases | 11,403 | 8,877 | 13,755 |
| Estimated number of Attributable Cases per 100,000 Population at Risk | 167.70 | 130.55 | 202.29 |

AirQ+

English

Glossary | Manuals | Disclaimer | Citation

Burden Evaluation | Detailed Results

**Burden Evaluation (PM2.5)**

Evaluation Name: NCDs+ALRI

**Health Endpoint**

**Health Endpoint:** Mortality nonaccidental deaths (NCDs+ALRI), adults age 25+

**Incidence (per 100 000 Population at risk per year):** 670

Population at risk: 6800000

**Calculation Parameters**

**Calculation Method:** Global Exposure Mortality Model - GEMM (2018)

Location mean concentration: 42

Cut-off value: 2.4

Calculate

**Results (last calculation 2024-02-29 09:30:49)**

| | Central | Lower | Upper |
|---|---|---|---|
| Estimated Attributable Proportion | 26.34% | 20.55% | 31.71% |
| Estimated number of Attributable Cases | 12,001 | 9,361 | 14,448 |
| Estimated number of Attributable Cases per 100,000 Population at Risk | 176.48 | 137.66 | 212.47 |

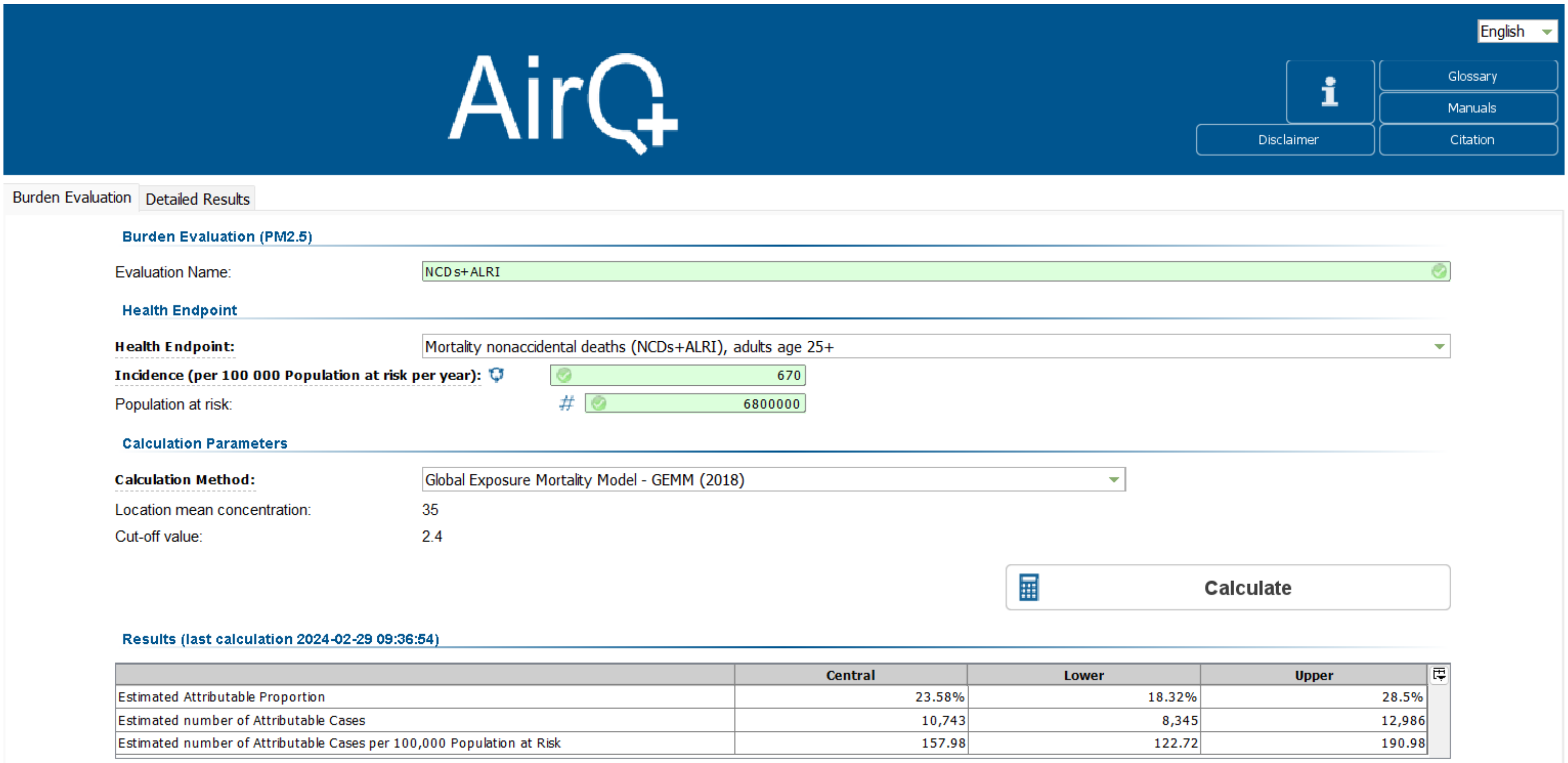


**Figure S16.** The number of all-cause mortality for adults (aged 25+ years) by changing the annual mean of $PM_{2.5}$ concentration from 38.6 to 42 and 35 µg $m^{-3}$.

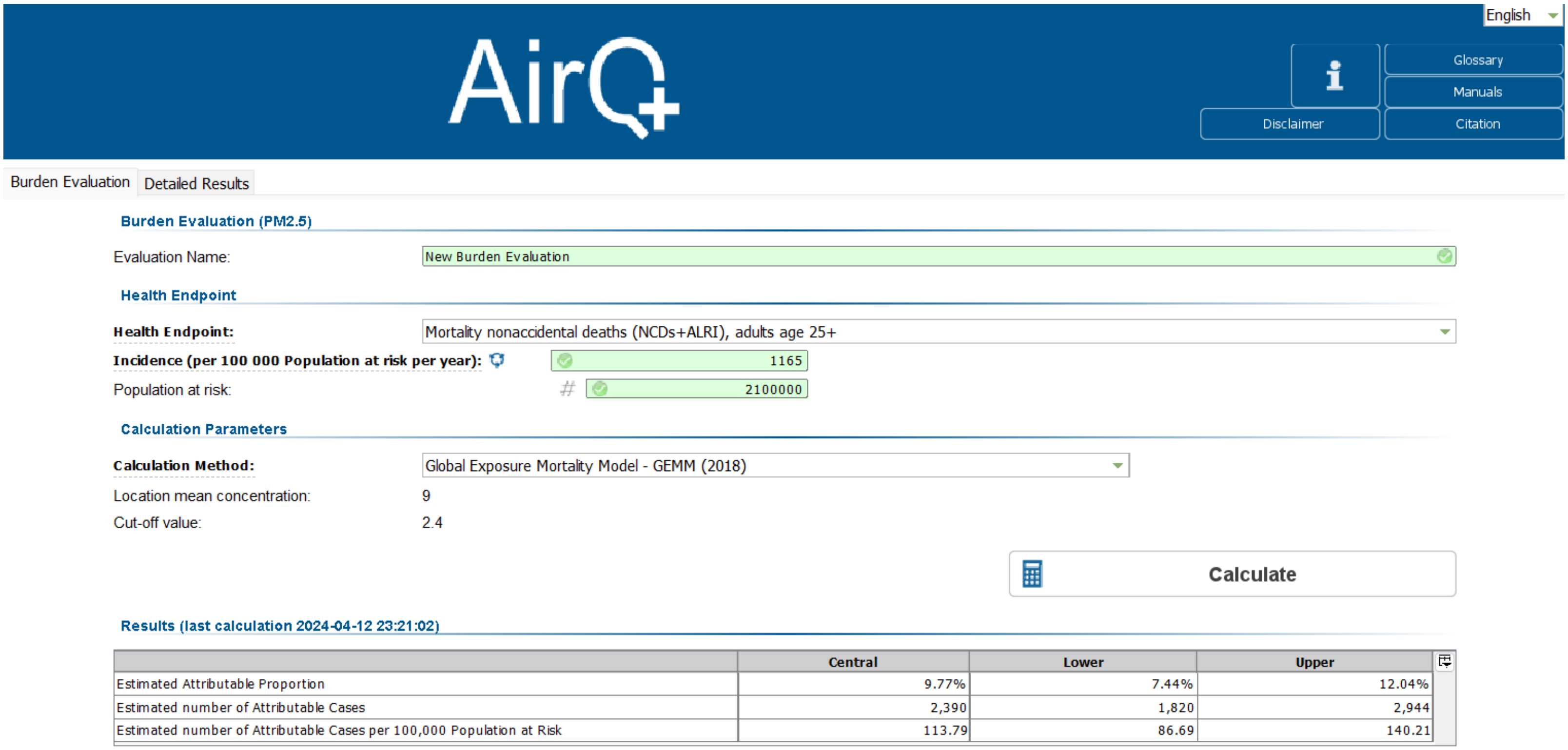
English
AirQ+
Glossary
Manuals
Disclaimer
Citation
Burden Evaluation
Detailed Results
Burden Evaluation (PM2.5)
Evaluation Name: New Burden Evaluation
Health Endpoint
Health Endpoint: Mortality nonaccidental deaths (NCDs+ALRI), adults age 25+
Incidence (per 100 000 Population at risk per year): 1165
Population at risk: 2100000
Calculation Parameters
Calculation Method: Global Exposure Mortality Model - GEMM (2018)
Location mean concentration: 9
Cut-off value: 2.4
Calculate
Results (last calculation 2024-04-12 23:21:02)
Central Lower Upper
Estimated Attributable Proportion 9.77% 7.44% 12.04%
Estimated number of Attributable Cases 2,390 1,820 2,944
Estimated number of Attributable Cases per 100,000 Population at Risk 113.79 86.69 140.21

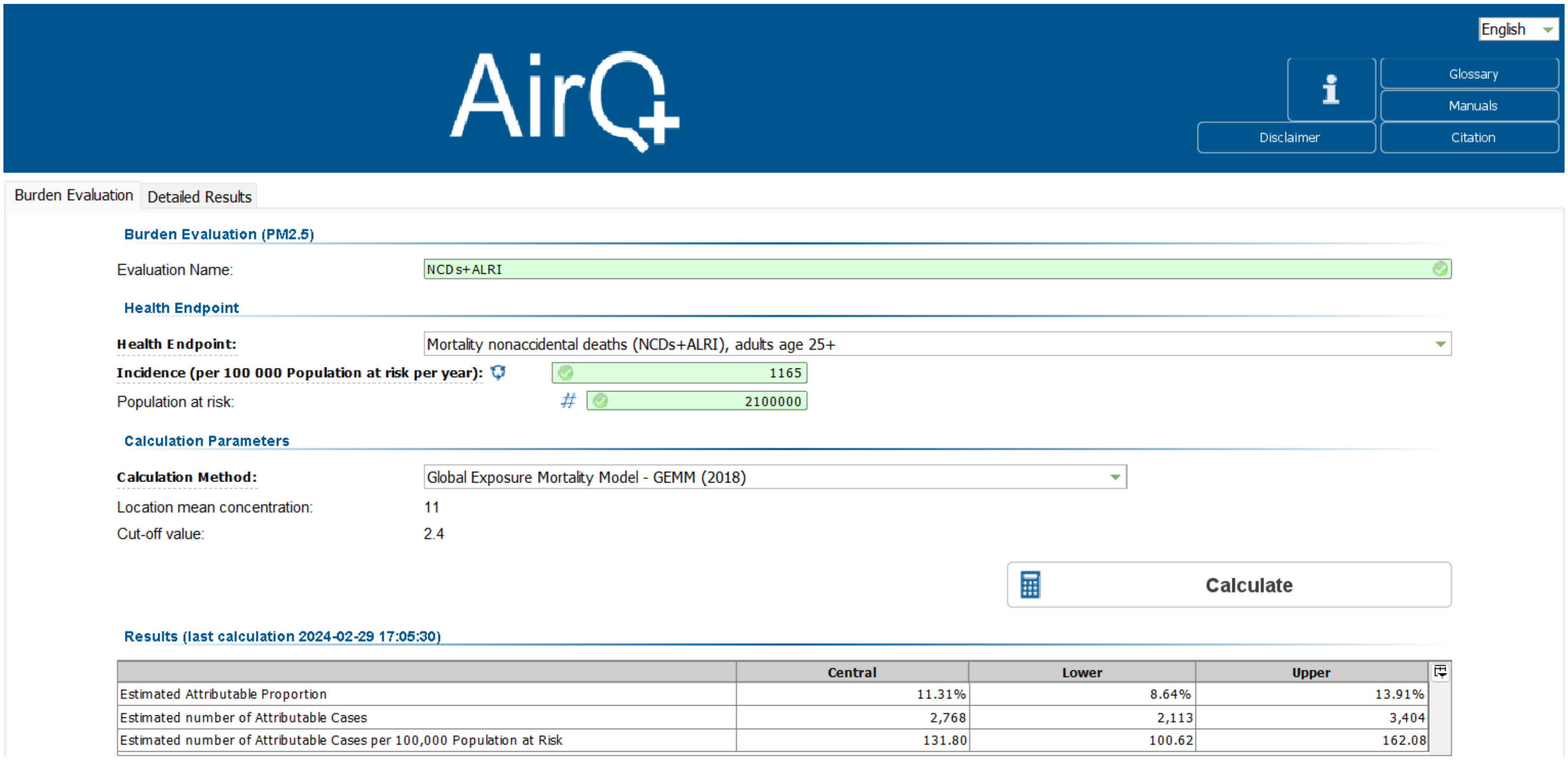
English
AirQ+
Glossary
Manuals
Disclaimer
Citation
Burden Evaluation
Detailed Results
Burden Evaluation (PM2.5)
Evaluation Name: NCDs+ALRI
Health Endpoint
Health Endpoint: Mortality nonaccidental deaths (NCDs+ALRI), adults age 25+
Incidence (per 100 000 Population at risk per year): 1165
Population at risk: 2100000
Calculation Parameters
Calculation Method: Global Exposure Mortality Model - GEMM (2018)
Location mean concentration: 11
Cut-off value: 2.4
Calculate
Results (last calculation 2024-02-29 17:05:30)
Central Lower Upper
Estimated Attributable Proportion 11.31% 8.64% 13.91%
Estimated number of Attributable Cases 2,768 2,113 3,404
Estimated number of Attributable Cases per 100,000 Population at Risk 131.80 100.62 162.08

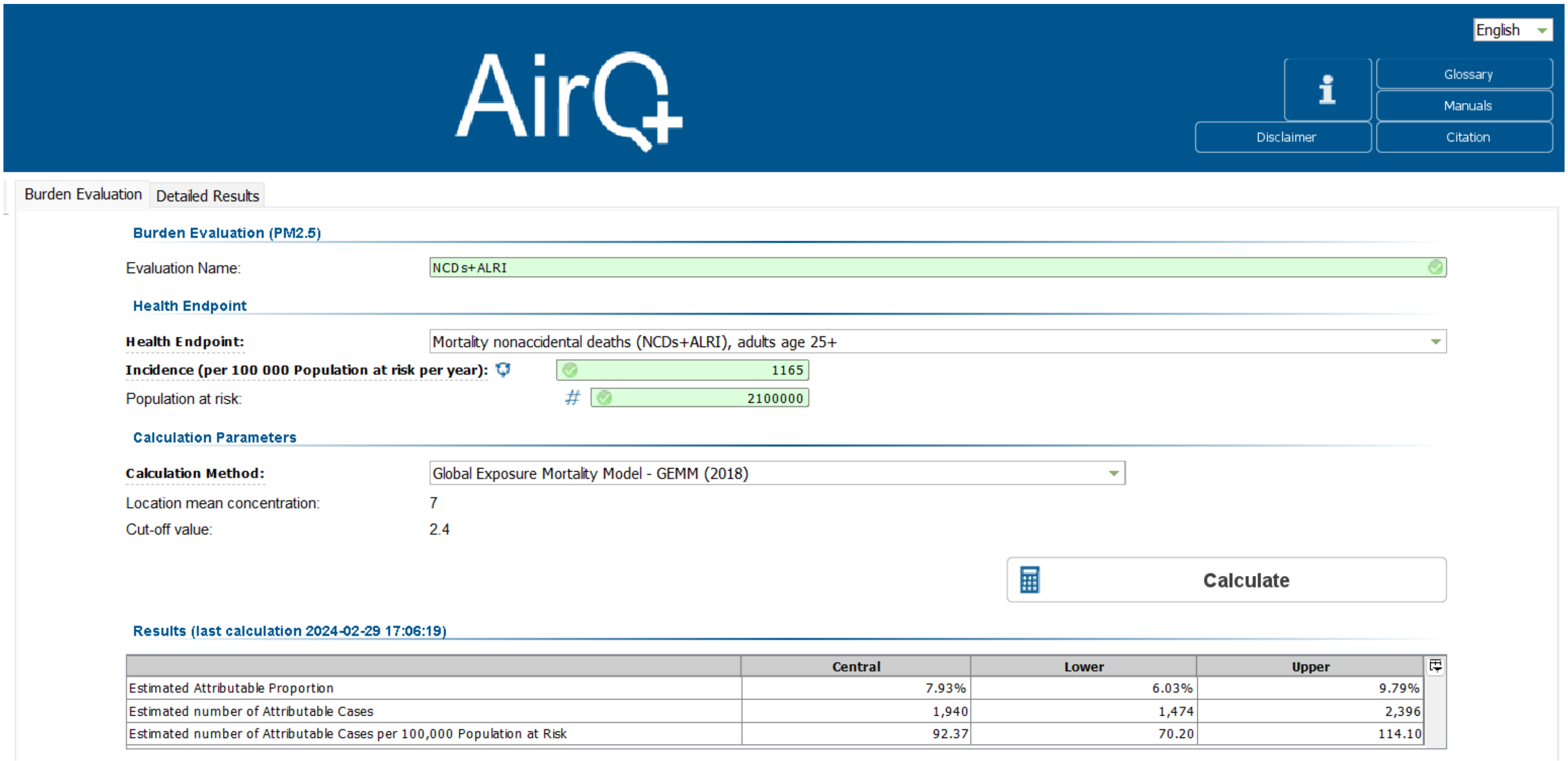


**Figure S17.** The number of all-cause mortality for adults (aged 25+ years) by changing the annual mean of $PM_{2.5}$ concentration from 9 to 11 and 7 μg $m^{-3}$.

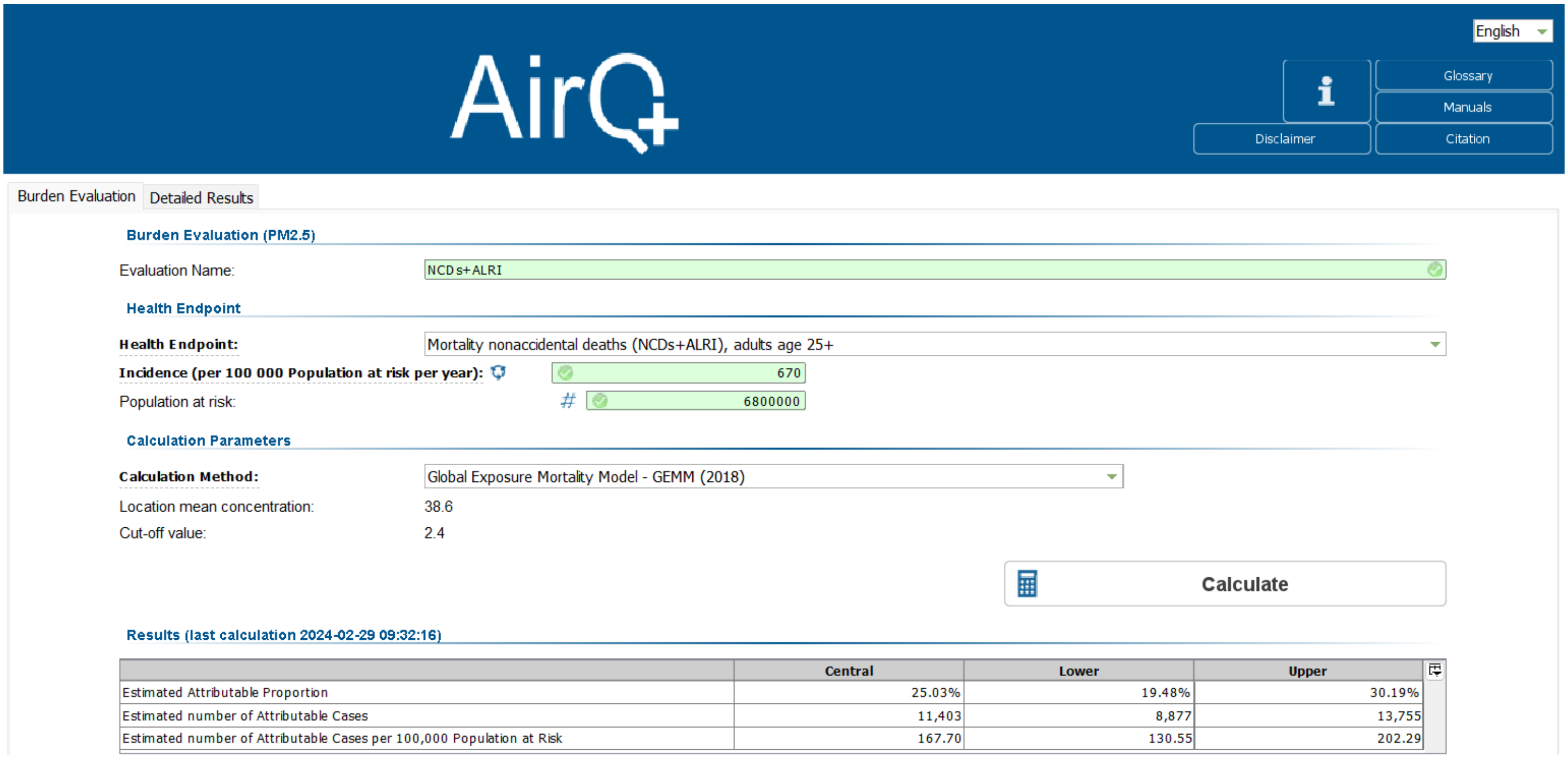
English
AirQ+
Glossary
Manuals
Disclaimer
Citation
Burden Evaluation
Detailed Results
Burden Evaluation (PM2.5)
Evaluation Name: NCDs+ALRI
Health Endpoint
Health Endpoint: Mortality nonaccidental deaths (NCDs+ALRI), adults age 25+
Incidence (per 100 000 Population at risk per year): 670
Population at risk: 6800000
Calculation Parameters
Calculation Method: Global Exposure Mortality Model - GEMM (2018)
Location mean concentration: 38.6
Cut-off value: 2.4
Calculate
Results (last calculation 2024-02-29 09:32:16)
Central | Lower | Upper
Estimated Attributable Proportion | 25.03% | 19.48% | 30.19%
Estimated number of Attributable Cases | 11,403 | 8,877 | 13,755
Estimated number of Attributable Cases per 100,000 Population at Risk | 167.70 | 130.55 | 202.29

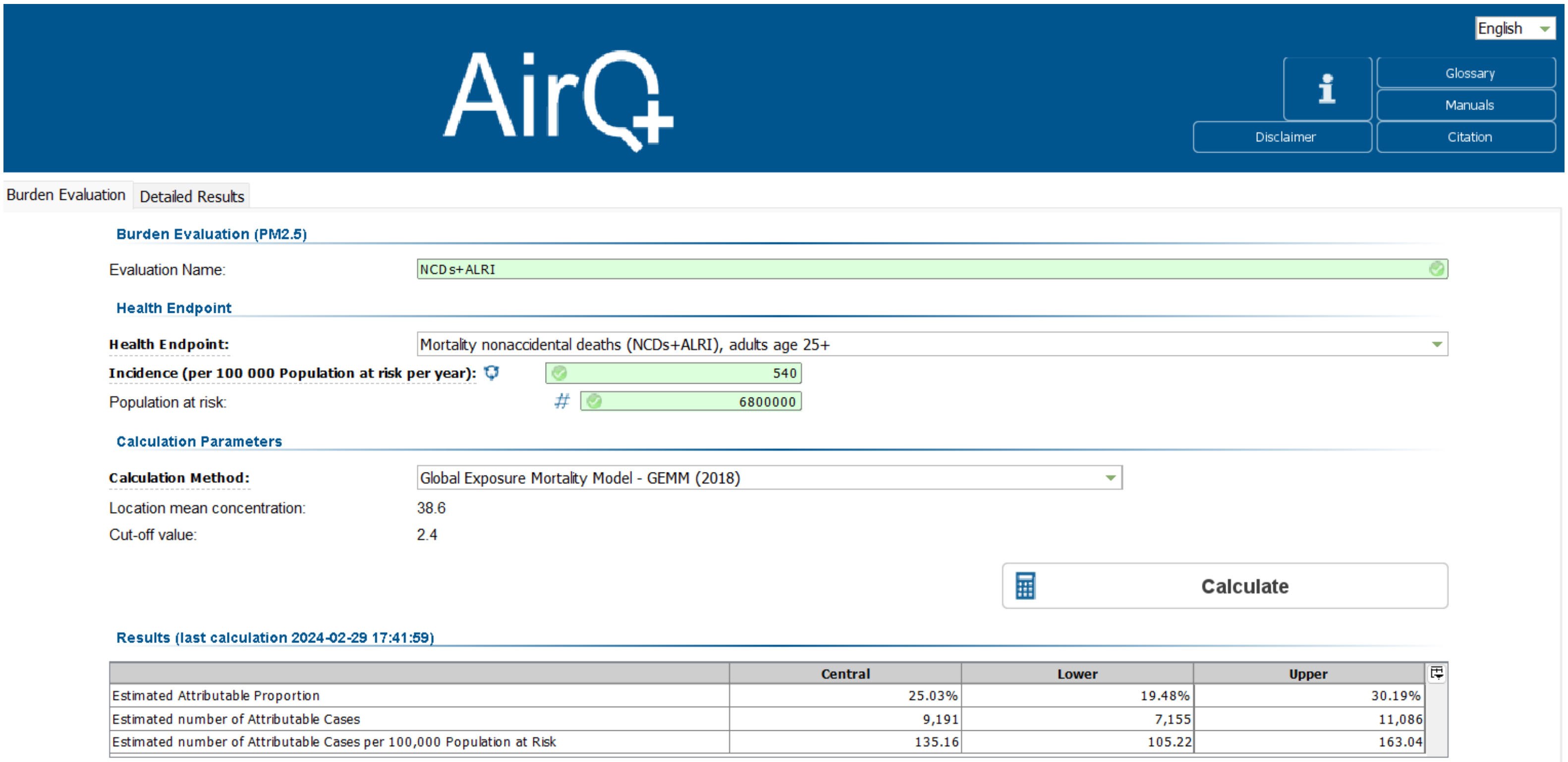
English
AirQ+
Glossary
Manuals
Disclaimer
Citation
Burden Evaluation
Detailed Results
Burden Evaluation (PM2.5)
Evaluation Name: NCDs+ALRI
Health Endpoint
Health Endpoint: Mortality nonaccidental deaths (NCDs+ALRI), adults age 25+
Incidence (per 100 000 Population at risk per year): 540
Population at risk: 6800000
Calculation Parameters
Calculation Method: Global Exposure Mortality Model - GEMM (2018)
Location mean concentration: 38.6
Cut-off value: 2.4
Calculate
Results (last calculation 2024-02-29 17:41:59)
Central | Lower | Upper
Estimated Attributable Proportion | 25.03% | 19.48% | 30.19%
Estimated number of Attributable Cases | 9,191 | 7,155 | 11,086
Estimated number of Attributable Cases per 100,000 Population at Risk | 135.16 | 105.22 | 163.04

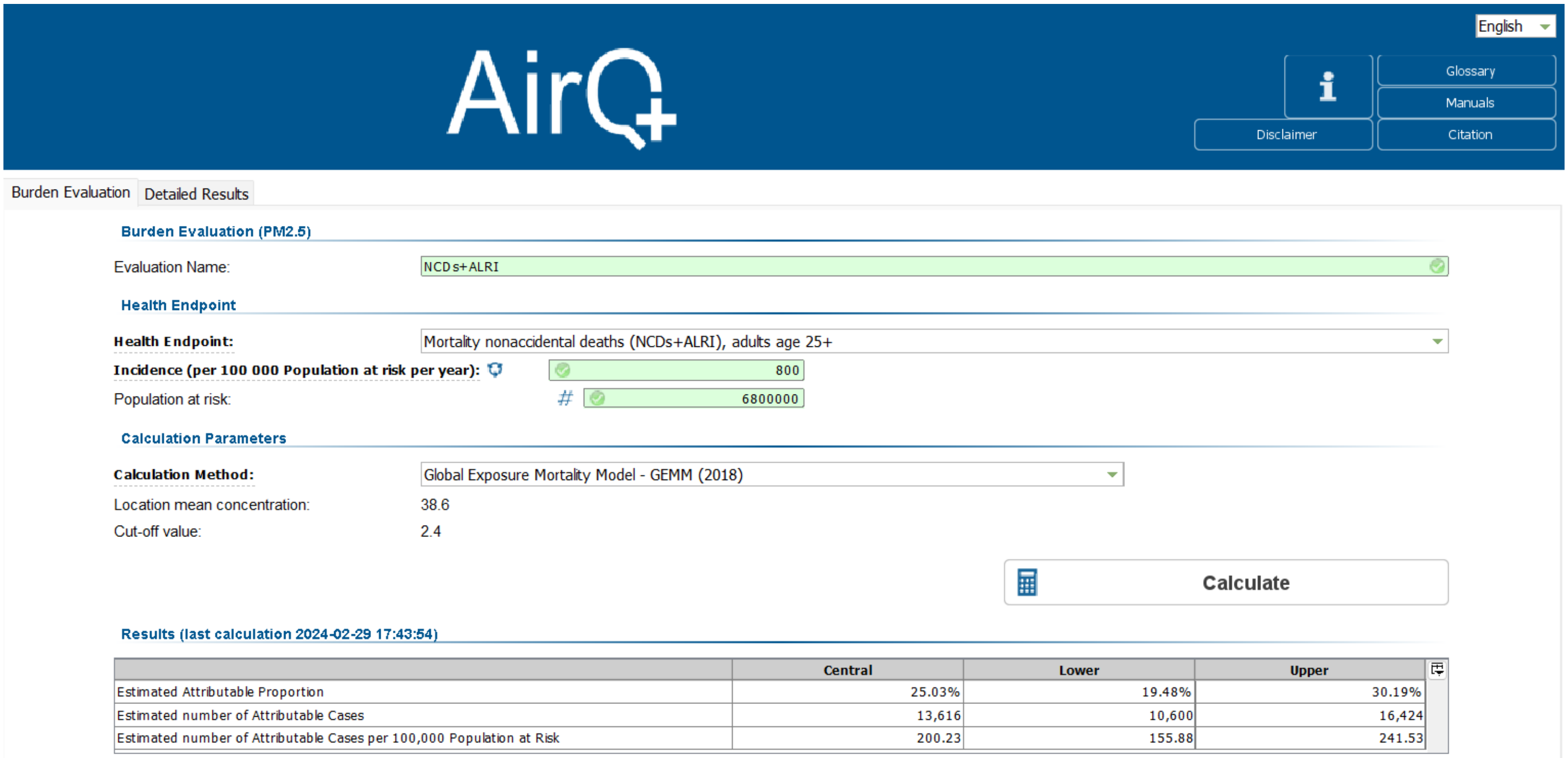


**Figure S18.** The number of all-cause mortality for adults (aged 25+ years) by changing baseline incidence from 670 to 540 and 800 at the annual mean of $PM_{2.5}$ concentration 38.6 μg $m^{-3}$.

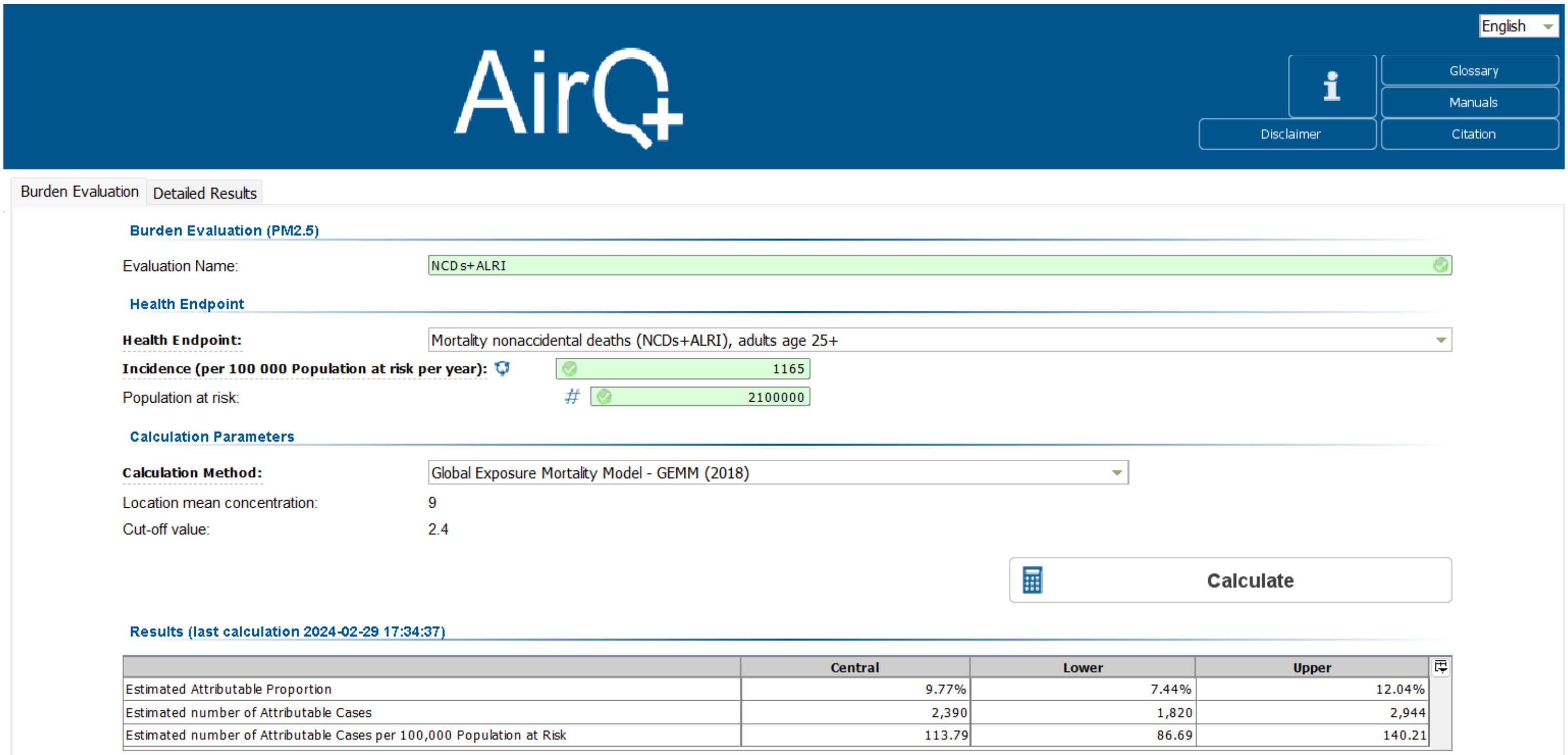
English
AirQ+
Glossary
Manuals
Disclaimer
Citation
Burden Evaluation
Detailed Results
Burden Evaluation (PM2.5)
Evaluation Name: NCDs+ALRI
Health Endpoint
Health Endpoint: Mortality nonaccidental deaths (NCDs+ALRI), adults age 25+
Incidence (per 100 000 Population at risk per year): 1165
Population at risk: 2100000
Calculation Parameters
Calculation Method: Global Exposure Mortality Model - GEMM (2018)
Location mean concentration: 9
Cut-off value: 2.4
Calculate
Results (last calculation 2024-02-29 17:34:37)

| | Central | Lower | Upper |
|---|---|---|---|
| Estimated Attributable Proportion | 9.77% | 7.44% | 12.04% |
| Estimated number of Attributable Cases | 2,390 | 1,820 | 2,944 |
| Estimated number of Attributable Cases per 100,000 Population at Risk | 113.79 | 86.69 | 140.21 |


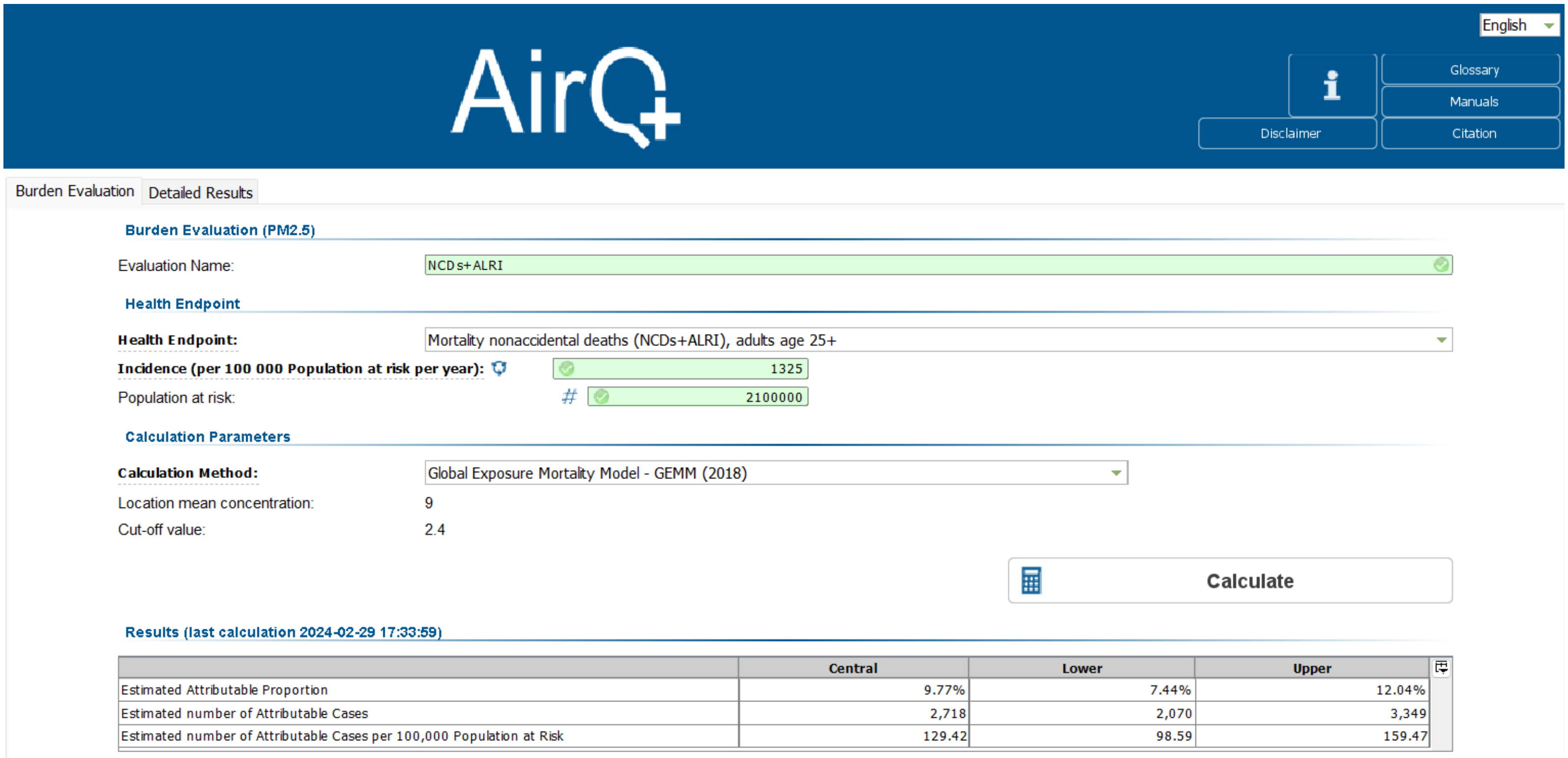
English
AirQ+
Glossary
Manuals
Disclaimer
Citation
Burden Evaluation
Detailed Results
Burden Evaluation (PM2.5)
Evaluation Name: NCDs+ALRI
Health Endpoint
Health Endpoint: Mortality nonaccidental deaths (NCDs+ALRI), adults age 25+
Incidence (per 100 000 Population at risk per year): 1325
Population at risk: 2100000
Calculation Parameters
Calculation Method: Global Exposure Mortality Model - GEMM (2018)
Location mean concentration: 9
Cut-off value: 2.4
Calculate
Results (last calculation 2024-02-29 17:33:59)

| | Central | Lower | Upper |
|---|---|---|---|
| Estimated Attributable Proportion | 9.77% | 7.44% | 12.04% |
| Estimated number of Attributable Cases | 2,718 | 2,070 | 3,349 |
| Estimated number of Attributable Cases per 100,000 Population at Risk | 129.42 | 98.59 | 159.47 |

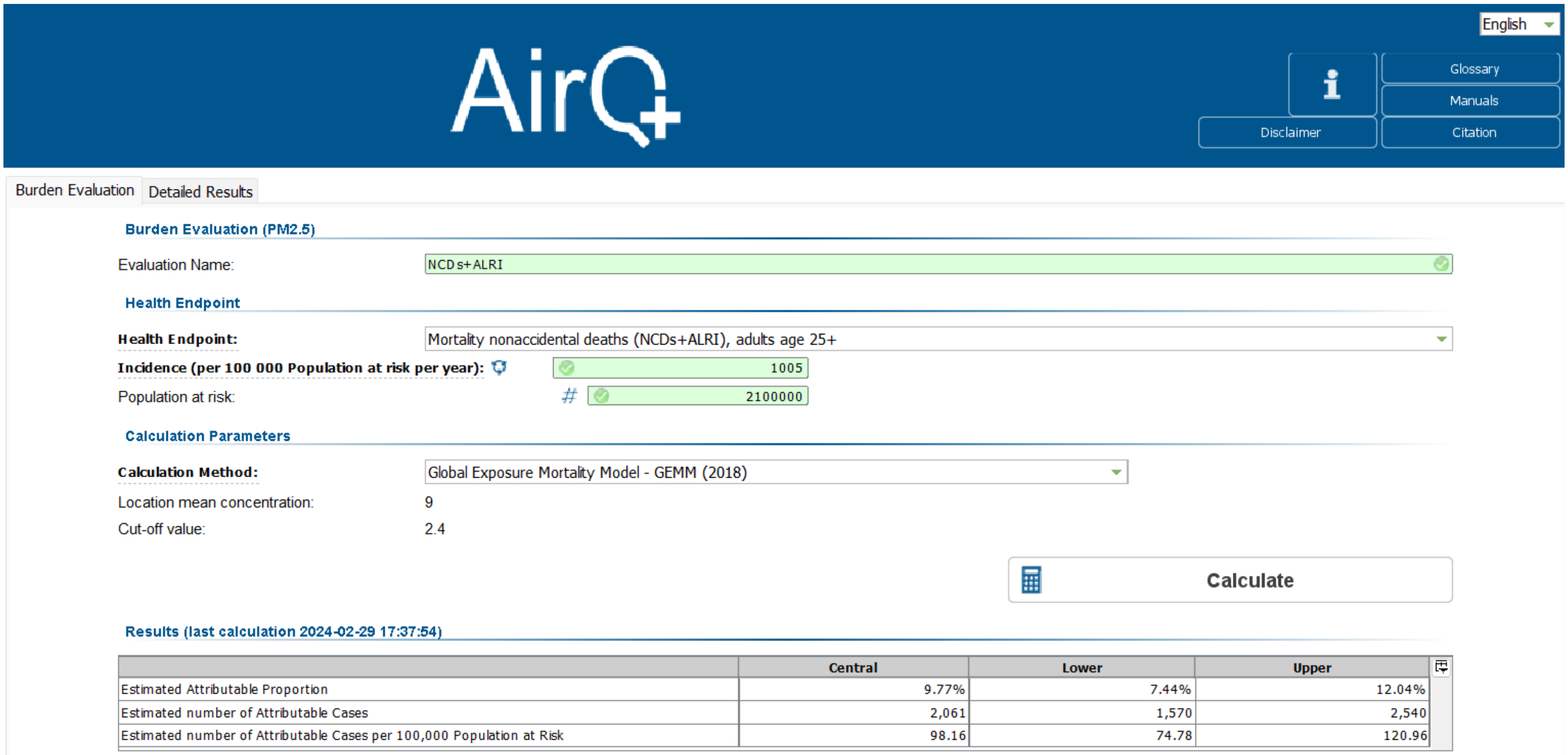


**Figure S19.** The number of all-cause mortality for adults (aged 25+ years) by changing baseline incidence from 1165 to 1325 and 1005 at the annual mean of $PM_{2.5}$ concentration 9 µg $m^{-3}$.

**Table S2.** A summary of included studies that used AirQ for HIA from 2002 to 2022 (Abbreviations used in the table: AFR (African Region), AMR (Region of Americas), SEAR (South-East Asian Region), EUR (European Region), WPR (Western Pacific Region), AQMS (Air Quality Monitoring Station), NR (Not data reported), RR (Relative Risk), BI (Baseline Incidence), CI (Confidence Interval)).

| Author/date | Country | WHO regions (AFR:1, AMR:2, SEAR: 3, EUR:4, EMR:5, WPR:6) | City | Type of publication (Article:1, Report:2, Thesis:3) | Language | Air Quality data | | | | Health endpoints (*Mortality* (All-cause: 1, CVD:2, Respiratory:3, YLG:10; LCM:11, Cardiopulmonary: 12, *Hospitalization* (CVD:4, Respiratory:5, COPD:6, CD:7, AMI:8, YLL:9, life expectancy: 14, Asthma:15, ARI:16, Acute bronchitis:17) | RR Sources | RR Sources (Categorized) | BI Sources | BI Sources (Categorized) | Type of exposure (Long-term (LT)/ Short-term (ST)) | Number of cases reported (Yes/No) | PAF reported (Yes/No) | CI reported (Yes/No) | Sensitivity/ Comparative analyses conducted (Yes/No) | Cut-off value used to estimate health effects reported (Yes/No) | Motivation or context of study | Population size reported (Yes/No) |
|---|---|---|---|---|---|---|---|---|---|---|---|---|---|---|---|---|---|---|---|---|---|---|
| | | | | | | Air Pollutants | AQMS network, Self-measured, Modeling, Satellite data | Data Coverage reported (Yes/No) | Air quality data processing and validation reported (Yes/No) | | | | | | | | | | | | | |
| (Khaefi, Geravandi et al. 2017) | Iran | 5 | Ahvaz | 1 | English | $PM_{10}$ | AQMS | NO | NO | 6 | WHO (Middle East) drawn from Health Effects Association of Particulate Matter | WHO (studies, data, reports and values) | the WHO (Middle East) drawn from Health Effects Association of Particulate Matter | WHO (studies, data, reports and values) | NR | NO | Yes | Yes | NO | per 10 µg/m3 increase of concentration | Research | NO |
| (Omidi Khaniabadi, Sicard et al. 2019) | Iran | 5 | Khorramabad | 1 | English | $PM_{10}$, $PM_{2.5}$, $SO_2$ | AQMS | More than 75% of validated data | NO | 1,2,3,6,7,8 | WHO | WHO (studies, data, reports and values) | WHO | WHO (studies, data, reports and values) | ST | Yes | Yes | Yes | NO | per 10 µg/m3 increase of concentration | Research | NO |
| (Javan, Rahdar et al. 2021) | Iran | 5 | Zabol | 1 | English | $PM_{10}$ | AQMS | NO | NO | 1, 2,3,4,5 | the time-series studies | Previous studies | Local | Local | ST | Yes | Yes | Yes | NO | per 10 µg/m3 increase of concentration | Research | NO |
| (Effatpanah, Effatpanah et al. 2020) | Iran | 5 | Ahvaz | 1 | English | $PM_{10}$, $O_3$, $NO_2$, SO2, PM1 | AQMS | NO | NO | 4,5,6,7,8 | World Health Organization (WHO) for Middle East region | WHO (studies, data, reports and values) | World Health Organization (WHO) for Middle East region | WHO (studies, data, reports and values) | NR | Yes | Yes | Yes | NO | NO | Research | NO |
| (Luo, Guan et al. 2020) | China | 6 | Six cities: Loess Plateau, Tibetan Plateau, and Mongolian Plateau. | 1 | English | $PM_{10}$, $PM_{2.5}$, NO2, SO2, O3, CO | AQMS | NO | NO | 1, 2, 3 | Previous studies | Previous studies | average mortality data from 2015 to 2018 | Local | NR | Yes | Yes | Yes | NO | per 10 µg/m3 increase of concentration | Research | NO |
| (Gholampour, Nabizadeh et al. 2014) | Iran | 5 | Tabriz | 1 | English | TSP, $PM_{10}$, PM2.5 | Self-measured (Urban residential, industrial suburban) | NO | NO | 1,2,3,5,6,7 | Previous studies | Previous studies | Previous studies | Previous studies | ST | Yes | Yes | Yes | NO | NO | Research | NO |
| (Kermani, Fallah Jokandan et al. 2016) | Iran | 5 | Tehran, Mashhad, Tabriz, Isfahan, Shiraz, Urmia | 1 | English | $SO_2$ | AQMS | NO | NO | 4,6 | Previous studies | Previous studies | Previous studies | Previous studies | ST | Yes | Yes | Yes | NO | per 10 µg/m3 increase of concentration | Research | NO |
| (Hadei, Hopke et al. 2017) | Iran | 5 | Tehran | 1 | English | $PM_{10}$, $PM_{2.5}$, O3, NO2, SO2 | AQMS | NO | deletion of zero and negative data | 1, 2, 3, 4, 5, 6, 8 | Previous studies | Previous studies | Local | Local | ST | Yes | Yes | Yes | NO | per 10 µg/m3 increase of concentration | Research | NO |

| Author/date | Country | WHO regions (AFR:1, AMR:2, SEAR: 3, EUR:4, EMR:5, WPR:6) | City | Type of publication (Article:1, Report:2, Thesis:3) | Language | Air Quality data | | | | Health endpoints (*Mortality* (All-cause: 1, CVD:2, Respiratory:3, YLG:10; LCM:11, Cardiopulmonary: 12, *Hospitalization* (CVD:4, Respiratory:5, COPD:6, CD:7, AMI:8, YLL:9, life expectancy: 14, Asthma:15, ARI:16, Acute bronchitis:17) | RR Sources | RR Sources (Categorized) | BI Sources | BI Sources (Categorized) | Type of exposure (Long-term (LT)/ Short-term (ST)) | Number of cases reported (Yes/No) | PAF reported (Yes/No) | CI reported (Yes/No) | Sensitivity/ Comparative analyses conducted (Yes/No) | Cut-off value used to estimate health effects reported (Yes/No) | Motivation or context of study | Population size reported (Yes/No) |
|---|---|---|---|---|---|---|---|---|---|---|---|---|---|---|---|---|---|---|---|---|---|---|
| | | | | | | Air Pollutants | AQMS network, Self-measured, Modeling, Satellite data | Data Coverage reported (Yes/No) | Air quality data processing and validation reported (Yes/No) | | | | | | | | | | | | | |
| (Mohammed, Ibrahim et al. 2019) | Egypt | 5 | Ain Sokhna sector, Shoubra El-Khaimasector | 1 | English | $SO_2$, $NO_2$ | Self-measured (Residential, industrial) | NO | NO | 5 | Previous studies | Previous studies | NR | NR | ST | Yes | Yes | Yes | NO | each 10 μg/m3 increase in daily concentration | Research | NO |
| (Asl, Leili et al. 2018) | Iran | 5 | Hamedan | 1 | English | $NO_2$, $SO_2$, $O_3$, CO, PM10, PM2.5 | AQMS | NO | NO | 1, 2,3 | Previous studies | Previous studies | Previous studies | Previous studies | ST | Yes | Yes | Yes | NO | each 10 μg/m3 increase in daily concentration | Research | NO |
| (Khaniabadi, Goudarzi et al. 2017) | Iran | 5 | Kermanshah | 1 | English | $PM_{10}$, $NO_2$, $O_3$ | AQMS | NO | NO | 2 | Previous studies | Previous studies | NR | NR | ST | Yes | Yes | Yes | NO | per 10 μg/m3 increase of concentration | Research | NO |
| (Fattore, Paiano et al. 2011) | Italy | 4 | Mazzano, Rezzato | 1 | English | $O_3$, $NO_2$, $PM_{2.5}$, $PM_{10}$ | AQMS | NO | NO | 1,2,3,9 | NO2, O3 and PM10: Previous studies PM2.5: WHO Air Quality Guidelines for Europe | Previous studies/ International | statistics available on-line from the Brescia district local health authority | Local | ST/LT | Yes | Yes | Yes | NO | per 10 μg/m3 increase of concentration | Research | 30-95 |
| (Javanmardi, Morovati et al. 2018) | Iran | 5 | Ahvaz | 1 | English | $O_3$ | AQMS and modeling | NO | NO | 2, 8 | World Health Organization (WHO) database | WHO (studies, data, reports and values) | WHO database | WHO (studies, data, reports and values) | ST | Yes | Yes | NO | NO | per 10 μg/m3 increase of concentration | Research | NO |
| (Ghaffari, Aval et al. 2017) | Iran | 5 | Mashhad | 1 | English | $NO_2$, $SO_2$, $O_3$ | AQMS | NO | NO | 15 | Previous studies | Previous studies | WHO database | WHO (studies, data, reports and values) | ST | Yes | Yes | Yes | NO | each 10 μg/m3 increase in daily concentration | Research | less than 15 and from 15 to 64 years old |
| (Ghozikali, Heibati et al. 2016) | Iran | 5 | Tabriz | 1 | English | $O_3$, $NO_2$, $SO_2$ | AQMS | NO | NO | 6 | World Health Organization (WHO) database | WHO (studies, data, reports and values) | WHO database | WHO (studies, data, reports and values) | ST | Yes | Yes | Yes | NO | each 10 μg/m3 increase in daily concentration | Research | NO |
| (Goudarzi, Geravandi et al. 2016) | Iran | 5 | Ahvaz | 1 | English | $SO_2$ | AQMS | NO | NO | 5 | WHO data | WHO (studies, data, reports and values) | WHO database | WHO (studies, data, reports and values) | NR | Yes | Yes | NO | NO | each 10 μg/m3 increase in daily concentration | Research | NO |
| (Maji, Dikshit et al. 2016) | India | 3 | Maharashtra | 1 | English | $PM_{10}$, $SO_2$, $NO_2$ | AQMS | NO | NO | 1,2,3, 4,5, 6 | World Health Organization recommended value | WHO (studies, data, reports and values) | WHO database | WHO (studies, data, reports and values) | NR | Yes | NO | Yes | NO | per 10 μg/m3 increase of concentration | Research | NO |

| Author/date | Country | WHO regions (AFR:1, AMR:2, SEAR: 3, EUR:4, EMR:5, WPR:6) | City | Type of publication (Article:1, Report:2, Thesis:3) | Language | Air Quality data | | | | Health endpoints (*Mortality* (All-cause: 1, CVD:2, Respiratory:3, YLG:10; LCM:11, Cardiopulmonary: 12, *Hospitalization* (CVD:4, Respiratory:5, COPD:6, CD:7, AMI:8, YLL:9, life expectancy: 14, Asthma:15, ARI:16, Acute bronchitis:17) | RR Sources | RR Sources (Categorized) | BI Sources | BI Sources (Categorized) | Type of exposure (Long-term (LT)/ Short-term (ST)) | Number of cases reported (Yes/No) | PAF reported (Yes/No) | CI reported (Yes/No) | Sensitivity/ Comparative analyses conducted (Yes/No) | Cut-off value used to estimate health effects reported (Yes/No) | Motivation or context of study | Population size reported (Yes/No) |
|---|---|---|---|---|---|---|---|---|---|---|---|---|---|---|---|---|---|---|---|---|---|---|
| | | | | | | Air Pollutants | AQMS network, Self-measured, Modeling, Satellite data | Data Coverage reported (Yes/No) | Air quality data processing and validation reported (Yes/No) | | | | | | | | | | | | | |
| (Abdolahnejad, Jafari et al. 2017) | Iran | 5 | Isfahan | 1 | English | $NO_2$, $SO_2$, $O_3$ | AQMS | NO | NO | 1,2,3,6,8 | Previous studies | Previous studies | WHO database | WHO (studies, data, reports and values) | ST/LT | Yes | Yes | Yes | NO | per 10 µg/m3 increase of concentration | Research | NO |
| (Khaniabadi, Polosa et al. 2018) | Iran | 5 | Khorramabad | 1 | English | $PM_{10}$, $SO_2$ | AQMS | NO | NO | 1,2,3 | WHO European Center for Environment and Health based on various peer-reviewed studies conducted | WHO (studies, data, reports and values) | WHO European Center for Environment and Health based on various peer-reviewed studies conducted | WHO (studies, data, reports and values) | ST | Yes | Yes | Yes | NO | PM10 and SO2 were recorded into 10 µg/m3 categories | Research | NO |
| (Marzouni, Alizadeh et al. 2016) | Iran | 5 | Kermanshah | 1 | English | $PM_{10}$ | AQMS | NO | NO | 1,2,3,4,5 | previous meta-analysis studies | Previous studies | Previous meta-analysis studies | Previous studies | ST | Yes | Yes | Yes | NO | per 10 µg/m3 increase of concentration | Dust storm | NO |
| (Jeong 2013) | Korea | 6 | Suwon | 1 | English | $PM_{10}$, $O_3$, $NO_2$, SO2 | AQMS | NO | NO | 1,2,3,4,5,6,8 | PM10: Previous studies, For O3, SO2 and NO2, the RR values came directly from published studies on short-term effects within the APHEA project | Previous studies | National | National | ST | Yes | Yes | Yes | NO | per 10 µg/m3 increase of concentration | Research | NO |
| (Miri, Derakhshan et al. 2016) | Iran | 5 | Mashhad | 1 | English | $PM_{10}$, $PM_{2.5}$, $O_3$, $NO_2$, $SO_2$ | AQMS | NO | NO | 1,2,3,4,5,6,8 | Previous studies | Previous studies | Local | Local | ST | Yes | Yes | Yes | NO | per 10 µg/m3 increase of concentration | Research | NO |
| (Marzouni, Moradi et al. 2017) | Iran | 5 | Ahvaz, Isfahan, Shiraz, Tehran | 1 | English | $PM_{10}$ | AQMS | NO | NO | 1,2,3,4,5 | epidemiological studies | Previous studies | Previous studies | Previous studies | ST | Yes | NO | Yes | NO | per 10 µg/m3 increase of concentration | Research, Dust storm | NO |
| (Sokoty, Kermani et al. 2020) | Iran | 5 | Urmia | 1 | English | $PM_{10}$ | AQMS | The ratio of warm seasons/cold seasons needed to be less than 2. Also, | Yes | 4,5 | Previous studies | Previous studies | Previous studies | Previous studies | ST | Yes | Yes | Yes | NO | per 10 µg/m3 increase of concentration | Research | NO |

| Author/date | Country | WHO regions (AFR:1, AMR:2, SEAR:3, EUR:4, EMR:5, WPR:6) | City | Type of publication (Article:1, Report:2, Thesis:3) | Language | Air Quality data | | | | Health endpoints (*Mortality* (All-cause: 1, CVD:2, Respiratory:3, YLG:10; LCM:11, Cardiopulmonary: 12, *Hospitalization* (CVD:4, Respiratory:5, COPD:6, CD:7, AMI:8, YLL:9, life expectancy: 14, Asthma:15, ARI:16, Acute bronchitis:17) | RR Sources | RR Sources (Categorized) | BI Sources | BI Sources (Categorized) | Type of exposure (Long-term (LT)/ Short-term (ST)) | Number of cases reported (Yes/No) | PAF reported (Yes/No) | CI reported (Yes/No) | Sensitivity/ Comparative analyses conducted (Yes/No) | Cut-off value used to estimate health effects reported (Yes/No) | Motivation or context of study | Population size reported (Yes/No) |
|---|---|---|---|---|---|---|---|---|---|---|---|---|---|---|---|---|---|---|---|---|---|---|
| | | | | | | Air Pollutants | AQMS network, Self-measured, Modeling, Satellite data | Data Coverage reported (Yes/No) | Air quality data processing and validation reported (Yes/No) | | | | | | | | | | | | | |
| | | | | | | | | 50% of the data needed to have enough validity to estimate the daily mean | | | | | | | | | | | | | | |
| (Goudarzi, Daryanoosh et al. 2017) | Iran | 5 | Kermanshah | 1 | English | $PM_{10}$ | AQMS | NO | NO | 2,3, 4,5,6 | WHO European Center for Environment and Health based on various peer-reviewed studies | Previous studies/ International | WHO European Center for Environment and Health based on various peer-reviewed studies | WHO (studies, data, reports and values) | ST | Yes | Yes | NO | NO | per 10 µg/m3 increase of concentration | Dust storm | NO |
| (Orru, Laukaitienė et al. 2012) | Lithuania | 4 | Vilnius, Kaunas | 1 | English | $PM_{10}$, $PM_{2.5}$ | AQMS and modeling | NO | NO | 1,4,5 | Previous studies | Previous studies | National | National | ST/LT | Yes | NO | Yes | NO | NO | Research | NO |
| (Orru, Kaasik et al. 2009) | Lithuania | 4 | Tartu | 1 | English | $PM_{2.5}$ | AQMS and modeling | NO | NO | 9 | recommended RR in the AirQ software. the calculations in this study were made also with RR from the Jerrett et al. | AirQ default values/ Previous studies | NR | NR | ST | Yes | NO | Yes | NO | NO | Burning of peat | NO |
| (Samek 2016) | Poland | 4 | Kraków | 1 | English | $PM_{10}$ | AQMS (Industry, residential and traffic stations) | NO | Any missing air pollution data in a specific monitoring station was replaced by an average of the corresponding values for the other monitoring stations. | 1,2,3,4,5 | WHO default values | AirQ default values | WHO default values | AirQ default values | NR | Yes | Yes | Yes | NO | NO | Research | NO |

| Author/date | Country | WHO regions (AFR:1, AMR:2, SEAR:3, EUR:4, EMR:5, WPR:6) | City | Type of publication (Article:1, Report:2, Thesis:3) | Language | Air Quality data | | | | Health endpoints (*Mortality* (All-cause: 1, CVD:2, Respiratory:3, YLG:10; LCM:11, Cardiopulmonary: 12, *Hospitalization* (CVD:4, Respiratory:5, COPD:6, CD:7, AMI:8, YLL:9, life expectancy: 14, Asthma:15, ARI:16, Acute bronchitis:17) | RR Sources | RR Sources (Categorized) | BI Sources | BI Sources (Categorized) | Type of exposure (Long-term (LT)/ Short-term (ST)) | Number of cases reported (Yes/No) | PAF reported (Yes/No) | CI reported (Yes/No) | Sensitivity/ Comparative analyses conducted (Yes/No) | Cut-off value used to estimate health effects reported (Yes/No) | Motivation or context of study | Population size reported (Yes/No) |
|---|---|---|---|---|---|---|---|---|---|---|---|---|---|---|---|---|---|---|---|---|---|---|
| | | | | | | Air Pollutants | AQMS network, Self-measured, Modeling, Satellite data | Data Coverage reported (Yes/No) | Air quality data processing and validation reported (Yes/No) | | | | | | | | | | | | | |
| (Mohammadi, Geravandi et al. 2017) | Iran | 5 | Bushehr, Kermanshah, Ahvaz | 1 | English | CO | AQMS | NO | NO | 2 | APP default | AirQ default values | APP default | AirQ default values | ST | Yes | Yes | NO | NO | NO | Research | NO |
| (Maji, Dikshit et al. 2017) | India | 3 | Agra | 1 | English | $NO_2$, $SO_2$, $PM_{10}$ | AQMS and modeling | NO | NO | 1,2,3,4,5,6 | Previous studies | Previous studies | Previous studies | Previous studies | ST | Yes | Yes | Yes | NO | NO | Research | NO |
| (Nikoonahad, Naserifar et al. 2017) | Iran | 5 | Illam | 1 | English | $PM_{10}$ | AQMS | NO | NO | 1,2,3,4,5 | Previous studies | Previous studies | Previous studies | Previous studies | ST | Yes | Yes | NO | NO | per 10 µg/m3 increase of concentration | Research | NO |
| (Maji, Dikshit et al. 2017) | India | 3 | Mumbai | 1 | English | $PM_{10}$, $SO_2$, $NO_2$ | AQMS and modeling | NO | NO | 1,2,3,4,5,6 | WHO default value | AirQ default values | WHO default value | AirQ default values | LT | Yes | NO | Yes | NO | NO | Research | NO |
| (Miri, Ebrahimi Aval et al. 2017) | Iran | 5 | Yazd | 1 | English | $PM_{10}$ | AQMS (traffic station) | More than 60% of days | NO | 1,2,3,4,5 | Previous studies | Previous studies | Local | Local | ST | Yes | Yes | Yes | NO | per 10 µg/m3 increase of concentration | Research | NO |
| (Mohammadi, Azhdarpoor et al. 2016) | Iran | 5 | Shiraz | 1 | English | $PM_{10}$, $SO_2$, $NO_2$, $O_3$ | AQMS | NO | NO | 1,2,3,4,5, | obtained from the WHO tool for health impact assessment of air quality | AirQ default values | WHO default value | AirQ default values | ST | Yes | NO | Yes | NO | per 10 µg/m3 increase of concentration | Research | 15–64 age group, And ≥ 65 |
| (Omidi Khaniabadi, Sicard et al. 2018) | Iran | 5 | Hamedan | 1 | English | $PM_{10}$, $SO_2$ | AQMS | NO | NO | 1,2,4,5,6,8 | Previous studies | Previous studies | Previous studies | Previous studies | ST | Yes | Yes | Yes | NO | per 10 µg/m3 increase of concentration | Research | NO |
| (Ghozikali, Borgini et al. 2015) | Iran | 5 | Tabriz | 1 | English | $NO_2$ | AQMS (residential, commercial, and industrial stations) | NO | NO | 2,6,8 | WHO report | WHO (studies, data, reports and values) | WHO report | WHO (studies, data, reports and values) | ST | Yes | Yes | Yes FOR AP ONLY | NO | per 10 µg/m3 increase of concentration | Research | NO |
| (Khaniabadi, Hopke et al. 2017) | Iran | 5 | Kermanshah | 1 | English | $O_3$ | AQMS | NO | NO | 2,3,6 | Previous studies | Previous studies | Previous studies | Previous studies | ST | Yes | Yes | Yes | NO | per 10 µg/m3 increase of concentration | Research | NO |

| **Author/date** | **Country** | **WHO regions (AFR:1, AMR:2, SEAR: 3, EUR:4, EMR:5, WPR:6)** | **City** | **Type of publication (Article:1, Report:2, Thesis:3)** | **Language** | **Air Quality data** | | | | **Health endpoints (*Mortality* (All-cause: 1, CVD:2, Respiratory:3, YLG:10; LCM:11, Cardiopulmonary: 12, *Hospitalization* (CVD:4, Respiratory:5, COPD:6, CD:7, AMI:8, YLL:9, life expectancy: 14, Asthma:15, ARI:16, Acute bronchitis:17)** | **RR Sources** | **RR Sources (Categorized)** | **BI Sources** | **BI Sources (Categorized)** | **Type of exposure (Long-term (LT)/ Short-term (ST))** | **Number of cases reported (Yes/No)** | **PAF reported (Yes/No)** | **CI reported (Yes/No)** | **Sensitivity/ Comparative analyses conducted (Yes/No)** | **Cut-off value used to estimate health effects reported (Yes/No)** | **Motivation or context of study** | **Population size reported (Yes/No)** |
|---|---|---|---|---|---|---|---|---|---|---|---|---|---|---|---|---|---|---|---|---|---|---|
| | | | | | | **Air Pollutants** | **AQMS network, Self-measured, Modeling, Satellite data** | **Data Coverage reported (Yes/No)** | **Air quality data processing and validation reported (Yes/No)** | | | | | | | | | | | | | |
| (Miri, Alahabadi et al. 2018) | Iran | 5 | Sabzevar | 1 | English | $PM_{2.5}$, $PM_{10}$ | AQMS | 159 days of 2017 | PM2.5 (Z-score = 2.53; P-value = 0.011) and PM10 (Z-score = 2.82; P-value = 0.004) were clustered. Given the z-score of 2.53 for PM2.5 and 2.82 for PM10. | 1,2,3,4,5 | Previous studies | Previous studies | Local | Local | ST | Yes | Yes | Yes | NO | NO | Research for traffic (the stormy days were excluded) | NO |
| (Bonyadi, Arfaeinia et al. 2020) | Iran | 5 | Shiraz | 1 | English | $PM_{10}$, $PM_{2.5}$, $O_3$, $NO_2$, $SO_2$ | AQMS | NO | NO | 1,2,3,4,5,6,8 | Previous studies | Previous studies | NR | NR | ST | Yes | Yes | Yes | NO | per 10 µg/m3 increase of concentration | Research | NO |
| (Bonyadi, Arfaeinia et al. 2020) | Iran | 5 | Shiraz | 1 | English | $O_3$, $NO_2$, $SO_2$ | AQMS | The ratio of the number of valid data for the summer season to the winter data and vice versa should not be more than 2 and to achieve an hourly average of data, at least 75% of the data must | NO | 15 | Previous studies | Previous studies | WHO value | WHO (studies, data, reports and values) | ST | Yes | Yes | Yes | NO | NO | Research | NO |

| Author/date | Country | WHO regions (AFR:1, AMR:2, SEAR: 3, EUR:4, EMR:5, WPR:6) | City | Type of publication (Article:1, Report:2, Thesis:3) | Language | Air Quality data | | | | Health endpoints (*Mortality* (All-cause: 1, CVD:2, Respiratory:3, YLG:10; LCM:11, Cardiopulmonary: 12, *Hospitalization* (CVD:4, Respiratory:5, COPD:6, CD:7, AMI:8, YLL:9, life expectancy: 14, Asthma:15, ARI:16, Acute bronchitis:17) | RR Sources | RR Sources (Categorized) | BI Sources | BI Sources (Categorized) | Type of exposure (Long-term (LT)/ Short-term (ST)) | Number of cases reported (Yes/No) | PAF reported (Yes/No) | CI reported (Yes/No) | Sensitivity/ Comparative analyses conducted (Yes/No) | Cut-off value used to estimate health effects reported (Yes/No) | Motivation or context of study | Population size reported (Yes/No) |
|---|---|---|---|---|---|---|---|---|---|---|---|---|---|---|---|---|---|---|---|---|---|---|
| | | | | | | Air Pollutants | AQMS network, Self-measured, Modeling, Satellite data | Data Coverage reported (Yes/No) | Air quality data processing and validation reported (Yes/No) | | | | | | | | | | | | | |
| | | | | | | | | be valid. | | | | | | | | | | | | | | |
| (Dehghani, Hopke et al. 2020) | Iran | 5 | Tabriz | 1 | English | $PM_{10}$ | AQMS (commercial, residential, and industrial stations) | NO | NO | 1,2,3,4,5 | WHO from epidemiological studies | WHO (studies, data, reports and values) | WHO values from epidemiological studies | WHO (studies, data, reports and values) | NR | Yes | Yes | Yes | NO | NO | decreasing level of Urmia Lake | NO |
| (Khaniabadi, Daryanoosh et al. 2017) | Iran | 5 | Khorramabad | 1 | English | $SO_2$ | AQMS | For each day when more than 75% of validated hourly data were available, 24-h mean concentrations were calculated. | Yes | 6,8 | peer-reviewed studies | Previous studies | peer-reviewed studies | Previous studies | ST | Yes | Yes | Yes | NO | NO | Research | NO |
| (Khaniabadi, Daryanoosh et al. 2018) | Iran | 5 | Kermanshah | 1 | English | $PM_{10}$, $O_3$, $NO_2$, $SO_2$ | AQMS | NO | NO | 6 | peer-reviewed studies | Previous studies | WHO value | WHO (studies, data, reports and values) | ST | Yes | Yes | Yes | NO | per 10 µg/m3 increase of concentration | Research | NO |
| (Bonyadi, Ehrampoush et al. 2016) | Iran | 5 | Mashhad | 1 | English | $PM_{2.5}$ | AQMS | NO | NO | 1,2,3 | Previous studies | Previous studies | NR | NR | NR | Yes | Yes | NO | NO | NO | Research | NO |
| (Kermani, Goudarzi et al. 2018) | Iran | 5 | Tehran, Mashhad, Tabriz, Isfahan, Shiraz, Ahwaz, Arak, Urmia | 1 | English | $PM_{10}$ | AQMS | NO | NO | PM10 (1,2,3,4,5), PM2.5 (1) | Previous studies | Previous studies | Previous studies | Previous studies | ST | Yes | Yes | Yes | NO | NO | Research | NO |

| Author/date | Country | WHO regions (AFR:1, AMR:2, SEAR: 3, EUR:4, EMR:5, WPR:6) | City | Type of publication (Article:1, Report:2, Thesis:3) | Language | Air Quality data | | | | Health endpoints (*Mortality* (All-cause: 1, CVD:2, Respiratory:3, YLG:10; LCM:11, Cardiopulmonary: 12, *Hospitalization* (CVD:4, Respiratory:5, COPD:6, CD:7, AMI:8, YLL:9, life expectancy: 14, Asthma:15, ARI:16, Acute bronchitis:17) | RR Sources | RR Sources (Categorized) | BI Sources | BI Sources (Categorized) | Type of exposure (Long-term (LT)/ Short-term (ST)) | Number of cases reported (Yes/No) | PAF reported (Yes/No) | CI reported (Yes/No) | Sensitivity/ Comparative analyses conducted (Yes/No) | Cut-off value used to estimate health effects reported (Yes/No) | Motivation or context of study | Population size reported (Yes/No) |
|---|---|---|---|---|---|---|---|---|---|---|---|---|---|---|---|---|---|---|---|---|---|---|
| | | | | | | Air Pollutants | AQMS network, Self-measured, Modeling, Satellite data | Data Coverage reported (Yes/No) | Air quality data processing and validation reported (Yes/No) | | | | | | | | | | | | | |
| (Goudarzi, Geravandi et al. 2015) | Iran | 5 | Ahvaz | 1 | English | $O_3$ | AQMS | NO | NO | 2,3 | NR | NR | NR | NR | NR | Yes | NO | Yes | NO | per 10 μg/m3 increase of concentration | Research | NO |
| (Omidi Khaniabadi, Daryanoosh et al. 2016) | Iran | 5 | Illam | 1 | English | $PM_{10}$ | AQMS | The daily 24-h averages were calculated from more than 75% of validated hourly data. | Yes | 3, 6 | WHO from epidemiological studies | WHO (studies, data, reports and values) | WHO values from epidemiological studies | WHO (studies, data, reports and values) | ST | Yes | Yes | Yes | NO | <50 mg/m3, (50-200 mg/m3), and >200 mg/m3 | Dust storm | NO |
| (Khaniabadi, Fanelli et al. 2017) | Iran | 5 | Khorramabad | 1 | English | $PM_{10}$ | AQMS | For the aggregation of hourly data to longer averaging periods (i.e., 24-h) a minimum data capture rate of 75% was imposed to calculate a valid aggregated value. | Yes | 4, 5 | WHO studies | WHO (studies, data, reports and values) | WHO studies | WHO (studies, data, reports and values) | ST | Yes | Yes | Yes | NO | <50 mg/m3, (50-200 mg/m3), and >200 mg/m3 | Dust storm | NO |
| (Maji, Dikshit et al. 2017) | India | 3 | Delhi | 1 | English | TSP, $NO_2$, $SO_2$ | AQMS and modeling | NO | NO | 1,2,3,6 | WHO default value | AirQ default values | WHO default value | AirQ default values | ST | Yes | NO | Yes | NO | NO | Research | NO |

| Author/date | Country | WHO regions (AFR:1, AMR:2, SEAR:3, EUR:4, EMR:5, WPR:6) | City | Type of publication (Article:1, Report:2, Thesis:3) | Language | Air Quality data | | | | Health endpoints (*Mortality* (All-cause: 1, CVD:2, Respiratory:3, YLG:10; LCM:11, Cardiopulmonary: 12, *Hospitalization* (CVD:4, Respiratory:5, COPD:6, CD:7, AMI:8, YLL:9, life expectancy: 14, Asthma:15, ARI:16, Acute bronchitis:17) | RR Sources | RR Sources (Categorized) | BI Sources | BI Sources (Categorized) | Type of exposure (Long-term (LT)/ Short-term (ST)) | Number of cases reported (Yes/No) | PAF reported (Yes/No) | CI reported (Yes/No) | Sensitivity/ Comparative analyses conducted (Yes/No) | Cut-off value used to estimate health effects reported (Yes/No) | Motivation or context of study | Population size reported (Yes/No) |
|---|---|---|---|---|---|---|---|---|---|---|---|---|---|---|---|---|---|---|---|---|---|---|
| | | | | | | Air Pollutants | AQMS network, Self-measured, Modeling, Satellite data | Data Coverage reported (Yes/No) | Air quality data processing and validation reported (Yes/No) | | | | | | | | | | | | | |
| (Maleki, Sorooshian et al. 2016) | Iran | 5 | Ahvaz | 1 | English | $PM_{10}$ | AQMS | NO | NO | 1,2,3,4,5,6 | NR | NR | NR | NR | NR | Yes | NO | NO | NO | NO | Dust storm | NO |
| (Yari, Goudarzi et al. 2016) | Iran | 5 | Ahvaz | 1 | English | $O_3$ | AQMS | NO | NO | 6 | WHO value | WHO (studies, data, reports and values) | WHO values | WHO (studies, data, reports and values) | ST | Yes | Yes | Yes | NO | NO | Research | NO |
| (Tahery, Geravandi et al. 2021) | Iran, Kyrgyz Republic | 5, 4 | Ahvaz, Bishkek | 1 | English | $PM_{10}$ | AQMS | NO | NO | 1,2,4,5 | Previous studies | Previous studies | Previous studies | Previous studies | ST | Yes | Yes | Yes | NO | NO | Dust storm | NO |
| (Kermani, Dowlati et al. 2018) | Iran | 5 | Tehran | 1 | English | $O_3$, $NO_2$, $SO_2$, $PM_{10}$, PM2.5 | AQMS | Y | NO | 1,2,3 | Previous studies | Previous studies | Previous studies | Previous studies | ST | Yes | Yes | Yes | NO | per 10 µg/m3 increase of concentration | Research | NO |
| (Abdolahnejad, Jafari et al. 2017) | Iran | 5 | Isfahan | 1 | English | $PM_{2.5}$, $PM_{10}$ | AQMS | NO | NO | 1,2,3,4,5 | WHO recommended value | WHO (studies, data, reports and values) | Local | Local | ST | Yes | Yes | NO | NO | NO | Research | NO |
| (Ghanbari Ghozikali, Borgini et al. 2016) | Iran | 5 | Tabriz | 1 | English | $PM_{10}$ | AQMS | NO | NO | 1,2,3,4,5 | WHO default value | AirQ default values | WHO default value | AirQ default values | ST | Yes | Yes | Yes | NO | per 10 µg/m3 increase of concentration | Research | NO |
| (Saki, Goudarzi et al. 2020) | Iran | 5 | Bushehr | 1 | English | $NO_2$ | AQMS | NO | NO | 6 | WHO default value | AirQ default values | WHO default value | AirQ default values | NR | Yes | Yes | Yes | NO | NO | Research | NO |
| (Vahedian, Garkaz et al. 2020) | Iran | 5 | Arak | 1 | English | $SO_2$, $PM_{10}$, $NO_2$, $O_3$ | AQMS | NO | NO | 2 | WHO databases | WHO (studies, data, reports and values) | WHO databases | WHO (studies, data, reports and values) | ST | Yes | Yes | Yes | NO | per 10 µg/m3 increase of concentration | Research | NO |
| (Zallaghi, Goudarzi et al. 2014) | Iran | 5 | Ahvaz | 1 | English | $PM_{10}$ | AQMS | NO | NO | 4,5 | NR | NR | NR | NR | ST | Yes | Yes | NO | NO | per 10 µg/m3 increase of concentration | Research | NO |
| (Nourmoradi, Omidi Khaniabadi et al. 2016) | Iran | 5 | Khorramabad | 1 | English | $PM_{10}$ | Self-measured | NO | NO | 1,2,3,4,5 | Previous studies | Previous studies | NR | NR | NR | Yes | Yes | NO | NO | per 10 µg/m3 increase of concentration | Research | NO |

| Author/date | Country | WHO regions (AFR:1, AMR:2, SEAR: 3, EUR:4, EMR:5, WPR:6) | City | Type of publication (Article:1, Report:2, Thesis:3) | Language | Air Quality data | | | | Health endpoints (*Mortality* (All-cause: 1, CVD:2, Respiratory:3, YLG:10; LCM:11, Cardiopulmonary: 12, *Hospitalization* (CVD:4, Respiratory:5, COPD:6, CD:7, AMI:8, YLL:9, life expectancy: 14, Asthma:15, ARI:16, Acute bronchitis:17) | RR Sources | RR Sources (Categorized) | BI Sources | BI Sources (Categorized) | Type of exposure (Long-term (LT)/ Short-term (ST)) | Number of cases reported (Yes/No) | PAF reported (Yes/No) | CI reported (Yes/No) | Sensitivity/ Comparative analyses conducted (Yes/No) | Cut-off value used to estimate health effects reported (Yes/No) | Motivation or context of study | Population size reported (Yes/No) |
|---|---|---|---|---|---|---|---|---|---|---|---|---|---|---|---|---|---|---|---|---|---|---|
| | | | | | | Air Pollutants | AQMS network, Self-measured, Modeling, Satellite data | Data Coverage reported (Yes/No) | Air quality data processing and validation reported (Yes/No) | | | | | | | | | | | | | |
| (Zallaghi, Geravandi et al. 2015) | Iran | 5 | Tabriz | 1 | English | $NO_2$ | AQMS | NO | NO | 2,6,8 | Previous studies | Previous studies | NR | NR | ST | Yes | Yes | Yes | NO | per 10 µg/m3 increase of concentration | Research | NO |
| (Mohammadi, Azhdarpoor et al. 2015) | Iran | 5 | Shiraz | 1 | English | $PM_{10}$ | AQMS | NO | NO | 1,2,3,4 | localized using local statistics | National | Local | Local | ST | Yes | Yes | Yes | NO | per 10 µg/m3 increase of concentration | Research | NO |
| (Shahsavani, Naddafi et al. 2012) | Iran | 5 | Ahvaz | 1 | English | $PM_{2.5}$, $PM_{10}$ | Self-measured | NO | NO | 1,2,3,4,5,16 | WHO default value | AirQ default values | WHO default value | AirQ default values | ST/LT | Yes | Yes | Yes | NO | NO | Dust storm | NO |
| (Tominz, Mazzoleni et al. 2005) | Italy | 4 | Trieste | 1 | Italian | $PM_{10}$ | AQMS | Yes | Yes | 1,2,3,4,5 | Relative risks from an Italian study MISA | National | Local | Local | ST/LT | Yes | Yes | Yes | Yes | 20ug/m3 target for PM10 | Orient decision-making | Yes |
| (Sówka, Pachurka et al. 2016) | Poland | 4 | Wroclaw (Al. Wiśniowa), Wroclaw (Na Grobli), Wyb. J. Conrada-Korzeniowskiegoul. Orzechowaul. Kosiby (badania własne-) ul. Kosiby (badania własne-) ul. Kosiby (badania własne-) | 1 | Polish | $PM_1$, PM2.5, PM10 | AQMS /Self-measured | NO | Yes | 1,2,3,4,5 | NR | NR | Local | Local | ST | Yes | Yes | Yes | NO | 10 and 40 mg/m3 targets for PM2,5 and PM10 respectively | Research | Yes |
| (KERMANI, AZARSHAB et al. 2017) | Iran | 5 | Boukan | 1 | Persian | $PM_{2.5}$ | AQMS | 321 days | NO | 1 | Previous studies | Previous studies | Previous studies | Previous studies | ST | Yes | Yes | NO | NO | NO | Dust storm | NO |
| (Kermani, Dowlati et al. 2018) | Iran | 5 | Tehran | 1 | English | $PM_{10}$ | AQMS | The ratio of valid data of warm seasons to the cold seasons should not be more than 2. Furthermore, to estima | Yes | 4,5,8 | Previous studies | Previous studies | Previous studies | Previous studies | NR | Yes | Yes | Yes | NO | per 10 µg/m3 increase of concentration | Research | NO |

| Author/date | Country | WHO regions (AFR:1, AMR:2, SEAR: 3, EUR:4, EMR:5, WPR:6) | City | Type of publication (Article:1, Report:2, Thesis:3) | Language | Air Quality data | | | | Health endpoints (*Mortality* (All-cause: 1, CVD:2, Respiratory:3, YLG:10; LCM:11, Cardiopulmonary: 12, *Hospitalization* (CVD:4, Respiratory:5, COPD:6, CD:7, AMI:8, YLL:9, life expectancy: 14, Asthma:15, ARI:16, Acute bronchitis:17) | RR Sources | RR Sources (Categorized) | BI Sources | BI Sources (Categorized) | Type of exposure (Long-term (LT)/ Short-term (ST)) | Number of cases reported (Yes/No) | PAF reported (Yes/No) | CI reported (Yes/No) | Sensitivity/ Comparative analyses conducted (Yes/No) | Cut-off value used to estimate health effects reported (Yes/No) | Motivation or context of study | Population size reported (Yes/No) |
|---|---|---|---|---|---|---|---|---|---|---|---|---|---|---|---|---|---|---|---|---|---|---|
| | | | | | | Air Pollutants | AQMS network, Self-measured, Modeling, Satellite data | Data Coverage reported (Yes/No) | Air quality data processing and validation reported (Yes/No) | | | | | | | | | | | | | |
| | | | | | | | | te the daily average, at least 50% of data should have adequate validity. | | | | | | | | | | | | | | |
| (Kermani, Dowlati et al. 2017) | Iran | 5 | Tehran | 1 | Persian | CO | AQMS | The ratio of warm seasons/cold seasons needed to be less than 2. Also, 50% of the data needed to have enough validity to estimate the daily mean. | Yes | 2 | Previous studies | Previous studies | Previous studies | Previous studies | ST | Yes | Yes | Yes | NO | NO | Research | NO |
| (Boldo, Medina et al. 2006) | European Countries | 4 | 23 European cities | 1 | English | $PM_{2.5}$ | AQMS | NO | NO | 1,11,12 | Previous studies | Previous studies | Previous studies | Previous studies | LT | Yes | Yes | NO | NO | NO | Effect of reduce PM2.5 concentration | NO |
| (Yorifuji, Yamamoto et al. 2005) | Japan | 6 | Tokyo | 1 | English | $PM_{2.5}$, TSP | AQMS | NO | NO | 1,11,12 | WHO meta-analysis | WHO (studies, data, reports and values) | NR | NR | ST/LT | Yes | Yes | Yes | NO | NO | Research | only more than 30 years old |
| (Tonne, Beevers et al. 2008) | UK | 4 | London | 1 | English | $NO_2$, $PM_{10}$ | AQMS and modeling | NO | NO | 10 | Previous studies | Previous studies | National | National | LT | Yes | Yes | Yes | To identify the sensitivity of our | NO | Changes in traffic flow | NO |

| Author/date | Country | WHO regions (AFR:1, AMR:2, SEAR:3, EUR:4, EMR:5, WPR:6) | City | Type of publication (Article:1, Report:2, Thesis:3) | Language | Air Quality data | | | | Health endpoints (*Mortality* (All-cause: 1, CVD:2, Respiratory:3, YLG:10; LCM:11, Cardiopulmonary: 12, *Hospitalization* (CVD:4, Respiratory:5, COPD:6, CD:7, AMI:8, YLL:9, life expectancy: 14, Asthma:15, ARI:16, Acute bronchitis:17) | RR Sources | RR Sources (Categorized) | BI Sources | BI Sources (Categorized) | Type of exposure (Long-term (LT)/ Short-term (ST)) | Number of cases reported (Yes/No) | PAF reported (Yes/No) | CI reported (Yes/No) | Sensitivity/ Comparative analyses conducted (Yes/No) | Cut-off value used to estimate health effects reported (Yes/No) | Motivation or context of study | Population size reported (Yes/No) |
|---|---|---|---|---|---|---|---|---|---|---|---|---|---|---|---|---|---|---|---|---|---|---|
| | | | | | | Air Pollutants | AQMS network, Self-measured, Modeling, Satellite data | Data Coverage reported (Yes/No) | Air quality data processing and validation reported (Yes/No) | | | | | | | | | | | | | |
| | | | | | | | | | | | | | | | | | | | results to the boundary of the CCZ wards, we conducted a third sensitivity analysis in which the CCZ wards area was redefined as wards with centroids within the IRR | | | |
| (Yang and Kao 2013) | Taiwan | 6 | 22 cities | 1 | English | TSP, $PM_{10}$, $PM_{2.5}$ | AQMS | NO | NO | 1,2, 3, 10, 11 | Previous studies | Previous studies | NR | NR | LT | Yes | Yes | Yes | NO | per 10 µg/m3 increase of concentration | Reducing PM2.5 annual levels | NO |
| (Mansouri, Sadeghi et al. 2018) | Iran | 5 | Sanandaj | 1 | Persian | $PM_{10}$ | AQMS | NO | NO | 2,4,5 | Previous studies | Previous studies | Previous studies | Previous studies | NR | Yes | Yes | Yes | NO | per 10 µg/m3 increase of concentration | Research | NO |
| (MOKHTARI, MIRI et al. 2015) | Iran | 5 | Yazd | 1 | Persian | $PM_{10}$, $PM_{2.5}$, $SO_2$ | AQMS | NO | NO | 1,2,3,4,5 | Previous studies | Previous studies | Previous studies | Previous studies | NR | Yes | Yes | NO | NO | NO | Research | NO |
| (Kermani, Dowlati et al. 2016) | Iran | 5 | Tehran | 1 | Persian | $O_3$ | AQMS | The ratio of valid data of warm seasons to the cold season | Yes | 1,2,3,6 | Previous studies | Previous studies | Previous studies | Previous studies | NR | Yes | Yes | Yes | NO | per 10 µg/m3 increase of concentration | Research | NO |

| Author/date | Country | WHO regions (AFR:1, AMR:2, SEAR: 3, EUR:4, EMR:5, WPR:6) | City | Type of publication (Article:1, Report:2, Thesis:3) | Language | Air Quality data | | | | Health endpoints (*Mortality* (All-cause: 1, CVD:2, Respiratory:3, YLG:10; LCM:11, Cardiopulmonary: 12, *Hospitalization* (CVD:4, Respiratory:5, COPD:6, CD:7, AMI:8, YLL:9, life expectancy: 14, Asthma:15, ARI:16, Acute bronchitis:17) | RR Sources | RR Sources (Categorized) | BI Sources | BI Sources (Categorized) | Type of exposure (Long-term (LT)/ Short-term (ST)) | Number of cases reported (Yes/No) | PAF reported (Yes/No) | CI reported (Yes/No) | Sensitivity/ Comparative analyses conducted (Yes/No) | Cut-off value used to estimate health effects reported (Yes/No) | Motivation or context of study | Population size reported (Yes/No) |
|---|---|---|---|---|---|---|---|---|---|---|---|---|---|---|---|---|---|---|---|---|---|---|
| | | | | | | Air Pollutants | AQMS network, Self-measured, Modeling, Satellite data | Data Coverage reported (Yes/No) | Air quality data processing and validation reported (Yes/No) | | | | | | | | | | | | | |
| | | | | | | | | s should not be more than 2. Furthermore, to estimate the daily average, at least 50% of data should have adequate validity. | | | | | | | | | | | | | | |
| (Dobaradaran, Geravandi et al. 2016) | Iran | 5 | Bushehr | 1 | Persian | $PM_{10}$ | AQMS | NO | NO | 3,4 | WHO epidemiology study | WHO (studies, data, reports and values) | WHO epidemiology study | WHO (studies, data, reports and values) | ST | Yes | Yes | Yes | NO | per 10 µg/m3 increase of concentration | Research | NO |
| (Vahidi, Fanaei et al. 2020) | Iran | 5 | Karaj | 1 | English | $PM_{10}$ | AQMS | NO | NO | 1,2,3,4,5 | NR | NR | NR | NR | LT | Yes | Yes | NO | NO | NO | Research | NO |
| (Kermani, Dowlati et al. 2017) | Iran | 5 | Tehran | 1 | Persian | $PM_{10}$ | AQMS | The ratio of valid data of warm seasons to the cold seasons should not be more than 2. Furthermore, to estimate the daily averag | Yes | 1,2,3,4,5 | Previous studies | Previous studies | Previous studies | Previous studies | ST | Yes | Yes | Yes | NO | per 10 µg/m3 increase of concentration | Research | NO |

| Author/date | Country | WHO regions (AFR:1, AMR:2, SEAR: 3, EUR:4, EMR:5, WPR:6) | City | Type of publication (Article:1, Report:2, Thesis:3) | Language | Air Quality data | | | | Health endpoints (*Mortality* (All-cause: 1, CVD:2, Respiratory:3, YLG:10; LCM:11, Cardiopulmonary: 12, *Hospitalization* (CVD:4, Respiratory:5, COPD:6, CD:7, AMI:8, YLL:9, life expectancy: 14, Asthma:15, ARI:16, Acute bronchitis:17) | RR Sources | RR Sources (Categorized) | BI Sources | BI Sources (Categorized) | Type of exposure (Long-term (LT)/ Short-term (ST)) | Number of cases reported (Yes/No) | PAF reported (Yes/No) | CI reported (Yes/No) | Sensitivity/ Comparative analyses conducted (Yes/No) | Cut-off value used to estimate health effects reported (Yes/No) | Motivation or context of study | Population size reported (Yes/No) |
|---|---|---|---|---|---|---|---|---|---|---|---|---|---|---|---|---|---|---|---|---|---|---|
| | | | | | | Air Pollutants | AQMS network, Self-measured, Modeling, Satellite data | Data Coverage reported (Yes/No) | Air quality data processing and validation reported (Yes/No) | | | | | | | | | | | | | |
| | | | | | | | | e, at least 50% of data should have adequate validity. | | | | | | | | | | | | | | |
| | Iran | 5 | Bushehr, Kermanshah, Ahvaz | 1 | English | $SO_2$ | AQMS | NO | NO | 2,3 | NR (use formula) | NR (use formula) | NR | NR | ST | Yes | Yes | Yes | NO | per 10 µg/m3 increase of concentration | Research | NO |
| (Nemati, Mahvi et al. 2016) | Iran | 5 | Mashhad | 1 | English | NO2 | AQMS | NO | NO | 2,5,6 | NR | NR | NR | NR | ST | Yes | NO | Yes | NO | NO | Research | NO |
| (Mohammadi, Faraji et al. 2019) | Iran | 5 | Urmia | 1 | English | $PM_{10}$ | AQMS | NO | NO | 1,2,3,4,5 | time-series studies | Previous studies | Local | Local | ST | Yes | Yes | Yes | NO | per 10 µg/m3 increase of concentration | Traffic and drying Lake Urmia and its effects on human health | NO |
| (Kermani, Asl et al. 2016) | Iran | 5 | Tehran, Mashhad, Tabriz, Isfahan, Arak | 1 | English | $O_3$, $NO_2$ | AQMS | NO | NO | 6 | epidemiological study and exposure-response functions | Previous studies | Previous studies | Previous studies | ST | Yes | Yes | Yes | NO | per 10 µg/m3 increase of concentration | Research | NO |
| (Goudarzi, Geravandi et al. 2015) | Iran | 5 | Ahvaz | 1 | English | $PM_{10}$ | AQMS | NO | NO | 1, 3 | WHO data | WHO (studies, data, reports and values) | WHO data | WHO (studies, data, reports and values) | ST | Yes | Yes | Yes | NO | per 10 µg/m3 increase of concentration | Research | NO |
| (Junk, Krein et al. 2009) | Germany | 4 | Trier | 1 | English | $PM_{10}$ | AQMS | NO | Missing values within the time series were replaced by means of different procedures. Gaps with up to two missing values were closed by linear | 2,3 | Previous studies | Previous studies | NR | NR | ST/LT | Yes | Yes | NO | NO | per 10 µg/m3 increase of concentration | Research | NO |

| Author/date | Country | WHO regions (AFR:1, AMR:2, SEAR:3, EUR:4, EMR:5, WPR:6) | City | Type of publication (Article:1, Report:2, Thesis:3) | Language | Air Quality data | | | | Health endpoints (*Mortality* (All-cause: 1, CVD:2, Respiratory:3, YLG:10; LCM:11, Cardiopulmonary: 12, *Hospitalization* (CVD:4, Respiratory:5, COPD:6, CD:7, AMI:8, YLL:9, life expectancy: 14, Asthma:15, ARI:16, Acute bronchitis:17) | RR Sources | RR Sources (Categorized) | BI Sources | BI Sources (Categorized) | Type of exposure (Long-term (LT)/ Short-term (ST)) | Number of cases reported (Yes/No) | PAF reported (Yes/No) | CI reported (Yes/No) | Sensitivity/ Comparative analyses conducted (Yes/No) | Cut-off value used to estimate health effects reported (Yes/No) | Motivation or context of study | Population size reported (Yes/No) |
|---|---|---|---|---|---|---|---|---|---|---|---|---|---|---|---|---|---|---|---|---|---|---|
| | | | | | | Air Pollutants | AQMS network, Self-measured, Modeling, Satellite data | Data Coverage reported (Yes/No) | Air quality data processing and validation reported (Yes/No) | | | | | | | | | | | | | |
| | | | | | | | | | interpolation considering the presence of precipitation as a secondary the gap was closed with data from a comparable station. This procedure was used too, condition. With fulfilled secondary condition, when more than two missing values occurred. | | | | | | | | | | | | | |
| (Fattore and Paiano 2008) | Italy | 4 | Mazzano, Rezzato | 1 | Italian | $O_3$, PM2.5, PM10 | Self-measured | Y | NO | 1,2,3,9 | WHO value | WHO (studies, data, reports and values) | Local | Local | ST/LT | Yes | Yes | Yes | NO | 10 µg/m3 cut-off chosen | Research | Yes |
| (Hosseini, Maleki et al. 2014) | Iran | 5 | Sanandaj | 1 | English | $PM_{2.5}$ | AQMS | NO | NO | 1,2,3,4,5 | WHO value | WHO (studies, data, reports and values) | WHO value | WHO (studies, data, reports and values) | ST | Yes | Yes | Yes | NO | per 10 µg/m3 increase of concentration | research | NO |
| (Mohammadi Rouzbahani, Mohammadi et al. 2017) | Iran | 5 | Ahvaz | 1 | English | $O_3$, $NO_2$, $SO_2$ | AQMS | NO | NO | 6 | NR | NR | NR | NR | ST | Yes | Yes | Yes | NO | NO | Research | NO |
| (Kermani, Aghaei et al. 2016) | Iran | 5 | Tehran | 1 | Persian | $NO_2$, $O_3$ | AQMS | The ratio of valid data of warm seasons to the | Yes | 1,2,3,6 | NR | NR | NR | NR | ST | Yes | Yes | Yes | NO | per 10 µg/m3 increase of concentration | Research | NO |

| Author/date | Country | WHO regions (AFR:1, AMR:2, SEAR:3, EUR:4, EMR:5, WPR:6) | City | Type of publication (Article:1, Report:2, Thesis:3) | Language | Air Quality data | | | | Health endpoints (*Mortality* (All-cause: 1, CVD:2, Respiratory:3, YLG:10; LCM:11, Cardiopulmonary: 12, *Hospitalization* (CVD:4, Respiratory:5, COPD:6, CD:7, AMI:8, YLL:9, life expectancy: 14, Asthma:15, ARI:16, Acute bronchitis:17) | RR Sources | RR Sources (Categorized) | BI Sources | BI Sources (Categorized) | Type of exposure (Long-term (LT)/ Short-term (ST)) | Number of cases reported (Yes/No) | PAF reported (Yes/No) | CI reported (Yes/No) | Sensitivity/ Comparative analyses conducted (Yes/No) | Cut-off value used to estimate health effects reported (Yes/No) | Motivation or context of study | Population size reported (Yes/No) |
|---|---|---|---|---|---|---|---|---|---|---|---|---|---|---|---|---|---|---|---|---|---|---|
| | | | | | | Air Pollutants | AQMS network, Self-measured, Modeling, Satellite data | Data Coverage reported (Yes/No) | Air quality data processing and validation reported (Yes/No) | | | | | | | | | | | | | |
| | | | | | | | | cold seasons should not be more than 2. Furthermore, to estimate the daily average, at least 50% of data should have adequate validity. | | | | | | | | | | | | | | |
| (Yaghmaeian, Ghobakhloo et al. 2018) | Iran | 5 | Semnan | 1 | Persian | $PM_{2.5}$ | AQMS | NO | NO | 1,2,3,4,5 | Previous studies | Previous studies | Previous studies | Previous studies | ST | Yes | Yes | Yes | NO | per 10 µg/m3 increase of concentration | Research | NO |
| (Geravandi, Goudarzi et al. 2016) | Iran | 5 | Bushehr | 1 | Persian | $SO_2$ | AQMS | NO | NO | 2,3 | WHO epidemiology study | WHO (studies, data, reports and values) | WHO epidemiology study | WHO (studies, data, reports and values) | ST | Yes | Yes | NO | NO | per 10 µg/m3 increase of concentration | Research | NO |
| (Naddafi, Hassanvand et al. 2012) | Iran | 5 | Tehran | 1 | English | $PM_{10}$, $SO_2$, $NO_2$, $O_3$ | AQMS | PM10: 7 station (2438 days). SO2: 10 station (3172 days). PM10: 13 station (4115 days). O3: 11 station (3530 days). | Yes | 1,2,3,4,5,6,8 | for PM10 were summary estimates derived from a quantitative meta-analysis of peer-reviewed studies focused on European investigations, while for SO2 the RR implemented in the software and proposed as summary estimate in the WHO Air | AirQ default values/ Previous studies | National and WHO values | AirQ default values/ Local | ST | Yes | Yes | Yes | NO | per 10 µg/m3 increase of concentration | Research | NO |

| Author/date | Country | WHO regions (AFR:1, AMR:2, SEAR: 3, EUR:4, EMR:5, WPR:6) | City | Type of publication (Article:1, Report:2, Thesis:3) | Language | Air Quality data | | | | Health endpoints (*Mortality* (All-cause: 1, CVD:2, Respiratory:3, YLG:10; LCM:11, Cardiopulmonary: 12, *Hospitalization* (CVD:4, Respiratory:5, COPD:6, CD:7, AMI:8, YLL:9, life expectancy: 14, Asthma:15, ARI:16, Acute bronchitis:17) | RR Sources | RR Sources (Categorized) | BI Sources | BI Sources (Categorized) | Type of exposure (Long-term (LT)/ Short-term (ST)) | Number of cases reported (Yes/No) | PAF reported (Yes/No) | CI reported (Yes/No) | Sensitivity/ Comparative analyses conducted (Yes/No) | Cut-off value used to estimate health effects reported (Yes/No) | Motivation or context of study | Population size reported (Yes/No) |
|---|---|---|---|---|---|---|---|---|---|---|---|---|---|---|---|---|---|---|---|---|---|---|
| | | | | | | Air Pollutants | AQMS network, Self-measured, Modeling, Satellite data | Data Coverage reported (Yes/No) | Air quality data processing and validation reported (Yes/No) | | | | | | | | | | | | | |
| | | | | | | | | | | | Quality Guidelines for Europe was used, for O3 and NO2 the RR values came directly from published studies on short-term effects within the Air Pollution and Health: a European Approach (APHEA) project | | | | | | | | | | | |
| (GERAVANDI, MOHAMMADI et al. 2015) | Iran | 5 | Ahvaz | 1 | Persian | $PM_{10}$ | AQMS | NO | NO | 1,2 | WHO value | WHO (studies, data, reports and values) | WHO values | WHO (studies, data, reports and values) | NR | Yes | NO | Yes | NO | per 10 µg/m3 increase of concentration | Research | NO |
| (Zallaghi, Shirmardi et al. 2014) | Iran | 5 | Kermanshah | 1 | English | $PM_{10}$ | Self-measured | NO | NO | 2,3,4,5 | NR | NR | NR | NR | ST | Yes | Yes | Yes | NO | per 10 µg/m3 increase of concentration | Research | NO |
| (Kamarehie, Ghaderpoori et al. 2017) | Iran | 5 | Boukan | 1 | English | $SO_2$, $NO_2$ | AQMS | To obtain one-hour average values from data with a smaller averaging time, at least 75% of valid data should be used. To obtain average | Yes | 1,2,3,6,8 | Previous studies | Previous studies | Previous studies | Previous studies | ST | Yes | Yes | Yes | NO | NO | Research | NO |

| Author/date | Country | WHO regions (AFR:1, AMR:2, SEAR: 3, EUR:4, EMR:5, WPR:6) | City | Type of publication (Article:1, Report:2, Thesis:3) | Language | Air Quality data | | | | Health endpoints (*Mortality* (All-cause: 1, CVD:2, Respiratory:3, YLG:10; LCM:11, Cardiopulmonary: 12, *Hospitalization* (CVD:4, Respiratory:5, COPD:6, CD:7, AMI:8, YLL:9, life expectancy: 14, Asthma:15, ARI:16, Acute bronchitis:17) | RR Sources | RR Sources (Categorized) | BI Sources | BI Sources (Categorized) | Type of exposure (Long-term (LT)/ Short-term (ST)) | Number of cases reported (Yes/No) | PAF reported (Yes/No) | CI reported (Yes/No) | Sensitivity/ Comparative analyses conducted (Yes/No) | Cut-off value used to estimate health effects reported (Yes/No) | Motivation or context of study | Population size reported (Yes/No) |
|---|---|---|---|---|---|---|---|---|---|---|---|---|---|---|---|---|---|---|---|---|---|---|
| | | | | | | Air Pollutants | AQMS network, Self-measured, Modeling, Satellite data | Data Coverage reported (Yes/No) | Air quality data processing and validation reported (Yes/No) | | | | | | | | | | | | | |
| | | | | | | | | values of 8-hour 'moving' from hourly measures, the number of hours where valid measures have been performed must be for at least 18 hours (75%). To obtain 24-hour average values from data, at least 50% of time should be available. To obtain seasonal and annual average values, at least 50% of | | | | | | | | | | | | | | |

| **Author/date** | **Country** | **WHO regions (AFR:1, AMR:2, SEAR: 3, EUR:4, EMR:5, WPR:6)** | **City** | **Type of publication (Article:1, Report:2, Thesis:3)** | **Language** | **Air Quality data** | | | | **Health endpoints (*Mortality* (All-cause: 1, CVD:2, Respiratory:3, YLG:10; LCM:11, Cardiopulmonary: 12, *Hospitalization* (CVD:4, Respiratory:5, COPD:6, CD:7, AMI:8, YLL:9, life expectancy: 14, Asthma:15, ARI:16, Acute bronchitis:17)** | **RR Sources** | **RR Sources (Categorized)** | **BI Sources** | **BI Sources (Categorized)** | **Type of exposure (Long-term (LT)/ Short-term (ST))** | **Number of cases reported (Yes/No)** | **PAF reported (Yes/No)** | **CI reported (Yes/No)** | **Sensitivity/ Comparative analyses conducted (Yes/No)** | **Cut-off value used to estimate health effects reported (Yes/No)** | **Motivation or context of study** | **Population size reported (Yes/No)** |
|---|---|---|---|---|---|---|---|---|---|---|---|---|---|---|---|---|---|---|---|---|---|---|
| | | | | | | **Air Pollutants** | **AQMS network, Self-measured, Modeling, Satellite data** | **Data Coverage reported (Yes/No)** | **Air quality data processing and validation reported (Yes/No)** | | | | | | | | | | | | | |
| | | | | | | | | valid data for the reported period should be used. | | | | | | | | | | | | | | |
| (Tabasideh, Vaisi et al. 2017) | Iran | 5 | Ilam | 1 | English | $PM_{10}$ | AQMS | NO | NO | 1,2,3,4,5 | epidemiological studies conducted by WHO | WHO (studies, data, reports and values) | epidemiological studies conducted by WHO | WHO (studies, data, reports and values) | ST | Yes | Yes | Yes | NO | per 10 µg/m3 increase of concentration | Research | NO |
| (Kohzadi, Amini et al. 2018) | Iran | 5 | Sanandaj | 1 | English | $PM_{10}$ | AQMS | NO | NO | 1,2,3,4,5 | NR | NR | NR | NR | ST | Yes | Yes | NO | NO | per 10 µg/m3 increase of concentration | Research | NO |
| (Geravandi, Goudarzi et al. 2016) | Iran | 5 | Ahvaz | 1 | English | $O_3$ | AQMS | NO | NO | 3,6 | WHO data | WHO (studies, data, reports and values) | NR | NR | ST/LT | Yes | Yes | NO | NO | NO | Research | NO |
| (Daryanoosh, Goudarzi et al. 2017) | Iran | 5 | Illam | 1 | English | $PM_{10}$ | AQMS | NO | NO | 1,2,3 | Previous studies | Previous studies | Previous studies | Previous studies | ST | Yes | Yes | Yes | NO | per 10 µg/m3 increase of concentration | Research | NO |
| (Motalleby, Mazaheri et al. 2015) | Iran | 5 | Kashan | 1 | Persian | $PM_{10}$, $NO_2$, $SO_2$, $O_3$ | AQMS | NO | NO | 1,2,3,4,5,6,8 | Previous studies | Previous studies | Previous studies | Previous studies | ST | Yes | Yes | Yes | NO | per 10 µg/m3 increase of concentration | Research | NO |
| (GERAVANDI, ZALAGHI et al. 2016) | Iran | 5 | Isfahan | 1 | Persian | $PM_{10}$ | Self-measured | NO | NO | 4,5 | WHO value | WHO (studies, data, reports and values) | WHO values | WHO (studies, data, reports and values) | NR | Yes | Yes | Yes | NO | per 10 µg/m3 increase of concentration | Research | NO |
| (KERMANI, GHOLAMI et al. 2016) | Iran | 5 | Tabriz, Mashhad, Shiraz, Isfahan, Ahvaz, Arak, Urmia | 1 | Persian | $PM_{10}$ | AQMS | The ratio of valid data of warm seasons to the cold seasons should not be more | Yes | 1,2,3,4,5 | Previous studies | Previous studies | Previous studies | Previous studies | NR | Yes | Yes | Yes | NO | per 10 µg/m3 increase of concentration | Research | NO |

| Author/date | Country | WHO regions (AFR:1, AMR:2, SEAR:3, EUR:4, EMR:5, WPR:6) | City | Type of publication (Article:1, Report:2, Thesis:3) | Language | Air Quality data | | | | Health endpoints (*Mortality* (All-cause: 1, CVD:2, Respiratory:3, YLG:10; LCM:11, Cardiopulmonary: 12, *Hospitalization* (CVD:4, Respiratory:5, COPD:6, CD:7, AMI:8, YLL:9, life expectancy: 14, Asthma:15, ARI:16, Acute bronchitis:17) | RR Sources | RR Sources (Categorized) | BI Sources | BI Sources (Categorized) | Type of exposure (Long-term (LT)/ Short-term (ST)) | Number of cases reported (Yes/No) | PAF reported (Yes/No) | CI reported (Yes/No) | Sensitivity/ Comparative analyses conducted (Yes/No) | Cut-off value used to estimate health effects reported (Yes/No) | Motivation or context of study | Population size reported (Yes/No) |
|---|---|---|---|---|---|---|---|---|---|---|---|---|---|---|---|---|---|---|---|---|---|---|
| | | | | | | Air Pollutants | AQMS network, Self-measured, Modeling, Satellite data | Data Coverage reported (Yes/No) | Air quality data processing and validation reported (Yes/No) | | | | | | | | | | | | | |
| | | | | | | | | than 2. Furthermore, to estimate the daily average, at least 50% of data should have adequate validity. | | | | | | | | | | | | | | |
| (Fallahizadeh 2018) | Iran | 5 | Gachsaran | 1 | Persian | $PM_{10}$ | AQMS | NO | NO | 1,2,3,4,5 | Previous studies | Previous studies | Previous studies | Previous studies | NR | Yes | Yes | Yes | NO | NO | Research | NO |
| (Goudarzi, Mohammadi et al. 2012) | Iran | 5 | Ahvaz | 1 | English | $NO_2$ | AQMS | NO | NO | 2,6,8 | WHO default value | AirQ default values | WHO default value | AirQ default values | ST | Yes | Yes | Yes | NO | per 10 µg/m3 increase of concentration | Research | NO |
| (Goudarzi, Zallaghi et al. 2013) | Iran | 5 | Ahvaz | 1 | English | $O_3$ | AQMS | NO | NO | 1,2,3,6 | NR | NR | NR | NR | NR | Yes | Yes | Yes | NO | per 10 µg/m3 increase of concentration | Research | NO |
| (Goudarzi, Geravandi et al. 2014) | Iran | 5 | Ahvaz | 1 | English | $SO_2$ | AQMS | NO | NO | 2,8 | WHO data | WHO (studies, data, reports and values) | WHO data | WHO (studies, data, reports and values) | ST | Yes | Yes | Yes | NO | per 10 µg/m3 increase of concentration | Research | NO |
| (Geravandia, Goudarzic et al. 2015) | Iran | 5 | Ahvaz | 1 | English | $PM_{10}$ | AQMS | NO | NO | 2,5 | WHO data | WHO (studies, data, reports and values) | WHO data | WHO (studies, data, reports and values) | ST | Yes | Yes | Yes | NO | per 10 µg/m3 increase of concentration | Research | NO |
| (Behrouzi Rad, Mohammadi et al. 2017) | Iran | 5 | Ahvaz, Bushehr, Kermanshah | 1 | English | $PM_{10}$ | AQMS | NO | NO | 3,5 | NR | NR | NR | NR | ST | Yes | Yes | Yes | NO | NO | Dust storm | NO |
| (Jafari, Nemati et al. 2017) | Iran | 5 | Isfahan | 1 | Persian | $PM_{2.5}$, $PM_{10}$, $O_3$, $SO_2$, $NO_2$ | AQMS | NO | NO | 1,2,3 | WHO data | WHO (studies, data, reports and values) | Local | Local | NR | Yes | Yes | Yes | NO | per 10 µg/m3 increase of concentration | Research | NO |

| Author/date | Country | WHO regions (AFR:1, AMR:2, SEAR:3, EUR:4, EMR:5, WPR:6) | City | Type of publication (Article:1, Report:2, Thesis:3) | Language | Air Quality data | | | | Health endpoints (*Mortality* (All-cause: 1, CVD:2, Respiratory:3, YLG:10; LCM:11, Cardiopulmonary: 12, *Hospitalization* (CVD:4, Respiratory:5, COPD:6, CD:7, AMI:8, YLL:9, life expectancy: 14, Asthma:15, ARI:16, Acute bronchitis:17) | RR Sources | RR Sources (Categorized) | BI Sources | BI Sources (Categorized) | Type of exposure (Long-term (LT)/ Short-term (ST)) | Number of cases reported (Yes/No) | PAF reported (Yes/No) | CI reported (Yes/No) | Sensitivity/ Comparative analyses conducted (Yes/No) | Cut-off value used to estimate health effects reported (Yes/No) | Motivation or context of study | Population size reported (Yes/No) |
|---|---|---|---|---|---|---|---|---|---|---|---|---|---|---|---|---|---|---|---|---|---|---|
| | | | | | | Air Pollutants | AQMS network, Self-measured, Modeling, Satellite data | Data Coverage reported (Yes/No) | Air quality data processing and validation reported (Yes/No) | | | | | | | | | | | | | |
| (Leili, Bahrami Asl et al. 2017) | Iran | 5 | Hamedan | 1 | Persian | $NO_2$, $SO_2$ | AQMS | NO | NO | 1,2,3,6,8 | Previous studies | Previous studies | Previous studies | Previous studies | ST | Yes | Yes | Yes | NO | per 10 µg/m3 increase of concentration | Research | NO |
| (Ghanizadeh, Khoshniyat et al. 2018) | Iran | 5 | Sanandaj | 1 | English | $PM_{10}$ | AQMS | NO | NO | 1,2,3,4,5 | Previous studies | Previous studies | Previous studies | Previous studies | ST | Yes | Yes | Yes | NO | per 10 µg/m3 increase of concentration | Dust storm | NO |
| (Goudarzi, Geravandi et al. 2015) | Iran | 5 | Ahvaz | 1 | Persian | $PM_{10}$ | AQMS | NO | NO | 6 | WHO data | WHO (studies, data, reports and values) | WHO data | WHO (studies, data, reports and values) | ST | Yes | Yes | Yes | NO | per 10 µg/m3 increase of concentration | Research | NO |
| (Goudarzi, Mohammadi et al. 2013) | Iran | 5 | Ahvaz | 1 | Persian | $NO_2$ | AQMS | NO | NO | 2,6,8 | NR | NR | NR | NR | NR | Yes | NO | Yes | NO | per 10 µg/m3 increase of concentration | Research | NO |
| (Goudarzi, Geravandi et al. 2014) | Iran | 5 | Ahvaz | 1 | English | $PM_{10}$ | AQMS | NO | NO | 2,3,4,5 | WHO data | WHO (studies, data, reports and values) | WHO data | WHO (studies, data, reports and values) | ST | Yes | Yes | Yes | NO | per 10 µg/m3 increase of concentration | Research | NO |
| (Goudarzi, Geravandi et al. 2014) | Iran | 5 | Ahvaz | 1 | English | CO | AQMS | NO | NO | 2 | WHO data | WHO (studies, data, reports and values) | WHO data | WHO (studies, data, reports and values) | ST | Yes | Yes | Yes | NO | per 10 µg/m3 increase of concentration | Research | NO |
| (Lak, Fazlzadeh et al. 2016) | Iran | 5 | Tabriz | 1 | Persian | $PM_{2.5}$ | AQMS | NO | NO | 1,2,3,4,5 | Default value | AirQ default values | Default value | AirQ default values | NR | Yes | Yes | Yes | NO | per 10 µg/m3 increase of concentration | Research | NO |
| (Kermani, Dowlati et al. 2017) | Iran | 5 | Tehran | 1 | Persian | $PM_{2.5}$, $PM_{10}$ | AQMS | The ratio of valid data of warm seasons to the cold seasons should not be more than 2. Furthermore, | Yes | 1,2,3,4,5 | Previous studies | Previous studies | Previous studies | Previous studies | NR | Yes | Yes | Yes | NO | per 10 µg/m3 increase of concentration | Research | NO |

| Author/date | Country | WHO regions (AFR:1, AMR:2, SEAR: 3, EUR:4, EMR:5, WPR:6) | City | Type of publication (Article:1, Report:2, Thesis:3) | Language | Air Quality data | | | | Health endpoints (*Mortality* (All-cause: 1, CVD:2, Respiratory:3, YLG:10; LCM:11, Cardiopulmonary: 12, *Hospitalization* (CVD:4, Respiratory:5, COPD:6, CD:7, AMI:8, YLL:9, life expectancy: 14, Asthma:15, ARI:16, Acute bronchitis:17) | RR Sources | RR Sources (Categorized) | BI Sources | BI Sources (Categorized) | Type of exposure (Long-term (LT)/ Short-term (ST)) | Number of cases reported (Yes/No) | PAF reported (Yes/No) | CI reported (Yes/No) | Sensitivity/ Comparative analyses conducted (Yes/No) | Cut-off value used to estimate health effects reported (Yes/No) | Motivation or context of study | Population size reported (Yes/No) |
|---|---|---|---|---|---|---|---|---|---|---|---|---|---|---|---|---|---|---|---|---|---|---|
| | | | | | | Air Pollutants | AQMS network, Self-measured, Modeling, Satellite data | Data Coverage reported (Yes/No) | Air quality data processing and validation reported (Yes/No) | | | | | | | | | | | | | |
| | | | | | | | | to estimate the daily average, at least 50% of data should have adequate validity. | | | | | | | | | | | | | | |
| (Bonyadi, Ehrampoush et al. 2016) | Iran | 5 | Mashhad | 1 | Persian | $PM_{2.5}$ | AQMS | The ratio between the numbers of valid data for the two seasons (warm and cold) should not be more than 2, at least 75% of valid data must exist in order to achieve the average one-hour values from data with a shorter averag | Yes | 1,2,3 | Previous studies | Previous studies | Previous studies | Previous studies | ST | Yes | Yes | Yes | NO | per 10 µg/m3 increase of concentration | Research | NO |

| Author/date | Country | WHO regions (AFR:1, AMR:2, SEAR:3, EUR:4, EMR:5, WPR:6) | City | Type of publication (Article:1, Report:2, Thesis:3) | Language | Air Quality data | | | | Health endpoints (*Mortality* (All-cause: 1, CVD:2, Respiratory:3, YLG:10; LCM:11, Cardiopulmonary: 12, *Hospitalization* (CVD:4, Respiratory:5, COPD:6, CD:7, AMI:8, YLL:9, life expectancy: 14, Asthma:15, ARI:16, Acute bronchitis:17) | RR Sources | RR Sources (Categorized) | BI Sources | BI Sources (Categorized) | Type of exposure (Long-term (LT)/ Short-term (ST)) | Number of cases reported (Yes/No) | PAF reported (Yes/No) | CI reported (Yes/No) | Sensitivity/ Comparative analyses conducted (Yes/No) | Cut-off value used to estimate health effects reported (Yes/No) | Motivation or context of study | Population size reported (Yes/No) |
|---|---|---|---|---|---|---|---|---|---|---|---|---|---|---|---|---|---|---|---|---|---|---|
| | | | | | | Air Pollutants | AQMS network, Self-measured, Modeling, Satellite data | Data Coverage reported (Yes/No) | Air quality data processing and validation reported (Yes/No) | | | | | | | | | | | | | |
| | | | | | | | | e time, at least 75% of one-hour data (18 hours) should exist and be valid to access the values of the eight-hour moving average form one-hour data. | | | | | | | | | | | | | | |
| (Kermani, Dowlati et al. 2017) | Iran | 5 | Tehran | 1 | Persian | $O_3$ | AQMS | The ratio between the number of valid data for the 2 seasons (warm and cold season) should not be more than 2. Also, in order to achieve the median 24-hour | Yes | 1,2,3,6 | Previous studies | Previous studies | Previous studies | Previous studies | NR | Yes | Yes | NO | NO | NO | Research | NO |

| Author/date | Country | WHO regions (AFR:1, AMR:2, SEAR:3, EUR:4, EMR:5, WPR:6) | City | Type of publication (Article:1, Report:2, Thesis:3) | Language | Air Quality data | | | | Health endpoints (*Mortality* (All-cause: 1, CVD:2, Respiratory:3, YLG:10; LCM:11, Cardiopulmonary: 12, *Hospitalization* (CVD:4, Respiratory:5, COPD:6, CD:7, AMI:8, YLL:9, life expectancy: 14, Asthma:15, ARI:16, Acute bronchitis:17) | RR Sources | RR Sources (Categorized) | BI Sources | BI Sources (Categorized) | Type of exposure (Long-term (LT)/ Short-term (ST)) | Number of cases reported (Yes/No) | PAF reported (Yes/No) | CI reported (Yes/No) | Sensitivity/ Comparative analyses conducted (Yes/No) | Cut-off value used to estimate health effects reported (Yes/No) | Motivation or context of study | Population size reported (Yes/No) |
|---|---|---|---|---|---|---|---|---|---|---|---|---|---|---|---|---|---|---|---|---|---|---|
| | | | | | | Air Pollutants | AQMS network, Self-measured, Modeling, Satellite data | Data Coverage reported (Yes/No) | Air quality data processing and validation reported (Yes/No) | | | | | | | | | | | | | |
| | | | | | | | | values, at least 50% data must exist with sufficient validity. | | | | | | | | | | | | | | |
| (Ghozikali, Borgini et al. 2015) | Iran | 5 | Tabriz | 1 | Persian | $O_3$ | AQMS | NO | NO | 1,2,3,6 | NR | NR | NR | NR | NR | Yes | Yes | Yes | NO | per 10 µg/m3 increase of concentration | Research | NO |
| (Khorsandi, Karimzade et al. 2016) | Iran | 5 | Urmia | 1 | Persian | $NO_2$ | AQMS | Y | NO | 1,2,6 | NR | NR | NR | NR | NR | Yes | Yes | Yes | NO | per 10 µg/m3 increase of concentration | Research | NO |
| (Khorsandi, Karimzade et al. 2016) | Iran | 5 | Urmia | 1 | Persian | $PM_{10}$, $SO_2$ | AQMS | The ratio between the numbers of valid data for the two seasons (warm and cold) should not be more than 2, at least 75% of valid data must exist in order to achieve the average one-hour | Yes | 1,2,3,6,8 | WHO data | WHO (studies, data, reports and values) | WHO data | WHO (studies, data, reports and values) | ST | Yes | Yes | Yes | NO | per 10 µg/m3 increase of concentration | Research | NO |

| Author/date | Country | WHO regions (AFR:1, AMR:2, SEAR:3, EUR:4, EMR:5, WPR:6) | City | Type of publication (Article:1, Report:2, Thesis:3) | Language | Air Quality data | | | | Health endpoints (*Mortality* (All-cause: 1, CVD:2, Respiratory:3, YLG:10; LCM:11, Cardiopulmonary: 12, *Hospitalization* (CVD:4, Respiratory:5, COPD:6, CD:7, AMI:8, YLL:9, life expectancy: 14, Asthma:15, ARI:16, Acute bronchitis:17) | RR Sources | RR Sources (Categorized) | BI Sources | BI Sources (Categorized) | Type of exposure (Long-term (LT)/ Short-term (ST)) | Number of cases reported (Yes/No) | PAF reported (Yes/No) | CI reported (Yes/No) | Sensitivity/ Comparative analyses conducted (Yes/No) | Cut-off value used to estimate health effects reported (Yes/No) | Motivation or context of study | Population size reported (Yes/No) |
|---|---|---|---|---|---|---|---|---|---|---|---|---|---|---|---|---|---|---|---|---|---|---|
| | | | | | | Air Pollutants | AQMS network, Self-measured, Modeling, Satellite data | Data Coverage reported (Yes/No) | Air quality data processing and validation reported (Yes/No) | | | | | | | | | | | | | |
| | | | | | | | | values from data with a shorter average time, at least 75% of one-hour data (18 hours) should exist and be valid to access the values of the eight-hour moving average form one-hour data. | | | | | | | | | | | | | | |
| (Kermani, Dowlati et al. 2017) | Iran | 5 | Tehran | 1 | English | $NO_2$ | AQMS | The ratio between the numbers of valid data for the two seasons (warm and cold) should not be more than 2, at least 75% of | Yes | 1,2,6 | Previous studies | Previous studies | WHO default values | AirQ default values | LT | Yes | Yes | Yes | NO | per 10 µg/m3 increase of concentration | Research | NO |

| Author/date | Country | WHO regions (AFR:1, AMR:2, SEAR: 3, EUR:4, EMR:5, WPR:6) | City | Type of publication (Article:1, Report:2, Thesis:3) | Language | Air Quality data | | | | Health endpoints (*Mortality* (All-cause: 1, CVD:2, Respiratory:3, YLG:10; LCM:11, Cardiopulmonary: 12, *Hospitalization* (CVD:4, Respiratory:5, COPD:6, CD:7, AMI:8, YLL:9, life expectancy: 14, Asthma:15, ARI:16, Acute bronchitis:17) | RR Sources | RR Sources (Categorized) | BI Sources | BI Sources (Categorized) | Type of exposure (Long-term (LT)/ Short-term (ST)) | Number of cases reported (Yes/No) | PAF reported (Yes/No) | CI reported (Yes/No) | Sensitivity/ Comparative analyses conducted (Yes/No) | Cut-off value used to estimate health effects reported (Yes/No) | Motivation or context of study | Population size reported (Yes/No) |
|---|---|---|---|---|---|---|---|---|---|---|---|---|---|---|---|---|---|---|---|---|---|---|
| | | | | | | Air Pollutants | AQMS network, Self-measured, Modeling, Satellite data | Data Coverage reported (Yes/No) | Air quality data processing and validation reported (Yes/No) | | | | | | | | | | | | | |
| | | | | | | | | valid data must exist in order to achieve the average one-hour values from data with a shorter average time, at least 75% of one-hour data (18 hours) should exist and be valid to access the values of the eight-hour moving average form one-hour data. | | | | | | | | | | | | | | |
| (Geravandi, Neisi et al. 2015) | Iran | 5 | Ahvaz | 1 | Persian | $O_3$ | AQMS | NO | NO | 2,3 | WHO data | WHO (studies, data, reports and values) | WHO data | WHO (studies, data, reports and values) | NR | Yes | Yes | Yes | NO | per 10 µg/m3 increase of concentration | Research | NO |
| (Geravandi, Goudarzi et al. 2016) | Iran | 5 | Ahvaz | 1 | Persian | $PM_{10}$ | AQMS | NO | NO | 2,5 | WHO data | WHO (studies, data, reports and values) | WHO data | WHO (studies, data, reports and values) | ST | Yes | Yes | NO | NO | per 10 µg/m3 increase of concentration | Research | NO |

| Author/date | Country | WHO regions (AFR:1, AMR:2, SEAR: 3, EUR:4, EMR:5, WPR:6) | City | Type of publication (Article:1, Report:2, Thesis:3) | Language | Air Quality data | | | | Health endpoints (*Mortality* (All-cause: 1, CVD:2, Respiratory:3, YLG:10; LCM:11, Cardiopulmonary: 12, *Hospitalization* (CVD:4, Respiratory:5, COPD:6, CD:7, AMI:8, YLL:9, life expectancy: 14, Asthma:15, ARI:16, Acute bronchitis:17) | RR Sources | RR Sources (Categorized) | BI Sources | BI Sources (Categorized) | Type of exposure (Long-term (LT)/ Short-term (ST)) | Number of cases reported (Yes/No) | PAF reported (Yes/No) | CI reported (Yes/No) | Sensitivity/ Comparative analyses conducted (Yes/No) | Cut-off value used to estimate health effects reported (Yes/No) | Motivation or context of study | Population size reported (Yes/No) |
|---|---|---|---|---|---|---|---|---|---|---|---|---|---|---|---|---|---|---|---|---|---|---|
| | | | | | | Air Pollutants | AQMS network, Self-measured, Modeling, Satellite data | Data Coverage reported (Yes/No) | Air quality data processing and validation reported (Yes/No) | | | | | | | | | | | | | |
| (Omidi, Goudarzi et al. 2016) | Iran | 5 | Kermanshah | 1 | English | $NO_2$ | AQMS | NO | NO | 2,4,6 | WHO data | WHO (studies, data, reports and values) | WHO data | WHO (studies, data, reports and values) | ST | Yes | Yes | Yes | NO | per 10 µg/m3 increase of concentration | Research | NO |
| (KERMANI, AGHAEI et al. 2016) | Iran | 5 | Mashhad, Shiraz, Isfahan, Tabriz, Ahvaz, Urmia, Tehran, Arak | 1 | Persian | CO, $PM_{2.5}$ | AQMS | NO | NO | 1,2 | Previous studies | Previous studies | Previous studies | Previous studies | NR | Yes | Yes | Yes | NO | per 10 µg/m3 increase of concentration | Research | NO |
| (Nourmoradi, Goudarzi et al. 2015) | Iran | 5 | Khorramabad | 1 | English | $PM_{10}$ | Self-measured | NO | NO | 1,2,3,5,4 | WHO data | WHO (studies, data, reports and values) | WHO data | WHO (studies, data, reports and values) | ST | Yes | Yes | Yes | NO | per 10 µg/m3 increase of concentration | Research | NO |
| (Mokhtari, Jafari et al. 2017) | Iran | 5 | Isfahan | 1 | Persian | $PM_{2.5}$ | AQMS | NO | NO | 1 | Previous studies | Previous studies | Local | Local | ST | Yes | Yes | Yes | NO | per 10 µg/m3 increase of concentration | Research | NO |
| (Daryanoosh, Goudarzi et al. 2017) | Iran | 5 | Illam | 1 | English | $PM_{10}$ | AQMS | NO | NO | 4,5 | WHO data | WHO (studies, data, reports and values) | WHO data | WHO (studies, data, reports and values) | ST | Yes | Yes | Yes | NO | per 10 µg/m3 increase of concentration | Research | NO |
| (Kermani, Dowlati et al. 2016) | Iran | 5 | Tehran | 1 | Persian | CO | AQMS | The ratio between the number of valid data for the 2 seasons (warm and cold seasons) should not be more than 2. Also, in order to achieve the median 24-hour | Yes | 2 | NR | NR | NR | NR | ST | Yes | Yes | Yes | NO | per 10 µg/m3 increase of concentration | Research | NO |

| **Author/date** | **Country** | **WHO regions (AFR:1, AMR:2, SEAR: 3, EUR:4, EMR:5, WPR:6)** | **City** | **Type of publication (Article:1, Report:2, Thesis:3)** | **Language** | **Air Quality data** | | | | **Health endpoints (*Mortality* (All-cause: 1, CVD:2, Respiratory:3, YLG:10; LCM:11, Cardiopulmonary: 12, *Hospitalization* (CVD:4, Respiratory:5, COPD:6, CD:7, AMI:8, YLL:9, life expectancy: 14, Asthma:15, ARI:16, Acute bronchitis:17)** | **RR Sources** | **RR Sources (Categorized)** | **BI Sources** | **BI Sources (Categorized)** | **Type of exposure (Long-term (LT)/ Short-term (ST))** | **Number of cases reported (Yes/No)** | **PAF reported (Yes/No)** | **CI reported (Yes/No)** | **Sensitivity/ Comparative analyses conducted (Yes/No)** | **Cut-off value used to estimate health effects reported (Yes/No)** | **Motivation or context of study** | **Population size reported (Yes/No)** |
|---|---|---|---|---|---|---|---|---|---|---|---|---|---|---|---|---|---|---|---|---|---|---|
| | | | | | | **Air Pollutants** | **AQMS network, Self-measured, Modeling, Satellite data** | **Data Coverage reported (Yes/No)** | **Air quality data processing and validation reported (Yes/No)** | | | | | | | | | | | | | |
| | | | | | | | | values, at least 50% data must exist with sufficient validity. | | | | | | | | | | | | | | |
| (Moustris, Ntourou et al. 2017) | Greece | 4 | Lykovrissi, Maroussi, Piraeus (port), Agia Paraskevi, Thrakomakedones, Aristotelous (Athens city center) | 1 | English | $PM_{10}$ | AQMS (city center, traffic, suburban, and background stations) | NO | NO | 5 | NR | NR | NR | NR | NR | Yes | NO | Yes | NO | per 10 µg/m3 increase of concentration | Research | NO |
| (Zallaghi, Goudarzi et al. 2014) | Iran | 5 | Kermanshah | 1 | English | $NO_2$ | AQMS | NO | NO | 2,4,6,8 | NR | NR | NR | NR | ST | Yes | Yes | Yes | NO | per 10 µg/m3 increase of concentration | Research | NO |
| (Geravandi 2015) | Iran | 5 | Kermanshah | 1 | Persian | $SO_2$ | AQMS | NO | NO | 2,3 | WHO data | WHO (studies, data, reports and values) | WHO data | WHO (studies, data, reports and values) | ST | Yes | Yes | NO | NO | per 10 µg/m3 increase of concentration | Research | NO |
| (Omidi, Goudarzi et al. 2016) | Iran | 5 | Kermanshah | 1 | English | $NO_2$ | AQMS | NO | NO | 2,6,8 | WHO data | WHO (studies, data, reports and values) | WHO data | WHO (studies, data, reports and values) | ST | Yes | Yes | Yes | NO | per 10 µg/m3 increase of concentration | Research | NO |
| (Kamarehie, Ghaderpoori et al. 2017) | Iran | 5 | Boukan | 1 | English | $PM_{2.5}$, $PM_{10}$ | AQMS | NO | NO | 1,2,3,4,5 | Previous studies | Previous studies | Previous studies | Previous studies | ST | Yes | Yes | Yes | NO | per 10 µg/m3 increase of concentration | Research | NO |
| (Momtazan, Geravandi et al. 2018) | Iran | 5 | Abadan and Khorramshahr | 1 | English | $PM_{10}$ | AQMS | NO | NO | 2,3 | Default value | AirQ default values | Default values | AirQ default values | ST | Yes | Yes | Yes | NO | per 10 µg/m3 increase of concentration | Dust storm | NO |
| (Kermani, Bahrami Asl et al. 2015) | Iran | 5 | Mashhad, Tabriz, Shiraz, Isfahan, Arak | 1 | Persian | $O_3$ | AQMS | The ratio between the numbers of valid data | Yes | 1,2,3 | Previous studies | Previous studies | Previous studies | Previous studies | ST/LT | Yes | Yes | Yes | NO | per 10 µg/m3 increase of concentration | Research | NO |

| Author/date | Country | WHO regions (AFR:1, AMR:2, SEAR: 3, EUR:4, EMR:5, WPR:6) | City | Type of publication (Article:1, Report:2, Thesis:3) | Language | Air Quality data | | | | Health endpoints (*Mortality* (All-cause: 1, CVD:2, Respiratory:3, YLG:10; LCM:11, Cardiopulmonary: 12, *Hospitalization* (CVD:4, Respiratory:5, COPD:6, CD:7, AMI:8, YLL:9, life expectancy: 14, Asthma:15, ARI:16, Acute bronchitis:17) | RR Sources | RR Sources (Categorized) | BI Sources | BI Sources (Categorized) | Type of exposure (Long-term (LT)/ Short-term (ST)) | Number of cases reported (Yes/No) | PAF reported (Yes/No) | CI reported (Yes/No) | Sensitivity/ Comparative analyses conducted (Yes/No) | Cut-off value used to estimate health effects reported (Yes/No) | Motivation or context of study | Population size reported (Yes/No) |
|---|---|---|---|---|---|---|---|---|---|---|---|---|---|---|---|---|---|---|---|---|---|---|
| | | | | | | Air Pollutants | AQMS network, Self-measured, Modeling, Satellite data | Data Coverage reported (Yes/No) | Air quality data processing and validation reported (Yes/No) | | | | | | | | | | | | | |
| | | | | | | | | for the two seasons (warm and cold) should not be more than 2, at least 75% of valid data must exist in order to achieve the average one-hour values from data with a shorter average time, at least 75% of one-hour data (18 hours) should exist and be valid to access the values of the eight-hour moving averag | | | | | | | | | | | | | | |

| Author/date | Country | WHO regions (AFR:1, AMR:2, SEAR: 3, EUR:4, EMR:5, WPR:6) | City | Type of publication (Article:1, Report:2, Thesis:3) | Language | Air Quality data | | | | Health endpoints (*Mortality* (All-cause: 1, CVD:2, Respiratory:3, YLG:10; LCM:11, Cardiopulmonary: 12, *Hospitalization* (CVD:4, Respiratory:5, COPD:6, CD:7, AMI:8, YLL:9, life expectancy: 14, Asthma:15, ARI:16, Acute bronchitis:17) | RR Sources | RR Sources (Categorized) | BI Sources | BI Sources (Categorized) | Type of exposure (Long-term (LT)/ Short-term (ST)) | Number of cases reported (Yes/No) | PAF reported (Yes/No) | CI reported (Yes/No) | Sensitivity/ Comparative analyses conducted (Yes/No) | Cut-off value used to estimate health effects reported (Yes/No) | Motivation or context of study | Population size reported (Yes/No) |
|---|---|---|---|---|---|---|---|---|---|---|---|---|---|---|---|---|---|---|---|---|---|---|---|
| | | | | | | Air Pollutants | AQMS network, Self-measured, Modeling, Satellite data | Data Coverage reported (Yes/No) | Air quality data processing and validation reported (Yes/No) | | | | | | | | | | | | | | |
| | | | | | | | | e form one-hour data. | | | | | | | | | | | | | | | |
| (GOUDARZI, GERAVANDI et al. 2015) | Iran | 5 | Ahvaz | 1 | English | $PM_{10}$ | AQMS | NO | NO | 6 | Default value | AirQ default values | Default values | AirQ default values | NR | Yes | Yes | Yes | NO | per 10 µg/m3 increase of concentration | Research | NO |
| (Panahi, Ommi et al. 2017) | Iran | 5 | Tehran | 1 | Persian | $NO_2$ | AQMS | NO | NO | 2 | Default value | AirQ default values | Default values | AirQ default values | NR | Yes | NO | Yes | NO | per 10 µg/m3 increase of concentration | Research | 30-65 years old |
| (Thishan Dharshana 2008) | Sri Lanka | 3 | Colombo | 1 | English | $PM_{10}$ | AQMS | NO | NO | 5 | NR | NR | NR | NR | NR | Yes | Yes | Yes | NO | NO | Research | NO |
| (Fustel, Rueda et al. 2005) | Spain | 4 | Barcelona, Sevilla, Valencia, Madrid, Bilbao | 1 | Spanish | $PM_{10}$, BS | AQMS | I would say Yes. They reported the reference to the rules followed to accept data for use. | NO | 1,2,3,4,5 | Various sources (for short-term BS, PM10 and PM10 40 days and Long-term PM2.5: WHO, 2004; Le Tertre et al , 2002; APHEIS3, Zanobetti et al., 2002; Kunzli et al, 2000; Pope, 2002) | Previous studies | Local | Local | ST/LT | Yes | NO | Yes | NO | Two targets fr BS. Reduction of 5µg/m3 and reduction of days exceeding 20 µg/m3 to 20µg/m3 for BS; Four targets for PM10. reduction of 5µg/m3 and reduction of days exceeding 50 µg/m3 to 50µg/m3 and 30 and 40 ug/m3 for PM10. Two targets for PM2.5 reduction of 3.5µg/m3 and <=15µg/m3. | Research | Yes |
| (Geravandi 2015) | Iran | 5 | Ahvaz | 1 | Persian | $SO_2$ | AQMS | NO | NO | 1,2,3,4 | WHO data | WHO (studies, data, reports and values) | WHO data | WHO (studies, data, reports and values) | NR | Yes | NO | Yes | NO | per 10 µg/m3 increase of concentration | Research | NO |
| (GERAVANDI 2014) | Iran | 5 | Tabriz | 1 | Persian | $PM_{10}$ | AQMS | NO | NO | 4,5 | NR | NR | NR | NR | ST | Yes | Yes | Yes | NO | per 10 µg/m3 increase of concentration | Research | NO |

| Author/date | Country | WHO regions (AFR:1, AMR:2, SEAR: 3, EUR:4, EMR:5, WPR:6) | City | Type of publication (Article:1, Report:2, Thesis:3) | Language | Air Quality data | | | | Health endpoints (*Mortality* (All-cause: 1, CVD:2, Respiratory:3, YLG:10; LCM:11, Cardiopulmonary: 12, *Hospitalization* (CVD:4, Respiratory:5, COPD:6, CD:7, AMI:8, YLL:9, life expectancy: 14, Asthma:15, ARI:16, Acute bronchitis:17) | RR Sources | RR Sources (Categorized) | BI Sources | BI Sources (Categorized) | Type of exposure (Long-term (LT)/ Short-term (ST)) | Number of cases reported (Yes/No) | PAF reported (Yes/No) | CI reported (Yes/No) | Sensitivity/ Comparative analyses conducted (Yes/No) | Cut-off value used to estimate health effects reported (Yes/No) | Motivation or context of study | Population size reported (Yes/No) |
|---|---|---|---|---|---|---|---|---|---|---|---|---|---|---|---|---|---|---|---|---|---|---|
| | | | | | | Air Pollutants | AQMS network, Self-measured, Modeling, Satellite data | Data Coverage reported (Yes/No) | Air quality data processing and validation reported (Yes/No) | | | | | | | | | | | | | |
| (Ntourou, Moustris et al. 2017) | Greece | 4 | Athen | 1 | English | $PM_{10}$ | AQMS (city center, traffic, suburban, and background stations) | NO | NO | 5 | Previous studies | Previous studies | Previous studies | Previous studies | NR | Yes | NO | NO | NO | NO | Research | NO |
| (Geravandi, Goudarzi et al. 2015) | Iran | 5 | Ahvaz | 1 | English | $SO_2$ | AQMS | NO | NO | 1,2,3,4 | WHO data | WHO (studies, data, reports and values) | WHO data | WHO (studies, data, reports and values) | ST | Yes | Yes | Yes | NO | per 10 µg/m3 increase of concentration | Research | NO |
| (Cassadou, Quénel et al. 2003) | France | 4 | 9 cities:Bordeaux, Le Havre, Lille, Lyon, Marseille, Paris, Rouen, Strasbourg, Toulouse | 1 | French | $NO_2$, $SO_2$, $O_3$ | AQMS | NO | NO | 1, 2, 3, 5 | National estimates | National | Local | Local | ST | Yes | NO | NO | NO | Impact of pollution on days when the level is above 10 µg/m (whatever the pollutant), this level characterizing a low pollution situation. This is the number of cases that could be avoided if all pollution indicators were reduced to a level of 10 µg/m. | Research | Yes |
| (Kermani, Dowlati et al. 2017) | Iran | 5 | Mashhad, Tabriz, Isfahan, Shiraz, Tehran, Urmia | 1 | English | $SO_2$, CO | AQMS | NO | Zero values meant that disturbances were removed from calculating average that conducted with moving average. | 1,2,3 | Previous studies | Previous studies | Previous studies | Previous studies | ST | Yes | Yes | Yes | NO | per 10 µg/m3 increase of concentration | Research | NO |
| (Hamid, Goudarzi et al. 2019) | Iran | 5 | Karaj | 1 | Persian | $PM_{2.5}$ | AQMS | NO | NO | 1,4,6 | Previous studies | Previous studies | Previous studies | Previous studies | ST/LT | Yes | Yes | Yes | NO | NO | Research | More than 30 years old and all ages |
| (Pública 2002) | Spain | 4 | Madrid | 2 | Spanish | $PM_{10}$ | AQMS | | | 1, 4,5 | | | | | | | | | | | | |

| Author/date | Country | WHO regions (AFR:1, AMR:2, SEAR: 3, EUR:4, EMR:5, WPR:6) | City | Type of publication (Article:1, Report:2, Thesis:3) | Language | Air Quality data | | | | Health endpoints (*Mortality* (All-cause: 1, CVD:2, Respiratory:3, YLG:10; LCM:11, Cardiopulmonary: 12, *Hospitalization* (CVD:4, Respiratory:5, COPD:6, CD:7, AMI:8, YLL:9, life expectancy: 14, Asthma:15, ARI:16, Acute bronchitis:17) | RR Sources | RR Sources (Categorized) | BI Sources | BI Sources (Categorized) | Type of exposure (Long-term (LT)/ Short-term (ST)) | Number of cases reported (Yes/No) | PAF reported (Yes/No) | CI reported (Yes/No) | Sensitivity/ Comparative analyses conducted (Yes/No) | Cut-off value used to estimate health effects reported (Yes/No) | Motivation or context of study | Population size reported (Yes/No) |
|---|---|---|---|---|---|---|---|---|---|---|---|---|---|---|---|---|---|---|---|---|---|---|
| | | | | | | Air Pollutants | AQMS network, Self-measured, Modeling, Satellite data | Data Coverage reported (Yes/No) | Air quality data processing and validation reported (Yes/No) | | | | | | | | | | | | | |
| (sl 2003) | Spain | 4 | Bilbao | 2 | Spanish | $PM_{10}$, BS | AQMS | Y | NO | 1, 4,5 | Previous studies | Previous studies | Local | Local | ST | Yes | NO | Yes | NO | Benefits from reducing by 5 mg/m3 the values of BS and PM10, and eliminate days with pollution higher than 20 mg/m3 and 50 mg/m3 | Create an air pollution and health monitoring system | Yes |
| (Chardon 2004) | France | 4 | Paris | 2 | French | $PM_{2.5}$ | AQMS | Y | NO | 1, 9, 12, 11, 13, 14 | WHO | WHO (studies, data, reports and values) | Local | Local | LT | They used AirQ for a life table analysis for years of life lost. They used an excel worksheet for the analysis of mortality. This is an example of use of two software. | NO | NO | NO | 3 scenarios : reduction to 20 µ/m reduction to 15 µ/m3 reduction by 3,5 µ/m3 | Research | Yes |
| (Zabalaga 2005) | Bolivia | 2 | Cochabamba | 3 | Spanish | $NO_2$, $O_3$ | AQMS | | | 5 | | | | | | | | | | | Research | |
| (Vicentino 2005) | Italy | 4 | North Vicentino, Italy | 2 | Italian | $PM_{10}$ | AQMS | Y | NO | 1,4,5 | WHO AirQ default values | AirQ default values | Local | Local | ST/LT | Yes | NO | NO | NO | Yes | Analysis by the health authority | Yes |
| (Marquis 2005) | France | 4 | Saint Denis, Reunion island | 3 | French | $PM_{10}$, $O_3$, $NO_2$, $SO_2$ | AQMS | Yes | Yes | 1,2,3,4,5 | Other studies | Previous studies | Local | Local | ST/LT | Yes | NO | Yes | Yes | Yes | Research | Yes |

| Author/date | Country | WHO regions (AFR:1, AMR:2, SEAR: 3, EUR:4, EMR:5, WPR:6) | City | Type of publication (Article:1, Report:2, Thesis:3) | Language | Air Quality data | | | | Health endpoints (*Mortality* (All-cause: 1, CVD:2, Respiratory:3, YLG:10; LCM:11, Cardiopulmonary: 12, *Hospitalization* (CVD:4, Respiratory:5, COPD:6, CD:7, AMI:8, YLL:9, life expectancy: 14, Asthma:15, ARI:16, Acute bronchitis:17) | RR Sources | RR Sources (Categorized) | BI Sources | BI Sources (Categorized) | Type of exposure (Long-term (LT)/ Short-term (ST)) | Number of cases reported (Yes/No) | PAF reported (Yes/No) | CI reported (Yes/No) | Sensitivity/ Comparative analyses conducted (Yes/No) | Cut-off value used to estimate health effects reported (Yes/No) | Motivation or context of study | Population size reported (Yes/No) |
|---|---|---|---|---|---|---|---|---|---|---|---|---|---|---|---|---|---|---|---|---|---|---|
| | | | | | | Air Pollutants | AQMS network, Self-measured, Modeling, Satellite data | Data Coverage reported (Yes/No) | Air quality data processing and validation reported (Yes/No) | | | | | | | | | | | | | |
| (Bologna 2005) | Italy | 4 | Imola | 2 | Italian | $PM_{10}$, $O_3$ | AQMS | NO | NO | 1,2,3,4,5 | WHO AirQ default values | AirQ default values | NR | NR | ST | Yes | Yes | NO | NO | NO | Analysis by the health authority | Yes |
| (Schneider 2006) | Austria | 4 | Austria | 2 | German | $PM_{2.5}$ | AQMS | Yes | Yes | 14 | WHO AirQ default values | AirQ default values | Local | Local | ST | Yes | NO | NO | NO | Cut-off at 8 μ/m3 | Analysis by the environmental authority | Yes |
| (Scarnato 2006) | Italy | 4 | Bologna | 2 | Italian | $PM_{10}$, $PM_{2.5}$, $O_3$ | AQMS | | | 6,9, 1,2,3,4,5 | | | | | LT | | | | | | Analysis by the health authority | Yes |
| (Bešlić 2006) | Croatia | 4 | Zagreb | 2 | English | BS, $PM_{10}$, $PM_{2.5}$, TSP | AQMS | NO | NO | 1,2,3,4,5 | Previous studies | Previous studies | Previous studies | Previous studies | NR | Yes | Yes | Yes | NO | NO | Research | 15-64 years old and less than 15 years old |
| (Paiano 2008) | Italy | 4 | Mazzano e Rezzato | 2 | Italian | $PM_{10}$, $PM_{2.5}$, $O_3$, $NO_2$ | AQMS | NO | NO | 1,2,3,9 | WHO AirQ default values | AirQ default values | Local | Local | ST/LT | Yes | NO | Yes | NO | PM and O3, cut off = 10 µg/m3 | Health Impact assessment | Yes |
| (Forli'-Cesena 2007) | Italy | 4 | Provincia di Forlì-Cesena | 2 | Italian | PM10 | AQMS | NO | NO | 1 | WHO AirQ default values | AirQ default values | Local | Local | ST | Yes | NO | Yes | NO | 10 or 20 or 30 µg/m3 | Analysis by the environmental authority | Yes |
| (Puglia 2007) | Italy | 4 | Ferrara | 2 | Italian | $PM_{2.5}$ | AQMS | NO | NO | 9 | WHO AirQ default values | AirQ default values | Local | Local | LT | Yes | NO | NO | NO | 15 µg/m3 | Report on the health status of the population | Yes |
| (Generale 2008) | Italy | 4 | Comune di Modugno | 2 | Italian | $NO_2$ | AQMS | Yes | Yes | 1,2,3,5,6 | WHO AirQ default values | AirQ default values | Local | Local | ST/LT | Yes | Yes | Yes | NO | 40 µg/m3 and 50 µg/m3 | Analysis by the environmental authority | Yes |
| (Modena 2008) | Italy | 4 | Marzaglia | 2 | Italian | $PM_{10}$, $NO_2$ | AQMS | | | 1,4,5 | | | | | ST/LT | | | | | | | |
| (Dias 2008) | Portugal | 4 | Área Metropolitana de Lisboa (AML) e a Área Metropolitana do Porto (AMP), | 3 | Portuguese | $PM_{2.5}$ | AQMS | Y | NO | 9, 14 | WHO AirQ default values | AirQ default values | Local | Local | LT | Yes | Yes | Yes | NO | Cut-off: AirQ papers | Research | Yes |

| Author/date | Country | WHO regions (AFR:1, AMR:2, SEAR:3, EUR:4, EMR:5, WPR:6) | City | Type of publication (Article:1, Report:2, Thesis:3) | Language | Air Quality data | | | | Health endpoints (*Mortality* (All-cause: 1, CVD:2, Respiratory:3, YLG:10; LCM:11, Cardiopulmonary: 12, *Hospitalization* (CVD:4, Respiratory:5, COPD:6, CD:7, AMI:8, YLL:9, life expectancy: 14, Asthma:15, ARI:16, Acute bronchitis:17) | RR Sources | RR Sources (Categorized) | BI Sources | BI Sources (Categorized) | Type of exposure (Long-term (LT)/ Short-term (ST)) | Number of cases reported (Yes/No) | PAF reported (Yes/No) | CI reported (Yes/No) | Sensitivity/ Comparative analyses conducted (Yes/No) | Cut-off value used to estimate health effects reported (Yes/No) | Motivation or context of study | Population size reported (Yes/No) |
|---|---|---|---|---|---|---|---|---|---|---|---|---|---|---|---|---|---|---|---|---|---|---|
| | | | | | | Air Pollutants | AQMS network, Self-measured, Modeling, Satellite data | Data Coverage reported (Yes/No) | Air quality data processing and validation reported (Yes/No) | | | | | | | | | | | | | |
| (Pérez 2008) | Bolivia | 2 | Cochabamba | 2 | Spanish | $PM_{10}$, $O_3$, $SO_2$, $NO_2$ | AQMS | Y | NO | 1,6,15,16,17 | WHO AirQ default values and from other studies | AirQ default values | Local | Local | ST | Yes | Yes | Yes | NO | PM10=25µg/m3 , O3=50µg/m3 , NO2=100µg/m3, SO=10µg/m3 | Research | Yes |
| (Ambiente 2007) | Peru | 2 | Lima | 2 | Spanish | TSP, $PM_{10}$ | AQMS | NO | NO | 1,2,3,6,5,4, | WHO AirQ default values and from other studies | AirQ default values | Local | Local | ST/LT | Yes | NO | NO | NO | WHO AQG | Analysis by the environmental authority | Yes |
| (Milano 2009) | Italy | 4 | Milan | 2 | Italian | $PM_{10}$, $PM_{2.5}$, $O_3$, $NO_2$ | AQMS | NO | NO | 1,2,3,14,9,4 | WHO AirQ default values and from other studies | AirQ default values | Local | Local | ST/LT | Yes | Yes | Yes | NO | 30 µg/m3 (PM2.5), 40 µg/m3 (PM10), 70 µg/m3 (O3), 40µg/m3 (NO2) | Analysis by the health authority | Yes |
| (Pokorný 2009) | Czech Republic | 4 | Jihlava | 2 | Czech | $NO_2$, $PM_{10}$, $O_3$, $PM_{2.5}$ | AQMS | Yes | Yes | 1,2,3,5,9,14,15 | WHO AirQ default values | AirQ default values | Local | Local | ST/LT | Yes | Yes | NO | NO | PM10 : 20µg/m3, O3 : 100µg/m3, PM2.5: 15µg/m3 | Analysis by the health authority | Yes |
| (Uapipatanakul 2009) | Thailand | 3 | Maptaphut and Rayong city | 3 | English | $NO_2$, $SO_2$, $PM_{10}$, $O_3$ | AQMS and modeling | NO | NO | 5 | WHO data | WHO (studies, data, reports and values) | Local | Local | NR | Yes | NO | NO | NO | NO | monsoon effect | NO |
| (Vitolo 2009) | Italy | 4 | Comune di Borgo a Mozzano (Lucca) | 2 | Italian | $PM_{2.5}$ | AQMS | NO | NO | 9 | WHO AirQ default values | AirQ default values | Local | Local | LT | Yes | NO | NO | NO | AQ data evaluated against AQG values of 20 and 10 µgr/m3 | Expert report for an Environmental Impact Assessment procedure carry out by a group of citizens vs an industrial activity | Yes |
| (Polato 2010) | Italy | 4 | Udine | 2 | Italian | $PM_{10}$, $O_3$ | AQMS | Yes | Yes | 1 | WHO AirQ default values | AirQ default values | Local | Local | ST/LT | Yes | Yes | Yes | NO | PM10 : 20µg/m3, O3 : 100µg/m3, | Analysis by the health authority | Yes |
| (Rocchetti 2010) | Italy | 4 | 16 municipalities in the Marche Region | 2 | Italian | $PM_{10}$, $O_3$ | AQMS | Y | NO | 1,2,3,4,5 | WHO AirQ default values | AirQ default values | Local | Local | ST/LT | Yes | NO | Yes | NO | PM10=10µgr/m3, O3=70 µg/m3 | Analysis by the environmental authority | Yes |
| (Spangl 2010) | Austria | 4 | Styria region | 2 | German | $PM_{2.5}$ | AQMS | Yes | Yes | 14 | WHO AirQ default values | AirQ default values | Local | Local | LT | Yes | NO | NO | NO | PM2.5=7 µg/m³ | Analysis by the environmental authority | Yes |

| Author/date | Country | WHO regions (AFR:1, AMR:2, SEAR: 3, EUR:4, EMR:5, WPR:6) | City | Type of publication (Article:1, Report:2, Thesis:3) | Language | Air Quality data | | | | Health endpoints (*Mortality* (All-cause: 1, CVD:2, Respiratory:3, YLG:10; LCM:11, Cardiopulmonary: 12, *Hospitalization* (CVD:4, Respiratory:5, COPD:6, CD:7, AMI:8, YLL:9, life expectancy: 14, Asthma:15, ARI:16, Acute bronchitis:17) | RR Sources | RR Sources (Categorized) | BI Sources | BI Sources (Categorized) | Type of exposure (Long-term (LT)/ Short-term (ST)) | Number of cases reported (Yes/No) | PAF reported (Yes/No) | CI reported (Yes/No) | Sensitivity/ Comparative analyses conducted (Yes/No) | Cut-off value used to estimate health effects reported (Yes/No) | Motivation or context of study | Population size reported (Yes/No) |
|---|---|---|---|---|---|---|---|---|---|---|---|---|---|---|---|---|---|---|---|---|---|---|
| | | | | | | Air Pollutants | AQMS network, Self-measured, Modeling, Satellite data | Data Coverage reported (Yes/No) | Air quality data processing and validation reported (Yes/No) | | | | | | | | | | | | | |
| (Campri 2011) | Italy | 4 | Bologna | 3 | Italian | $NO_2$ | AQMS | Yes | Yes | 1,2,6,5 | WHO AirQ default values | AirQ default values | Local | Local | ST | Yes | Yes | Yes | NO | 10 μg/m3 | Research | Yes |
| (Bešlić 2011) | Croatia | 4 | Zagreb | 2 | Croatian | $PM_{2.5}$ | AQMS | NO | NO | 1, 2,3,9 | WHO AirQ default values | AirQ default values | Local | Local | ST/LT | Yes | Yes | Yes | NO | 10 μg/m3 | Analysis by the environmental authority | Yes |
| (Orru 2011) | Estonia | 4 | Tallinn, Tartu, Kohtla Jarve, Narva and Parnu | 2 | Estonian | $PM_{2.5}$, $PM_{10}$ | AQMS | NO | NO | 9, 14, 4, 5 | WHO AirQ default values and from other studies | AirQ default values | Local | Local | ST/LT | Yes | NO | Yes | NO | Yes, national limits | Research | Yes |
| (Bartolacci 2012) | Italy | 4 | Ancona | 2 | Italian | $PM_{10}$, $PM_{2.5}$, $O_3$ | AQMS | Yes | Yes | 1, 2, 3, 4, 5, 9 | WHO AirQ default values | AirQ default values | Local | Local | ST/LT | Yes | NO | Yes | Yes | PM10 = 50 μg/m3 and = 10 μg/m3; O3 = 120 μg/m3 and = 70 μg/m3; PM2.5 = 10 μg/m3 and = 20 μg/m3 | Analysis by the health and the environmental authorities | Yes |
| (Bortoletto 2013) | Italy | 4 | Farra di Soligo, Moriago, Battle, Pederobba and Sernaglia della Battaglia (Province of Treviso) | 2 | Italian | $PM_{10}$ | AQMS | NO | NO | 1, 2, 3, 4,5 | WHO AirQ default values | AirQ default values | Local | Local | ST | Yes | Yes | Yes | NO | PM10=20μgr/m3 | Analysis by the health authority | Yes |
| (Orru 2013) | Sweden | 4 | Stockholm | 2 | English | NOX and $PM_{10}$ | AQMS and modeling | NO | NO | 9, 14, 4, 5 | Previous studies | Previous studies | Previous studies | Previous studies | ST/LT | Yes | NO | Yes | NO | per 10 μg/m3 increase of concentration | Tunnel users | Less than 19 and 20-65 years old |
| (Bobvos 2014) | Hungary | 4 | 14 cities in Hungary (Budapest, Debrecen, Eger, Győr, Miskolc, Nyíregyháza, Pécs, Salgótarján, Szeged, Székesfehérvár, Szolnok, Tatabánya, Várpalota, Veszprém) | 2 | Hungarian | $PM_{2.5}$, $PM_{10}$ | AQMS | NO | NO | 1, 10 | WHO AirQ default values | AirQ default values | Local | Local | ST/LT | Yes | NO | Yes | Yes | Calculation of avoidable mortality under short-term scenarios:<br>§ Reduction of days above the daily PM10 limit value (50 μg/m3) to 50 μg/m3<br>§ Reduction of annual PM10 values by 5 μg/m3,<br>§ Reduction of annual PM10 values to 20 μg/m3<br>Calculation of avoidable deaths and life-year gains for long-term scenarios: | Analysis by the environmental authority | Yes |

| Author/date | Country | WHO regions (AFR:1, AMR:2, SEAR: 3, EUR:4, EMR:5, WPR:6) | City | Type of publication (Article:1, Report:2, Thesis:3) | Language | Air Quality data | | | | Health endpoints (*Mortality* (All-cause: 1, CVD:2, Respiratory:3, YLG:10; LCM:11, Cardiopulmonary: 12, *Hospitalization* (CVD:4, Respiratory:5, COPD:6, CD:7, AMI:8, YLL:9, life expectancy: 14, Asthma:15, ARI:16, Acute bronchitis:17) | RR Sources | RR Sources (Categorized) | BI Sources | BI Sources (Categorized) | Type of exposure (Long-term (LT)/ Short-term (ST)) | Number of cases reported (Yes/No) | PAF reported (Yes/No) | CI reported (Yes/No) | Sensitivity/ Comparative analyses conducted (Yes/No) | Cut-off value used to estimate health effects reported (Yes/No) | Motivation or context of study | Population size reported (Yes/No) |
|---|---|---|---|---|---|---|---|---|---|---|---|---|---|---|---|---|---|---|---|---|---|---|
| | | | | | | Air Pollutants | AQMS network, Self-measured, Modeling, Satellite data | Data Coverage reported (Yes/No) | Air quality data processing and validation reported (Yes/No) | | | | | | | | | | | | | |
| | | | | | | | | | | | | | | | | | | | | § Reduction of annual PM2.5 by 5 μg/m3 and Reduction of annual PM2.5 by 10 μg/m3 | | |
| (Stivanello 2014) | Italy | 4 | Bologna | 2 | Italian | $PM_{10}$, $PM_{2.5}$, $O_3$, $NO_2$ | AQMS | Yes | Yes | 1, 2, 3, 4, 5, 9 | WHO suggested values | WHO (studies, data, reports and values) | Local | Local | ST/LT | Yes | Yes | Yes | Yes | PM10=40;20;10 PM2.5=20;10 O3=110;70;10 | Analysis by the health authority | Yes |
| (Negri 2015) | Italy | 4 | Realmonte | 2 | Italian | $PM_{10}$, $PM_{2.5}$ | Modeling | NO | Yes | 1, 2, 3,9 | WHO AirQ default values | AirQ default values | Local | Local | ST/LT | Yes | Yes | Yes | NO | PM2.5 and PM10 = 10 μg/m3 | Report for an HIA to authorize an industrial activity | Yes |
| (Orru 2016) | Estonia | 4 | Estonia | 2 | Estonian | O3 | Modeling | Yes | Yes | Mortality long-term exposure, life years lost per year; average loss of life expectancy per capita; respiratory and cardiovascular hospitalisations per year. | WHO AirQ default values and from other studies | AirQ default values/ Previous studies | Local | Local | ST/LT | Yes | NO | Yes | Yes | O3 = 25 ppb and = 35 and = 10 ppb. | Research | Yes |
| (Bednářová 2007) | Czech Republic | 4 | Czechia | 3 | Czech | $PM_{10}$, $NO_2$ | Modeling | NO | Yes | 1,2,3,4,5,6 | WHO AirQ default values and from other studies | AirQ default values/ Previous studies | Local | Local | ST/LT | Yes | NO | Yes | NO | NO | Research | Yes |
| (Skotak 2008) | Poland | 4 | southern part of Poland | 2 | Polish | $PM_{10}$ | AQMS | Yes | Yes | 1,2,3,4,5 | WHO AirQ default values | AirQ default values | NR | NR | ST/LT | Yes | NO | NO | NO | national standards | Research | NO |
| (Šega 2006) | Croatia | 4 | Zagreb | 3 | English | TSP, BS, $SO_2$, $PM_{10}$, $PM_{2.5}$ | AQMS | NO | Yes | 1,2,3,4,5,6 | WHO AirQ default values | AirQ default values | Default values from AirQ | AirQ default values | ST/LT | Yes | Yes | Yes | Yes | Cut-off values suggested by AirQ | Analysis by the health authority | Yes |
| (Borsi, Goudarzi et al. 2022) | Iran | 5 | Ahvaz | 1 | English | $PM_{10}$, $NO_2$, $O_3$, $SO_2$ | AQMS | NO | NO | 2, 4, 5 | Previous studies | Previous studies | Previous studies | Previous studies | NR | Yes | NO | NO | NO | NO | Research | NO |
| (Fard, Torkamani et al. 2021) | Iran | 5 | Arak | 1 | English | $PM_{2.5}$, $PM_{10}$ | AQMS | NO | NO | 1,2,3,4,5 | reference documents (AirQ+) | AirQ default values | Default values | AirQ default values | ST | Yes | Yes | Yes | NO | per 10 μg/m3 increase of concentration | Research | NO |

| Author/date | Country | WHO regions (AFR:1, AMR:2, SEAR:3, EUR:4, EMR:5, WPR:6) | City | Type of publication (Article:1, Report:2, Thesis:3) | Language | Air Quality data | | | | Health endpoints (*Mortality* (All-cause: 1, CVD:2, Respiratory:3, YLG:10; LCM:11, Cardiopulmonary: 12, *Hospitalization* (CVD:4, Respiratory:5, COPD:6, CD:7, AMI:8, YLL:9, life expectancy: 14, Asthma:15, ARI:16, Acute bronchitis:17) | RR Sources | RR Sources (Categorized) | BI Sources | BI Sources (Categorized) | Type of exposure (Long-term (LT)/ Short-term (ST)) | Number of cases reported (Yes/No) | PAF reported (Yes/No) | CI reported (Yes/No) | Sensitivity/ Comparative analyses conducted (Yes/No) | Cut-off value used to estimate health effects reported (Yes/No) | Motivation or context of study | Population size reported (Yes/No) |
|---|---|---|---|---|---|---|---|---|---|---|---|---|---|---|---|---|---|---|---|---|---|---|
| | | | | | | Air Pollutants | AQMS network, Self-measured, Modeling, Satellite data | Data Coverage reported (Yes/No) | Air quality data processing and validation reported (Yes/No) | | | | | | | | | | | | | |
| (Rameez, Varekar et al. 2020) | India | 3 | Navi Mumbai | 1 | English | $PM_{10}$, PM2.5, SO2, NO2 | AQMS | NO | NO | 1, 2, 3, 4, 5, 6 | Previous studies (AirQ+) | AirQ default values | Previous studies | Previous studies | ST | Yes | Yes | Yes | NO | NO | Solid Waste Disposal Facility in monsoon and non-monsoon seasons | NO |
| (Tabibzadeh, Hosseini et al. 2022) | Iran | 5 | Shiraz | 1 | English | $PM_{10}$ | AQMS | NO | NO | 2, 3 | Previous studies | Previous studies | epidemiological studies conducted by the WHO and systematic reviews | Previous studies | LT | Yes | Yes | Yes | NO | per 10 µg/m3 increase of concentration | Research | NO |
| (Toolabi, Bonyadi et al. 2022) | Iran | 5 | Rigan | 1 | English | $PM_{2.5}$, $PM_{10}$ | AQMS | NO | NO | 1,2,3,4,5,8 | Previous studies | Previous studies | Previous studies | Previous studies | ST | Yes | Yes | Yes | NO | per 10 µg/m3 increase of concentration | Research | NO |
| (Bahrami Asl, Amini Rabati et al. 2022) | Iran | 5 | Tabriz and Urmia | 1 | English | $PM_{10}$, $O_3$, $SO_2$, and $NO_2$ | AQMS | NO | NO | 3, 6 | Previous studies | Previous studies | Previous studies | Previous studies | ST | Yes | Yes | Yes | NO | per 10 µg/m3 increase of concentration | Research | NO |
| (Xu, Zhang et al. 2022) | China | 6 | Cities surrounding Beijing | 1 | English | $PM_{2.5}$, $PM_{10}$, CO, $SO_2$, $NO_2$, and $O_3$ | AQMS | NO | NO | 1,2,3 | Previous studies | Previous studies | Previous studies | Previous studies | NR | Yes | Yes | Yes | NO | per 10 µg/m3 increase of concentration | Research | NO |
| (Gharehchahi, Mahvi et al. 2013) | Iran | 5 | Shiraz | 1 | English | $PM_{10}$, $NO_2$, $SO_2$ | AQMS | NO | NO | 4,5,6 | Localized using local statistics and WHO AirQ software | AirQ default values/ Local | Localized using local statistics and WHO AirQ software | AirQ default values/ Local | ST | Yes | NO | NO | NO | per 10 µg/m3 increase of concentration | Research | NO |
| (Kermani, Dowlati et al. 2016) | Iran | 5 | Tehran | 1 | Persian | $O_3$, $NO_2$, $SO_2$ | AQMS | The ratio between the numbers of valid data for the two seasons (warm and cold) should not be more than 2, at least 75% of valid | NO | 1,2,3,6,8 | NR | NR | NR | NR | NR | Yes | Yes | Yes | NO | per 10 µg/m3 increase of concentration | Research | NO |

| Author/date | Country | WHO regions (AFR:1, AMR:2, SEAR: 3, EUR:4, EMR:5, WPR:6) | City | Type of publication (Article:1, Report:2, Thesis:3) | Language | Air Quality data | | | | Health endpoints (*Mortality* (All-cause: 1, CVD:2, Respiratory:3, YLG:10; LCM:11, Cardiopulmonary: 12, *Hospitalization* (CVD:4, Respiratory:5, COPD:6, CD:7, AMI:8, YLL:9, life expectancy: 14, Asthma:15, ARI:16, Acute bronchitis:17) | RR Sources | RR Sources (Categorized) | BI Sources | BI Sources (Categorized) | Type of exposure (Long-term (LT)/ Short-term (ST)) | Number of cases reported (Yes/No) | PAF reported (Yes/No) | CI reported (Yes/No) | Sensitivity/ Comparative analyses conducted (Yes/No) | Cut-off value used to estimate health effects reported (Yes/No) | Motivation or context of study | Population size reported (Yes/No) |
|---|---|---|---|---|---|---|---|---|---|---|---|---|---|---|---|---|---|---|---|---|---|---|
| | | | | | | Air Pollutants | AQMS network, Self-measured, Modeling, Satellite data | Data Coverage reported (Yes/No) | Air quality data processing and validation reported (Yes/No) | | | | | | | | | | | | | |
| | | | | | | | | data must exist in order to achieve the average one-hour values from data with a shorter average time, at least 75% of one-hour data (18 hours) should exist and be valid to access the values of the eight-hour moving average form one-hour data. | | | | | | | | | | | | | | |
| (KERMANI, AGHAEI et al. 2016) | Iran | 5 | Tehran, Mashhad, Isfahan, Shiraz, Tabriz, Urmia | 1 | Persian | SO2 | AQMS | The ratio between the numbers of valid data for the two seasons | NO | 1,2,3,6,8 | NR | NR | NR | NR | NR | Yes | Yes | Yes | NO | per 10 µg/m3 increase of concentration | Research | NO |

| Author/date | Country | WHO regions (AFR:1, AMR:2, SEAR: 3, EUR:4, EMR:5, WPR:6) | City | Type of publication (Article:1, Report:2, Thesis:3) | Language | Air Quality data | | | | Health endpoints (*Mortality* (All-cause: 1, CVD:2, Respiratory:3, YLG:10; LCM:11, Cardiopulmonary: 12, *Hospitalization* (CVD:4, Respiratory:5, COPD:6, CD:7, AMI:8, YLL:9, life expectancy: 14, Asthma:15, ARI:16, Acute bronchitis:17) | RR Sources | RR Sources (Categorized) | BI Sources | BI Sources (Categorized) | Type of exposure (Long-term (LT)/ Short-term (ST)) | Number of cases reported (Yes/No) | PAF reported (Yes/No) | CI reported (Yes/No) | Sensitivity/ Comparative analyses conducted (Yes/No) | Cut-off value used to estimate health effects reported (Yes/No) | Motivation or context of study | Population size reported (Yes/No) |
|---|---|---|---|---|---|---|---|---|---|---|---|---|---|---|---|---|---|---|---|---|---|---|---|
| | | | | | | Air Pollutants | AQMS network, Self-measured, Modeling, Satellite data | Data Coverage reported (Yes/No) | Air quality data processing and validation reported (Yes/No) | | | | | | | | | | | | | | |
| | | | | | | | | (warm and cold) should not be more than 2, at least 75% of valid data must exist in order to achieve the average one-hour values from data with a shorter average time, at least 75% of one-hour data (18 hours) should exist and be valid to access the values of the eight-hour moving average form one-hour data. | | | | | | | | | | | | | | | |

| Author/date | Country | WHO regions (AFR:1, AMR:2, SEAR:3, EUR:4, EMR:5, WPR:6) | City | Type of publication (Article:1, Report:2, Thesis:3) | Language | Air Quality data | | | | Health endpoints (*Mortality* (All-cause: 1, CVD:2, Respiratory:3, YLG:10; LCM:11, Cardiopulmonary: 12, *Hospitalization* (CVD:4, Respiratory:5, COPD:6, CD:7, AMI:8, YLL:9, life expectancy: 14, Asthma:15, ARI:16, Acute bronchitis:17) | RR Sources | RR Sources (Categorized) | BI Sources | BI Sources (Categorized) | Type of exposure (Long-term (LT)/ Short-term (ST)) | Number of cases reported (Yes/No) | PAF reported (Yes/No) | CI reported (Yes/No) | Sensitivity/ Comparative analyses conducted (Yes/No) | Cut-off value used to estimate health effects reported (Yes/No) | Motivation or context of study | Population size reported (Yes/No) |
|---|---|---|---|---|---|---|---|---|---|---|---|---|---|---|---|---|---|---|---|---|---|---|
| | | | | | | Air Pollutants | AQMS network, Self-measured, Modeling, Satellite data | Data Coverage reported (Yes/No) | Air quality data processing and validation reported (Yes/No) | | | | | | | | | | | | | |
| (Kermani, Dowlati et al. 2016) | Iran | 5 | Tehran | 1 | English | $O_3$, $NO_2$, $SO_2$, $PM_{10}$ | AQMS | The ratio between the number of valid data for the 2 seasons (warm and cold season) should not be more than 2. Also, in order to achieve the median 24-hour values, at least 50% data must exist with sufficient validity | NO | 4, 5, 8, 6 | WHO AirQ default values | AirQ default values | WHO AirQ default values | AirQ default values | NR | Yes | Yes | Yes | NO | per 10 μg/m$^3$ increase of concentration | Research | NO |
| (Kermani, Dowlati et al. 2018) | Iran | 6 | Tehran | 1 | Persian | $SO_2$ | AQMS | NO | NO | 1,2,3,6,8 | WHO AirQ default values | AirQ default values | WHO AirQ default values | AirQ default values | NR | Yes | Yes | NO | NO | per 10 μg/m$^3$ increase of concentration | Research | NO |

**Table S3.** A summary of included studies that used AirQ+ for HIA from 2017 to 2022 (Abbreviations used in the table: AFR (African Region), AMR (Region of Americas), SEAR (South-East Asian Region), EUR (European Region), WPR (Western Pacific Region), AQMS (Air Quality Monitoring Station), NR (Not data reported), RR (Relative Risk), BI (Baseline Incidence), CI (Confidence Interval)).

| **ID/ Author date** | **Country of study** | **WHO regions (AFR:1, AMR:2, SEAR: 3, EUR:4, EMR:5, WPR:6)** | **Name of city** | **Air quality** | | | | | **Health endpoints** | **RR Sources** | **BI Sources** | **BI Sources (Categorized)** | **Type of exposure (Long-term (LT)/ Short-term (ST))** | **Number of cases reported (Yes/No)** | **PAF reported (Yes/No)** | **CI reported (Yes/No)** | **Sensitivity analyses (Yes/No)** | **Cut-off value (μg/m³) to estimate health effects** | **Motivation or context of study** | **Population Size reported (Yes/No)** | **use of life-tables module** |
|---|---|---|---|---|---|---|---|---|---|---|---|---|---|---|---|---|---|---|---|---|---|
| | | | | **Air Pollutants (APs)** | **AQMS or self-measured - Modeling - satellite** | **Type of montoring stations (Res:1/TR:2/BG:3/Ind:4/ None:5, Commercial:6)** | **Data Coverage reported (Yes/No)** | **Air quality data processing and validation reported (Yes/No)** | | | | | | | | | | | | | |
| (Hadei, Nazari et al. 2017) | Iran | 5 | Tehran, Mashhad, Isfahan, Shiraz, Tabriz, Ahvaz, Arak, Sanandaj, Khoram Abad and Ilam | $PM_{2.5}$ | Self-measured | 1 | Y | Zero, negative and outliers, WHO approach | Lung cancer (LC) Mortality | AirQ+ default | Ministry of Health | National | LT | Y | Y | Y | N | 10 | Research | N | N |
| (Al-Hemoud, Gasana et al. 2018) | Kuwait | 5 | All over the Kuwait | $PM_{2.5}$<br>$PM_{10}$ | AQMS | NR | Y | Aphekom project (Pascal et al. 2011) | Mortality (All)<br>Post neonatal infant mortality | AirQ+ default | Ministry of Health | National | LT | Y | N | Y | Y | 35<br>70 | Policy/Planning | Y | Y<br>N |
| (Aliyu and Botai 2018) | Nigeria | 1 | Zaria | $PM_{2.5}$<br>$PM_{10}$ | AQMS | 1,2,3 | Y | NR | Hospital (Respiratory)<br>Hospital (Respiratory) | AirQ+ default | Local hospitals | Local | ST | Y | N | Y | Y | 25<br>50 | Policy/Planning | N | |
| (De Marco, Amoatey et al. 2018) | Italy | 4 | Rome | $PM_{2.5}$ | AQMS | 1,2,3 | Y | NR | Mortality (COPD, IHD), Hospital (CVD, Respiratory) | Previous studies | Previous studies | Previous studies | LT/ ST | Y | Y | Y | N | 10 | Research | N | N |
| (Faridi, Shamsipour et al. 2018) | Iran | 5 | Tehran | $PM_{2.5}$<br>$O_3$ | AQMS | NR | Y | Z-score | Mortality (All, COPD, LC, IHD, stroke, ALRI)<br>Mortality (Respiratory) | AirQ+ default | Ministry of Health | National | LT | Y | N | Y | N | 10<br>SOMO35 | Research | Y | Y |

| ID/ Author date | Country of study | WHO regions (AFR:1, AMR:2, SEAR: 3, EUR:4, EMR:5, WPR:6) | Name of city | Air quality | | | | | Health endpoints | RR Sources | BI Sources | BI Sources (Categorized) | Type of exposure (Long-term (LT)/ Short-term (ST)) | Number of cases reported (Yes/No) | PAF reported (Yes/No) | CI reported (Yes/No) | Sensitivity analyses (Yes/No) | Cut-off value (µg/m$^3$) to estimate health effects | Motivation or context of study | Population Size reported (Yes/No) | use of life-tables module |
|---|---|---|---|---|---|---|---|---|---|---|---|---|---|---|---|---|---|---|---|---|---|
| | | | | Air Pollutants (APs) | AQMS or self-measured - Modeling - satellite | Type of montoring stations (Res:1/TR:2/BG:3/Ind:4/ None:5, Commercial:6) | Data Coverage reported (Yes/No) | Air quality data processing and validation reported (Yes/No) | | | | | | | | | | | | | |
| (Hopke, Nazari et al. 2018) | Iran | 5 | Tehran, Mashhad, Isfahan, Shiraz, Tabriz, Ahvaz, Arak, Sanandaj, Khoram Abad, and Ilam. | $PM_{2.5}$ | AQMS | 1,2,3,4 | Y | Zero, negative, EU and WHO approach | Mortality (All) | AirQ+ default | Ministry of Health | National | ST | Y | Y | Y | N | 25 | Research | N | N |
| (Yarahmadi, Hadei et al. 2018) | Iran | 5 | Tehran | $PM_{2.5}$ | AQMS | 1 | Y | Zero, negative, EU and WHO approach | Mortality (All, COPD, LC) | AirQ+ default | Ministry of Health | National | LT | Y | Y | Y | N | 10 | Research | N | N |
| (Amoatey, Takdastan et al. 2019) | Iran | 5 | Ahvaz | $O_3$ | AQMS | NR | Y | NR | Mortality (All, CVD, Respiratory) | Previous studies | Previous studies | Previous studies | LT/ST | Y | Y | Y | N | SOMO35 | Research | N | N |
| (Ansari and Ehrampoush 2019) | Iran | 5 | Tehran | $PM_{2.5}$ | AQMS | NR | Y | NR | Mortality (All, IHD, COPD, Stroke, ALRI) | AirQ+ default | Ministry of Health and Civil Registration Office | National | LT | Y | Y | Y | N | 10 | Research | N | N |
| (Ebrahimi Ghadi, Qaderi et al. 2019) | Iran | 5 | Tehran | $NO_2$ | AQMS | NR | Y | Modelled data | Mortality (All) | AirQ+ default | NR | NR | ST | Y | Y | Y | Y | NR | Research | N | N |
| (Gupta, Jangid et al. 2019) | India | 3 | Agra | $PM_{2.5}$ | AQMS data and Modelling | NR | N | NR | Mortality (All, COPD, LC, Stroke) | AirQ+ default | NR | NR | LT | N | Y | Y | N | NR | Research | N | N |
| | | | | $PM_{10}$ | | | | | Mortality (All, Bronchitis in adults and children) | | | | | | | | | NR | | | |
| (Karimi, Shirmardi et al. 2019) | Iran | 5 | Ahvaz | $PM_{2.5}$ | Self-measured | NR | Y | Zero and negatives | Mortality (All, COPD, LC, IHD, ALRI) | AirQ+ default | Previous studies | Previous studies | LT/ST | Y | Y | Y | N | 10, 25 | Research | Y | N |
| | | | | $NO_2$ | | | | | Mortality (All) | | | | | | | | | 40, 200 | | | |
| | | | | $O_3$ | | | | | Mortality (All, Respiratory) | | | | | | | | | SOMO35 | | | |

| ID/ Author date | Country of study | WHO regions (AFR:1, AMR:2, SEAR: 3, EUR:4, EMR:5, WPR:6) | Name of city | Air quality | | | | | Health endpoints | RR Sources | BI Sources | BI Sources (Categorized) | Type of exposure (Long-term (LT)/ Short-term (ST)) | Number of cases reported (Yes/No) | PAF reported (Yes/No) | CI reported (Yes/No) | Sensitivity analyses (Yes/No) | Cut-off value (µg/m$^3$) to estimate health effects | Motivation or context of study | Population Size reported (Yes/No) | use of life-tables module |
|---|---|---|---|---|---|---|---|---|---|---|---|---|---|---|---|---|---|---|---|---|---|
| | | | | Air Pollutants (APs) | AQMS or self-measured - Modeling - satellite | Type of montoring stations (Res:1/TR:2/BG:3/Ind:4/ None:5, Commercial:6) | Data Coverage reported (Yes/No) | Air quality data processing and validation reported (Yes/No) | | | | | | | | | | | | | |
| (Mehmood, Tianle et al. 2019) | Pakistan | 5 | Islamabad | $PM_{2.5}$, $PM_{10}$ | AQMS | NR | Y | NR | Mortality all-cause, heart disease and (LC), Hospital respiratory and cardiovascular | AirQ+ default | Previous studies | Previous studies | NR | N | N | Y | N | NR | Strategy/policy | N | N |
| (Sicard, Khaniabadi et al. 2019) | France, Iran, Italy | 4,5 | Rome, Naples and Livorno in Italy; Marseille, Nice and Perpignan in France; and Ahvāz, Arāk and Kermānshāh in Iran | $PM_{2.5}$ | AQMS | 1 | Y | NR | Mortality (All, CVD, Res, COPD), Hospital (CVD, Res) | Previous studies | Previous studies | Previous studies | ST | Y | N | Y | N | 10 | Research | N | N |
| | | | Rome, Naples and Livorno in Italy; Marseille, Nice and Perpignan in France; and Ahvāz, Arāk and Kermānshāh in Iran | $O_3$ | | | | | Mortality (All, CVD, Respiratory), Hospital (CVD, Respiratory) | | | | | | | | | 20 | | | |
| | | | Rome, Naples and Livorno in Italy; Marseille, Nice and Perpignan in France; and Ahvāz, Arāk and Kermānshāh in Iran | $PM_{10}$ | | | | | Mortality (All, CVD, Respiratory), Hospital (CVD, Respiratory) | | | | | | | | | 20 | | | |

| **ID/ Author date** | **Country of study** | **WHO regions (AFR:1, AMR:2, SEAR:3, EUR:4, EMR:5, WPR:6)** | **Name of city** | **Air quality** | | | | | **Health endpoints** | **RR Sources** | **BI Sources** | **BI Sources (Categorized)** | **Type of exposure (Long-term (LT)/ Short-term (ST))** | **Number of cases reported (Yes/No)** | **PAF reported (Yes/No)** | **CI reported (Yes/No)** | **Sensitivity analyses (Yes/No)** | **Cut-off value (µg/m³) to estimate health effects** | **Motivation or context of study** | **Population Size reported (Yes/No)** | **use of life-tables module** |
|---|---|---|---|---|---|---|---|---|---|---|---|---|---|---|---|---|---|---|---|---|---|
| | | | | **Air Pollutants (APs)** | **AQMS or self-measured - Modeling - satellite** | **Type of montoring stations (Res:1/TR:2/BG:3/Ind:4/ None:5, Commercial:6)** | **Data Coverage reported (Yes/No)** | **Air quality data processing and validation reported (Yes/No)** | | | | | | | | | | | | | |
| (Sówka, Nych et al. 2019) | Poland | 4 | 12 Polish cities | $PM_{2.5}$ | AQMS | 1,3,4 | Y | NR | Mortality (All), Hospital (CVD, Respiratory), Incidence of asthma | AirQ+ default | Central Statistical Office and Ministry of Health | National | LT/ST | Y | N | Y | N | 10 | Research, Strategy/policy planning | N | N |
| | | | | $PM_{10}$ | | | | | Mortality (All for children) | | | | | | | | | | | | |
| (Todorović, Radenković et al. 2019) | Serbia | 4 | Belgrade, Novi Sad and Nis | $PM_{10}$ | AQMS | 1,2 | Y | NR | Mortality (All, CVD, Respiratory) | Previous studies | Statistical Office | National | LT | Y | Y | Y | N | 10, 20 | Research, Strategy/policy planning | N | N |
| | | | | $NO_2$ | | | | | Mortality (All, CVD) | | | | | | | | | 10, 20 | | | |
| | | | | $O_3$ | | | | | Mortality (All, CVD, Respiratory) | | | | | | | | | 10, 20 | | | |
| | | | | $SO_2$ | | | | | Mortality (All, CVD, Respiratory) | | | | | | | | | 10, 20 | | | |
| (Afghan and Patidar 2020) | India | 3 | Delhi | $PM_{2.5}$ | AQMS | NR | N | NR | Mortality (All, COPD, LC, IHD, Stroke, ALRI), Hospital (CVD, Respiratory) | AirQ+ default | Previous studies | Previous studies | LT/ST | Y | N | Y | Y | 10 | Research | N | N |
| | | | | $PM_{10}$ | | | | | Mortality (All for children), Bronchitis in adults and children, Incidence of asthma | | | | | | | | | 20 | | | |
| | | | | $NO_2$ | | | | | Mortality (All), bronchitis in children | | | | | | | | | 50 | | | |
| (Amoatey, Sicard et al. 2020) | Italy | 4 | Rome | $PM_{2.5}$ | AQMS | NR | Y | NR | Mortality (IHD, LC, COPD, stroke) | AirQ+ default | Previous studies | Previous studies | LT | Y | Y | Y | N | 10 | Research | N | N |

| ID/ Author date | Country of study | WHO regions (AFR:1, AMR:2, SEAR:3, EUR:4, EMR:5, WPR:6) | Name of city | Air quality | | | | | Health endpoints | RR Sources | BI Sources | BI Sources (Categorized) | Type of exposure (Long-term (LT)/ Short-term (ST)) | Number of cases reported (Yes/No) | PAF reported (Yes/No) | CI reported (Yes/No) | Sensitivity analyses (Yes/No) | Cut-off value (µg/m³) to estimate health effects | Motivation or context of study | Population Size reported (Yes/No) | use of life-tables module |
|---|---|---|---|---|---|---|---|---|---|---|---|---|---|---|---|---|---|---|---|---|---|---|
| | | | | Air Pollutants (APs) | AQMS or self-measured - Modeling - satellite | Type of montoring stations (Res:1/TR:2/BG:3/Ind:4/ None:5, Commercial:6) | Data Coverage reported (Yes/No) | Air quality data processing and validation reported (Yes/No) | | | | | | | | | | | | | |
| (Barzeghar, Sarbakhsh et al. 2020) | Iran | 5 | Tabriz | $PM_{2.5}$ | AQMS | NR | Y | Z-score | Mortality (All, IHD, LC, COPD, Stroke, ALRI) | AirQ+ default | Ministry of Health | National | LT | Y | Y | Y | N | 10 | Research | N | N |
| | | | | $PM_{10}$ | | | | | Mortality (infants), bronchitis in adults | | | | | | | | | 20 | | | |
| | | | | $O_3$ | | | | | Mortality (Respiratory) | | | | | | | | | SOMO35 | | | |
| (Eskandari, Maleki et al. 2020) | Iran | 5 | Dezful | $PM_{2.5}$ | AQMS | NR | N | NR | Mortality (All, COPD, IHD, LC, stroke, ALRI), Hospital (CVD, Respiratory) | AirQ+ default | Ministry of Health | National | LT/ST | Y | N | Y | N | NR | Research, Impact of pollution episodes (Dust storm) | N | N |
| (Hadei, Shahsavani et al. 2020) | Iran | 5 | Tehran, Mashhad, Isfahan, Karaj, Shiraz, Tabriz, Ghom, Ahvaz, Kermanshah, Urmia, Rasht, Kerman, Yazd, Hamadan, Arak, Ghazvin, Sanandaj, Khoram Abad, Shahre Kord, Bushehr, Bojnourd, Birjand, Ilam, Yasuj, and Zabol | $PM_{2.5}$ | AQMS | 1,2,3 | Y | Zero and negatives | Mortality (All, COPD, LC, IHD, stroke) | AirQ+ default | Ministry of Health | National | LT/ST | Y | Y | Y | N | 10, 25 | Research, Impact of pollution episodes (Dust storm) | N | Y |

| ID/ Author date | Country of study | WHO regions (AFR:1, AMR:2, SEAR: 3, EUR:4, EMR:5, WPR:6) | Name of city | Air quality | | | | | Health endpoints | RR Sources | BI Sources | BI Sources (Categorized) | Type of exposure (Long-term (LT)/ Short-term (ST)) | Number of cases reported (Yes/No) | PAF reported (Yes/No) | CI reported (Yes/No) | Sensitivity analyses (Yes/No) | Cut-off value (μg/m³) to estimate health effects | Motivation or context of study | Population Size reported (Yes/No) | use of life-tables module |
|---|---|---|---|---|---|---|---|---|---|---|---|---|---|---|---|---|---|---|---|---|---|
| | | | | Air Pollutants (APs) | AQMS or self-measured - Modeling - satellite | Type of montoring stations (Res:1/TR:2/BG:3/Ind:4/ None:5, Commercial:6) | Data Coverage reported (Yes/No) | Air quality data processing and validation reported (Yes/No) | | | | | | | | | | | | | |
| (Hajizadeh, Jafari et al. 2020) | Iran | 5 | Isfahan | $PM_{2.5}$ | AQMS | NR | | | Mortality (All, COPD, LC, IHD, stroke) | AirQ+ default | Ministry of Health | National | LT | Y | Y | Y | N | 10 | Research | N | N |
| (Hwang, Lin et al. 2020) | Taiwan | 6 | North (four districts, namely Keelung, Taipei, New Taipei, and Taoyuan), Chu-Miao (two districts, namely Hsinchu and Miaoli), Central (three districts, namely Taichung, Jhanghua, and Nantou), Yun-Chia-Nan (three districts, namely Yunlin, Chiayi, and Tainan), Kao-Ping (two districts, namely Kaohsiung and Pingdong), Yilan (one district, namely Yilan), and Hua-Tung regions (two districts, namely | $PM_{2.5}$ | AQMS | 1 | N | NR | Mortality (COPD, LC, IHD, stroke) | AirQ+ default | National Death Registry | National | LT | Y | Y | Y | N | NR | Research | N | N |

| ID/ Author date | Country of study | WHO regions (AFR:1, AMR:2, SEAR: 3, EUR:4, EMR:5, WPR:6) | Name of city | Air quality | | | | | Health endpoints | RR Sources | BI Sources | BI Sources (Categorized) | Type of exposure (Long-term (LT)/ Short-term (ST)) | Number of cases reported (Yes/No) | PAF reported (Yes/No) | CI reported (Yes/No) | Sensitivity analyses (Yes/No) | Cut-off value (μg/m$^3$) to estimate health effects | Motivation or context of study | Population Size reported (Yes/No) | use of life-tables module |
|---|---|---|---|---|---|---|---|---|---|---|---|---|---|---|---|---|---|---|---|---|---|
| | | | | Air Pollutants (APs) | AQMS or self-measured - Modeling - satellite | Type of montoring stations (Res:1/TR:2/BG:3/Ind:4/ None:5, Commercial:6) | Data Coverage reported (Yes/No) | Air quality data processing and validation reported (Yes/No) | | | | | | | | | | | | | |
| | | | Hualian and Taidong) | | | | | | | | | | | | | | | | | | |
| (kermani, Arfaeinia et al. 2020) | Iran | 5 | Karaj | $PM_{2.5}$ | AQMS | NR | N | Removing invalid concentrations | Mortality (All, COPD, LC, IHD, stroke, ALRI) | AirQ+ default | Ministry of Health, Office of Civil Registration | National | LT | Y | N | Y | N | 10 | Research | N | N |

| ID/ Author date | Country of study | WHO regions (AFR:1, AMR:2, SEAR: 3, EUR:4, EMR:5, WPR:6) | Name of city | Air quality | | | | | Health endpoints | RR Sources | BI Sources | BI Sources (Categorized) | Type of exposure (Long-term (LT)/ Short-term (ST)) | Number of cases reported (Yes/No) | PAF reported (Yes/No) | CI reported (Yes/No) | Sensitivity analyses (Yes/No) | Cut-off value (μg/m$^3$) to estimate health effects | Motivation or context of study | Population Size reported (Yes/No) | use of life-tables module |
|---|---|---|---|---|---|---|---|---|---|---|---|---|---|---|---|---|---|---|---|---|---|
| | | | | Air Pollutants (APs) | AQMS or self-measured - Modeling - satellite | Type of montoring stations (Res:1/TR:2/BG:3/Ind:4/ None:5, Commercial:6) | Data Coverage reported (Yes/No) | Air quality data processing and validation reported (Yes/No) | | | | | | | | | | | | | |
| (Li, Zhao et al. 2020) | China | 6 | Across the mainland China | $PM_{2.5}$ | AQMS | NR | N | NR | Mortality (All, COPD, LC, IHD, stroke) | AirQ+ default | GBD database | International | LT | Y | N | Y | Y | 10, 15, 25, 35, and the partial reduction in PM2.5 concentration that is needed to achieve a certain percentage reduction in mortality | Research, Strategy/policy planning | N | N |
| (Malakootian and Mohammadi 2020) | Iran | 5 | Kerman | $PM_{2.5}$ | AQMS | NR | Y | NR | Mortality (All, COPD, IHD, stroke) | AirQ+ default | Kerman University of Medical Sciences | Local | LT | Y | Y | Y | N | NR | Research | Y | N |
| | | | | $NO_2$ | | | | | Mortality (All) | | | | | | | | | NR | | | |
| | | | | $O_3$ | | | | | Mortality (All, CVD, Respiratory) | | | | | | | | | SOMO35 | | | |
| (Manojkumar, Manish Kumar et al. 2020) | India | 3 | Hyderabad | $PM_{2.5}$ | AQMS | NR | N | NR | Hospital (COPD) | NR | Previous studies | Previous studies | ST | Y | Y | Y | N | 10 | Research | Y | N |
| (Matkovic, Mulić et al. 2020) | Bosnia and Herzegovina | 4 | tuzla and lukavac | $PM_{2.5}$ | AQMS | 1,4 | Y | Removing negatives and missings | Mortality (All) | AirQ+ default | Local agencies | Local | ST | Y | N | Y | N | 10, 25 | Research, Strategy/policy planning | N | Y |
| (Oliveira, Delerue-Matos et al. 2020) | Portugal | 4 | Five rural monitoring in the center of Portugal (Coimbra, Leiria, Viseu, Castelo Branco, and Santarém) | $PM_{2.5}$ | AQMS | 3 | N | NR | Mortality (All), Hospital (CVD) | AirQ+ default | Pordata database | National | ST | Y | Y | Y | N | NR | Research, Impact of pollution episodes | N | N |
| | | | | $PM_{10}$ | | | | | Incidence of asthma | | | | | | | | | | | | |
| (Pahrol, Mahiyuddin et al. 2020) | Malaysia | 6 | 13 states and 3 federal territories | PM2.5 | Satellite | NR | N | NR | Mortality (All) | AirQ+ default | Department of Statistics | National | LT | N | N | Y | N | 10 | Research | N | N |

| ID/ Author date | Country of study | WHO regions (AFR:1, AMR:2, SEAR: 3, EUR:4, EMR:5, WPR:6) | Name of city | Air quality | | | | | Health endpoints | RR Sources | BI Sources | BI Sources (Categorized) | Type of exposure (Long-term (LT)/ Short-term (ST)) | Number of cases reported (Yes/No) | PAF reported (Yes/No) | CI reported (Yes/No) | Sensitivity analyses (Yes/No) | Cut-off value (µg/m³) to estimate health effects | Motivation or context of study | Population Size reported (Yes/No) | use of life-tables module |
|---|---|---|---|---|---|---|---|---|---|---|---|---|---|---|---|---|---|---|---|---|---|
| | | | | Air Pollutants (APs) | AQMS or self-measured - Modeling - satellite | Type of montoring stations (Res:1/TR:2/BG:3/Ind:4/ None:5, Commercial:6) | Data Coverage reported (Yes/No) | Air quality data processing and validation reported (Yes/No) | | | | | | | | | | | | | |
| (Rovira, Domingo et al. 2020) | Spain | 4 | Reus and Tarragona | $PM_{2.5}$ | AQMS | 1,4 | N | NR | Mortality (All, COPD, LC, IHD, stroke) | AirQ+ default | Catalan Health System Observatory and Health Department of Catalonia | Local | LT | Y | Y | Y | N | 10 | Research | N | Y |
| | | | | $NO_2$ | | | | | Mortality (All) | | | | | | | | | 10 | | | |
| | | | | $O_3$ | | | | | Mortality (All) | | | | | | | | | 100 | | | |
| (Sacks, Fann et al. 2020) | Hungary | 4 | Budapest | $PM_{2.5}$ | AQMS | NR | N | NR | Mortality (All) | AirQ+ default | Previous studies | Previous studies | LT | Y | Y | Y | Y | 5, 10, 12, 25 | Research, strategy/policy planning | N | N |
| (Al-Hemoud, Gasana et al. 2021) | Kuwait | 5 | All over the Kuwait | $O_3$ | AQMS | NR | Y | NR | Mortality (All, CVD, Respiratory), Hospital (CVD, Respiratory) | AirQ+ default | National Center for Health Information | National | LT/ST | Y | Y | Y | N | SOMO35 | Research | N | N |
| | | | | $NO_2$ | | | | | Mortality (All), Hospital (Respiratory) | | | | | | | | | SOMO35 | | | |
| (Amoatey, Khaniabadi et al. 2021) | Iran | 5 | Arak | $PM_{2.5}$ | AQMS | NR | N | NR | Restricted activity days, Work days lost | AirQ+ default | NR | NR | ST | Y | Y | Y | N | 10 | Research | N | N |
| | | | | $PM_{10}$ | | | | | incidence of chronic bronchitis and prevalence of bronchitis | | | | | | | | | 10 | | | |
| (Balidemaj, Isaxon et al. 2021) | Ethiopia | 1 | Adama | Household | Self-measured | NR | N | NR | DALY (COPD, LC, IHD, stroke, ALRI), Mortality (All) | AirQ+ default | WHO | International | LT | Y | Y | Y | N | NR | Research | Y | Y |
| (Chandra, Nisa et al. 2021) | Indonesia | 3 | Bandung | $PM_{2.5}$ | Self-measured | NR | N | NR | Mortality (COPD, LC, IHD, stroke, ALRI), Hospital (CVD, Respiratory) | AirQ+ default | Hospitals data | Local | LT/ST | Y | Y | Y | N | NR | Research | N | N |

| ID/ Author date | Country of study | WHO regions (AFR:1, AMR:2, SEAR: 3, EUR:4, EMR:5, WPR:6) | Name of city | Air quality | | | | | Health endpoints | RR Sources | BI Sources | BI Sources (Categorized) | Type of exposure (Long-term (LT)/ Short-term (ST)) | Number of cases reported (Yes/No) | PAF reported (Yes/No) | CI reported (Yes/No) | Sensitivity analyses (Yes/No) | Cut-off value (μg/m$^3$) to estimate health effects | Motivation or context of study | Population Size reported (Yes/No) | use of life-tables module |
|---|---|---|---|---|---|---|---|---|---|---|---|---|---|---|---|---|---|---|---|---|---|
| | | | | Air Pollutants (APs) | AQMS or self-measured - Modeling - satellite | Type of montoring stations (Res:1/TR:2/BG:3/Ind:4/ None:5, Commercial:6) | Data Coverage reported (Yes/No) | Air quality data processing and validation reported (Yes/No) | | | | | | | | | | | | | |
| | | | | $PM_{10}$ | | | | | Incidence of chronic bronchitis, Infant mortality, prevalence of bronchitis, Asthmatic symptoms | | | | | | | | | NR | | | |
| (Dragic, Bijelovic et al. 2021) | Serbia | 4 | Novi Sad | $PM_{2.5}$ | AQMS | 1,2,3,4 | Y | NR | Mortality (All) | AirQ+ default | Institute of Public Health of Vojvodina | Local | ST | Y | Y | Y | N | 0 | Research, Impact of COVID era | N | N |
| (Ekland, Olsson et al. 2021) | Iran | 5 | Yasuj | $PM_{10}$ | AQMS | NR | N | Negative and zero | Incidence of chronic bronchitis, Infant mortality, prevalence of bronchitis | AirQ+ default | Previous studies | Previous studies | LT | Y | Y | Y | N | 20 | Research | N | N |
| (Goel, Saxena et al. 2021) | India | 3 | Agra, Kanpur, Lucknow, and Varanasi, and the Delhi-NCR | $PM_{2.5}$ | AQMS | NR | Y | NR | Mortality (All) | AirQ+ default | official census by the Government of India | National | ST | Y | N | Y | Y | NR | Research, Impact of COVID era | N | N |
| (Goudarzi, Hopke et al. 2021) | Iran | 5 | Ahvaz | $PM_{2.5}$ | AQMS | NR | Y | Negative and zero | Mortality (All, COPD, LC, IHD, stroke, ALRI) | AirQ+ default | Previous studies | Previous studies | LT | Y | Y | Y | N | 10 | Research | N | N |
| (Hajizadeh, Jafari et al. 2021) | Iran | 5 | Isfahan | $PM_{2.5}$ | AQMS | NR | N | NR | Mortality (All, COPD, LC, IHD, stroke) | AirQ+ default | Ministry of Health | National | LT/ST | Y | Y | Y | N | 10 | Research | N | N |
| | | | | $O_3$ | | | | | Mortality (Respiratory), Mortality (All) | | | | | | | | | SOMO35 | | | |
| (Kanhai, Fobil et al. 2021) | Ghana | 1 | Accra | $PM_{2.5}$ | Modelling (emission from waste management) | NR | N | NR | Mortality (All) | AirQ+ default | Ghana Statistical Service | National | LT | Y | N | Y | N | NR | Research, strategy/policy planning | N | N |

| ID/ Author date | Country of study | WHO regions (AFR:1, AMR:2, SEAR: 3, EUR:4, EMR:5, WPR:6) | Name of city | Air quality | | | | | Health endpoints | RR Sources | BI Sources | BI Sources (Categorized) | Type of exposure (Long-term (LT)/ Short-term (ST)) | Number of cases reported (Yes/No) | PAF reported (Yes/No) | CI reported (Yes/No) | Sensitivity analyses (Yes/No) | Cut-off value (μg/m³) to estimate health effects | Motivation or context of study | Population Size reported (Yes/No) | use of life-tables module |
|---|---|---|---|---|---|---|---|---|---|---|---|---|---|---|---|---|---|---|---|---|---|
| | | | | Air Pollutants (APs) | AQMS or self-measured - Modeling - satellite | Type of montoring stations (Res:1/TR:2/BG:3/Ind:4/ None:5, Commercial:6) | Data Coverage reported (Yes/No) | Air quality data processing and validation reported (Yes/No) | | | | | | | | | | | | | |
| (Kumie, Worku et al. 2021) | Ethiopia | 1 | Addis Ababa | $PM_{2.5}$ | Self-measured | NR | Y | NR | Mortality (All) | AirQ+ default | Addis Ababa Mortality Surveillance Program | National | LT | Y | Y | Y | N | 10,15, 25, 35 | Research, strategy/policy planning | N | N |
| (Manojkumar and Srimuruganandam 2021) | India | 3 | Chennai, Delhi, Faridabad, Gurgaon, Hyderabad, Kanpur, Lucknow, Nagpur, Thane, and Varanasi | $PM_{2.5}$ | AQMS | NR | Y | NR | Mortality (All, COPD, LC, IHD, stroke, ALRI), Hospital (CVD, Respiratory) | AirQ+ default | Previous studies | Previous studies | LT | Y | N | Y | N | 2.4, 5.9, 10 | Research | N | N |
| (Markandeya, Verma et al. 2021) | India | 3 | Lucknow | $PM_{2.5}$ | AQMS | NR | N | NR | Mortality (All) | AirQ+ default | NR | NR | LT | N | Y | Y | N | 40, 50 | Research, Impact of COVID era | N | N |
| (Mirzaei, Tahriri et al. 2021) | Iran | 5 | Tehran | $PM_{2.5}$ | AQMS | NR | Y | NR | Mortality (All, IHD, LC) | AirQ+ default | Cemetery of the city (Behesht Zahra) | Local | LT | Y | N | Y | N | 10 | Research | N | N |
| (Moradi, Hadei et al. 2021) | Iran | 5 | Ahvaz | $PM_{2.5}$ | AQMS | NR | Y | Negative and zero | Mortality (All) | AirQ+ default | Ministry of Health | National | LT | Y | Y | Y | N | 10 | Research | Y | Y |
| (Naghan, Neisi et al. 2021) | Iran | 5 | Ahvaz | $PM_{2.5}$ | AQMS | NR | Y | NR | Mortality (All, COPD, LC, IHD, stroke, ALRI) | AirQ+ default | Ministry of Health | National | LT | Y | Y | Y | N | 10 | Research | N | N |
| | | | | $NO_2$ | | | | | Mortality (All) | | | | | | | | | 40 | | | |
| | | | | $O_3$ | | | | | Mortality (Respiratory) | | | | | | | | | 100 | | | |
| (Nguyen, Nghiem et al. 2021) | Vietnam | 6 | Hanoi | $PM_{10}$ | Modelling (based on emission factor) | NR | N | NR | Incidence of chronic bronchitis, prevalence of bronchitis | AirQ+ default | NR | NR | LT | Y | Y | Y | N | NR | Research, strategy/policy planning | N | N |
| | | | | $NO_2$ | | | | | Mortality (All), prevalence of bronchitis symptoms | | | | | | | | | NR | | | |

| ID/ Author date | Country of study | WHO regions (AFR:1, AMR:2, SEAR: 3, EUR:4, EMR:5, WPR:6) | Name of city | Air quality | | | | | Health endpoints | RR Sources | BI Sources | BI Sources (Categorized) | Type of exposure (Long-term (LT)/ Short-term (ST)) | Number of cases reported (Yes/No) | PAF reported (Yes/No) | CI reported (Yes/No) | Sensitivity analyses (Yes/No) | Cut-off value (µg/m³) to estimate health effects | Motivation or context of study | Population Size reported (Yes/No) | use of life-tables module |
|---|---|---|---|---|---|---|---|---|---|---|---|---|---|---|---|---|---|---|---|---|---|
| | | | | Air Pollutants (APs) | AQMS or self-measured - Modeling - satellite | Type of montoring stations (Res:1/TR:2/BG:3/Ind:4/ None:5, Commercial:6) | Data Coverage reported (Yes/No) | Air quality data processing and validation reported (Yes/No) | | | | | | | | | | | | | |
| (Pala, Aykac et al. 2021) | Turkey | 4 | All over Terkey | $PM_{2.5}$ | AQMS | NR | Y | NR | Mortality (All) | AirQ+ default | Classification of Statistical Region Units, Deaths by Gender and Age Group | National | LT | Y | N | Y | N | 10 | Research | Y | N |
| (Palanbek Yavaş, Önal et al. 2021) | Turkey | 4 | İstanbul, Kocaeli, Edirne, Tekirdağ, Kırklareli, Sakarya, Bilecik, Bursa, Balıkesir, Çanakkale and Yalova | $PM_{10}$ | AQMS | NR | N | NR | Incidence of chronic bronchitis | AirQ+ default | National Burden of Disease of Turkey | National | LT | Y | N | Y | N | 20 | Research | N | N |
| | | | | $NO_2$ | | | | | Mortality (All) | | TURKSTAT Death Statistics | | | | | | | 10 | | | |
| (Passi, Shiva Nagendra et al. 2021) | India | 3 | Chennai | $PM_{2.5}$ | Self-measured | NR | N | NR | Mortality (All, COPD, LC, IHD, stroke, ALRI), Hospital (CVD, Res), Restricted activity days, work days lost | AirQ+ default | Previous studies | Previous studies | LT | Y | Y | Y | N | 25 | Research | Y | N |
| | | | | $PM_{10}$ | | | | | Incidence asthma symptoms | | | | | | | | | 50 | | | |
| (Saeedi, Khani Jazani et al. 2021) | Iran | 5 | Tehran | $PM_{2.5}$ | Self-measured | NR | N | NR | Mortality (All, COPD, IHD, stroke) | AirQ+ default | GBD database | International | LT | Y | N | Y | N | NR | Research | N | N |
| (Soleimani, Akbari et al. 2021) | Iran | 5 | Isfahan | $PM_{2.5}$ | AQMS data and Modelling | NR | N | NR | Mortality (All, COPD, LC, IHD) | AirQ+ default | Isfahan University of Medical Sciences | Local | LT | Y | Y | Y | N | 10 | Research | N | Y |
| (Varol, Tokuc et al. 2021) | Turkey | 4 | Süleymanpaşa | $PM_{2.5}$ | AQMS | 1,2 | Y | NR | Mortality (All) | AirQ+ default | Ministry of Health | National | LT | Y | N | Y | N | 10 | Research | N | N |

| ID/ Author date | Country of study | WHO regions (AFR:1, AMR:2, SEAR:3, EUR:4, EMR:5, WPR:6) | Name of city | Air quality | | | | | Health endpoints | RR Sources | BI Sources | BI Sources (Categorized) | Type of exposure (Long-term (LT)/ Short-term (ST)) | Number of cases reported (Yes/No) | PAF reported (Yes/No) | CI reported (Yes/No) | Sensitivity analyses (Yes/No) | Cut-off value (µg/m³) to estimate health effects | Motivation or context of study | Population Size reported (Yes/No) | use of life-tables module |
|---|---|---|---|---|---|---|---|---|---|---|---|---|---|---|---|---|---|---|---|---|---|
| | | | | Air Pollutants (APs) | AQMS or self-measured - Modeling - satellite | Type of montoring stations (Res:1/TR:2/BG:3/Ind:4/ None:5, Commercial:6) | Data Coverage reported (Yes/No) | Air quality data processing and validation reported (Yes/No) | | | | | | | | | | | | | |
| (Zallaghi, Goudarzi et al. 2021) | Iran | 5 | Ahvaz | $PM_{2.5}$ | AQMS | NR | Y | NR | Mortality (All) ELR, YLL | AirQ+ default | Department of Health | National | LT | Y | N | Y | N | 10 | Research | N | Y |
| (Brito, Bernardo et al. 2022) | Portugal | 4 | Various municipalities | $PM_{2.5}$ | AQMS | NR | Y | NR | Mortality (All) | AirQ+ default | PORDATA | National | LT | Y | Y | Y | N | 10 | Research | N | N |
| | | | | $NO_2$ | | | | | Mortality (All) | | | | | | | | | 10 | | | |
| | | | | $O_3$ | | | | | Mortality (All) | | | | | | | | | SOMO35 | | | |
| (Kahraman and Sivri 2022) | Turkey | 4 | Balıkesir, Bursa, Istanbul, Kocaeli, Sakarya and Tekirdağ | $PM_{2.5}$ | AQMS | NR | Y | NR | Mortality (All) | AirQ+ default | Turkish Statistical Institute | National | LT | Y | Y | Y | N | 10 | Research | N | N |
| (Kliengchuay, Srimanus et al. 2022) | Thailand | 3 | Ratchaburi | $PM_{2.5}$ | AQMS | NR | N | NR | Mortality (All, COPD, IHD, stroke) | AirQ+ default | Ministry of Public Health | National | LT | Y | Y | Y | N | 10 | Research | N | Y |
| (Kuldeep, Sen et al. 2022) | India | 3 | Alwar | $PM_{2.5}$ | AQMS | NR | N | NR | Mortality (All, COPD, LC, IHD, Stroke, ALRI) | AirQ+ default | NR | NR | LT/ST | Y | Y | Y | N | NR | Research | N | N |
| | | | | $PM_{2.5}$ | | | | | Mortality (All), Hospital (CVD, Respiratory) | | | | | | | | | NR | | | |
| | | | | $PM_{10}$ | | | | | Post neonatal infant Mortality, Incidence of chronic Bronchitis in adults, Prevalence of Bronchitis in children, Incidence of Asthma symptoms in asthmatic children | | | | | | | | | NR | | | |

| ID/ Author date | Country of study | WHO regions (AFR:1, AMR:2, SEAR: 3, EUR:4, EMR:5, WPR:6) | Name of city | Air quality | | | | | Health endpoints | RR Sources | BI Sources | BI Sources (Categorized) | Type of exposure (Long-term (LT)/ Short-term (ST)) | Number of cases reported (Yes/No) | PAF reported (Yes/No) | CI reported (Yes/No) | Sensitivity analyses (Yes/No) | Cut-off value (µg/m³) to estimate health effects | Motivation or context of study | Population Size reported (Yes/No) | use of life-tables module |
|---|---|---|---|---|---|---|---|---|---|---|---|---|---|---|---|---|---|---|---|---|---|
| | | | | Air Pollutants (APs) | AQMS or self-measured - Modeling - satellite | Type of montoring stations (Res:1/TR:2/BG:3/Ind:4/ None:5, Commercial:6) | Data Coverage reported (Yes/No) | Air quality data processing and validation reported (Yes/No) | | | | | | | | | | | | | |
| (Kuldeep, Sisodiya et al. 2022) | India | 3 | Kota | $PM_{2.5}$ | AQMS | NR | N | NR | Mortality (All, COPD, LC, IHD, Stroke, ALRI), Hospital (CVD, Respiratory) | AirQ+ default | NR | NR | LT/ST | Y | Y | Y | N | 10 | Research | N | N |
| | | | | $PM_{10}$ | | | | | Post neonatal infant Mortality, Incidence of chronic Bronchitis in adults, Prevalence of Bronchitis in children, Incidence of Asthma symptoms in asthmatic children | | | | | | | | | 20 | | | |
| (Kumar and Middey 2022) | India | 3 | Nagpur | $PM_{2.5}$ | AQMS data and Modelling | NR | N | NR | Mortality (COPD) | AirQ+ default | NR | NR | LT | N | N | Y | N | NR | Research | N | N |
| (Lam, Chang et al. 2022) | India, Indonesia, China, Vietnamn Sri Lanka, Peru, Kosovo, Ethiopia, UAE, USA, UK, Japan, Switzerland | 1,2,3,4, 5,6 | New Delhi; Jakarta; Beijing; Hanoi; Colombo; Lima; Pristina; Addis Ababa; Abu Dhabi; London; Los Angeles; New York; Hong Kong; Tokyo; Zurich | $PM_{2.5}$ | AQMS | 1,2 | N | NR | Mortality (All) | AirQ+ default | NR | NR | ST | Y | Y | Y | N | 25 | Research, Impact of COVID era | N | N |

| ID/ Author date | Country of study | WHO regions (AFR:1, AMR:2, SEAR:3, EUR:4, EMR:5, WPR:6) | Name of city | Air quality | | | | | Health endpoints | RR Sources | BI Sources | BI Sources (Categorized) | Type of exposure (Long-term (LT)/ Short-term (ST)) | Number of cases reported (Yes/No) | PAF reported (Yes/No) | CI reported (Yes/No) | Sensitivity analyses (Yes/No) | Cut-off value (μg/m$^3$) to estimate health effects | Motivation or context of study | Population Size reported (Yes/No) | use of life-tables module |
|---|---|---|---|---|---|---|---|---|---|---|---|---|---|---|---|---|---|---|---|---|---|
| | | | | Air Pollutants (APs) | AQMS or self-measured - Modeling - satellite | Type of montoring stations (Res:1/TR:2/BG:3/Ind:4/ None:5, Commercial:6) | Data Coverage reported (Yes/No) | Air quality data processing and validation reported (Yes/No) | | | | | | | | | | | | | |
| (Manoj, Satheesh Kumar et al. 2022) | India | 3 | Delhi | $O_3$ | AQMS | NR | N | NR | Mortality (All) | AirQ+ default | Delhi State Government | Local | ST | Y | N | Y | N | SOMO35 | Research, Impact of COVID era | N | N |
| (Manojkumar and Srimuruganandam 2022) | India | 3 | Vellore | $PM_{2.5}$ | Satellite | NR | N | NR | Mortality (All, COPD, IHD, stroke) | Previous studies | Institute for Health Metrics and Evaluation | International | LT | Y | N | Y | Y | 10 | Research | N | N |
| (Moradi, Mokhtari et al. 2022) | Iran | 5 | Ardabil | $PM_{2.5}$ | AQMS | NR | Y | Removing zero, negative, and hourly abnormal concentrations (repetitive patterns such as exactly the same concentrations in a row) | Mortality (All, COPD, IHD, stroke, ALRI) | AirQ+ default | Deputy of Treatment and Statistics Unit of Ardabil hospitals | Local | LT/ST | Y | N | Y | N | 10 | Research | N | N |
| | | | Ardabil | $PM_{2.5}$ | | | | | Hospital (CVD, Respiratory) | | | | | | | | | 25 | | | |
| (Naghan, Neisi et al. 2022) | Iran | 5 | Shahrekord | $PM_{2.5}$ | AQMS | NR | Y | NR | Mortality (All, COPD, LC, IHD, stroke, ALRI) | AirQ+ default | Ministry of Health | National | LT | Y | Y | Y | N | 10 | Research | N | N |
| | | | | $NO_2$ | | | | | Mortality (All) | | | | | | | | | 40 | | | |
| | | | | $O_3$ | | | | | Mortality (Respiratory) | | | | | | | | | 100 | | | |
| (Nasir, Nawaz et al. 2022) | Pakistan | 5 | Kalar Kahar | $PM_{2.5}$ | Self-measured, modelled | NR | N | NR | Mortality (All, LC) | AirQ+ default | bureau of statistics, research works, the health department of Punjab, and World Bank | National/International | LT | Y | Y | Y | N | NR | Research | N | N |
| | | | | $PM_{10}$ | | | | | Post neonatal infant mortality | | | | | | | | | 10 | | | |
| | | | | $NO_2$ | | | | | Mortality (All) | | | | | | | | | 10 | | | |
| | | | | $O_3$ | | | | | Mortality (Respiratory) | | | | | | | | | SOMO35 | | | |

| ID/ Author date | Country of study | WHO regions (AFR:1, AMR:2, SEAR: 3, EUR:4, EMR:5, WPR:6) | Name of city | Air quality | | | | | Health endpoints | RR Sources | BI Sources | BI Sources (Categorized) | Type of exposure (Long-term (LT)/ Short-term (ST)) | Number of cases reported (Yes/No) | PAF reported (Yes/No) | CI reported (Yes/No) | Sensitivity analyses (Yes/No) | Cut-off value (µg/m$^3$) to estimate health effects | Motivation or context of study | Population Size reported (Yes/No) | use of life-tables module |
|---|---|---|---|---|---|---|---|---|---|---|---|---|---|---|---|---|---|---|---|---|---|
| | | | | Air Pollutants (APs) | AQMS or self-measured - Modeling - satellite | Type of montoring stations (Res:1/TR:2/BG:3/Ind:4/ None:5, Commercial:6) | Data Coverage reported (Yes/No) | Air quality data processing and validation reported (Yes/No) | | | | | | | | | | | | | |
| (Orru, Olstrup et al. 2022) | Finland, Norway, and Denmark | 4 | Helsinki Metropolitan Area, Oslo, and Copenhagen, and Umeå | $PM_{2.5}$ | Modelling (emission from residential wood combustion) | NR | N | NR | Mortality (All) | Previous studies | Statistics Sweden, Statistics Finland, Statistics Norway, and Statistics Denmark | National | LT | Y | N | Y | N | NR | Research, strategy/policy planning | N | N |
| (Prabhu, Singh et al. 2022) | India | 3 | Bengaluru | $PM_{2.5}$ | AQMS, self-measured | NR | N | NR | Mortality (All, COPD, LC, IHD, stroke, ALRI) | AirQ+ default | GBD | International | LT | Y | N | Y | N | 2.4 | Research | N | N |
| (Sasmita, Kumar et al. 2022) | India | 3 | Bhubaneswar | $PM_{10}$ | Self-measured | NR | N | NR | Incidence of chronic bronchitis in adults ( > = 30 years of age), post neonatal infant (0–1 year) mortality and, prevalence of bronchitis in children | AirQ+ default | Previous studies | Previous studies | LT | Y | Y | Y | N | NR | Research, impact of pollution peaks | N | N |
| (Ravindra, Singh et al. 2022) | India | 5 | Khera | $PM_{2.5}$ | Self-measured | NR | N | NR | Mortality (All) | AirQ+ default | Civil Hospital | Local | ST | Y | N | Y | N | 10, 40 | Research | N | N |
| (Soleimani, Akbari et al. 2022) | Iran | 5 | Isfahan | $PM_{2.5}$ | AQMS data and Modelling | NR | Y | NR | Mortality (All, COPD, LC, IHD) | AirQ+ default | Isfahan University of Medical Sciences | Local | LT | Y | Y | Y | N | 10 | Research | Y | Y |
| (Tabibzadeh, Hosseini et al. 2022) | Iran | 5 | Shiraz | $PM_{10}$ | Self-measured | NR | N | NR | Mortality (COPD, LC, Heart HID, stroke) | AirQ+ default | WHO | International | ST | Y | Y | Y | N | NR | Research | N | N |

| ID/ Author date | Country of study | WHO regions (AFR:1, AMR:2, SEAR: 3, EUR:4, EMR:5, WPR:6) | Name of city | Air quality | | | | | Health endpoints | RR Sources | BI Sources | BI Sources (Categorized) | Type of exposure (Long-term (LT)/ Short-term (ST)) | Number of cases reported (Yes/No) | PAF reported (Yes/No) | CI reported (Yes/No) | Sensitivity analyses (Yes/No) | Cut-off value (µg/m³) to estimate health effects | Motivation or context of study | Population Size reported (Yes/No) | use of life-table module |
|---|---|---|---|---|---|---|---|---|---|---|---|---|---|---|---|---|---|---|---|---|---|
| | | | | Air Pollutants (APs) | AQMS or self-measured - Modeling - satellite | Type of montoring stations (Res:1/TR:2/BG:3/Ind:4/ None:5, Commercial:6) | Data Coverage reported (Yes/No) | Air quality data processing and validation reported (Yes/No) | | | | | | | | | | | | | |
| (Xu, Zhang et al. 2022) | China | 6 | Jiangsu province (Nanjing (NJ), Zhenjiang (ZJ), Changzhou (CZ), Wuxi (WX), Suzhou (SZ), Yangzhou (YZ), Taizhou (TZ), Nantong (NT), Yancheng (YC), Lianyungang (LYG), Xuzhou (XZ), Suqian (SQ) and Huaian (HA)) | $PM_{2.5}$, $PM_{10}$, $SO_2$, $NO_2$, CO and O3 | AQMS | NR | N | NR | Mortality (All, Respiratory and Cardiovascular) | NR | NR | NR | NR | Y | Y | Y | N | the upper threshold values of Chinese Ambient Air Quality Standard (CAAQS) Grade II standard (GB 3095-2012) were used as C0 | Research | N | N |
| (Namrata and Drashti 2022) | India | 3 | Surat | $O_3$ | AQMS | NR | Y | NR | Mortality (All, Respiratory, Cardiovascular), Hospital Admissions (Cardiovascular Disease (HACVD), and Respiratory Disease (HARD)) | NR | NR | NR | LT/ST | Y | Y | Y | N | NR | Research | N | N |
| (Begou 2023) | Greece | 4 | Attica Region | $PM_{2.5}$ | AQMS | NR | N | NR | Mortality (All, | AirQ+ default | NR | NR | LT | Y | Y | Y | N | NR | Research | N | N |

| ID/ Author date | Country of study | WHO regions (AFR:1, AMR:2, SEAR: 3, EUR:4, EMR:5, WPR:6) | Name of city | Air quality | | | | | Health endpoints | RR Sources | BI Sources | BI Sources (Categorized) | Type of exposure (Long-term (LT)/ Short-term (ST)) | Number of cases reported (Yes/No) | PAF reported (Yes/No) | CI reported (Yes/No) | Sensitivity analyses (Yes/No) | Cut-off value (µg/m³) to estimate health effects | Motivation or context of study | Population Size reported (Yes/No) | use of life-tables module |
|---|---|---|---|---|---|---|---|---|---|---|---|---|---|---|---|---|---|---|---|---|---|
| | | | | Air Pollutants (APs) | AQMS or self-measured - Modeling - satellite | Type of montoring stations (Res:1/TR:2/BG:3/Ind:4/ None:5, Commercial:6) | Data Coverage reported (Yes/No) | Air quality data processing and validation reported (Yes/No) | | | | | | | | | | | | | |
| | | | | | | | | | COPD, IHD, LC, Stroke) | | | | | | | | | | | | |
| (Mohd Shafie, Mahmud et al. 2022) | Malaysia | 6 | Klang Valley | $PM_{10}$ | AQMS | NR | N | NR | Incidence of chronic bronchitis | NR | NR | NR | LT | Y | N | Y | N | NR | Research | N | N |
| (Mohammadi, Azhdarpoor et al. 2016) | Iran | 5 | Yasuj | $PM_{2.5}$, $NO_2$, $O_3$ | AQMS | NR | Y | Zero and negatives | Mortality (All, ALRI, LC, IHD, COPD) | AirQ+ default | Local data | Local | LT/ST | Y | Y | Y | N | NR | Research | N | N |
| (Organization) 2019) | Serbia | 4 | Belgrade, Obrenovac, Lazarevac, Novi Sad, Beočin, Smederevo, Kragujevac, Užice, Kosjerić, Niš, Valjevo | PM2.5 | AQMS | NR | N | NR | Mortality, YLL and DALY (All, ALRI, LC, IHD,COPD, Stroke) | AirQ+ default | National | National | LT | Y | Y | Y | N | per 10 ug/m3 increses in , for YLL per 5, 10, 20 ug/m3 increase | Research | Y | N |
| (ÖZTÜRK and Öztürk 2023) | Turkey | 4 | İstanbul, Ankara, İzmir, and Bursa | NO2 | AQMS | 5 | N | NR | Mortality | | | | NR | N | | N | | per 20 ug/m3 increase | Research | N | N |
| (KAVUNCUOĞLU, YILMAZ et al. 2021) | Turkey | 4 | Erzurum | NO2 | AQMS | 5 | N | NR | Mortality | | | | NR | Y | | N | | NR | Research | N | N |
| (ULUTAŞ 2022) | Turkey | 4 | Sakarya | NO2 | AQMS | 5 | N | NR | Mortality | NR | NR | NR | NR | N | Y | Y | N | per 20, 10 ug/m3 increase | Research | N | N |
| (中井里史, 丸山隆太 et al. 2022) | Japan | 3 | Yokohama | NO2 | AQMS + LUR modeling | 5 | N | NR | Mortality | | | | | | | | | per 10 ug/m3 increases | Research | Y | N |
| (Yenerçağ and Sünter 2022) | Turkey | 4 | Samsun Province | PM2.5 | AQMS | 5 | Y | Y | Mortality | AirQ+ default | Local | Local | ST | Y | Y | Y | N | per 10 ug/m3 increases | Research | Y | N |
| (Hermayurisca 2021) | Thailand | 3 | 77 provinces in all 6 regions | PM2.5 | AQMS | 5 | N | Y, using the single imputation technique | All-cause-mortality, HA-R, HA-CVD, COPD, IHD, Strok, | RRs value from meta-analysis studies in Thailand and | Local | Local | LT/ST | Y | Y | Y | N | WHO, National standards and EPA standards | Research (Thesis) | Y | N |

| ID/ Author date | Country of study | WHO regions (AFR:1, AMR:2, SEAR: 3, EUR:4, EMR:5, WPR:6) | Name of city | Air quality | | | | | Health endpoints | RR Sources | BI Sources | BI Sources (Categorized) | Type of exposure (Long-term (LT)/ Short-term (ST)) | Number of cases reported (Yes/No) | PAF reported (Yes/No) | CI reported (Yes/No) | Sensitivity analyses (Yes/No) | Cut-off value (µg/m³) to estimate health effects | Motivation or context of study | Population Size reported (Yes/No) | use of life-tables module |
|---|---|---|---|---|---|---|---|---|---|---|---|---|---|---|---|---|---|---|---|---|---|
| | | | | Air Pollutants (APs) | AQMS or self-measured - Modeling - satellite | Type of montoring stations (Res:1/TR:2/BG:3/Ind:4/ None:5, Commercial:6) | Data Coverage reported (Yes/No) | Air quality data processing and validation reported (Yes/No) | | | | | | | | | | | | | |
| | | | | | | | | | | provided by WHO guideline in 2021 | | | | | | | | | | | |
| (KUMAR SINGH 2021) | India | 3 | Varanasi | PM10, SO2, NO2 | AQMS | 5 | N | N | All-cause death, HA-COPD, bronchitis, HA-CVD | AirQ+ default | WHO | WHO | LT/ST | Y | Y | Y | N | per 10 ug/m3 increases | Research (Thesis) | Y | N |
| (Egerstrom, Rojas-Rueda et al. 2023) | Australia, Brunei Darussalam, Cambodia, China, Fiji, Japan, Kiribati, Malaysia, Micronesia (Federated States of), Mongolia, New Zealand, Papua New Guinea, Philippines, Republic of Korea, Samoa, Singapore, Solomon Islands, Tonga, Vanuatu, Viet Nam | 6 | NR | PM2.5 | WHO Global Health Observatory | NR | NR | NR | Mortality all-cause | Previous studies | NR | NR | LT | Y | Y | Y | N | 5 and 10 | Research, strategy/policy planning | Y | N |

## References


Abdolahnejad, A., N. Jafari, A. Mohammadi, M. Miri, Y. Hajizadeh and A. Nikoonahad (2017). "Cardiovascular, respiratory, and total mortality ascribed to PM(10) and PM(2.5) exposure in Isfahan, Iran." J Educ Health Promot 6: 109.
Afghan, F. R. and S. K. Patidar (2020). "Health Impacts Assessment due to PM2.5, PM10 and NO2 Exposure in National Capital Territory (NCT) Delhi." Pollution **6**(1): 115-126.
Al-Hemoud, A., J. Gasana, A. N. Al-Dabbous, A. Al-Shatti and A. Al-Khayat (2018). "Disability Adjusted Life Years (DALYs) in Terms of Years of Life Lost (YLL) Due to Premature Adult Mortalities and Postneonatal Infant Mortalities Attributed to PM(2.5) and PM(10) Exposures in Kuwait." Int J Environ Res Public Health **15**(11).
Al-Hemoud, A., J. Gasana, A. Alajeel, E. Alhamoud, A. Al-Shatti and A. Al-Khayat (2021). "Ambient exposure of O(3) and NO(2) and associated health risk in Kuwait." Environ Sci Pollut Res Int **28**(12): 14917-14926.
Aliyu, Y. A. and J. O. Botai (2018). "An Exposure Appraisal of Outdoor Air Pollution on the Respiratory Well-being of a Developing City Population." J Epidemiol Glob Health **8**(1-2): 91-100.
Ambiente, P. C. N. d. (2007). "Valoración Económica de los Impactos de la Contaminación Atmosférica por PTS y PM10 en la Salud para Lima Metropolitana."
Amoatey, P., Y. O. Khaniabadi, P. Sicard, S. A. Siddiqi, A. De Marco and H. Sulaiman (2021). "Temporal Incidence and Prevalence of Bronchitis and Morbidities from Exposure to Ambient PM2.5 and PM10." Environmental Justice **14**(4): 267-276.
Amoatey, P., P. Sicard, A. de Marco and Y. O. Khaniabadi (2020). "Long-term exposure to ambient PM2.5 and impacts on health in Rome, Italy." Clinical Epidemiology and Global Health **8**(2): 531-535.
Amoatey, P., A. Takdastan, P. Sicard, P. K. Hopke, M. Baawain, H. Omidvarborna, S. Allahyari, A. Esmaeilzadeh, A. De Marco and Y. O. Khanaibadi (2019). "Short and long-term impacts of ambient ozone on health in Ahvaz, Iran." Human and Ecological Risk Assessment **25**(5): 1336-1351.
Ansari, M. and M. H. Ehrampoush (2019). "Meteorological correlates and AirQ(+) health risk assessment of ambient fine particulate matter in Tehran, Iran." Environ Res **170**: 141-150.
Asl, F. B., M. Leili, Y. Vaziri, S. S. Arian, A. Cristaldi, G. O. Conti and M. Ferrante (2018). "Health impacts quantification of ambient air pollutants using AirQ model approach in Hamadan, Iran." Environmental research **161**: 114-121.
Bahrami Asl, F., S. E. Amini Rabati, Y. Poureshgh, M. Kermani, M. E. Kalan, F. Hosseini, A. Dehghani and K. Taghi Livari (2022). "Ambient air pollutants and respiratory health outcomes in Tabriz and Urmia, two metropolises of Iran." Environmental Monitoring and Assessment **194**(11): 812.
Balidemaj, F., C. Isaxon, A. Abera and E. Malmqvist (2021). "Indoor Air Pollution Exposure of Women in Adama, Ethiopia, and Assessment of Disease Burden Attributable to Risk Factor." Int J Environ Res Public Health **18**(18).
Bartolacci (2012). "Valutazione dell'impatto sanitario del particolato fine e dell'ozono sui residenti nel comune di Ancona."
Barzeghar, V., P. Sarbakhsh, M. S. Hassanvand, S. Faridi and A. Gholampour (2020). "Long-term trend of ambient air PM10, PM2.5, and O3 and their health effects in Tabriz city, Iran, during 2006–2017." Sustainable Cities and Society **54**.
Bednářová, Z. (2007). "RETROSPEKTIVNÍ HODNOCENÍ ZDRAVOTNÍCH RIZIK PLYNOUCÍCH Z PŘÍTOMNOSTI POLUTANTŮ V ŽIVOTNÍM PROSTŘEDÍ ".
Begou, P. (2023). "Application of AirQ+ Software in the Attica Region, Greece: The Hospitalizations and Work Days Lost Attributed to Air Pollutants." Environmental Sciences Proceedings **26**(1): 36.
Behrouzi Rad, B., M. J. Mohammadi, S. Geravandi, A. R. Yari, S. Sadeghi and E. Zallaghi (2017). "Investigation of Years of Life Lost Caused by Dust Storm in Western Part of Iran." Archives of Hygiene Sciences **6**(2): 221-228.
Bešlić, Š. (2011). "Modelling of PM2.5 particle fraction concentration effects on mortalitiy in Zagreb and Republic of Croatia."
Bešlić, Š. a. (2006). "Health impact assessment of black smoke and airborne particulates on Zagreb population."
Bobvos (2014). "Health impact assessment of suspended particulate matter in some Hungarian cities."
Boldo, E., S. Medina, A. Le Tertre, F. Hurley, H.-G. Mücke, F. Ballester and I. Aguilera (2006). "Apheis: Health impact assessment of long-term exposure to PM2. 5 in 23 European cities." European journal of epidemiology **21**(6): 449-458.
Bologna, D. d. S. P. d. A. d. I. O. E. d. A. d. (2005). "Valutazione dell'impatto sanitario dell'inquinamento atmosferico da PM10 e Ozono nel territorio della AUSL di Imola 2003-2005 ".
Bonyadi, Z., H. Arfaeinia, M. Fouladvand, S. Farjadfard, M. Omidvar and B. Ramavandi (2020). "Impact of exposure to ambient air pollutants on the admission rate of hospitals for asthma disease in Shiraz, southern Iran." Chemosphere **262**: 128091.
Bonyadi, Z., M. H. Ehrampoush and M. T. Ghaneian (2016). "Health Impact Assessment of the Ambient PM2.5 Concentration in Mashhad, Iran, in 2013." Journal of Rafsanjan University of Medical Sciences **15**(5): 389-398.
Bonyadi, Z., M. H. Ehrampoush, M. T. Ghaneian, M. Mokhtari and A. Sadeghi (2016). "Cardiovascular, respiratory, and total mortality attributed to PM2.5 in Mashhad, Iran." Environ Monit Assess **188**(10): 570.
Borsi, S. H., G. Goudarzi, G. Sarizadeh, M. Dastoorpoor, S. Geravandi, H. A. Shahriyari, Z. A. Mohammadi and M. J. Mohammadi (2022). "Health Endpoint of Exposure to Criteria Air Pollutants in Ambient Air of on a Populated in Ahvaz City, Iran." Frontiers in Public Health **10**.
Bortoletto (2013). "Correlazione tra qualita' dell'aria e indicatori di salute analisi valutativa derivata dai valori di pm10 in quattro comuni della provincia di Treviso ".
Brito, J., A. Bernardo and L. L. Gonçalves (2022). "Atmospheric pollution and mortality in Portugal: Quantitative assessment of the environmental burden of disease using the AirQ+ model." Sci Total Environ **815**: 152964.
Campri (2011). "Valutazione dei rischi per la salute umana da esposizione ai criteria pollutants nell'area urbana di bologna."
Cassadou, S., P. Quénel, A. Zeghnoun, P. Saviuc, H. Prouvost, L. Pascal, C. Nunes, S. Medina, A. Le Tertre and L. Filleul (2003). "Évaluation de l 'impact sanitaire à court terme de la pollution atmosphérique urbaine: nouveaux résultats sur neuf villes françaises et utilité en santé publique." Environnement, Risques & Santé **2**(1): 19-25.
Chandra, I., K. Nisa and E. Rosdiana (2021). Preliminary study: Health risk analysis of PM2.5 and PM10 mass concentrations in Bandung Metropolitan. IOP Conference Series: Earth and Environmental Science.
Chardon, L. a. (2004). "APHEIS. Situation à Paris et proche couronne. Rapport de la troisième phase 2002-2003."
Daryanoosh, S. M., G. Goudarzi, A. Harbizadeh, H. Nourmoradi, A. A. Vaisi, H. Armin, S. Sadeghi and Y. Omidi Khaniabadi (2017). "Hospital Admission for Respiratory and Cardiovascular Diseases Due to Particulate Matter in Ilam, Iran." **9**(1): e36106.
Daryanoosh, S. M., G. Goudarzi, M. J. Mohammadi, H. Armin, Y. Omidi Khaniabadi and S. Sadeghi (2017). "Exposure to Particulate Matter and its Health Impacts (an AirQ Approach)." Archives of Hygiene Sciences **6**(1): 88-95.
De Marco, A., P. Amoatey, Y. O. Khaniabadi, P. Sicard and P. K. Hopke (2018). "Mortality and morbidity for cardiopulmonary diseases attributed to PM(2.5) exposure in the metropolis of Rome, Italy." Eur J Intern Med **57**: 49-57.
Dehghani, M. H., P. K. Hopke, F. B. Asghari, A. A. Mohammadi and M. Yousefi (2020). "The effect of the decreasing level of Urmia Lake on particulate matter trends and attributed health effects in Tabriz, Iran." Microchemical Journal **153**: 104434.
Dias (2008). "Avaliação de risco para a saúde humana associado a partículas inaláveis."
Dobaradaran, S., S. Geravandi, G. Goudarzi, E. Idani, S. Salmanzadeh, F. Soltani, A. R. Yari and M. J. Mohammadi (2016). "Determination of Cardiovascular and Respiratory Diseases Caused by PM10 Exposure in Bushehr, 2013." Journal of Mazandaran University of Medical Sciences **26**(139): 42-52.
Dragic, N., S. Bijelovic, M. Jevtic, R. Velicki and I. Radic (2021). "Short-term health effects of air quality changes during the COVID-19 pandemic in the City of Novi Sad, the Republic of Serbia." Int J Occup Med Environ Health **34**(2): 223-237.
Ebrahimi Ghadi, M., F. Qaderi and E. Babanezhad (2019). "Prediction of mortality resulted from NO 2 concentration in Tehran by Air Q+ software and artificial neural network." International Journal of Environmental Science and Technology **16**(3): 1351-1368.
Effatpanah, M., H. Effatpanah, S. Jalali, I. Parseh, G. Goudarzi, G. Barzegar, S. Geravandi, F. Darabi, N. Ghasemian and M. J. Mohammadi (2020). "Hospital admission of exposure to air pollution in Ahvaz megacity during 2010–2013." Clinical epidemiology and global health **8**(2): 550-556.
Egerstrom, N., D. Rojas-Rueda, M. Martuzzi, B. Jalaludin, M. Nieuwenhuijsen, R. So, Y.-H. Lim, S. Loft, Z. J. Andersen and T. Cole-Hunter (2023). "Health and economic benefits of meeting WHO air quality guidelines, Western Pacific Region." Bulletin of the World Health Organization **101**(2): 130.
Ekland, J., D. Olsson, B. Forsberg, C. Andersson and H. Orru (2021). "The effect of current and future maternal exposure to near-surface ozone on preterm birth in 30 European countries-an EU-wide health impact assessment." Environmental Research Letters **16**(5).

Eskandari, Z., H. Maleki, A. Neisi, A. Riahi, V. Hamid and G. Goudarzi (2020). "Temporal fluctuations of PM(2.5) and PM(10), population exposure, and their health impacts in Dezful city, Iran." J Environ Health Sci Eng **18**(2): 723-731.

Fallahizadeh, S. (2018). "Quantification of Health Effects of Ambient PM10 in Gachsaran City, Iran, in Year 2015, Using the AirQ Software." health system research **14**(1): 73-78.

Fard, R. F., F. D. Torkamani, A. H. Mahvi, M. Fahiminia, A. Koolivand, M. R. Hosseini, B. Eshrati, M. Fiore, A. O. Oskouei and N. Rezaei (2021). "Health effects from particulate air pollution in one of the industrial cities of Iran." Journal of Air Pollution and Health **6**(2): 135-148.

Faridi, S., M. Shamsipour, M. Krzyzanowski, N. Künzli, H. Amini, F. Azimi, M. Malkawi, F. Momeniha, A. Gholampour, M. S. Hassanvand and K. Naddafi (2018). "Long-term trends and health impact of PM2.5 and O3 in Tehran, Iran, 2006–2015." Environment International **114**: 37-49.

Fattore, E. and V. Paiano (2008). "Il rischio sanitario in relazione alla qualità dell'aria." Ricerca & Pratica **24**(6).

Fattore, E., V. Paiano, A. Borgini, A. Tittarelli, M. Bertoldi, P. Crosignani and R. Fanelli (2011). "Human health risk in relation to air quality in two municipalities in an industrialized area of Northern Italy." Environmental research **111**(8): 1321-1327.

Forli'-Cesena, P. d. (2007). "Piano di gestione della qualita' dell'aria della provincia di Forli'-Cesena."

Fustel, E. A., T. M. Rueda, K. C. Contín, L. Lopez, I. A. Jiménez, S. T. Cárdenas, C. I. Hernandez, F. B. Diez, F. G. García and A. P. Taradach (2005). "Evaluación en cinco ciudades españolas del impacto en salud de la contaminación atmosférica por partículas. Proyecto Europeo APHEIS." Rev Esp Salud Pública **79**(2): 297-308.

Generale, D. (2008). "Valutazione Ambientale di Incidenza su Area Vasta della Centrale Turbogas di Modugno. Impatto sulla salute della popolazione.".

GERAVANDI, S. (2014). "Estimating the prevalence of cardiovascular and respiratory diseases due to particulate air pollutants in Tabriz air."

Geravandi, S. (2015). "The cardiovascular and respiratory deaths attributed to sulfur dioxide in Kermanshah."

Geravandi, S. (2015). "Estimation of deaths due to cardiovascular and respiratory diseases and the number of cases of myocardial infarction due to contact with sulfur dioxide in Ahwaz air Health effects due to exposure to sulfur dioxide ".

Geravandi, S., G. Goudarzi, S. Salmanzadeh, S. BeytMashal, M. Mohammadi and A. Naiemabadi (2016). "Determination of cardiovascular deaths and hospital admissions due to respiratory disease for particulate matter less than 10 microns exposure in Ahvaz city during 2010-2012." Journal of Rafsanjan University of Medical Sciences **14**(10): 853-864.

Geravandi, S., G. Goudarzi, F. Soltani, S. Dobaradaran, S. Salmanzadeh, S. Kamaei, A. Yari, F. Kalantar and M. Mohammadi (2016). "Sulfur dioxide pollutant and its effects on disease incidence and death among the citizens of Bushehr city." Iranian South Medical Journal **19**(4): 598-607.

Geravandi, S., G. Goudarzi, A. R. Yari, E. Idani, F. Yousefi, F. Soltani, M. J. Mohammadi, S. Salmanzadeh, R. Nashibi, M. Khishdost, R. Malihi and F. Kalantar (2016). "An estimation of COPD cases and respiratory mortality related to Ground-Level Ozone in the metropolitan Ahvaz during 2011." Archives of Hygiene Sciences **5**(1): 15-21.

Geravandi, S., G. R. Goudarzi, A. A. Babaei, A. Takdastan, M. J. Mohammadi, M. Vosoughi Niri, S. Salmanzadeh and E. Shirbeigi (2015). "HEALTH ENDPOINT ATTRIBUTED TO SULFUR DIOXIDE AIR POLLUTANTS." JUNDISHAPUR JOURNAL OF HEALTH SCIENCES **7**(3): 15-19.

GERAVANDI, S., M. J. MOHAMMADI, G. GOUDARZI, K. AHMADI ANGALI, A. K. NEISI and E. ZALAGHI (2015). "HEALTH EFFECTS OF EXPOSURE TO PARTICULATE MATTER LESS THAN 10 MICRONS (PM10) IN AHVAZ." JOURNAL OF INFLAMMATORY DISEASES (THE JOURNAL OF QAZVIN UNIVERSITY OF MEDICAL SCIENCES) **18**(5 (76)): -.

Geravandi, S., A. Neisi, G. Goudarzi, M. VousoghiNiri and M. Mohammadi (2015). "Estimation of cardiovascular and respiratory deaths related to ozone exposure in Ahvaz, during 2011." Journal of Rafsanjan University of Medical Sciences **13**(11): 1073-1082.

GERAVANDI, S., E. ZALAGHI, G. GOUDARZI, M. J. MOHAMMADI, A. A. BABAEI, A. R. YARI and M. NORIZADEH HADAD (2016). "EXPOSURE TO PARTICULATE MATTER OF LESS THAN 10 MICRONS AND ITS EFFECT ON RESPIRATORY AND CARDIOVASCULAR DISEASES IN ISFAHAN, IRAN IN 2013." HEALTH SYSTEM RESEARCH **11**(4): -.

Geravandia, S., G. Goudarzic, M. Vosoughid, S. Salmanzadehe, M. Mohammadib and E. Zallaghif (2015). Determination of behavior particulate matter less than 10 microns and effects on human health in Ahvaz, Southwest Iran. Arch Hyg Sci. 2015; 4 (2): 64-72.

Ghaffari, H. R., H. E. Aval, A. Alahabadi, A. Mokammel, R. Khamirchi, S. Yousefzadeh, E. Ahmadi, A. Rahmani-Sani, M. Estaji and A. Ghanbarnejad (2017). "Asthma disease as cause of admission to hospitals due to exposure to ambient oxidants in Mashhad, Iran." Environmental Science and Pollution Research **24**(35): 27402-27408.

Ghanbari Ghozikali, M., A. Borgini, A. Tittarelli, A. Amrane, M. Mohammadyan and H. B (2016). "Quantification of health effects of exposure to air pollution (PM10) in Tabriz, Iran." Global Nest Journal **Global NEST Journal, Vol 18**.

Ghanizadeh, G., R. Khoshniyat, F. Karimi, M. R. Haghshenas, E. Hamidi and M. Rahimi (2018). "Short-term effects of PM10 to increase rate of hospital admission cardiovascular and respiratory of Sanandaj, Iran during 2015." Iranian Journal of Health, Safety and Environment **5**(2): 957-965.

Gharehchahi, E., A. H. Mahvi, H. Amini, R. Nabizadeh, A. A. Akhlaghi, M. Shamsipour and M. Yunesian (2013). "Health impact assessment of air pollution in Shiraz, Iran: a two-part study." Journal of Environmental Health Science and Engineering **11**: 1-8.

Gholampour, A., R. Nabizadeh, S. Naseri, M. Yunesian, H. Taghipour, N. Rastkari, S. Nazmara, S. Faridi and A. H. Mahvi (2014). "Exposure and health impacts of outdoor particulate matter in two urban and industrialized area of Tabriz, Iran." Journal of Environmental Health Science and Engineering **12**(1): 1-10.

Ghozikali, M. G., A. Borgini, A. Tittarelli, A. Amrane, K. Naddafi, M. Mohammadyan, G. Goudarzi, R. Bono and B. Heibati (2015). "Quantification of the health effects of exposure to air pollution (NO2) in Tabriz, Iran."

Ghozikali, M. G., B. Heibati, K. Naddafi, I. Kloog, G. O. Conti, R. Polosa and M. Ferrante (2016). "Evaluation of chronic obstructive pulmonary disease (COPD) attributed to atmospheric O3, NO2, and SO2 using Air Q Model (2011–2012 year)." Environmental research **144**: 99-105.

Goel, A., P. Saxena, S. Sonwani, S. Rathi, A. Srivastava, A. K. Bharti, S. Jain, S. Singh, A. Shukla and A. Srivastava (2021). "Health Benefits Due to Reduction in Respirable Particulates during COVID-19 Lockdown in India." Aerosol and Air Quality Research **21**(5).

Goudarzi, G., S. Daryanoosh, H. Godini, P. Hopke, P. Sicard, A. De Marco, H. D. Rad, A. Harbizadeh, F. Jahedi and M. Mohammadi (2017). "Health risk assessment of exposure to the Middle-Eastern Dust storms in the Iranian megacity of Kermanshah." Public health **148**: 109-116.

Goudarzi, G., S. Geravandi, H. Foruozandeh, A. A. Babaei, N. Alavi, M. V. Niri, M. J. Khodayar, S. Salmanzadeh and M. J. Mohammadi (2015). "Cardiovascular and respiratory mortality attributed to ground-level ozone in Ahvaz, Iran." Environ Monit Assess **187**(8): 487.

Goudarzi, G., S. Geravandi, E. Idani, S. A. Hosseini, M. M. Baneshi, A. R. Yari, M. Vosoughi, S. Dobaradaran, S. Shirali and M. B. Marzooni (2016). "An evaluation of hospital admission respiratory disease attributed to sulfur dioxide ambient concentration in Ahvaz from 2011 through 2013." Environmental science and pollution research **23**(21): 22001-22007.

Goudarzi, G., S. Geravandi, M. Mohammadi, M. Vosoughi, K. Angali, E. Zallaghi, A. Neisi, S. Saeidimehr and B. Mohammadi (2015). "Total number of deaths and respiratory mortality attributed to particulate matter (PM <sub>10</sub> ) in Ahvaz, Iran during 2009." International Journal of Environmental Health Engineering **4**(1): 33-33.

GOUDARZI, G., S. GERAVANDI, M. J. MOHAMMADI, S. SALMANZADEH, M. Vosoughi and M. SAHEBALZAMANI (2015). "The relationship between air pollution exposure and chronic obstructive pulmonary disease in Ahvaz, Iran." CHRONIC DISEASES JOURNAL **3**(1): -.

Goudarzi, G., S. Geravandi, S. Saeidimehr, M. Mohammadi, N. M. Vosoughi, S. Salmanzadeh, A. K. AHMADI, A. Neisi and A. Babaei (2015). "Estimation of health effects for PM10 exposure using of Air Q model in Ahvaz City during 2009."

Goudarzi, G., S. Geravandi, S. Salmanzadeh, M. . Mohammadi and E. Zallaghi (2014). "The Number of Myocardial Infarction and Cardiovascular Death Cases Associated with Sulfur Dioxide Exposure in Ahvaz, Iran." Archives of Hygiene Sciences **3**(3): 112-119.

Goudarzi, G., S. Geravandi, M. Vosoughi, M. j. Mohammadi, A. neisi and S. sadat Taghavirad (2014). "Cardiovascular deaths related to Carbon monoxide Exposure in Ahvaz, Iran." Iranian Journal of Health, Safety and Environment; Vol 1, No 3 (2014).

Goudarzi, G., P. K. Hopke and M. Yazdani (2021). "Forecasting PM2.5 concentration using artificial neural network and its health effects in Ahvaz, Iran." Chemosphere **283**.

Goudarzi, G., M. Mohammadi, A. K. AHMADI, B. Mohammadi, Z. Soleimani, A. Babaei, A. Neisi and S. Geravandi (2013). "ESTIMATION OF NUMBER ESTIMATION OF NUMBER OF CARDIOVASCULAR DEATH, MYOCARDIAL INFARCTION AND CHRONIC OBSTRUCTIVE PULMONARY DISEASE (COPD) FROM NO2 EXPOSURE USING AIR Q MODEL IN AHVAZ CITY DURING 2009."

Goudarzi, G., M. J. Mohammadi, K. Ahmadi Angali, A. K. Neisi, A. A. Babaei, B. Mohammadi, Z. Soleimani and S. Geravandi (2012). "Estimation of Health Effects Attributed to NO2 Exposure Using AirQ Model." Archives of Hygiene Sciences **1**(2): 59-66.

Goudarzi, G., E. Zallaghi, A. Neissi, K. Ahmadi Ankali, A. Saki, B. Ali Akbar, N. Alavi and M. J. Mohammadi (2013). "Cardiopulmonary Mortalities and Chronic Obstructive Pulmonary Disease Attributed to Ozone Air Pollution." Archives of Hygiene Sciences **2**(2): 62-72.

Gupta, P., A. Jangid and R. Kumar (2019). "Measurement of PM10, PM2.5 and Black Carbon and Assessment of Their Health Effects in Agra, A Semiarid Region of India." Proceedings of the Indian National Science Academy **85**(3): 667-679.

Hadei, M., P. K. Hopke, S. S. Hashemi Nazari, M. Yarahmadi, A. Shahsavani and M. R. Alipour (2017). "Estimation of mortality and hospital admissions attributed to criteria air pollutants in Tehran Metropolis, Iran (2013–2016)." Aerosol and air quality research **17**(10): 2474-2481.

Hadei, M., S. S. H. Nazari, M. Yarahmadi, M. Kermani, M. Farhadi and A. Shahsavani (2017). "Estimation of Gender-Specific Lung Cancer Deaths due to Exposure to PM2.5 in 10 Cities of Iran During 2013 - 2016: A Modeling Approach." International Journal of Cancer Management **10**(8).

Hadei, M., A. Shahsavani, M. Krzyzanowski, X. Querol, M. Stafoggia, S. S. H. Nazari, A. J. Jafari, M. Yarahmadi, M. Kermani and A. Khosravi (2020). "Burden of mortality attributed to PM2.5 exposure in cities of Iran; contribution of short-term pollution peaks." Atmospheric Environment **224**.

Hajizadeh, Y., N. Jafari, F. Fanaei, R. Ghanbari, A. Mohammadi, A. Behnami, A. Jafari, M. Aghababayi and A. Abdolahnejad (2021). "Spatial patterns and temporal variations of traffic-related air pollutants and estimating its health effects in Isfahan city, Iran." J Environ Health Sci Eng **19**(1): 781-791.

Hajizadeh, Y., N. Jafari, A. Mohammadi, S. M. Momtaz, F. Fanaei and A. Abdolahnejad (2020). "Concentrations and mortality due to short- and long-term exposure to PM2.5 in a megacity of Iran (2014–2019)." Environmental Science and Pollution Research **27**(30): 38004-38014.

Hamid, V., G. Goudarzi, A. Neisi and M. Dastoorpoor (2019). "Health Impact Assessment of the Ambient PM2.5 Concentration in Karaj, Iran, during 2012-2015." Journal of Research in Environmental Health **5**(1): 65-76.

Hermayurisca, F. (2021). "The health impact assessment of particulate matter (PM 2.5) in Thailand."

Hopke, P. K., S. S. H. Nazari, M. Hadei, M. Yarahmadi, M. Kermani, E. Yarahmadi and A. Shahsavani (2018). "Spatial and temporal trends of short-term health impacts of PM2.5in Iranian cities; A modelling approach (2013–2016)." Aerosol and Air Quality Research **18**(2): 497-504.

Hosseini, G., A. Maleki, H. Amini, S. Mohammadi, M. S. Hassanvand, O. Giahi and F. Gharibi (2014). "Health impact assessment of particulate matter in Sanandaj, Kurdistan, Iran." Journal of Advances in Environmental Health Research **2**(1): 54-62.

Hwang, S. L., Y. C. Lin, K. Y. Hsiao, C. M. Lin and M. C. Chi (2020). "Spatiotemporal assessment of mortality attributable to ambient PM2.5 exposure in Taiwan during 2008-2015." Air Quality Atmosphere and Health **13**(2): 233-245.

Jafari, N., S. Nemati, Y. Hajizadeh and A. Abdolahnejad (2017). "Spatial analysis and attributable mortality to outdoor air pollutants in Isfahan." Journal of health research in community **2**(4): 11-25.

Javan, S., S. Rahdar, M. Miri, B. Djahed, H. Kazemian, Y. Fakhri, H. Eslami, R. A. Fallahzadeh, A. Gholizadeh and M. Taghavi (2021). "Modeling of the PM10 pollutant health effects in a semi-arid area: a case study in Zabol, Iran." Modeling Earth Systems and Environment **7**(1): 455-463.

Javanmardi, P., P. Morovati, M. Farhadi, S. Geravandi, Y. O. Khaniabadi, K. A. Angali, A. M. Taiwo, P. Sicard, G. Goudarzi and A. Valipour (2018). "Monitoring the impact of ambient ozone on human health using time series analysis and air quality model approaches." Fresenius environmental bulletin **27**(1): 533-544.

Jeong, S. J. (2013). "The impact of air pollution on human health in Suwon City." Asian journal of atmospheric environment **7**(4): 227-233.

Junk, J., A. Krein and A. Helbig (2009). "Mortality rates and air pollution levels under different weather conditions: An example from Western Europe." International Journal of Environment and Waste Management **4**(1-2): 197-212.

Kahraman, A. C. and N. Sivri (2022). "Comparison of metropolitan cities for mortality rates attributed to ambient air pollution using the AirQ model." Environ Sci Pollut Res Int **29**(28): 43034-43047.

Kamarehie, B., M. Ghaderpoori, A. Jafari, M. Karami, A. Mohammadi, K. Azarshab, A. Ghaderpoury, A. Alinejad and N. Noorizadeh (2017). "Quantification of health effects related to SO2 and NO2 pollutants by using air quality model." Journal of Advances in Environmental Health Research **5**(1): 44-50.

Kamarehie, B., M. Ghaderpoori, A. Jafari, M. Karami, A. Mohammadi, K. Azarshab, A. Ghaderpoury and N. Noorizadeh (2017). "Estimation of health effects (morbidity and mortality) attributed to PM10 and PM2.5 exposure using an Air Quality model in Bukan city, from 2015-2016 exposure using air quality model." Environmental Health Engineering and Management Journal **4**(3): 137-142.

Kanhai, G., J. N. Fobil, B. A. Nartey, J. V. Spadaro and P. Mudu (2021). "Urban Municipal Solid Waste management: Modeling air pollution scenarios and health impacts in the case of Accra, Ghana." Waste Manag **123**: 15-22.

Karimi, A., M. Shirmardi, M. Hadei, Y. T. Birgani, A. Neisi, A. Takdastan and G. Goudarzi (2019). "Concentrations and health effects of short- and long-term exposure to PM2.5, NO2, and O3 in ambient air of Ahvaz city, Iran (2014–2017)." Ecotoxicology and Environmental Safety **180**: 542-548.

KAVUNCUOĞLU, D., S. YILMAZ and Z. KOŞAN (2021). "Erzurum'da 2016-2018 yillarinda NO2 düzeylerinin mortalite üzerine etkisi." ESTÜDAM Halk Sağlığı Dergisi **6**(2): 114-122.

KERMANI, M., M. AGHAEI, F. BAHRAMIASL, M. GHOLAMI, J. S. FALLAH, M. DOLATI and S. KARIMZADEH (2016). "Estimation of cardiovascular death, myocardial infarction and chronic obstructive pulmonary disease (COPD) attributed to SO2 exposure in six industrialized metropolises of Iran."

Kermani, M., M. Aghaei and M. Dolati (2016). "Estimation the Number of Mortality Due to Cardiovascular and Respiratory disease, Attributed to pollutants O3, and NO2 in the Air of Tehran." Journal of health research in community **1**(4): 1-11.

KERMANI, M., M. AGHAEI, M. GHOLAMI, F. BAHRAMI ASL, S. KARIMZADEH, S. FALLAH JOKANDAN and M. DOWLATI (2016). "Estimation of mortality attributed to PM2. 5 and CO exposure in eight industrialized cities of Iran during 2011." IRAN OCCUPATIONAL HEALTH JOURNAL **13**(4 #r00361): -.

kermani, M., H. Arfaeinia, K. Masroor, A. Abdolahnejad, F. Fanaei, A. Shahsavani, M. Tahmasbizadeh and M. H. Vahidi (2020). "Health impacts and burden of disease attributed to long-term exposure to atmospheric PM10/PM2.5 in Karaj, Iran: effect of meteorological factors." International Journal of Environmental Analytical Chemistry.

Kermani, M., F. B. Asl, M. Aghaei, S. F. Jokandan, S. Karimzadeh and M. Dowlati (2016). "EVALUATION OF CHRONIC OBSTRUCTIVE PULMONARY DISEASE (COPD) ATTRIBUTED TO O3 AND NO2 IN SIX METROPOLITANS OF IRAN BY USING AIRQ MODEL." Journal of Air Pollution and Health **1**(4): 227-236.

KERMANI, M., K. AZARSHAB, M. DOWLATI and M. GHADERPOORI (2017). "A SURVEY OF AIR QUALITY INDEX AND QUANTIFICATION OF CARDIOVASCULAR MORTALITY DUE TO EXPOSURE TO PARTICULATE MATTER SMALLER THAN 2.5 MICRON IN BOUKAN IN 2015." JOURNAL OF ENVIRONMENTAL HEALTH ENGINERING **4**(4 #F00130): -.

Kermani, M., F. Bahrami Asl, M. Aghaei, S. Karimzadeh, H. Arfaeinia, G. Godarzi and S. Salahshour Arian (2015). "Quantification of Health Effects Attributed to Ozone in Five Metropolises of Iran Using AirQ Model." Journal of Health **6**(3): 266-280.

Kermani, M., M. Dowlati, S. Fallah Jokandan, M. Aghaei, F. Bahrami Asl and S. Karimzadeh (2017). "Evaluation of cardiovascular and respiratory mortality attributed to atmospheric SO2 and CO using AirQ model." Environmental Health Engineering and Management Journal **4**(2): 101-108.

Kermani, M., M. Dowlati, A. J. Jafari and R. Kalantari (2018). "Number of mortality, chronic obstructive pulmonary disease and acute myocardial infarction due to exposure to sulfur dioxide in Tehran, during 2005-2014."

Kermani, M., M. Dowlati, A. Joneidi Jafari and R. Rezaei Kalantari (2017). "Evaluation of Cardiovascular Death, Attributed to CO Exposure in Tehran Megacity in During a Five-Year Period (2010-2014) by Using AirQ Model." Journal of Environmental Health Engineering **4**(1): 82-92.

Kermani, M., M. Dowlati, A. Jonidi Jafari and R. Rezaei Kalantari (2016). "Estimation of mortality, acute myocardial infarction and chronic obstructive pulmonary disease due to exposure to O3, NO2, and SO2 in ambient air in Tehran." Journal of Mazandaran University of Medical Sciences **26**(138): 96-107.

Kermani, M., M. Dowlati, A. Jonidi Jafari, R. Rezaei Kalantari and F. Sadat Sakhaei (2016). "Effect of air pollution on the emergency admissions of cardiovascular and respiratory patients, using the air quality model: a study in Tehran, 2005-2014." Health in Emergencies and Disasters Quarterly **1**(3): 137-146.

Kermani, M., M. Dowlati, A. Jonidi jafari and R. Rezaei Kalantary (2016). "Study the number of cases cardiovascular mortality Attributed to CO in Tehran in during a five-year." Rahavard Salamat Journal **2**: 38-47.

Kermani, M., M. Dowlati, A. Jonidi jafari and R. Rezaei Kalantary (2017). "Estimation of Mortality and Morbidity due to Exposure to Respirable Particulate Matter (RPM) in the Air of Tehran in 2014-2015." Journal of Occupational and Environmental Health **2**: 301-310.

Kermani, M., M. Dowlati, A. Jonidi jafari and R. Rezaei Kalantary (2017). "Health impact caused by exposure to particulate matter in the air of Tehran in the past decade." Tehran University Medical Journal **74**: 885-892.

Kermani, M., M. Dowlati, A. Jonidi jafari and R. Rezaei Kalantary (2017). "Number of total mortality, cardiovascular mortality and Chronic Obstructive Pulmonary Disease due to exposure with Nitrogen dioxide in Tehran during 2005-2014." The Journal of Urmia University of Medical Sciences **28**: 22-32.

Kermani, M., M. Dowlati, A. Jonidi Jafari and R. Rezaei Kalantary (2018). "The association of hospital emergency admissions due to respiratory-cardiovascular diseases and acute myocardial infarction with air pollution in Tehran during 2005-2014." Medical Journal of the Islamic Republic Of Iran **32**(1): 440-445.

Kermani, M., M. Dowlati, A. Jonidi jafari, R. Rezaei Kalantary and F. Sakhaei (2018). "Study of number of total mortality, cardiovascular and Respiratory mortality attributed to air pollutants of Tehran in 2005-2014." Global Nest Journal **20**: 439–448.

Kermani, M., M. Dowlati, A. Jonidi Jafari and R. RezaeiKalantari (2017). "Evaluation the number of cases of mortality and hospitalization due to cardiovascular disease and Chronic Obstructive Pulmonary Disease (COPD) Attributed to ozone in the air of Tehran Megacity During past decade (2005-2015)." Journal of North Khorasan University of Medical Sciences **9**(1): 89-99.

Kermani, M., S. Fallah Jokandan, M. Aghaei, F. Bahrami Asl, S. Karimzadeh and M. Dowlati (2016). "Estimation of the number of excess hospitalizations attributed to sulfur dioxide in six major cities of Iran." Health Scope **5**(4): e38736.
KERMANI, M., M. GHOLAMI, M. AGHAEI, F. BAHRAMI ASL, A. SHAHSAVANI, S. KARIMZADEH and H. ARFAEINIA (2016). "QUANTIFICATION OF MORTALITY AND HOSPITAL ADMISSION DUE TO CARDIOVASCULAR AND RESPIRATORY DISEASE ATTRIBUTED TO PM<SUB>10</SUB> IN 7 METROPOLITANS OF IRAN DURING 2011-2012." HEALTH SYSTEM RESEARCH **13**(2): -.
Kermani, M., G. Goudarzi, A. Shahsavani, M. Dowlati, F. Bahrami Asl, S. Karimzadeh, S. Jokandan, M. Aghaei, B. Kakavandi, B. Rastgarimehr, S. Ghorbani-Kalkhajeh and R. Tabibi (2018). "Estimation of Short-term Mortality and Morbidity Attributed to Fine Particulate Matter in the Ambient Air of Eight Iranian Cities." Annals of Global Health **84**: 408-418.
Khaefi, M., S. Geravandi, G. Hassani, A. R. Yari, F. Soltani, S. Dobaradaran, S. Moogahi, M. J. Mohammadi, M. Mahboubi and N. Alavi (2017). "Association of particulate matter impact on prevalence of chronic obstructive pulmonary disease in Ahvaz, southwest Iran during 2009-2013." Aerosol and air quality research **17**(1): 230-237.
Khaniabadi, Y. O., M. Daryanoosh, P. Sicard, A. Takdastan, P. K. Hopke, S. Esmaeili, A. De Marco and R. Rashidi (2018). "Chronic obstructive pulmonary diseases related to outdoor PM(10), O(3), SO(2), and NO(2) in a heavily polluted megacity of Iran." Environ Sci Pollut Res Int **25**(18): 17726-17734.
Khaniabadi, Yusef O., S. M. Daryanoosh, P. K. Hopke, M. Ferrante, A. De Marco, P. Sicard, G. Oliveri Conti, G. Goudarzi, H. Basiri, M. J. Mohammadi and F. Keishams (2017). "Acute myocardial infarction and COPD attributed to ambient SO2 in Iran." Environmental Research **156**: 683-687.
Khaniabadi, Y. O., R. Fanelli, A. De Marco, S. M. Daryanoosh, I. Kloog, P. K. Hopke, G. O. Conti, M. Ferrante, M. J. Mohammadi, A. A. Babaei, H. Basiri and G. Goudarzi (2017). "Hospital admissions in Iran for cardiovascular and respiratory diseases attributed to the Middle Eastern Dust storms." Environ Sci Pollut Res Int **24**(20): 16860-16868.
Khaniabadi, Y. O., G. Goudarzi, S. M. Daryanoosh, A. Borgini, A. Tittarelli and A. De Marco (2017). "Exposure to PM10, NO2, and O3 and impacts on human health." Environmental science and pollution research **24**(3): 2781-2789.
Khaniabadi, Y. O., P. K. Hopke, G. Goudarzi, S. M. Daryanoosh, M. Jourvand and H. Basiri (2017). "Cardiopulmonary mortality and COPD attributed to ambient ozone." Environ Res **152**: 336-341.
Khaniabadi, Y. O., R. Polosa, R. Z. Chuturkova, S. M. Daryanoosh, G. Goudarzi, A. Borgini, A. Tittarelli, H. Basiri, H. Armin and H. Nourmoradi (2018). "Air pollution health impact assessment on total, cardiovascular, and respiratory mortality in Khorramabad, Iran (The AirQ Approach)." Process Saf. Environ. Prot.
Khorsandi, H., S. Karimzade, M. Aghaei, A. A. Aghapour, S. Mousavi Moghanjooghi and H. Kargar (2016). "Health Impact Assessment of Exposure to Particulate Matter less than 10 Micron and Sulfur Dioxide using AIRQ Model in Urmia, Iran." Studies in Medical Sciences **27**(5): 438-448.
Kliengchuay, W., W. Srimanus, R. Srimanus, N. Kiangkoo, K. Moonsri, S. Niampradit, S. Suwanmanee and K. Tantrakarnapa (2022). "The association of meteorological parameters and AirQ+ health risk assessment of PM2.5 in Ratchaburi province, Thailand." Scientific Reports **12**(1).
Kohzadi, S., A. Amini, B. Shahmoradi, S. Mohammadi and H. P. Shivaraju (2018). "Estimating the health effects of PM10 on human in Sanandaj City during 2010-2014 using AirQ model." Journal of Advances in Environmental Health Research **6**(2): 61-66.
Kuldeep, S. Sen, S. Sisodiya and A. K. Mathur (2022). Particulate Matter Associated Human Health Effects Determination through AirQ+ for Alwar, Rajasthan (India). ECS Transactions.
Kuldeep, S. Sisodiya and A. K. Mathur (2022). A Case Study on Human Health Risk Assessment Attributable to Particulate Matter at Kota Metropolis, Rajasthan (India). ECS Transactions.
Kumar, N. and A. Middey (2022). "Prognosis of boundary layer assimilative capacity over a landlocked urban district in India: A prelude to exposure risk assessment." Urban Climate **41**.
KUMAR SINGH, A. (2021). Air Pollution and Health Risk Assessment in Varanasi, NATIONAL INSTITUTE OF TECHNOLOGY KURUKSHETRA 136119.
Kumie, A., A. Worku, Z. Tazu, W. Tefera, A. Asfaw, G. Boja, M. Mekashu, D. Siraw, S. Teferra, K. Zacharias, J. Patz, J. Samet and K. Berhane (2021). "Fine particulate pollution concentration in Addis Ababa exceeds the WHO guideline value: Results of 3 years of continuous monitoring and health impact assessment." Environ Epidemiol **5**(3): e155.
Lak, S., M. Fazlzadeh and M. Ghanbari Ghozigoli (2016). "Quantification of health impacts of exposure to atmospheric PM2. 5 using AirQ model in Tabriz city." Journal of Occupational and Environmental Health **2**(3): 210-219.
Lam, Y. F., J. M. H. Chang, B. P. Y. Loo, H. S. Zhang, K. K. M. Leung and K. W. Axhausen (2022). "Screening Approach for Short-Term PM2.5 Health Co-Benefits: A Case Study from 15 Metropolitan Cities around the World during the COVID-19 Pandemic." Atmosphere **13**(1).
Leili, M., F. Bahrami Asl, M. Hesam, M. Molamahmoudi and S. Arian (2017). "Estimation of Diseases and Mortality Attibuted to Atmospheric NO2 and SO2 Using AirQ Model in Hamadan City, Iran." Avicenna Journal of Clinical Medicine **23**: 314-322.
Li, Y., X. G. Zhao, Q. Liao, Y. Tao and Y. Bai (2020). "Specific differences and responses to reductions for premature mortality attributable to ambient PM2.5 in China." Science of the Total Environment **742**.
Luo, H., Q. Guan, J. Lin, Q. Wang, L. Yang, Z. Tan and N. Wang (2020). "Air pollution characteristics and human health risks in key cities of northwest China." Journal of Environmental Management **269**: 110791.
Maji, K. J., A. K. Dikshit and R. Chaudhary (2017). "Human health risk assessment due to air pollution in the megacity Mumbai in India." Asian Journal of Atmospheric Environment **11**(2): 61-70.
Maji, K. J., A. K. Dikshit and A. Deshpande (2016). "Human health risk assessment due to air pollution in 10 urban cities in Maharashtra, India." Cogent Environmental Science **2**(1): 1193110.
Maji, K. J., A. K. Dikshit and A. Deshpande (2017). "Assessment of City Level Human Health Impact and Corresponding Monetary Cost Burden due to Air Pollution in India Taking Agra as a Model City." Aerosol and Air Quality Research **17**(3): 831-842.
Maji, K. J., A. K. Dikshit and A. Deshpande (2017). "Human health risk as a basis for prioritizing air quality monitoring stations in a megacity: a case study." Arabian Journal of Geosciences **10**(18): 394.
Malakootian, M. and A. Mohammadi (2020). "ESTIMATING HEALTH IMPACT of EXPOSURE to PM2.5, NO2 and O3 USING AIRQ+ MODEL in KERMAN, IRAN." Environmental Engineering and Management Journal **19**(8): 1317-1323.
Maleki, H., A. Sorooshian, G. Goudarzi, A. H. Nikfal and m. m. Baneshi (2016). "Temporal profile of PM10 and associated health effects in one of the most polluted cities of the world (Ahvaz, Iran) between 2009 and 2014." Aeolian Research **22**: 135-140.
Manoj, M. G., M. K. Satheesh Kumar, K. T. Valsaraj, S. K. Vijayan and T. Nishanth (2022). "Exacerbation of Fatality Rates Induced by Poor Air Quality Due to Open-Air Mass Funeral Pyre Cremation during the Second Wave of COVID-19." Toxics **10**(6).
Manojkumar, N., M. Manish Kumar, S. K. Somwanshi, M. Monish Raj and B. Srimuruganandam (2020). Estimation of PM2.5-Related Hospital Admissions and Its Monetary Burden in Hyderabad, India. Lecture Notes in Civil Engineering. **71:** 1-10.
Manojkumar, N. and B. Srimuruganandam (2021). "Health effects of particulate matter in major Indian cities." Int J Environ Health Res **31**(3): 258-270.
Manojkumar, N. and B. Srimuruganandam (2022). "Spatio-temporal health benefits attributable to PM(2.5) reduction in an Indian city." Int J Environ Health Res: 1-11.
Mansouri, A., Y. Sadeghi, A. Mohammadi, A. Azadi, B. Mansouri and J. Majidi (2018). "Assessment of urban air quality in Sanandaj city and attribution of cardiovascular- respiratory diseases, and preterm birth in 2015-16." Scientific Journal of Kurdistan University of Medical Sciences **23**.
Markandeya, P. K. Verma, V. Mishra, N. K. Singh, S. P. Shukla and D. Mohan (2021). "Spatio-temporal assessment of ambient air quality, their health effects and improvement during COVID-19 lockdown in one of the most polluted cities of India." Environ Sci Pollut Res Int **28**(9): 10536-10551.
Marquis (2005). "Faisabilité d'une évaluation d'impact sanitaire de la pollution atmosphérique urbaine à la réunion."
Marzouni, M. B., T. Alizadeh, M. R. Banafsheh, A. M. Khorshiddoust, M. G. Ghozikali, S. Akbaripoor, R. Sharifi and G. Goudarzi (2016). "A comparison of health impacts assessment for PM10 during two successive years in the ambient air of Kermanshah, Iran." Atmospheric Pollution Research **7**(5): 768-774.
Marzouni, M. B., M. Moradi, A. Zarasvandi, S. Akbaripoor, M. S. Hassanvand, A. Neisi, G. Goudarzi, M. J. Mohammadi, R. Sheikhi, M. Kermani, M. Shirmardi, A. Naimabadi, M. Gholami, S. P. Mozhdehi, M. Esmaeili and K. Barari (2017). "Health benefits of PM(10) reduction in Iran." Int J Biometeorol **61**(8): 1389-1401.
Matkovic, V., M. Mulić, S. Azabagić and M. Jevtić (2020). "Premature adult mortality and years of life lost attributed to long-term exposure to ambient particulate matter pollution and potential for mitigating adverse health effects in tuzla and lukavac, bosnia and herzegovina." Atmosphere **11**(10).
Mehmood, T., Z. Tianle, I. Ahmad and X. Li (2019). Integration of AirQ+ and particulate matter mass concentration to calculate health and ecological constraints in Islamabad, Pakistan. Proceedings of 2019 16th International Bhurban Conference on Applied Sciences and Technology, IBCAST 2019.
Milano, A. C. d. (2009). "Stima dell’impatto dell’inquinamento atmosferico su mortalita’e morbosita’nella citta’di Milano - anno 2007."

Miri, M., A. Alahabadi, M. H. Ehrampush, A. Rad, M. H. Lotfi, M. H. Sheikhha and M. J. Z. Sakhvidi (2018). "Mortality and morbidity due to exposure to ambient particulate matter." Ecotoxicol Environ Saf **165**: 307-313.
Miri, M., Z. Derakhshan, A. Allahabadi, E. Ahmadi, G. O. Conti, M. Ferrante and H. E. Aval (2016). "Mortality and morbidity due to exposure to outdoor air pollution in Mashhad metropolis, Iran. The AirQ model approach." Environmental research **151**: 451-457.
Miri, M., H. Ebrahimi Aval, M. H. Ehrampoush, A. Mohammadi, A. Toolabi, A. Nikonahad, Z. Derakhshan and A. Abdollahnejad (2017). "Human health impact assessment of exposure to particulate matter: an AirQ software modeling." Environ Sci Pollut Res Int **24**(19): 16513-16519.
Mirzaei, A., H. Tahriri and B. Khorsandi (2021). "Comparison between AirQ+ and BenMAP-CE in estimating the health benefits of PM2.5 reduction." Air Quality, Atmosphere and Health **14**(6): 807-815.
Modena, C. d. (2008). "Piano particolareggiato d'iniziativa pubblica "centro guida sicura"."
Mohammadi, A., A. Azhdarpoor, A. Shahsavani and H. Tabatabaee (2015). "Health impacts of exposure to PM 10 on inhabitants of Shiraz, Iran." Health Scope **4**(4).
Mohammadi, A., A. Azhdarpoor, A. Shahsavani and H. Tabatabaee (2016). "Investigating the Health Effects of Exposure to Criteria Pollutants Using AirQ2.2.3 in Shiraz, Iran." Aerosol and Air Quality Research **16**(4): 1034-1043.
Mohammadi, A., M. Faraji, S. Mousavi, S. Nemati, M. Momtaz, A. Abdolahnejad and M. Miri (2019). "Health effects of airborne particulate matter related to traffic in Urmia, north-west Iran." Journal of Air Pollution and Health **4**(2): 99-108.
Mohammadi, M., S. Geravandi, F. Darabpour, Y. Omidi Khaniabadi, E. Charkhloo, M. Mahboubi, Y. Tahmasebi Birgani, F. Gholami-borujeni, A. Yari, B. Hashemzadeh, A. Shahsavani, G. Goudarzi and G. Hassani (2017). "AN ANALYSIS ON CARDIOVASCULAR MORTALITY ATTRIBUTED TO CARBON MONOXIDE IN PEOPLE OVER 65 YEARS IN THE SOUTH WESTERN OF IRAN." Fresenius Environmental Bulletin **26**: 4082-4087.
Mohammadi Rouzbahani, M., M. J. Mohammadi, S. Geravandi, E. Zallaghi, A. Valipour, B. Rastegarimehr, A. Karimyan and A. R. Yari (2017). "Association of Health Effects of Air Pollution on Chronic Obstructive Pulmonary Disease (COPD)." Archives of Hygiene Sciences **6**(4): 333-340.
Mohammed, A. M., Y. H. Ibrahim and I. A. Saleh (2019). "Estimation of hospital admission respiratory disease cases attributed to exposure to SO2 and NO2 in two different sectors of Egypt." African Health Sciences **19**(4): 2892-2905.
Mohd Shafie, S. H., M. Mahmud, S. Mohamad, N. L. F. Rameli, R. Abdullah and A. F. Mohamed (2022). "Influence of urban air pollution on the population in the Klang Valley, Malaysia: a spatial approach." Ecological Processes **11**(1).
Mokhtari, M., N. Jafari, Y. Hajizadeh, A. Mohammadi, M. Miri and A. Abdollahnejad (2017). "Estimation of health effects of PM2. 5 exposure using Air Q model in Isfahan during 2013." Health and Development Journal **6**(1): 74-84.
MOKHTARI, M., M. MIRI, A. MOHAMMADI, H. KHORSANDI, Y. HAJIZADEH and A. ABDOLAHNEJAD (2015). "ASSESSMENT OF AIR QUALITY INDEX AND HEALTH IMPACT OF PM<SUB>10</SUB>, PM<SUB>2.5</SUB> AND SO<SUB>2</SUB> IN YAZD, IRAN." JOURNAL OF MAZANDARAN UNIVERSITY OF MEDICAL SCIENCES **25**(131): -.
Momtazan, M., S. Geravandi, B. Rastegarimehr, A. Valipour, A. Ranjbarzadeh, A. R. Yari, S. Dobaradaran, H. Bostan, M. Farhadi and F. Darabi (2018). "An investigation of particulate matter and relevant cardiovascular risks in Abadan and Khorramshahr in 2014–2016." Toxin reviews.
Moradi, M., M. Hadei, M. Yazdani, M. Goudarzi, Z. Baboli, Y. Tahmasebi Birgani, A. Neisi and G. Goudarzi (2021). "Effect of long-term exposure to PM(2.5) on years of life lost in a populated Middle Eastern city." Environ Geochem Health **43**(8): 3229-3235.
Moradi, M., A. Mokhtari, M. J. Mohammadi, M. Hadei and M. Vosoughi (2022). "Estimation of long-term and short-term health effects attributed to PM2.5 standard pollutants in the air of Ardabil (using Air Q + model)." Environmental Science and Pollution Research **29**(15): 21508-21516.
Motalleby, M., A. Mazaheri, M. Mosayebi and M. Takhtfiroozeh (2015). "Assessing Health Impacts of Air Pollution in Kashan 2011." Journal of Arak University of Medical Sciences **18**(5): 77-87.
Moustris, K. P., K. Ntourou and P. T. Nastos (2017). "Estimation of Particulate Matter Impact on Human Health within the Urban Environment of Athens City, Greece." Urban Science **1**(1): 6.
Naddafi, K., M. S. Hassanvand, M. Yunesian, F. Momeniha, R. Nabizadeh, S. Faridi and A. Gholampour (2012). "Health impact assessment of air pollution in megacity of Tehran, Iran." Iranian journal of environmental health science & engineering **9**(1): 1-7.
Naghan, D. J., A. Neisi, G. Goudarzi, M. Dastoorpoor, A. Fadaei and K. A. Angali (2021). "Evaluation of ozone, PM2.5 and NO2 concentrations and estimation of attributed health effects based on AirQ+ on residents of Ahvaz, Iran (2012-2018)." Journal of Air Pollution and Health **6**(4): 243-256.
Naghan, D. J., A. Neisi, G. Goudarzi, M. Dastoorpoor, A. Fadaei and K. A. Angali (2022). "Estimation of the effects PM2.5, NO2, O3 pollutants on the health of Shahrekord residents based on AirQ+ software during (2012–2018)." Toxicology Reports **9**: 842-847.
Namrata, J. and K. Drashti (2022). "Estimation of health impact using AirQ+ model attributed to surface ozone in sub-regions of Surat city, India." Journal of Air Pollution and Health **7**(3).
Nasir, A. H., R. Nawaz, R. Haider and M. A. Irshad (2022). "Modeling Air Pollution Health Risk for Environmental Management of an Internationally Important Site: The Salt Range (Kallar Kahar), Pakistan." Atmosphere **13**(1).
Negri, I. M. (2015). "Valutazione del rischio tossicologico-sanitario dovuto alle emissioni dell'impianto di lavorazione della kainite sulla popolazione del comune di Realmonte."
Nemati, F., A. Mahvi and S. M. Mohseni (2016). "Health Impacts Of No2 In Mashhad, Iran." Journal of Air Pollution and Health **1**(1): 15-20.
Nguyen, Y. L. T., T. D. Nghiem, A. T. Le, K. N. Duc and D. H. Nguyen (2021). "Emission characterization and co-benefits of bus rapid transit: A case study in Hanoi, Vietnam." Atmospheric Pollution Research **12**(8).
Nikoonahad, A., R. Naserifar, V. Alipour, A. Poursafar, M. Miri, H. R. Ghafari, A. Abdolahnejad, S. Nemati and A. Mohammadi (2017). "Assessment of hospitalization and mortality from exposure to PM(10) using AirQ modeling in Ilam, Iran." Environ Sci Pollut Res Int **24**(27): 21791-21796.
Nourmoradi, H., G. Goudarzi, S. M. Daryanoosh, F. Omidi-Khaniabadi, M. Jourvand and Y. Omidi-Khaniabadi (2015). "Health Impacts of Particulate Matter in Air using AirQ Model in Khorramabad City, Iran." Journal of Basic Research in Medical Sciences **2**(2): 44-52.
Nourmoradi, H., Y. Omidi Khaniabadi, G. Goudarzi, S. M. Daryanoosh, M. Khoshgoftar, F. Omidi and H. Armin (2016). "Air Quality and Health Risks Associated With Exposure to Particulate Matter: A Cross-Sectional Study in Khorramabad, Iran." **5**(2): e31766.
Ntourou, K., K. Moustris, M. Giannouli, P. Nastos and A. Paliatsos (2017). Estimation of hospital admissions respiratory disease attributed to PM10 exposure using the AirQ model within the greater Athens area. Perspectives on atmospheric sciences, Springer**:** 1105-1110.
Oliveira, M., C. Delerue-Matos, M. C. Pereira and S. Morais (2020). "Environmental Particulate Matter Levels during 2017 Large Forest Fires and Megafires in the Center Region of Portugal: A Public Health Concern?" Int J Environ Res Public Health **17**(3).
Omidi Khaniabadi, Y., S. Daryanoosh, A. Amrane, R. Polosa, P. Hopke, G. Goudarzi, M. Mohammadi, P. Sicard and H. Armin (2016). "Impact of Middle Eastern Dust storms on human health." Atmospheric Pollution Research **8**.
Omidi Khaniabadi, Y., P. Sicard, A. Omidi Khaniabadi, S. Mohammadinejad, F. Keishams, A. Takdastan, A. Najafi, A. De Marco and M. Daryanoosh (2019). "Air quality modeling for health risk assessment of ambient PM10, PM2. 5 and SO2 in Iran." Human and Ecological Risk Assessment: An International Journal **25**(5): 1298-1310.
Omidi Khaniabadi, Y., P. Sicard, A. Takdastan, P. Hopke, A. Taiwo, F. Khaniabadi and M. Daryanoosh (2018). "Mortality and morbidity due to ambient air pollution in Iran." Clinical Epidemiology and Global Health **7**.
Omidi, Y., G. Goudarzi, A. Mirza Heidari and S. M. Daryanoosh (2016). "Health impact assessment of short-term exposure to NO2 in Kermanshah, Iran using AirQ model." Environmental Health Engineering and Management Journal **3**(2): 91-97.
Organization), W. W. H. (2019). "Health impact of ambient air pollution in Serbia: a call to action. World Health Organization." Regional Office for Europe.
Orru, H. (2011). "Välisõhu kvaliteedi mõju inimeste tervisele – peentest osakestest tuleneva mõju hindamine kogu Eesti lõikes."
Orru, H. (2013). "Estimated health impacts of changes in air pollution exposure associated with the planned by-pass Förbifart Stockholm."
Orru, H. (2016). "Maapinnalähedase osooni õhusaaste ekspositsiooni analüüs ja tervisemõjude hinnang."
Orru, H., M. Kaasik, E. Merisalu and B. Forsberg (2009). "Health impact assessment in case of biofuel peat – Co-use of environmental scenarios and exposure-response functions." Biomass and Bioenergy **33**(8): 1080-1086.
Orru, H., A. Laukaitienė and I. Zurlytė (2012). "Particulate air pollution and its impact on health in Vilnius and Kaunas." Medicina **48**(9): 70.
Orru, H., H. Olstrup, J. Kukkonen, S. López-Aparicio, D. Segersson, C. Geels, T. Tamm, K. Riikonen, A. Maragkidou, T. Sigsgaard, J. Brandt, H. Grythe and B. Forsberg (2022). "Health impacts of PM(2.5) originating from residential wood combustion in four nordic cities." BMC Public Health **22**(1): 1286.
ÖZTÜRK, E. N. Y. and M. Öztürk (2023). "Evaluation of the effect of NO2 levels on mortality in four key cities of Türkiye between 2017-2019." Journal of Experimental and Clinical Medicine **40**(1): 66-71.

Pahrol, M. A., W. R. W. Mahiyuddin, R. Shaharudin, M. I. Mazeli, N. M. Sham, A. S. A. Shakor, M. T. Latif, M. Sahani, N. A. F. K. Zaman, K. D. Kanniah and N. Mohamed (2020). Assessment of population exposure to PM2.5 and long-term mortality in Malaysia. 40th Asian Conference on Remote Sensing, ACRS 2019: Progress of Remote Sensing Technology for Smart Future.
Paiano, F. a. (2008). "Valutazione del rischio sanitaria della popolazione dei Comuni di Mazzano e Rezzato in relazione alla qualità dell'aria."
Pala, K., N. Aykac and Y. Yasin (2021). "Premature deaths attributable to long-term exposure to PM(2.5) in Turkey." Environ Sci Pollut Res Int **28**(37): 51940-51947.
Palanbek Yavaş, S., A. E. Önal, S. Pacci and Ç. Çağlayan (2021). "Effect of particulate matter and nitrogen dioxide levels on chronic bronchitis incidence and mortality in marmara region: Retrospective cohort study." Turkiye Klinikleri Journal of Medical Sciences **41**(3): 225-233.
Panahi, A., F. Ommi and Y. Rashidi (2017). "Estimated economic damage of cardiac deaths in Tehran due to NO2 gas." Environmental Sciences **15**(1): 89-102.
Passi, A., S. M. Shiva Nagendra and M. P. Maiya (2021). "ASSESSMENT of INDOOR and OUTDOOR EXPOSURE to AIRBORNE PARTICLES in SUBWAY METRO STATIONS and THEIR ASSOCIATED HEALTH RISKS." WIT Transactions on Ecology and the Environment **252**(2021): 85-96.
Pérez (2008). "Evaluación preliminar del impacto de la contaminación atmosférica en la salud de la población de la ciudad de Cochabamba."
Pokorný (2009). "Vyhodnocení kvality ovzduší průmyslové zóny města Jihlavy a z něho vyplývajících zdravotních rizik."
Polato, P. (2010). "Sistema di sorveglianza inquinamento atmosferico e salute comune di Udine."
Prabhu, V., P. Singh, P. Kulkarni and V. Sreekanth (2022). "Characteristics and health risk assessment of fine particulate matter and surface ozone: results from Bengaluru, India." Environ Monit Assess **194**(3): 211.
Pública, I. d. S. (2002). "Evaluación del impacto de la contaminación atmosférica sobre la salud en Madrid (programa APHEIS)."
Puglia, A. (2007). "Profilo di salute-benessere di Ferrara."
Rameez, H., V. Varekar, N. Rai and H. Mishra (2020). Seasonal variations in human health impacts of PM10, PM2.5, SO2, and NO2 emissions from a solid waste disposal facility at Turbhe, Navi Mumbai. Lecture Notes in Civil Engineering. **57:** 527-540.
Ravindra, K., T. Singh and S. Mor (2022). "Preventable mortality attributable to exposure to air pollution at the rural district of Punjab, India." Environ Sci Pollut Res Int **29**(21): 32271-32278.
Rocchetti, M., and Mariottini (2010). "Valutazione di impatto sanitario del PM10 e dell'O3 in 16 comuni della regione Marche nel 2007 e nel 2008."
Rovira, J., J. L. Domingo and M. Schuhmacher (2020). "Air quality, health impacts and burden of disease due to air pollution (PM10, PM2.5, NO2 and O-3): Application of AirQ plus model to the Camp de Tarragona County (Catalonia, Spain)." Science of the Total Environment **703**.
Sacks, J. D., N. Fann, S. Gumy, I. Kim, G. Ruggeri and P. Mudu (2020). "Quantifying the public health benefits of reducing air pollution: Critically assessing the features and capabilities of WHO's AirQ+ and U.S. EPA's environmental benefits mapping and analysis program-community edition (BenMAP-CE)." Atmosphere **11**(5).
Saeedi, R., R. Khani Jazani, S. S. Khaloo, S. Amirkhani Ardeh, R. Fouladi-Fard and H. Nikukalam (2021). "Risk assessment of occupational and public exposures to airborne particulate matter arising from a subway construction site in Tehran, Iran." Air Quality, Atmosphere and Health **14**(6): 855-862.
Saki, H., G. Goudarzi, S. Jalali, G. Barzegar, M. Farhadi, I. Parseh, S. Geravandi, S. Salmanzadeh, F. Yousefi and M. J. Mohammadi (2020). "Study of relationship between nitrogen dioxide and chronic obstructive pulmonary disease in Bushehr, Iran." Clinical Epidemiology and Global Health **8**(2): 446-449.
Samek, L. (2016). "Overall human mortality and morbidity due to exposure to air pollution." International Journal of Occupational Medicine and Environmental Health **29**(3): 417-426.
Sasmita, S., D. B. Kumar and B. Priyadharshini (2022). "Assessment of sources and health impacts of PM10 in an urban environment over eastern coastal plain of India." Environmental Challenges **7**.
Scarnato, P., and Pipitone (2006). "Valutazione Sanitaria della Qualità dell'aria a Bologna – anno 2005."
Schneider (2006). "Abschätzung der gesundheitsauswirkungen von schwebestaub in Österreich."
Šega, K. (2006). "Health Impact Assessment of Air Pollution on Zagreb population."
Shahsavani, A., K. Naddafi, N. J. Haghighifard, A. Mesdaghinia, M. Yunesian, R. Nabizadeh, M. Arahami, M. Sowlat, M. Yarahmadi and H. Saki (2012). "The evaluation of PM10, PM2. 5, and PM1 concentrations during the Middle Eastern Dust (MED) events in Ahvaz, Iran, from april through september 2010." Journal of arid environments **77**: 72-83.
Sicard, P., Y. O. Khaniabadi, S. Perez, M. Gualtieri and A. De Marco (2019). "Effect of O-3, PM10 and PM2.5 on cardiovascular and respiratory diseases in cities of France, Iran and Italy." Environmental Science and Pollution Research **26**(31): 32645-32665.
Skotak, K. (2008). "Potential human health effects of PM10 exposure in Poland."
sl, C. r. (2003). "Contaminación atmosférica y efectos en salud. Resultados del proyecto apheis en el gran Bilbao 1998-2000."
Sokoty, L., M. Kermani, L. Janani, M. Dowlat, B. Hassanlouei and S. Rimaz (2020). "Estimation of cardiovascular and respiratory diseases attributed to PM10 using AirQ model in Urmia during 2011-2017." Medical Journal of the Islamic Republic of Iran **34**: 60.
Soleimani, M., N. Akbari, B. Saffari and H. Haghshenas (2021). "Estimation of economic costs of air pollution caused by motor vehicles in Iran (Isfahan)." Environ Sci Pollut Res Int **28**(47): 66535-66555.
Soleimani, M., N. Akbari, B. Saffari and H. Haghshenas (2022). "Health effect assessment of PM2.5 pollution due to vehicular traffic (case study: Isfahan)." Journal of Transport & Health **24**.
Sówka, I., A. Nych, D. Kobus, Y. Bezyk and M. Zathey (2019). Analysis of exposure of inhabitants of Polish cities to air pollution with particulate matters with application of statistical and geostatistical tools. E3S Web of Conferences.
Sówka, I., Ł. Pachurka, M. Przepiórka, W. Rogula-Kozłowska and A. Zwoździak (2016). "Ocena krótkoterminowego wpływu stężeń pyłu zawieszonego na zdrowie mieszkańców Wrocławia." Rocznik Ochrona Środowiska **18**(cz. 2): 603--615.
Spangl (2010). "Gesundheitsauswirkungen der PM2,5-Exposition – Steiermark."
Stivanello (2014). "Rapporto sulla valutazione sanitaria della qualità dell'aria a Bologna, anno 2013."
Tabasideh, S., A. Vaisi, A. Jafari, R. Rezaee, M. Safari, G. Hosseini and R. Ebrahimi (2017). "Health impact assessment of exposure to PM10 in Ilam city using Air Q software." Journal of Advances in Environmental Health Research **5**(4): 196-204.
Tabibzadeh, S. A. S., S. A. Hosseini, P. Mohammadi, A. Etminan and H. Norouzi (2022). "Quantification of Mortality Associated with Particulate Matter Using Air Q model in Ambient Air in Shiraz, Iran." Polish Journal of Environmental Studies **31**(1): 551-559.
Tahery, N., S. Geravandi, G. Goudarzi, H. A. Shahriyari, S. Jalali and M. J. Mohammadi (2021). "Estimation of PM(10) pollutant and its effect on total mortality (TM), hospitalizations due to cardiovascular diseases (HACD), and respiratory disease (HARD) outcome." Environ Sci Pollut Res Int **28**(17): 22123-22130.
Thishan Dharshana, K. G. (2008). "Ambient PM10 and respiratory illnesses in Colombo City, Sri Lanka." Journal of Environmental Science and Health, Part A **43**(9): 1064-1070.
Todorović, M. N., M. B. Radenković, S. F. Rajšić and L. M. Ignjatović (2019). "Evaluation of mortality attributed to air pollution in the three most populated cities in Serbia." International Journal of Environmental Science and Technology **16**(11): 7059-7070.
Tominz, R., B. Mazzoleni and F. Daris (2005). "Stima dei potenziali benefici sanitari della riduzione dell'inquinamento atmosferico da PM10 nella città di Trieste." Epidemiol Prev **29**(3-4): 149-155.
Tonne, C., S. Beevers, B. Armstrong, F. Kelly and P. Wilkinson (2008). "Air pollution and mortality benefits of the London Congestion Charge: spatial and socioeconomic inequalities." Occupational and Environmental Medicine **65**(9): 620-627.
Toolabi, A., Z. Bonyadi and B. Ramavandi (2022). "Health impacts quantification attributed to ambient particulate matter in the nearest Iranian city to the main dust source." Environmental Monitoring and Assessment **194**(9): 666.
Uapipatanakul (2009). "Monitoring, modelling and health impacts of air pollutants arising from the Maptaphut Industrial Estates, Thailand."
ULUTAŞ, K. (2022). "PREDICTION OF MORTALITY ATTRIBUTED TO NO2 AIR POLLUTANT IN SAKARYA BY USING AIRQ+ SOFTWARE FOR 2018 AND 2019." ESTÜDAM Halk Sağlığı Dergisi **7**(2): 315-325.
Vahedian, M., O. Garkaz, N. KHANJANI, M. MIRZAEE and A. KOOLIVAND (2020). "The Effect of Exposure to Ambient Air Pollutants on Cardiovascular Mortality in Arak, Iran." INTERNATIONAL CARDIOVASCULAR RESEARCH JOURNAL **14**(4): -.
Vahidi, M., F. Fanaei and M. Kermani (2020). "Long-term Health Impact Assessment of PM2.5 and PM10 Karaj, Iran." International Journal of Environmental Health Engineering **9**: 8.
Varol, G., B. Tokuc, S. Ozkaya and C. Caglayan (2021). "Air quality and preventable deaths in Tekirdag, Turkey." Air Quality Atmosphere and Health **14**(6): 843-853.
Vicentino, D. d. P. U. A. (2005). "Valutazione del rischio sanitario correlato all'inquinamento atmosferico nel territorio dell'Alto Vicentino – PM10."

Vitolo (2009). "Comitato per l'Inchiesta Pubblica nell'ambito della procedura di Valutazione dell'Impatto Ambientale el Progetto dell'Impianto di co-incenerimento a servizio degli stabilimenti cartari di Diecimo e Porcari della Ditta Cartiera Lucchese S.p.A.".
Xu, C., Z. Zhang, G. Ling, G. Wang and M. Wang (2022). "Air pollutant spatiotemporal evolution characteristics and effects on human health in North China." Chemosphere **294**: 133814.
Yaghmaeian, K., S. Ghobakhloo and S. Mazloomi (2018). "Evaluation of Health Effects of PM2.5 Air Pollutant on Semnan Air Quality in 2017." Journal of health research in community **4**(3): 20-33.
Yang, C.-M. and K. Kao (2013). "Reducing fine particulate to improve health: a health impact assessment for Taiwan." Archives of environmental & occupational health **68**(1): 3-12.
Yarahmadi, M., M. Hadei, S. S. H. Nazari, G. O. Conti, M. R. Alipour, M. Ferrante and A. Shahsavani (2018). "Mortality assessment attributed to long-term exposure to fine particles in ambient air of the megacity of Tehran, Iran." Environ Sci Pollut Res Int **25**(14): 14254-14262.
Yari, A. R., G. Goudarzi, S. Geravandi, S. Dobaradaran, F. Yousefi, E. Idani, F. Jamshidi, S. Shirali, M. Khishdost and M. J. Mohammadi (2016). "Study of ground-level ozone and its health risk assessment in residents in Ahvaz City, Iran during 2013." Toxin Reviews **35**(3-4): 201-206.
Yenerçağ, F. N. T. and A. T. Sünter (2022). "Effects of Air Pollution on Mortality and Morbidity in Samsun Province of Turkey." Middle Black Sea Journal of Health Science **8**(4): 624-638.
Yorifuji, T., E. Yamamoto, T. Tsuda and N. Kawakami (2005). "Health impact assessment of particulate matter in Tokyo, Japan." Archives of Environmental & Occupational Health **60**(4): 179-185.
Zabalaga, A. (2005). "Implementación de una metodología para evaluar el impacto de la contaminación atmosférica sobre la salud en la ciudad de Cochabamba."
Zallaghi, E., S. Geravandi, M. Nourzadeh Haddad, G. Goudarzi, L. Valipour, S. Salmanzadeh, B. Hashemzadeh, A. Zahedi, M. J. Mohammadi and F. Soltani (2015). "Estimation of Health Effects Attributed to Nitrogen Dioxide Exposure Using the AirQ Model in Tabriz City, Iran." **4**(4): e30164.
Zallaghi, E., G. Goudarzi, S. Geravandi and M. J. Mohammadi (2014). "Epidemiological Indexes Attributed to Particulates With Less Than 10 Micrometers in the Air of Ahvaz City During 2010 to 2013." **3**(4): e22276.
Zallaghi, E., G. Goudarzi, M. Nourzadeh Haddad, S. M. Moosavian and M. J. Mohammadi (2014). "Assessing theEffects of Nitrogen Dioxide in Urban Air on Health of West and Southwest Cities of Iran." **6**(4): e23469.
Zallaghi, E., G. Goudarzi, S. Sabzalipour and A. Zarasvandi (2021). "Effects of long-term exposure to PM2.5 on years of life lost and expected life remaining in Ahvaz city, Iran (2008–2017)." Environmental Science and Pollution Research **28**(1): 280-286.
Zallaghi, E., M. Shirmardi, Z. Soleimani, G. Goudarzi, M. Heidari-Farsani, G. Al-Khamis and A. Sameri (2014). "Assessment of health impacts attributed to PM10 exposure during 2011 in Kermanshah City, Iran." Journal of advances in environmental health research **2**(4): 242-250.
中井里史, 丸山隆太 and 福﨑有希子 (2022). "2000–2018 各年の NO2 濃度に基づく横浜市での定量的健康影響評価." 大気環境学会誌 **57**(4): 101-108.
